# Design, Synthesis, and Structural Evolution of Pseudo‐Natural Product IDO1 Inhibitors and Degraders

**DOI:** 10.1002/anie.202518753

**Published:** 2025-11-27

**Authors:** Xiu‐Fen Cheng, Belén Lucas, Philipp Lampe, Stefano Ugel, Suyuan Chen, Anke Unger, Matthias Bischoff, Soheila Rezaei Adariani, Kesava Reddy Naredla, Kamal Kumar, Annika Schmidt, Carsten Strohmann, Petra Janning, Raphael Gasper, María Lucas, Malte Gersch, Sonja Sievers, Vincenzo Bronte, Slava Ziegler, Herbert Waldmann

**Affiliations:** ^1^ Abteilung Chemische Biologie Max‐Planck‐Institut für Molekulare Physiologie Otto‐Hahn‐Straße 11 44227 Dortmund Germany; ^2^ Fakultät Chemie und Chemische Biologie Technische Universität Dortmund Otto‐Hahn‐Straße 6 44221 Dortmund Germany; ^3^ Compound Management and Screening Center Otto‐Hahn‐Straße 15 44227 Dortmund Germany; ^4^ Immunology Section Department of Medicine, University and Hospital Trust (AOUI) of Verona P.le L.A. Scuro, 10 Verona 37134 Italy; ^5^ Lead Discovery Center GmbH (LDC) Otto‐Hahn‐ Straße 15 44227 Dortmund Germany; ^6^ Instituto de Biomedicina y Biotecnología de Cantabria IBBTEC Universidad de Cantabria‐CSIC C/ Albert Einstein 22, PCTCAN Santander 39011 Spain; ^7^ Chemical Genomics Center Max‐Planck‐Institut für Molekulare Physiologie Otto‐Hahn‐ Straße 15 44227 Dortmund Germany

**Keywords:** Bicyclic monoterpenes, Biophysical characterization, Co‐crystallization insights, IDO1 inhibitors and Degraders, Pyrrolidine alkaloid

## Abstract

Terpenoid alkaloids are derived from the fusion of structurally diverse terpenoid‐ and alkaloid moieties. The biologically relevant chemical space defined by this unique natural product (NP) class may be explored beyond the limitations of biosynthetic pathways by means of the pseudo natural product (PNP) principle, i.e., by combination of NP fragments in different arrangements. We describe the design, synthesis and structural evolution of a monoterpene–pyrrolidine PNP collection obtained by functionalization and combination of bicyclic monoterpenes with pyrrolidine alkaloid‐derived fragments. Diverse fusion strategies led to the discovery of (‐)‐myrtenal‐pyrrolidine PNPs that are indoleamine‐2,3‐dioxygenase 1 (IDO1) inhibitors and degraders, termed iDegs. Structural fine‐tuning modulated both degradation and inhibition potencies. Co‐crystallization revealed that iDegs induce unprecedented changes in the C‐terminus of IDO1 which promote degradation. iDegs inhibited tumor growth in SKOV‐3 tumor‐bearing mice and led to prolonged survival, which promises to inspire novel medicinal chemistry programs aimed at IDO1 in different diseases.

## Graphical abstract

The combination of bicyclic monoterpene‐ and pyrrolidine‐alkaloid fragments yields novel pseudo‐natural products. Biological characterization revealed that these iDegs are inhibitors and degraders of indoleamine‐2,3‐dioxygenase.

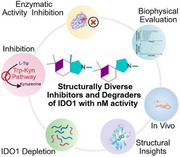

## Introduction

**Figure 1:**
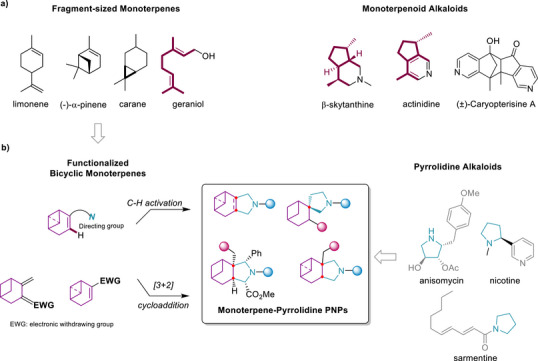
Design and strategy for the synthesis of a monoterpene‐pyrrolidone pseudo‐natural product collection. a) Structures of representative fragment‐sized monoterpenes and monoterpene alkaloids. b) Workflow for the synthesis of the monoterpene–pyrrolidine PNP collection.

Natural products (NPs) represent the fraction of biologically relevant chemical space explored by nature through evolution and serve as an invaluable source of bioactive small molecules for chemical biology and medicinal chemistry. Terpenes^[^ ^1^, ^2^, ^3^ ^]^ and alkaloids^[^ ^4^, ^5^ ^]^ two large and structurally diverse groups of natural products, cover a broad range of biological activities. However, terpene and alkaloid structures have been combined through biosynthesis in only a limited number of cases,^[^ ^6^ ^]^ primarily involving single alkaloid substructures such as indole, pyridine and piperidine. Tracing biosynthetic origins revealed that these terpenoid alkaloids and classical alkaloids have different biosynthetic pathways (Figure 1a).^[^ ^6^, ^7^ ^]^ Despite limited numbers and structural classes, terpenoid‐alkaloids possess pronounced biological activity.^[^ ^8^ ^]^ Consequently, the exploration of the biosynthetically hardly explored, biologically relevant chemical space defined by terpenoid‐alkaloids promises to yield novel compound classes endowed with diverse bioactivity.

The design, synthesis and biological evaluation of compound classes which embody unprecedented and biosynthetically not accessible combinations of NP fragments is at the heart of the pseudo‐natural product principle. Pseudo‐natural products (PNPs)^[^ ^9^, ^10^, ^11^, ^12^ ^]^ combine natural‐product fragments de novo in arrangements and combinations not observed in NPs and, thereby, go beyond limitations of natural evolution. The PNP approach retains the biological relevance of NPs through their respective fragments and gives access to new biologically relevant compound classes with unexpected and novel bioactivity and targets.

Inspired by the limited occurrence yet pronounced bioactivity of terpenoid alkaloids and guided by PNP design principles,^[^ ^11^, ^12^ ^]^ we synthesized a new PNP class in which the core scaffolds of fragment‐sized terpenes are fused with pyrrolidine‐alkaloid derived fragments (Figure 1b). Both NP fragments occur individually but their direct fusion has not been found in nature. Investigation of these novel PNPs in a cell‐based assay monitoring the kynurenine (Kyn) pathway led to the discovery of a new indoleamine‐dioxygenase 1 (IDO1) modulator chemotype.^[^ ^13^ ^]^

The Kyn pathway is of high relevance to immune‐suppression and diseases like neurodegeneration,^[^ ^14^, ^15^, ^16^, ^17^, ^18^ ^]^ auto‐immune diseases^[^ ^19^, ^20^, ^21^ ^]^ and cancers.^[^ ^22^, ^23^, ^24^ ^]^ The heme‐binding enzyme indoleamine‐2,3‐dioxygenase 1 (IDO1) catalyzes the first and rate‐limiting step in the conversion of tryptophan (Trp) to Kyn which leads to reduced T_eff_ cell proliferation and promotion of *T*_reg_ cell differentiation, linking IDO1 activity to reduced anti‐tumor immunity.^[^ ^25^, ^26^, ^27^, ^28^, ^29^ ^]^ In addition, the Epstein Barr virus (EBV) induces IDO1 expression, which is linked to EBV‐associated lymphoma.^[^ ^30^ ^]^ Furthermore, IDO1 expression is increased in amyloid and tau pathologies and associated with impaired spatial memory and synaptic plasticity.^[^ ^31^ ^]^ Clinical investigation of different IDO1 inhibitors has met with limited success,^[^ ^32^, ^33^ ^]^ possibly because IDO1 also has signaling functions not related to its enzymatic activity.^[^ ^33^, ^34^, ^35^, ^36^, ^37^, ^38^ ^]^ This limitation may be overcome by compounds that induce IDO1 degradation, thereby eliminating both enzymatic activity and signaling function, and this notion was supported by the development of IDO1‐directed PROTACs.^[^ ^39^, ^40^, ^41^ ^]^

We demonstrated that members of this monoterpene–pyrrolidine collection, i.e., a (‐)‐myrtanol‐derived PNP class, termed iDegs, reduce IDO1 protein levels by selectively targeting the heme‐free form of IDO1, inhibiting the enzymatic activity and inducing IDO1 protein degradation through the ubiquitin‐proteasome system by supercharging the native degradation pathway employing the cullin‐RING E3 ligase CRL2^KLHDC3^.^[^ ^13^ ^]^ Here we report the design and development of this monoterpene‐pyrrolidine collection and the structural evolution of the IDO1 inhibitors and degraders in the course of exploration of the structure‐activity relationship (SAR).

We report structural and stereochemical variation of the monoterpene fragment, investigation of fragment connectivity, exploration of substituent linkage to the scaffolds and variation of substituent structure. Biophysical characterization and structural analysis of several iDegs in complex with IDO1 revealed the key features determining the activity of this novel PNP class and identified the binding mode and critical IDO1 conformational changes induced by iDegs that govern inhibition and degradation potency. In addition, we describe, in vivo investigation of a representative iDeg in a mouse model. iDegs inhibited tumor growth in SKOV‐3 tumor‐bearing mice and led to prolonged survival, demonstrating efficacy in in vivo, which promises to inspire novel medicinal chemistry programs aimed at IDO1 in different diseases.

## Results and Discussion

### Design and Synthesis of Monoterpenoid‐Pyrrolidine Pseudo‐NPs

**Scheme 1:**
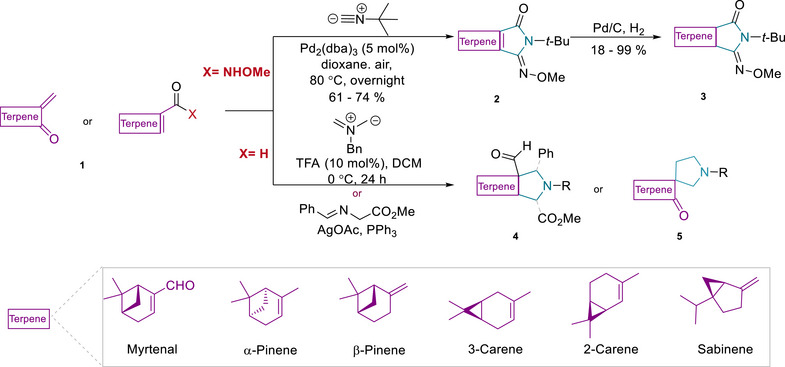
Synthesis of a monoterpene‐pyrrolidine pseudo‐natural product collection by means of C─H activation‐ and [3 + 2] cycloaddition reactions.

In the design of novel terpenoid‐pyrrolidine pseudo‐natural products, we envisaged to combine different fragment‐sized monoterpenes and the pyrrolidines by means of an *edge*‐ or *spiro*‐fusion (Scheme 1).^[^ ^11^, ^12^ ^]^ Bicyclic monoterpenes,^[^ ^42^ ^]^ characterized by their fused bicyclic structures and high enantiomeric purity, were preferentially employed as the combination partner. It was planned to employ either C─H activation or 1,3‐dipolar cycloaddition as key transformations to install pyrrolidine‐derived structures (Scheme 1), giving access to core scaffolds which would embody both fragments, and which would feature chemically differentiated functional groups enabling ready and broad structural variation of a respective compound class.

In the C‐H activation approach,^[^ ^43^, ^44^ ^]^ monoterpenes such as (+)‐ and (‐)‐α‐pinene, 2‐carene, were functionalized as *O*‐methylhydroxamic acids (**1a‐c**) (Figure S1a) serving as a directing group for regioselective C─H functionalization of an embedded olefin. 5‐Imino‐γ‐lactams (**2a‐c**) (Figure S1a) were obtained by reaction with *tert*‐butyl isonitrile followed by acyl migration and C─H activation in moderate to excellent yield (Scheme 1 and Figure S1a). After hydrogenation, the pinene‐pyrrolidine scaffolds (**3a‐c**) were obtained in up to quantitative yield (Scheme 1 and Figure S1a).

In the 1,3‐dipolar cycloaddition strategy, on the one hand (1*R*)‐(−)‐myrtenal (**1d**) (Figure S1b) was employed, which embodies an electron‐deficient alkene. The polar double bond facilitates regioselective control of the cycloaddition, and the stereocenters in the bridged four‐ and six‐membered rings in (1*R*)‐(−)‐myrtenal efficiently steered the stereoselective formation of the cycloadduct. Thus, (1*R*)‐(−)‐myrtenal was initially subjected to 1,3‐dipolar cycloaddition under different catalytic conditions, yielding the (‐)‐myrtenal‐pyrrolidine core structures **4a** and **4d** in excellent yields (Scheme 1, and Figure S1b). Specifically, for the synthesis of **4a**, an azomethine ylide, generated in situ from *N*‐(methoxymethyl)‐*N*‐(trimethylsilylmethyl)benzylamine, was employed in the presence of trifluoroacetic acid (TFA). Compound (**4a**) served as an intermediate *en route* to the pseudo‐NP collection. Alternatively, cycloaddition with an azomethine ylide derived from an amino acid ester imine yielded (‐)‐myrtenal‐pyrrolidine core structure (**4d**) featuring multi‐substituents with excellent yield. For further exploration of diverse monoterpenoid‐pyrrolidine scaffolds, bicyclic monoterpenes including 3‐carene, α‐pinene, β‐pinene and sabinene, and general cycloaliphatic rings were functionalized to introduce electron‐deficient alkenes (Figure S1b, compounds **1e‐h, 1i‐j**) to incorporate the pyrrolidine fragment via cycloaddition in yields of 70%–95% (Figure S1b, **4b‐c, 4e‐f, 5a‐b**). The *spiro*‐fused β‐pinene‐pyrrolidine core structure (**5a**) was obtained in 90% yield by employing functionalized β‐pinene (**1** **g**) in this 1,3‐dipolar cycloaddition (Figure S1b).^[^ ^45^, ^46^ ^]^

**Scheme 2:**
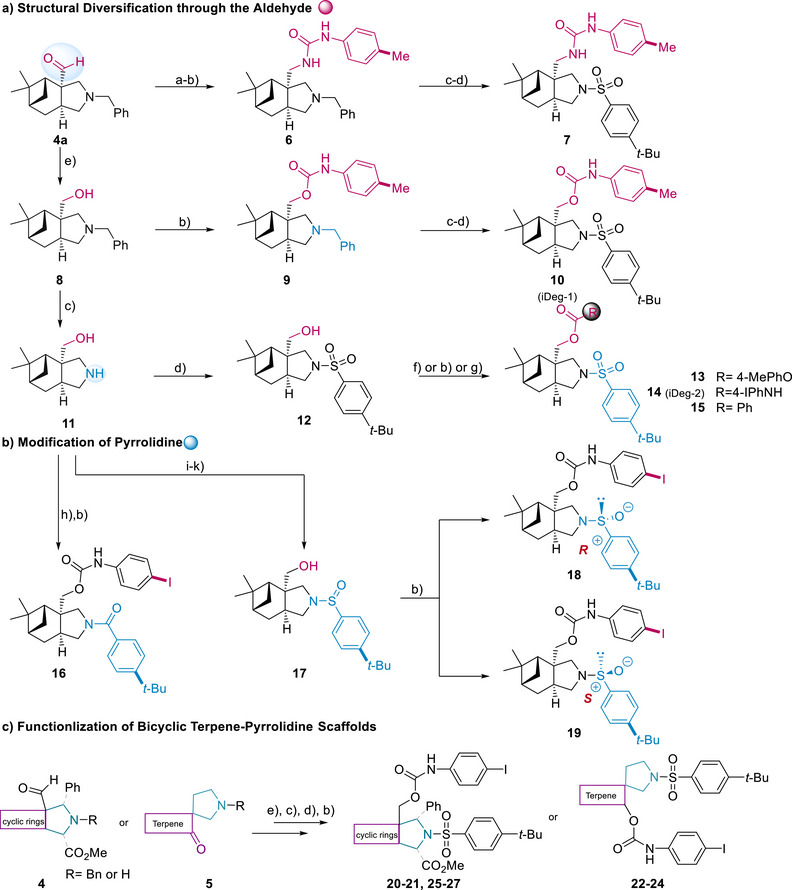
Modification of monoterpene‐pyrrolidine PNPs. a) Structural diversification through the aldehyde. (a) NH_3_‐*i*PrOH, Ti(O*i*Pr)_4_, ‐15 °C to rt, 4 h, then NaBH_4_, 30 min. 57% yield; (b) ArNCO (1.0 equiv.) toluene, ‐10 °C to rt, 24 h or NEt_3_, THF, rt, 2 h or DBU, THF, rt, 18 h. 50%–92% yield; (c) Pd/C, H_2,_ NH_4_HCO_3_, MeOH, 60 °C, 1 h or Pd/C, H_2_, EtOH/THF, rt, 18 h or Pd/C, H_2_, MeOH, rt, 16 h. 85%–95% yield; (d) 4‐*t*‐BuPhSO_2_Cl (1.0 equiv.), NEt_3_, DCM/THF, 0 °C to rt, 2 h or K_2_CO_3_, MeCN, 80 °C, 6 h. 45% to quant. yield; (e) NaBH_4_, MeOH/ MeOH:THF = 1, 0 °C to rt, 1 h. 85%–95% yield; (f) 4‐Me‐phenylchloroformate, pyridine, DCM, 0 °C to rt, overnight, 99% yield; (g) benzoyl chloride, NEt_3_, THF, 20 h. 98% yield; b) Modification of pyrrolidine: (h) 4‐(*tert*‐butyl)benzoyl chloride, NEt_3_, THF, 12 h. 80% yield; (i) TBSCl, NEt_3_, DMF, rt. overnight. 62% yield; (j) CuBr_2_, toluene, air, rt, 12 h. 52% yield; (k) TBAF, THF, rt, 2 h. 98% yield. c) Functionalization of bicyclic terpene‐pyrrolidine scaffolds.

For the synthesis of a compound collection, the aldehyde group incorporated in compound (**4a**) was converted to an amine with moderate yield via formation of an imine using NH_3_‐*i*PrOH, Ti(O*i*Pr)_4_, followed by reduction with NaBH_4_. Subsequent functionalization led to urea (**7**). Alternatively, reduction of the aldehyde group in compound (**4a**) by means of NaBH_4_ yielded primary alcohol (**8**), and hydrogenolytic removal of the benzyl group generated amino alcohol (**11**) in quantitative yield. These intermediates were further functionalized to form urethanes (**10**) and (**14**), carbonate (**13**) or ester (**15**) via reactions with isocyanates, chloroformates and acyl chlorides, respectively (Scheme 2a). Thus, the secondary amine of the pyrrolidine (**11**) was functionalized to sulfonamides (**14**), amide (**16**), or sulfinamide (**18**–**19**) via reactions with sulfonyl‐, acyl‐ or sulfenyl chloride (Scheme 2b), respectively with moderate to excellent yields. Additionally, structurally diverse monoterpene‐pyrrolidine scaffolds and general cycloaliphatic rings were also modified by urethane and sulfonamides and yielded compounds (**20**–**27**) (Scheme 2c and Figure S2).

**Figure 2:**
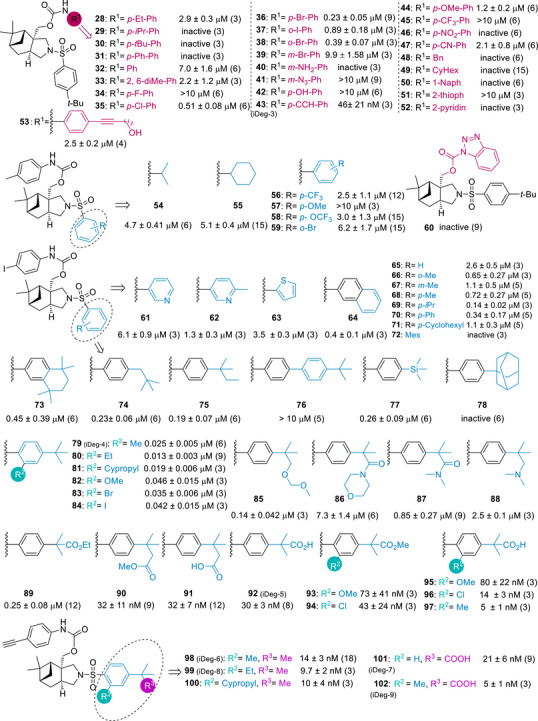
Structure‐activity relationship of myrtanol‐pyrrolidine PNPs (iDegs) for reduction of kynurenine levels. BxPC3 cells were incubated with IFN‐γ, L‐Trp and the compounds for 48 h prior to detection of Kyn levels. Data are mean values ± SD (biological replicates indicated in parenthesis). Inactive: > 75% residual Kyn levels observed at 10 µM; > 10 µM: Kyn levels of 50%–75% observed at 10 µM.

Following these general synthesis pathways, the urethane‐ and sulfonamide substituents attached to the myrtanol‐pyrrolidine PNP scaffold, as exemplified by compound (**8**) and (**11**), were varied. In total 75 compounds shown in Figure 2 were synthesized and subjected to biological analysis.

### Identification of a New Class of Kynurenine Pathway Inhibitors

To evaluate whether the novel terpenoid‐pyrrolidine PNPs show interesting or unexpected bioactivity, the collection was investigated in cell‐based assays monitoring kynurenine levels (Kyn assay), Hedgehog (Hh) signaling and autophagy. Notably, the (‐)‐myrtanol‐pyrrolidine derivative (**10** (iDeg‐1)) which carries a *p‐tert*‐butyl‐sulfonamide‐ and a *p‐*tolyl‐urethane reduced the levels of kynurenine that is generated by IDO1 in BxPC3 cells upon stimulation with the cytokine interferon gamma (IFN‐γ) (Figure S3a)^[^ ^47^ ^]^ with an IC_50_ of 0.83 ± 0.31 µM (Figure S3b). Furthermore, the analogue (**14** (iDeg‐2)) in which the methyl group in the urethane was replaced by an iodine inhibited Kyn formation with an IC_50_ of 0.12 ± 0.02 µM (Figure S3b), indicating that variation of the structure in this position increases bioactivity. Notably, the PNPs resulting from fusion of bicyclic monoterpenes with pyrrolidines (**3a‐c**) (Figure S3b) by means of C─H activation did not yield active compounds.

In order to explore the impact of scaffold structures, PNPs with *spiro*‐fusion of the fragments (**22**–**24**) and PNPs in which the terpene fragment was replaced with other bicyclic monoterpenes (**20**–**21**) or general cycloaliphatic rings (**25**–**26**) were investigated. *Spiro*‐fusion of β‐pinene with pyrrolidine yielded Kyn pathway inhibitor (**22**) which displayed an IC_50_ of 0.49 ± 0.06 µM (Figures S2 and S3b), whereas introduction of different monoterpenes such as 3‐carene (**21**), sabinene (**23**–**24**), as well as cyclohexyl‐ (**25**) or cyclopentyl‐ (**26**) groups into the fusion with pyrrolidine in different arrangements yielded inactive compounds. Enantiomer (**20**) of **14** (iDeg‐2) displayed 16‐fold lower activity indicating that the absolute configuration of the cycloadducts is important for activity (Figures S2 and S3b).

These results indicated that the myrtanol‐pyrrolidine fusion is the most favorable scaffold for further exploration of compound structure, and the attached functional groups were explored further. Replacement of the urethane group by a urea (**7**), a carbonate (**13**) or an ester (**15**), did not improve activity. Replacement of the phenylsulfonyl group with phenylsulfinyl analogs resulted in compounds (**18**) (IC_50 _= 0.15 ± 0.01 µM) and (**19**) (IC_50 _= 0.75 ± 0.06 µM), with compound (**18**) demonstrating comparable IC_50_ value to compound (**14** (iDeg‐2)). Replacement of the sulfonyl group with an *N*‐alkyl substituent (**9**) or a carbonyl group (**16**) abolished activity (Figure S3b).

### Structure‐Activity Relationship of Myrtanol‐Pyrrolidine PNPs

In order to determine how the key substituents of the myrtanol‐pyrrolidine PNP scaffold impact activity in Kyn pathway inhibition (Figure S3a), initially, the sulfonamide substituent of compound (**10** (iDeg‐1)) was kept constant and the urethane substituent R^1^ was varied (Figure 2, compounds **28**–**53**, **60**). Increase in size of the *para*‐substituent in the phenyl ring from methyl (**10**, iDeg‐1) to *tert*‐butyl and phenyl led to lower or to loss of activity (**10**, **28**–**31**), but omission of a *para*‐substituent (**32**, R^1^ = Ph) or combination with an *ortho*‐substituent (**33**, R^1^ = 2,6‐diMe‐Ph) was tolerated in principle. Replacement of the *para*‐CH_3_ by Cl (**35**), Br (**36**) or I (**14**, iDeg‐2) led to higher potency than (**10** (iDeg‐1)) which also increased with the size of the substituent, and loss of activity was detected with a smaller fluorine (**34**). Halogens (Br, I) were also tolerated in *ortho*‐position (**37**–**38**), but introduction of a halogen (Br) or a different substituent (NH_2_, N_3_) into the *meta*‐position led to loss of activity (**39**–**41**). Additional exploration of the *para*‐substituent (**42–47**, **53**) revealed that in this position limited structure variation is possible but may yield potent compounds. While ‐OH and a nitro group were not tolerated (**42, 46**), and ‐CF_3_ (**45**) and ‐CN‐substituents (**47**) reduced activity, introduction of an acetylene (**43**, iDeg‐3) substantially increased potency. Replacement of the phenyl group by alkyl substituents (**48**–**49**) or an annulated carbo‐ or heteroaromatic ring, such as thiophene and pyridine (**50**–**52, 60**) abolished activity. These investigations showed that for the urethane group, in the presence of a *p*‐*tert*‐butyl sulfonamide the most favorable substituents R^1^ are phenyl bearing a *p*‐methyl‐ (**10**, iDeg‐1), *p*‐iodo‐ (**14**, iDeg‐2) and *p*‐ethynyl‐substituent (**43**, iDeg‐3). Therefore, these substituted urethane groups were employed to explore variations of the sulfonamide substituents. Replacement of the *p*‐*tert*‐butyl group in the sulfonamide substituent of iDeg‐1 (**10**, R^1^ = *p*‐tolyl) by different substituents or exchange of the aromatic substituent to aliphatic analogs did not improve compound potency (**54**–**59**). However, exploration of sulfonamide substituents starting from iDeg‐2 (**14**, R^1^ = *p*‐iodophenyl) yielded more potent compounds. Exchange of the sulfonamide phenyl ring with pyridine or thiophene, as well as the *tert*‐butyl group in the *para* position of the sulfonamide phenyl ring by H or different aliphatic or aromatic compounds initially did not improve activity (**61**–**71**). An aliphatic ring (**73**), change to a neo‐amyl group (**74**) and addition of one methyl substituent to the *tert*‐butyl group (**75**) did not further increase the potency of iDeg‐2. However, notably, combination of the *p*‐*tert*‐butyl group with different *ortho*‐substituents enhanced activity by several fold (**79**–**84**), with methyl (**79**, iDeg‐4), ethyl (**80**) and cyclopropyl (**81**) displaying the highest potency. We further varied the *para*‐substituent by functionalizing the *tert*‐butyl group, initially in the absence of a further *ortho*‐substituent (**85**–**92**). A carboxyl group introduced instead of one of the methyl groups of the *p*‐*tert*‐butyl group in the sulfonamide phenyl ring resulted in a 4‐fold increase in compound potency (**92**, iDeg‐5) compared to iDeg‐2. We then combined different *ortho*‐substituents with a carboxylic acid replacing one methyl of the *p*‐*tert*‐butyl group (**93**–**97**). The combination of an *ortho*‐methyl group with a carboxylic acid group yielded the very potent compound (**97**) with an IC_50 _= 5 ± 1 nM.

Similar derivatization of iDeg‐3 (**43,** R^1^ = *para*‐CCH, Figure 2) yielded potent compounds with *ortho*‐substituted *para*‐*tert* butyl‐phenyl sulfonamide such as **98** (iDeg‐6), **99** (iDeg‐8) and **100**. Finally, also for acetylene‐substituted urethanes, replacement of a methyl group in the *p‐tert‐*butyl phenylsulfonamide by a carboxylic acid (**101**, iDeg‐7) and additional introduction of an *ortho*‐substituent (**102**, iDeg‐9) delivered potent compounds. Through this series of structure evolution, iDegs were identified that displayed activity in the cell‐based kynurenine assay in the low nanomolar range.

### Characterization of IDO1 Inhibition by iDegs

Reduction of the cellular Kyn level can be due to inhibition of the enzymatic activity of IDO1. Therefore, we selected key compounds functionalized with diverse features that exhibited submicromolar to nanomolar activity in the cellular Kyn assay and evaluated them in an in vitro assay monitoring the enzymatic activity of recombinant human IDO1 (Table 1 and Figure S4).

**Table 1:** Inhibition of in vitro IDO1 enzymatic activity by selected compounds.

	Cpd ^#^	**10** (iDeg‐1)	**14** (iDeg‐2)	**18**	**19**	**22**	**43** (iDeg‐3)	**79** (iDeg‐4)	80	81
IDO1 activity	Inhibition (%)a^)^	ns	ns	ns	ns	ns	ns	25 ± 5	35 ± 8	39 ± 1
	IC_50_ [µM]b^)^	–	–	–	–	–	–	>14	>14	>14

	**Cpd ^#^**	**82**	**83**	**84**	**91**	**92** (iDeg‐5)	**93**	**94**	**95**	**96**
IDO1 activity	Inhibition (%)*^a)^*	ns	ns	16 ± 4	59 ± 2	66 ± 8	ns	ns	57 ± 3	62 ± 1
	IC_50_ [µM]b^)^	–	–	–	1.9 ± 0.5	0.82 ± 0.14	–	–	0.67 ± 0.1	0.19 ± 0.1

	**Cpd ^#^**	**97**	**98** (iDeg‐6)	**99** (iDeg‐8)	**100**	**101** (iDeg‐7)	**102** (iDeg‐9)	Linrodostat	Epacadostat
IDO1 activity	Inhibition (%)^a)^	65 ± 4	40 ± 7	61 ± 1	39 ± 7	75 ± 4	64 ± 1	70 ± 5	91 ± 1
	IC_50_ [µM]*^b^*	0.11 ± 0.02	>14	4.0 ± 3.3	>14	0.31 ± 0.06	0.089 ± 0.03	0.036 ± 0.01	0.15 ± 0.03

For the analysis of IDO1 enzymatic activity, recombinant human IDO1 protein was pre‐incubated with the compounds at 37 °C for 45 min prior to detection of Kyn levels using a Kyn sensor.

^a)^ In vitro inhibition at a concentration of 14 µM. Data are mean values ± SD with three biological replicates unless otherwise noted in Figure S4. “ns”: not significant; “‐”: Inactive: >75% Kyn level observed up to a concentration of 14 µM and no IC_50_ value obtained.

^b)^ IC_50_ values were determined if inhibition at 14 µM was higher than 50%.

iDeg‐1 (**10**) and ‐2 (**14**) both embodying a *tert*‐butyl phenylsulfonamide and a *para*‐methyl or *para*‐iodo‐phenyl urethane, respectively, were not active at concentrations up to 14 µM when incubated with IDO1 for 45 min (Table 1). The phenylsulfinyl analogs **18**–**19** and *spiro*‐fused compound **22**, both showing activity comparable to iDeg‐2 in the cellular Kyn assay, also displayed no activity at 14 µM. iDeg‐4 (**79**)**, 80** and **81** (*ortho*‐alkyl‐*para*‐*tert*‐butyl‐phenyl sulfonamide combined with iodo‐phenyl urethane) were weak inhibitors. Replacement of one methyl group in the *tert*‐butylphenyl sulfonamide in iDeg‐2 (**14**) by a carboxylic acid yielded more potent inhibitors, including (**91**) and iDeg‐5 (**92**) with IC_50_ value of 1.9 ± 0.5 and 0.82 ± 0.14 µM, respectively (Table 1). Introduction of a further ortho‐substituent into the sulfonamide aryl group improved inhibitory activity (**95**–**97**) with compound (**97**) displaying an IC_50_ of 0.11 ± 0.02 µM. This also holds true for iDeg‐7 (**101**) and ‐9 (**102**), which demonstrated inhibition potencies comparable to that of the potent apo‐IDO1 inhibitor linrodostat,^[^ ^48^ ^]^ which displaces heme, and the holo‐IDO1 inhibitor epacadostat,^[^ ^49^ ^]^ a potent and reversible oxygen‐competitive IDO1 inhibitor. Both compounds have been extensively investigated in clinical trials (Table 1).^[^ ^50^, ^51^, ^52^ ^]^ iDeg‐9 (**102**) exhibited the best IC_50_ value of 0.089 ± 0.03 µM. Compounds (**101**–**102**) embody an alkynyl‐phenyl urethane and an *ortho*‐substituted carboxy‐bearing *para*‐*tert*‐butyl phenyl sulfonamide (Table 1, and Figure S4).

### iDegs Promote IDO1 Degradation in Cells

**Figure 3:**
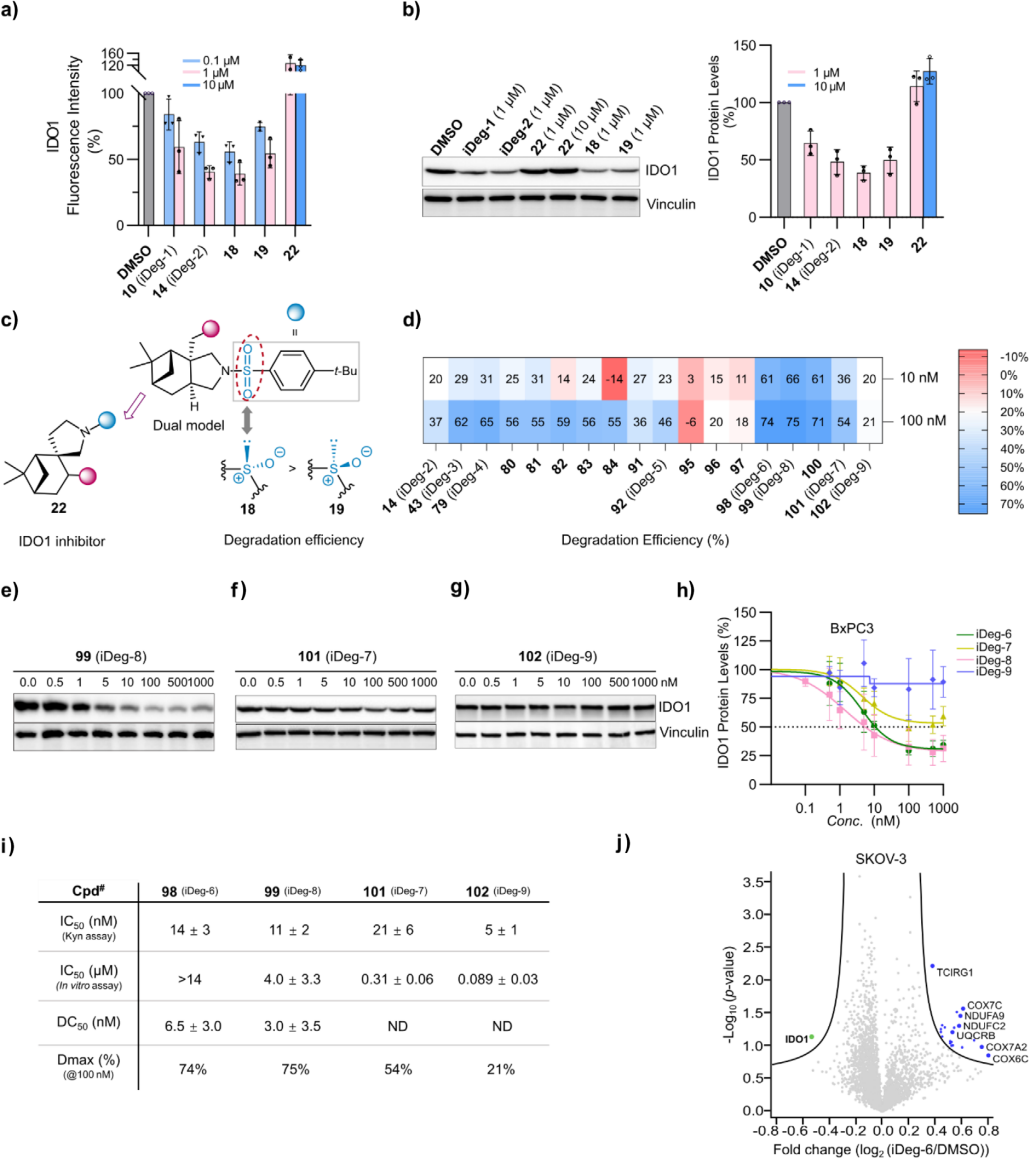
Comparison of IDO1 degradation efficiency for selected compounds. a) Detection of IDO1 levels using immunostaining. IFN‐γ stimulated HeLa cells were treated with the compounds for 24 h. IDO1 protein abundance was detected with IDO1 antibody. Data are mean values ± SD, *n* = 3. b) IDO1 protein levels in BxPC3 cells upon compounds treatment were detected using immunoblotting. BxPC3 cells were treated with IFN‐γ for 24 h prior to washout, followed by addition of compounds. Representative blot and quantified band intensities are shown (mean values ± SD, *n* = 3). See Figure S11a S12a for the uncropped blots. c) Key functions of hit compounds that influence degradation efficiency. d) Heatmap for IDO degradation efficiency. IDO1 levels were monitored as described in (c). See Table S1 for mean values and Figure S11b for the uncropped blots. e)–g) Dose dependent degradation of IDO1 protein in BxPC3 cells treated with iDeg‐7–9 and detected as described in (c). Representative data of *n* = 3 except for iDeg‐9 (*n* = 4). See Figure S12 S13 for the uncropped blots. h) Quantification of IDO1 protein levels from e‐g (mean values ± SD, *n* = 3 except for iDeg‐9 (*n* = 4). i) Tabulated inhibition and degradation ability of iDeg‐6–9. ND: not detected. j) Volcano plot of iDeg‐6‐induced changes on the global proteome. SKOV‐3 cells were treated with 10 µM iDeg‐6 or DMSO for 4 h followed by protein extraction, tryptic digest and LC‐MS/MS analysis (*n* = 4). Plot generated using UMSAP (*T*_0_ = 0.5, *S*_0_ = 0.5).

iDeg‐1 (**10**) and iDeg‐2 (**14**) effectively reduced Kyn levels in cells, but did not inhibit IDO1 activity in vitro under the used conditions. Therefore, we monitored IDO1 protein levels using immunostaining in HeLa cells upon induction of IDO1 expression with IFN‐γ (Figures 3a and S5a). Treatment of stimulated HeLa cells with iDeg‐1 and iDeg‐2 reduced the fluorescence intensity of the IDO1 staining. (Figures 3a and S5b). Notably, compounds with phenylsulfinyl group (**18** and **19**) also decreased the intensity of the IDO1 staining, akin to iDeg‐2 bearing a phenylsulfonyl group. In contrast, *spiro*‐fused compound (**22**) did not induce IDO1 protein degradation (Figures 3a and S5b). Reduced IDO1 protein levels in cells were detectable using immunoblotting, further confirming that both the phenylsulfonyl group (**10**, iDeg‐1 and **14**, iDeg‐2) and phenylsulfinyl functionalized compounds (**18, 19**) modulate IDO1 abundance while *spiro*‐fused compound (**22**) did not reduce the IDO1 protein level (Figure 3b,c). Overall, *spiro*‐fusion led to the discovery of IDO1 inhibitor while *edge*‐fusion induces IDO1 inhibition and degradation (Figure 3c). We further investigated compounds bearing both phenyl sulfonamide and phenyl urethane moieties with nanomolar activity in the Kyn assay (Figures 2 and  3d; Table S1). iDeg‐3 (**43**; *tert*‐butyl‐phenyl sulfonamide combined with acetylene‐phenyl urethane) was three‐fold more potent than iDeg‐2 in the Kyn assay and exhibited superior degradation efficiency (62%) compared to iDeg‐2 (37%) at a concentration of 100 nM (Figure 3d and Table S1, entries 1–2). Variation of the *ortho*‐substituent (**79**–**82**) in the *para*‐*tert*‐butylphenyl sulfonamide of iDeg‐2 increased both, enzyme inhibition and protein degradation (Figure 3d; Tables 1 and S1, entries 3–6, Dmax 55–65% at 100 nM). Compounds (**91**–**92** and **101**), bearing a carboxylic acid in the *para*‐substituted phenyl sulfonamide, were potent inhibitors of Kyn production in cells and in vitro, but only weakly to moderately induced IDO1 degradation (Table S1, entries 9–10, 17, Dmax of 36–54% at 100 nM). Compounds (**95**–**97** and **102**) which introduced additional *ortho*‐substituent into the sulfonamide aryl group bearing a carboxylic acid further decreased degradation efficiency (Figure 3d and Table S1, entries 11–13, 18). Notably, removal of the carboxylic acid moiety and introduction of a methyl‐, ethynyl‐ or cyclopropyl group as *ortho*‐substituent into the sulfonamide aryl group resulted in the highest degradation efficiency, as observed for iDeg‐6 (**98**, Table S1, entry 14, Dmax = 74% at 100 nM), iDeg‐8 (**99**, Dmax = 75% at 100 nM, entry 15, Table S1), and their close analog (**100**) (Dmax = 71% at 100 nM, entry 16, Table S1). These compounds, which embody an acetylene‐substituted phenyl urethane and an *ortho*‐aliphatic substituted *para*‐*tert*‐butylphenyl sulfonamide, also exhibited high degradation activity at 10 nM (**98**–**100**, Dmax up to 66% at 100 nM, Figure 3d; entries 14–16, Table S1). Dose‐response of iDeg‐8 reached a half‐maximal degradation concentration (DC_50_) with 3 nM (Figure 3e,h,i). iDeg‐9 (**102**) is the best inhibitor both in the cellular Kyn assay (IC_50_ of 5 nM) and in the in vitro IDO1 enzymatic assay (IC_50_ of 89 nM*)*. Similar results were obtained for iDeg‐7, bearing as well a carboxylic acid substituent on the *para*‐*tert*‐butylphenyl sulfonamide (Figure 2; Table 1). However, both, iDeg‐7 and iDeg‐9 were not efficient IDO1 degraders: iDeg‐7 only moderately reduced the IDO1 protein level (Figure 3f,h,i) and iDeg‐9 barely affected the IDO1 level. (Figure 3g–i).

Global proteome analysis was performed to assess the selectivity of IDO1 degradation by iDeg‐6. After 4 h treatment of SKOV‐3 cells with iDeg‐6, reduced levels were detected only for IDO1 among 4607 identified proteins, demonstrating high specificity for IDO1 degradation (Figure 3j). Concurrently, several proteins mostly related to mitochondrial function were upregulated, e.g., COX7A2, COX7C, NDUFA9 (Figure 3j and Table S2). Nicotinamide adenine dinucleotide (NAD^+^) is an enzyme cofactor and an electron transporter with crucial role for ATP synthesis during mitochondrial respiration. NAD^+^ is solely biosynthesized de novo through the kynurenine pathway. Therefore, IDO1 inhibition or decrease in IDO1 protein levels inhibit de novo NAD^+^ biosynthesis and may decrease the NAD^+^/NADH ratio.^[^ ^53^ ^]^ KEGG enrichment analysis also revealed modulation of oxidative phosphorylation (OXPHOS) by iDeg‐6 (Figures 3j and S6b), which is in agreement with observation made in human embryonic stem cells (hESCs).^[^ ^54^ ^]^

### iDeg Binding Induces Structural Changes in IDO1

To gain deeper insight into the binding mode of iDegs to IDO1 that leads to enzyme inhibition and degradation, we determined crystal structures of IDO1 in complex with iDeg‐3 (PDB ID: 9S1U), iDeg‐6 (PDB ID: 9S1V), iDeg‐7 (PDB ID: 9S1W), and iDeg‐9 (PDB ID: 9S1X). We selected iDegs with varying efficiencies in enzyme inhibition and degradation, and the obtained high resolution co‐crystal structures (resolution range: 1.85 Å – 2.10 Å) provided a structural basis for understanding their different activities (Figures 3i, and S7a; Table S3). The active site of IDO1 comprises five distinct pockets (A, B, C, D and the heme binding pocket).^[^ ^55^ ^]^ all compounds occupied pockets A, B, D and the heme binding pocket in the same binding pose as iDeg‐1 and iDeg‐2 (Figure S7b).^[^ ^13^ ^]^ Notably, all iDegs adopted a distinctive U‐shaped binding conformation allowing interactions with the entrance of the active site. The structure of the bicyclic terpene moiety and the quaternary carbon atom embedded in the fragment fusion conferred structural rigidity to the iDegs.

**Figure 4:**
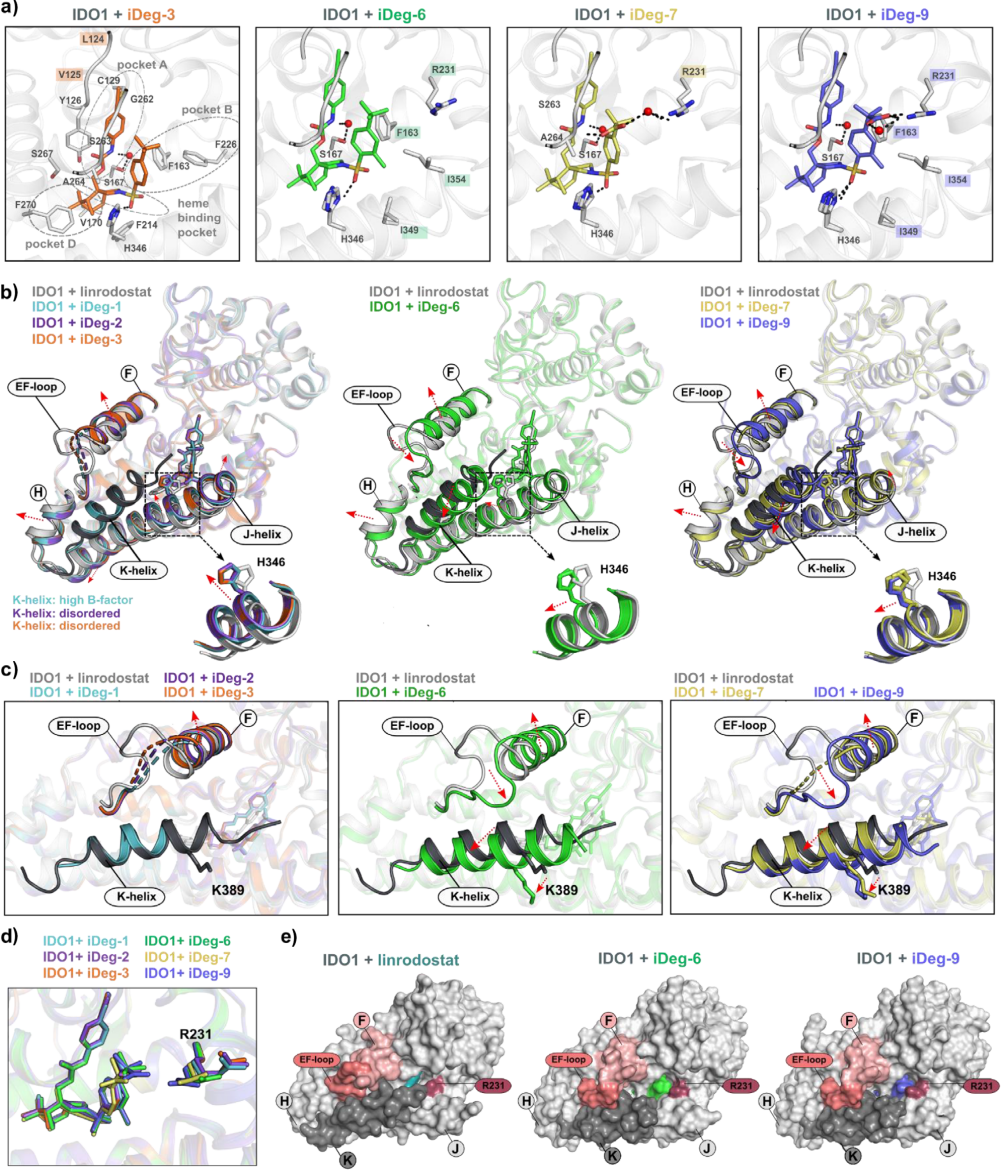
iDeg binding to IDO1 induces structural changes in the C‐terminal J‐helix, K‐helix and the EF‐loop. a) Cartoon representation of the iDeg binding site. Hydrogen bonds are shown as black dotted lines and water molecules as red spheres. All residues involved in iDeg‐3 binding are indicated (hydrogen bonds and hydrophobic contacts within ≤ 4 Å). For iDeg‐6, iDeg‐7, and iDeg‐9 only residues involved in hydrogen bonding and hydrophobic interactions within 4.0 Å that differ from those in iDeg‐3 are shown. b) Overlay of IDO1‐iDeg structures with IDO1‐linrodostat (PDB ID: 6DPR, chain B) and zoom in view of His346. Helices and loop undergoing reorientation upon iDeg‐binding are highlighted. Structural perturbations are indicated with red dotted arrows. The K‐helix of the IDO1‐linrodostat structure is highlighted in dark grey. c) As in b, zoomed‐in view of the EF‐loop, F‐helix and K‐helix, including Lys389. d) Position of Arg231 in the different IDO1‐iDeg structures. e) Surface representation of IDO1‐linrodostat, IDO1‐iDeg‐6 and IDO1‐iDeg‐9 structures illustrating the position of Arg231 in the entrance of the catalytic cleft and the EF loop shift in the iDeg‐6 and iDeg‐9 structures. The K‐helix is highlighted in dark gray, the EF‐loop in dark pink, the F‐helix in pink, and Arg231 in magenta. The surface of linrodostat is colored in teal, iDeg‐6 in green and iDeg‐9 in light neon blue.

iDegs‐3 to ‐9 bound in the highly hydrophobic active site of IDO1 through extensive hydrophobic interactions. The ethynyl group on the phenylcarbamate formed hydrophobic contacts within the distal region of the A‐pocket, specifically with the main chain including Leu124 and Val125 which contributed to the enhanced activity of iDeg‐3 compared to iDeg‐1 (Figures 4a and S7c). Tight binding in this region is critical for inhibition and degradation, as most compounds featuring an ethynyl group at this position exhibited enhanced activity in the cellular Kyn assay compared to corresponding compounds with other substituents. Another important hydrophobic interaction within the active site was mediated by the methyl group in the *meta* position of the *tert*‐butyl phenylsulfonyl group in iDeg‐6 and iDeg‐9. This additional methyl group induced a torsional adjustment of the *tert*‐butyl‐phenyl group facilitating interactions with Ile349, Ile354 and a robust π–π edge‐to‐face (T‐shaped) interaction with Phe163 (Figures 4a and S7c), thereby strengthening the binding of iDeg‐6 and iDeg‐9 to the B‐pocket.

**Figure 5:**
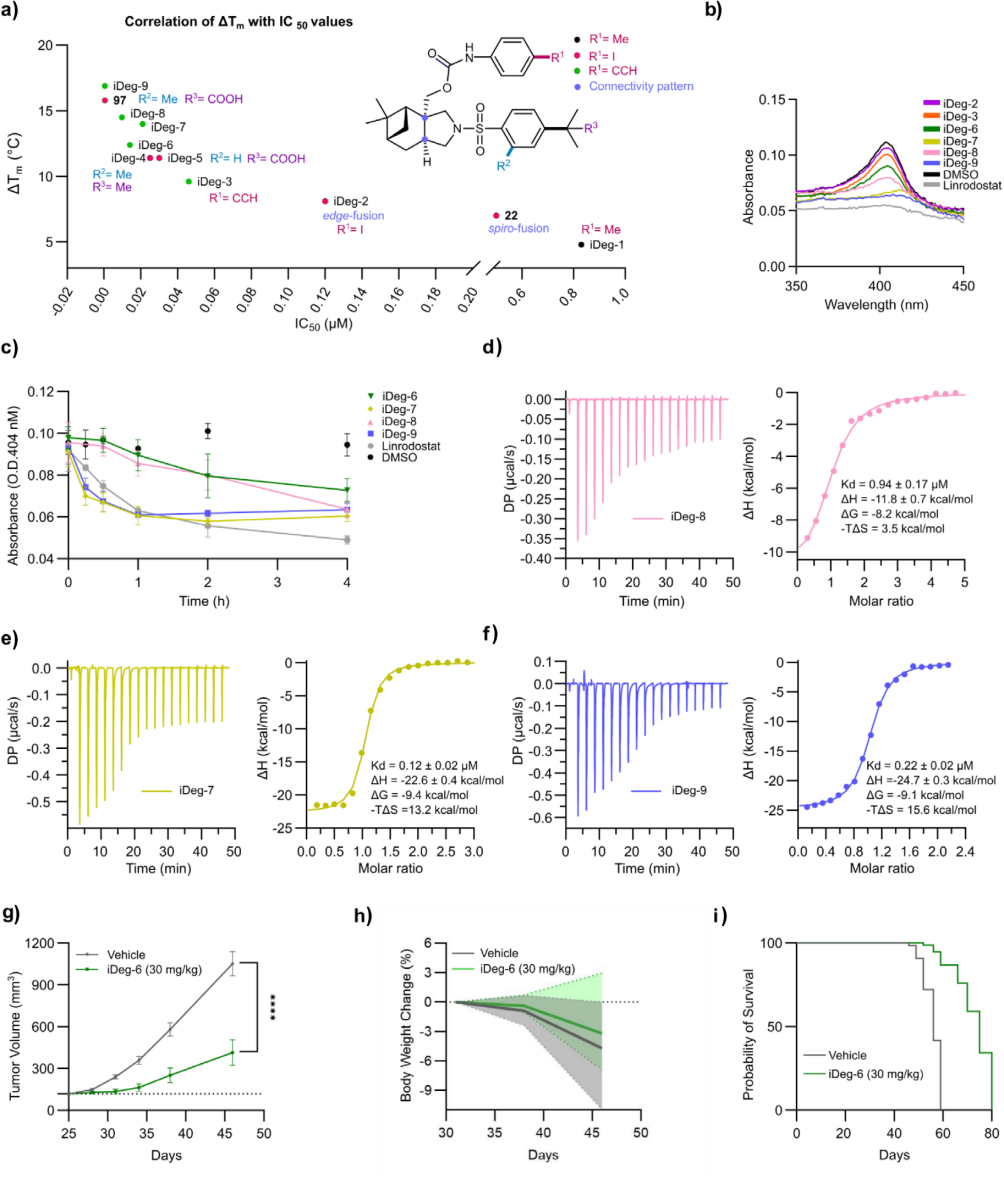
Biophysical evaluation of iDeg‐IDO1 binding and in vivo activity of iDeg‐6. a) Correlation of melting temperature changes for IDO1 with IC_50_ values of cellular Kyn assay, Pearson r and *p* values were computed by two‐tailed test and adjusted for multiple comparisons using correlation matrix procedure (*r *= 0.8. *p *< 0.005). IDO1 and compounds were pre‐incubated for 3 h at 37 °C prior to the nanoDSF measurement. Data are mean values ± SD, n = 3. b) Detection of heme‐bound IDO1 by means of UV/Vis spectroscopy in presence of iDegs (50 µM), DMSO or linrodostat (50 µM). Incubation at 37 °C for 3 h. Representative data for *n* = 3. c) Time‐course of heme displacement from IDO1 detected by the absorbance at the *Soret* peak (404 nm) as a function of time. Data are mean values ± SD, *n* = 2. d)–f) Isothermal titration calorimetry (ITC) measurement for evaluating the binding affinity of iDegs to IDO1 at 25 °C. All curves were fit using a one‐site model with *N* = 1 and defined IDO1 concentration. Left plots show baseline corrected data, while right plots show integrated binding isotherms versus iDegs/IDO1 ratio. IDO1 protein (125 µM) was titrated into the stirred sample cell containing iDegs (25 µM, except for iDeg‐8 at 50 µM). Representative data for *n* = 3, except for iDeg‐9 (*n* = 2). g)–i) In vivo activity of of iDeg‐6. NSG immunodeficient mice were injected with SKOV‐3 cells. Mice were treated with 18 intraperitoneal injections of iDeg‐6 (30 mg kg^−1^) or vehicle. The pharmacological treatment was twice daily. Tumor volume was determined weekly by digital caliper. g) SKOV‐3 tumor growth curves in iDeg‐6‐treated mice (*n* = 12, green lines) or vehicle‐treated mice (*n* = 13, gray lines). Statistical analysis was performed by two‐way ANOVA: ****p < 0.001. h) Body weight changes during treatment window (mean values ± range). i) Survival data are shown by Kaplan‐Meier survival analysis. Mantel–Haenszel statistical analysis: *p* < 0.0001).

In addition to extensive hydrophobic interactions, all iDegs bound to the active site of IDO1 via hydrogen bonds (Figures 4a and S7c). A critical hydrogen bond was formed between the sulfonyl group and His346, which coordinates the heme iron at the proximal site in the holo‐IDO1 structure.^[^ ^55^ ^]^ This interaction is crucial for both inhibition and degradation, as evidenced by the loss of activity in the Kyn assay when the sulfonyl group was replaced with a methylene (**9**) (Scheme 2 and Figure S3b). This was further confirmed by the T‐ planar amide (**16**) and sulfinamides (**18** and **19)** with asymmetric tetrahedral geometry, where a defined configuration of the sulfur atom controls the orientation of the oxygen atom (Scheme 2 and Figure S3b). Compound (**18**) has the optimal orientation for H‐bonding and exhibited a five‐fold increase in potency in the Kyn assay compared to compound (**19**). A second water‐mediated hydrogen bond was observed between the urethane NH of all iDegs and Ser167. This interaction appears essential for both binding and degradation, as replacement of the urethane group with carbonate (**13**) or ester (**15**) rendered the compounds inactive (Scheme 2a and Figure S3b). A third critical hydrogen bond was formed between the carboxylic acid group of iDeg‐7 and iDeg‐9 with Arg231. In iDeg‐7, this interaction was water‐mediated, while in iDeg‐9, molecular rotation facilitated the formation of an additional direct hydrogen bond with Arg231. This interaction, not previously observed for apo‐IDO1 inhibitors, contributed to the enhanced binding affinity of iDeg‐7 and iDeg‐9, as confirmed by the subsequent biophysical evaluation (Figure 5).

iDeg‐1 and iDeg‐2 induced a striking conformational rearrangement in IDO1, including high flexibility of the C‐terminal K‐helix (residues 381–399) and structural changes in the F‐, H‐, and J‐helices.^[^ ^13^ ^]^ Comparative analysis of the resolved IDO1‐iDeg structures with the structure of IDO1 bound to the well studied heme‐competitive inhibitor linrodostat^[^ ^48^ ^]^ and all previously reported IDO1 structures revealed that all analyzed iDegs induced similarly pronounced conformational changes (Figures 4b and S8a,b). These findings suggest that iDegs uniformly stabilize conformations that diverge from all previously characterized IDO1 structures. Notably, different iDegs induced distinct conformational alterations in the J‐helix, EF‐loop and the C‐terminal K‐helix (Figures 4b‐c and S8c,d).

The J‐helix, which harbors His346, deviated considerably in all IDO1‐iDegs structures compared to previously reported IDO1 structures. The position of the J‐helix correlated with the rotation and displacement of His346 to facilitate iDeg binding (Figures 4b and S8c). In the iDeg‐2 and iDeg‐3‐ structures, His346 induced an upward displacement of the adjacent J‐helix residues (∼2 Å). In contrast, this upward shift was absent in the IDO1‐iDeg‐6 structure, where His346 shifted toward the N‐terminus of the J‐helix, likely driven by enhanced hydrophobic interactions of the methyl group within the B‐pocket. A similar shift toward the N‐terminus of the J‐helix is observed in the iDeg‐7‐ and iDeg‐9‐complex structures.

For the C‐terminal K‐helix, which harbors the ubiquitinated Lys389, a closer comparison of IDO1 bound to iDeg‐1, iDeg‐2, iDeg‐3 and iDeg‐6 revealed significant differences in its conformation (Figures 4c and S8d). In the presence of iDeg‐1, the least potent degrader compared to iDeg‐2–6, the electron density map enabled modeling of a C‐terminal K‐helix in its standard position albeit shortened and with high B‐factors. In contrast, for the more effective degraders iDeg‐2 and iDeg‐3 no electron density corresponding to the K‐helix was detected, suggesting a disordered or highly mobile state (Figure S7d). The most efficient degrader among these four iDegs, iDeg‐6, induced a unique displacement of the K‐helix and a concurrent shift of the EF‐loop to a conformation, not previously observed in IDO1‐structures (Figures 4c and S8d). Notably, this new EF‐loop conformation would clash sterically with the canonical K‐helix position. In the IDO1–iDeg‐6 structure, the EF‐loop is accommodated at the site of the displaced K‐helix. These findings suggest a correlation between degradation efficiency and K‐helix displacement: the disordered K‐helix in the IDO1‐iDeg‐2 and IDO1‐iDeg‐3 structures represents a conformation favoring degradation and the shifted K‐helix and EF‐loop conformation observed in IDO1‐iDeg‐6 corresponds to a state even more susceptible for degradation. Although iDeg‐7 and iDeg‐9 exhibited potent inhibitory activity, their degradation efficiency was moderate or relatively weak, respectively. In both structures, the K‐helix displayed well‐defined electron density (Figures 4b‐c and S7d). In the IDO1‐iDeg‐7 structure, the EF‐loop remained disordered, and the K‐helix adopted an intermediate conformation between that observed in the IDO1‐iDeg‐6 complex and previously reported IDO1 structures. In the IDO1‐iDeg‐9 structure, the overall structural arrangement, including the EF‐loop and K‐helix positioning, closely resembled that of IDO1‐iDeg‐6 complex. Despite these structural similarities, iDeg‐9 exhibited weaker degradation efficiency than iDeg‐6, suggesting that different structural properties may account for this disparity. The primary difference is the additional carboxylic acid moiety in iDeg‐9, which alters the electrostatic environment at the entrance of the catalytic cleft and forms a hydrogen bond with Arg231 (Figure 4a). This interaction induces a conformational change positioning Arg231 closer to the iDeg molecule and partially occluding the entrance of the catalytic site (Figure 4d,e).

Altogether, the structural analysis revealed that the degradation efficiency of iDegs is linked to a remodeling of the J‐helix and displacement of the K‐helix. iDeg‐6 induced a unique EF‐loop conformation and K‐helix displacement, representing the most favorable state for degradation, while the additional carboxylic acid group in iDeg‐7 and iDeg‐9 altered the electrostatic environment at the entrance of the catalytic cleft.

### Biophysical Characterization of IDO1‐iDeg Interactions

To shed light on the properties responsible for the different potency, the binding of selected iDegs to IDO1 was analyzed in thermal shift assays (TSA) using nano differential scanning fluorimetry (nanoDSF). A significant shift in the melting temperature (Δ*T*_m_) was observed for IDO1 following the addition of the iDegs indicating binding of the compounds to the protein (Figure S9a‐d). The extent of thermal stabilization significantly correlated with the inhibition potency in the cellular Kyn assay (*P *= 0.0035, *r* = 0.8, Figure 5a) but not with the degradation potency for the tested compounds. For instance, iDeg‐9 displayed the best inhibition potency and the highest Δ*T*_m_ value but its degradation efficiency was relatively low (Figures 3i and S9d,e). IDO1 exists in two functional states: a heme‐bound form (holo‐IDO1) and a heme‐free form (apo‐IDO1). Binding of heme to IDO1 leads to a characteristic absorbance peak (Soret band) at 404 nm. UV/Vis spectroscopy demonstrated that iDegs were capable of displacing heme from holo‐IDO1. Notably, iDeg‐2 (**14**) and compound (**22**) displaced heme to a similar extent and showed identical binding in the TSA assay (Figures S10a and 5a). However, *spiro*‐fused compound (**22**) did not induce IDO1 degradation (Figure 3a,b). This observation indicates that compound (**22**) functions as an apo‐IDO1 inhibitor only. Further UV/Vis spectroscopic analysis of selected iDegs revealed most efficient heme displacement for iDeg‐7 and iDeg‐9 compared to other iDegs (Figures 5b and S10a‐b). We further analyzed the off‐rate of the heme cofactor in its ferric state in the presence of iDeg‐6 and ‐8 (inhibitors and degraders) compared to iDeg‐7 (inhibitor and moderate degrader) and iDeg‐9 (inhibitor) (Figures 5c and S10c). Heme displacement was faster in the presence of iDeg‐7 and iDeg‐9 compared to iDeg‐6 and ‐8. This property explains the superior IC_50_ value for iDeg‐9 both in the cellular Kyn assay and the in vitro IDO1 assay that is dependent on efficient heme displacement.

Dissociation constants were determined for iDeg‐6–9 using isothermal titration calorimetry (ITC) (Figure 5d–f). Both iDeg‐7 (*K*_D_ = 122 nM) and iDeg‐9 (*K*_D_ = 215 nM) demonstrated higher binding affinity than iDeg‐8 (*K_D_* = 0.94 µM, Figure 5d–f) and iDeg‐6 (*K*_D _= 1.46 µM).^[^ ^13^ ^]^ Furthermore, all three compounds bound to IDO1 in an enthalpy‐driven manner. Notably, iDeg‐8 showed a slight decrease in the enthalpy‐driven contribution, accompanied by an increase in entropy‐driven effects. This suggests that the interaction between iDeg‐8 and IDO1 might involve enhanced hydrophobic interactions.

### In Vivo Activity

The limited clinical efficacy of IDO1 inhibitors is likely due in part to its non‐enzymatic functions that are not targeted by catalytic inhibition.^[^ ^32^, ^33^, ^34^, ^35^, ^36^, ^37^, ^38^ ^]^ Since iDeg‐6 specifically inhibits IDO1 and induces IDO1 degradation in the SKOV‐3 cell line without affecting its transcription (Figure S5a), iDeg‐6 was selected as a promising candidate for in vivo investigation. Pharmacokinetic exposure and tolerability of iDeg‐6 was initially demonstrated in C57Bl/6 mice (see the Supporting Information). iDeg‐6 was well tolerated up to 30 mg kg^−1^ IP with no clinical signs. SKOV‐3 cells were subsequently engrafted into NSG immunodeficient mice to establish a SKOV‐3 tumor‐bearing model (Figure S11a). Mice were treated with iDeg‐6 at a dose of 30 mg kg^−1^, administered twice daily for 18 days. This treatment resulted in a significant inhibition of tumor growth, with a highly significant difference observed between the treatment and control groups (*p* < 0.001) (Figure 5g). Importantly, there was no significantly negative impact on body weight in either group, and the treatment group even exhibited a slight recovery in body weight (Figure 5h). This finding suggests that iDeg‐6 treatment and selective degradation of IDO1 are well tolerated in mice, with no apparent compound‐induced toxicity. In addition, the treatment significantly improved the overall survival time of the mice compared to the control group, as indicated by an increased median survival probability (Figure 5i).

### Discussion and Conclusion

Natural products are a rich source of small molecules with privileged structures for exploration of biologically relevant chemical space. However, natural evolution is inherently slow and limited by the constraint of existing biosynthetic pathways. In molecular discovery programs, the pseudo‐natural product (PNP) approach offers structurally diverse compound collections by recombining NP fragments into arrangements absent in existing NP structures, thereby enabling the identification of novel bioactivities.

We combined bicyclic monoterpenes with alkaloid‐derived pyrrolidine fragments with distinct fusions not observed in nature. Using C–H functionalization and [3 + 2] cycloaddition methodologies, we constructed structurally diverse bicyclic monoterpene‐pyrrolidine scaffolds, and subsequent functionalization enriched this monoterpene PNP collection in terms of both structural and bioactivity diversity. Different arrangements of scaffolds and features in functionalization led to the discovery of IDO1 inhibitors and of compounds with dual mechanism of action acting as IDO1 inhibitors and degraders. Specifically, *edge*‐fusion of myrtanol with pyrrolidine led to the discovery of IDO1 degraders with nanomolar activity, while *spiro*‐fusion of β‐pinene with pyrrolidine resulted in the identification of novel apo‐IDO1 inhibitors.

Of note, the precise adjustments of hydrophobic *ortho‐* and *para*‐substituents on the phenyl ring of the aryl sulfonamide embedded in the structure of the iDegs (e.g., iDeg‐6 and iDeg‐8) effectively enhanced both degradation and inhibition. Incorporation of a carboxylic acid group (e.g., iDeg‐7 and iDeg‐9) significantly increased inhibition potency in both cellular the Kyn assay and an in vitro enzymatic assay while decreasing the degradation efficiency (Figure S9f). Biophysical evaluation verified that introducing carboxylic acid moiety promote heme displacement thereby enhance inhibition potency.

Co‐crystal structure analysis revealed that all tested iDegs induced significant conformational changes in the C‐terminal K‐helix of the IDO1 protein. Notably, the most effective degrader, iDeg‐6, stabilized a unique IDO1 conformation, in which the K‐helix and the EF‐loop adopted an unprecedented position. This rearrangement likely enhances ubiquitination by the E3 ligase KLHDC3.^[^ ^13^ ^]^ The new position of the K‐helix and Lys389, to which ubiquitin is attached to induce degradation, likely represents a conformation that is efficiently ubiquitinated by the CRL2^KLHDC3^ E3 ligase. Furthermore, this new conformation of the K‐helix in all iDeg structures also exposes a hydrophobic surface of the protein that may facilitate novel protein interactions.

The quaternary carbon at the center of the iDegs and the bicyclic structure of these compounds impart conformational rigidity, which may be important for the observed structural rearrangements. Such conformationally rigid ligands may restrict the conformational flexibility of proteins upon binding IDO1, such that residues 261–265 adapt and fold around the bound ligands.^[^ ^55^ ^]^ The *edge*‐fusion of myrtanol with the pyrrolidine and the sulfonyl group define a novel promising scaffold that could be used for the design of biomolecules capable of binding within protein pockets. Furthermore, the myrtanol‐pyrrolidine structure has a steric volume that can promote conformational changes of the protein for optimal binding.

In addition, given the critical role of IDO1 in promoting tumor immune escape through tryptophan catabolism and immunosuppressive signaling, the dual targeting of IDO1 through inhibition and degradation represents a promising therapeutic strategy. Our in vivo result demonstrated that iDeg‐6 significantly suppressed tumor growth in SKOV‐3 tumor‐bearing mice and resulted in prolonged survival without inducing compound related toxicity.

In summary, a novel class of PNPs was designed and synthesized by combining bicyclic monoterpenes with structures derived from pyrrolidine alkaloids. These compounds potently reduced Kyn levels in cells by binding to the apo‐form of IDO1 and inducing conformational changes that favor IDO1 ubiquitination and degradation. SAR analysis and detailed biological evaluation revealed strong correlation between the key structural features of the iDegs and IDO1 inhibition and degradation. iDegs are a unique class of IDO1 inhibitors and their dual mode of action effectively suppresses both, IDO1 enzymatic activity and its non‐enzymatic signaling functions. Thereby, novel opportunities may be opened up to address the challenges previously faced by IDO1 inhibitors in clinical investigations.

## Supporting Information

The Supporting Information is available free of charge at https://onlinelibrary.wiley.com/doi/full/10.1002/anie.202518753. Supplementary crystallographic data for **18** has been published in the Cambridge Crystallographic Data Centre with publication numbers 2472577, and can be accessed free of charge via www.ccdc.com.ac.uk/data_request/cif. Proteomics raw data are available via ProteomeXchange (ID: PXD066412); The co‐crystal structures have been submitted to the Protein Data Bank (PDB) under the entries, 9S1U (iDeg‐3), 9S1V(iDeg‐6), 9S1W (iDeg‐7), and 9S1X (iDeg‐9).

## Conflict of Interests

The authors declare no conflict of interest.

## Supporting information



Supporting Information

## Data Availability

The data that support the findings of this study are available from the corresponding author upon reasonable request.
