# Supplementary material for: Design, Synthesis, and Structural Evolution of Pseudo‐Natural Product IDO1 Inhibitors and Degraders
Source: Angew Chem Int Ed Engl. 2025 Nov 27;65(3):e18753. doi: 10.1002/anie.202518753 (PMC12811658; doi:10.1002/anie.202518753)
Supplement: Supplementary file 1 — Supporting Information [file ANIE-65-e18753-s001.docx]

**Design, Synthesis and Structural Evolution of Pseudo-Natural Product IDO1 Inhibitors and Degraders**

Xiu-Fen Cheng^1^, Belén Lucas^1^, Philipp Lampe^3^, Stefano Ugel^4^, Suyuan Chen^1^, Anke Unger^5^, Matthias Bischoff^3^, Soheila Rezaei Adariani^1^, Kesava Reddy Naredla^1^, Kamal Kumar^1^, Annika Schmidt^2^, Carsten Strohmann^2^, Petra Janning^1^, Raphael Gasper^1^, María Lucas^6^, Malte Gersch^2,7^, Sonja Sievers^3^, Vincenzo Bronte^4^, Slava Ziegler^1^, Herbert Waldmann^1,2*^

^1^ Max-Planck-Institut für Molekulare Physiologie, Abteilung Chemische Biologie, Otto-Hahn-Straße 11, 44227 Dortmund, Germany.

^2^ Technische Universität Dortmund, Fakultät Chemie und Chemische Biologie, Otto-Hahn-Straße 6, 44221 Dortmund, Germany.

^3^ Compound Management and Screening Center Otto-Hahn-Straße 15, 44227 Dortmund, Germany.

^4^ Immunology Section, Department of Medicine, University and Hospital Trust (AOUI) of Verona, P.le L.A. Scuro, 10, 37134 Verona, Italy.

^5^ Lead Discovery Center GmbH (LDC), Otto-Hahn- Straße 15, 44227 Dortmund, Germany.

^6^ Instituto de Biomedicina y Biotecnología de Cantabria IBBTEC, Universidad de Cantabria-CSIC, C/ Albert Einstein 22, PCTCAN, 39011 Santander, Spain.

^7^ Chemical Genomics Center, Max-Planck-Institut für Molekulare Physiologie, Otto-Hahn- Straße 15, 44227 Dortmund, Germany.

* Corresponding author: herbert.waldmann@mpi-dortmund.mpg.de

**Supplementary** **Information**

**Table of Contents**

[Supplementary Figures 4](#_Toc213274113)

[Fig. S1: The construction of various bicyclic monoterpene-pyrrolidine scaffolds 4](#_Toc213274114)

[Fig. S2: The functionalization of different monoterpene–pyrrolidine scaffolds 5](#_Toc213274115)

[Fig. S3: Influence of structurally diverse monoterpene–pyrrolidine PNPs on cellular kynurenine levels. 6](#_Toc213274116)

[Fig. S4: *In vitro* assessment of IDO1 activity modulation by selected compounds*.* 7](#_Toc213274117)

[Fig. S5: Detection of IDO1 abundance by immunostaining 8](#_Toc213274118)

[Fig. S6: Influence of iDeg-6 treatment in SKOV-3 cells 9](#_Toc213274119)

[Fig. S7: Crystal structures of IDO1 and iDegs. 10](#_Toc213274120)

[Fig. S8: iDeg binding induces distinct structural changes 11](#_Toc213274121)

[Fig. S9: Influence of iDegs on IDO1 melting temperature and SAR analysis. 12](#_Toc213274122)

[Fig. S10: iDegs promotes heme displacement from IDO1. 13](#_Toc213274123)

[Fig. S11: iDeg-6 inhibits tumor growth in mice bearing SKOV-3 xenograft. 14](#_Toc213274124)

[Fig. S12: Uncropped immunoblots. Related to Figure 3b, 3d, and supplementary table S1. 16](#_Toc213274125)

[Fig. S13: Uncropped immunoblots. Related to Figure 3e-g. 16](#_Toc213274126)

[Supplementary Tables 17](#_Toc213274127)

[Table S1: IDO1 degradation induced by iDegs at 10 nM or 100 nM after 24 h incubation. 17](#_Toc213274128)

[Table S2: Changes in protein levels after treatment with iDeg-6 18](#_Toc213274129)

[Table S3: Crystallographic data collection and refinement statistics 19](#_Toc213274130)

[Methods 20](#_Toc213274131)

[Chemistry 28](#_Toc213274132)

[General procedure A: C-H functionalization with 2-isocyano-2-methylpropane 30](#_Toc213274133)

[General procedure B: [3+2] cycloaddition for Terpene-Pyrrolidine Scaffold 33](#_Toc213274134)

[General procedure C: Benzyl group cleavage 38](#_Toc213274135)

[General procedure D: Sulfonamide formation 39](#_Toc213274136)

[General procedure E: Carbamate formation 61](#_Toc213274137)

[General procedure F: Ethynyl installation 107](#_Toc213274138)

[X-Ray Structure Analysis of 18 128](#_Toc213274139)

[Fig. S14: X-ray crystal structure of 18. 129](#_Toc213274140)

[Table S4: Crystal data and structure refinement for 18. 130](#_Toc213274141)

[Pharmacokinetic and Tolerability analysis 131](#_Toc213274142)

[Representative NOESY Correlations for The Determination of Diastereomers 132](#_Toc213274143)

[References 146](#_Toc213274144)

[Publication Licenses 147](#_Toc213274145)

[NMR Spectra 150](#_Toc213274146)

# **Supplementary Figures**

**Fig. S1**: The construction of various bicyclic monoterpene-pyrrolidine scaffolds.

a,C-H activation was used for the construction of key scaffolds. b, [3+2] cycloaddition was used for the construction of key scaffolds, *^a^* **4e** and **4f** was derived from 1i-j based on the published method^[1]^.

**Fig. S2**: The functionalization of different monoterpene–pyrrolidine scaffolds.

a)NaBH_4_, MeOH, 0 °C to rt, 1 h. 85-95%; b) Pd/C, H_2_, MeOH, rt, 16 h. 85-95%; c) 4-*t*-BuPhSO_2_Cl (1.0 eq.), NEt_3_, DCM/THF, 0 °C to rt, 2 h; d) 4-IPhNCO, DBU, THF, rt, 18 h. 50-92%.


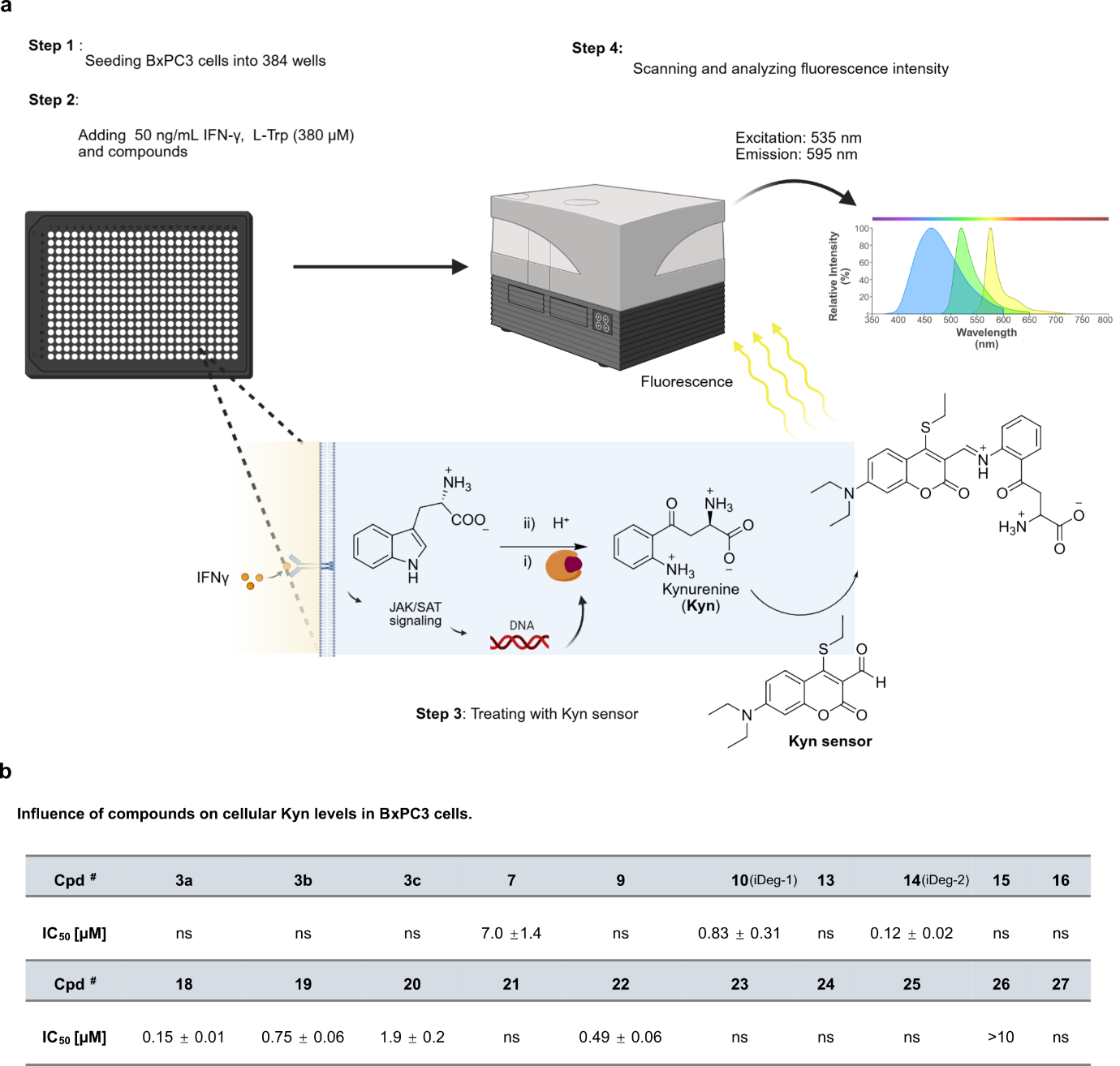


## **Fig. S3**: Influence of structurally diverse monoterpene–pyrrolidine PNPs on cellular kynurenine levels.

a, Workflow of the cellular Kyn assay. Cells were incubated with IFN-γ, L-Trp and the compounds for 48 h prior to the detection of Kyn levels. b, Influence of compounds on cellular Kyn levels in BxPC3 cells. Data are mean values ± SD. ns: not significant: > 75% residual Kyn levels observed at 10 µM; > 10 µM: Kyn levels of 50-75% observed at 10 µM. Created with BioRender.com.


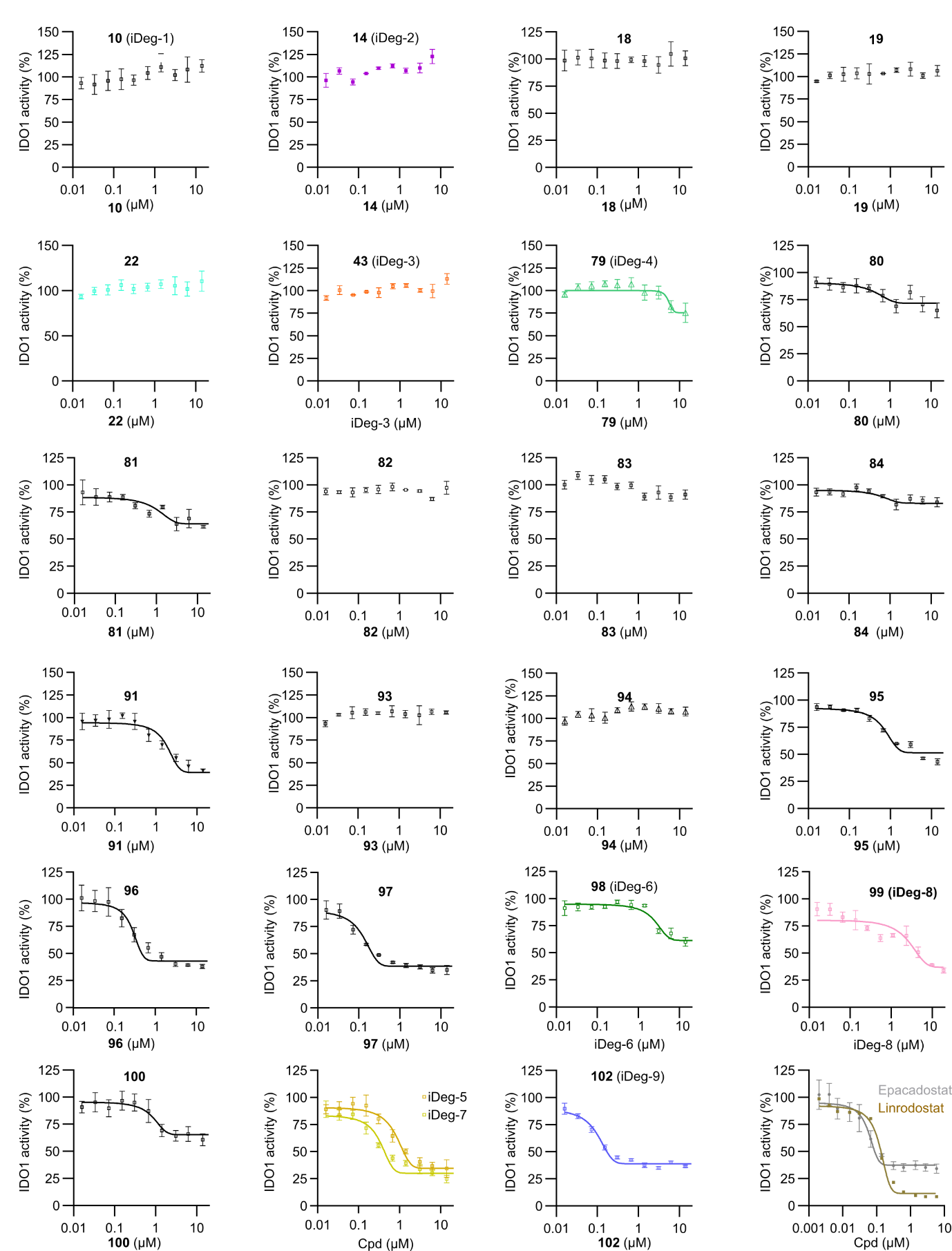


## **Fig. S4**: *In vitro* assessment of IDO1 activity modulation by selected compounds*.*

Recombinant human IDO1 protein was pre-incubated with the compounds at 37 °C for 45 min prior to detection of Kyn levels using a Kyn sensor. Data are mean values ± SD with three replicates except for **79-81**, **91-92**, **96-102,** Linrodostat, and Epacadostat (n = 4).


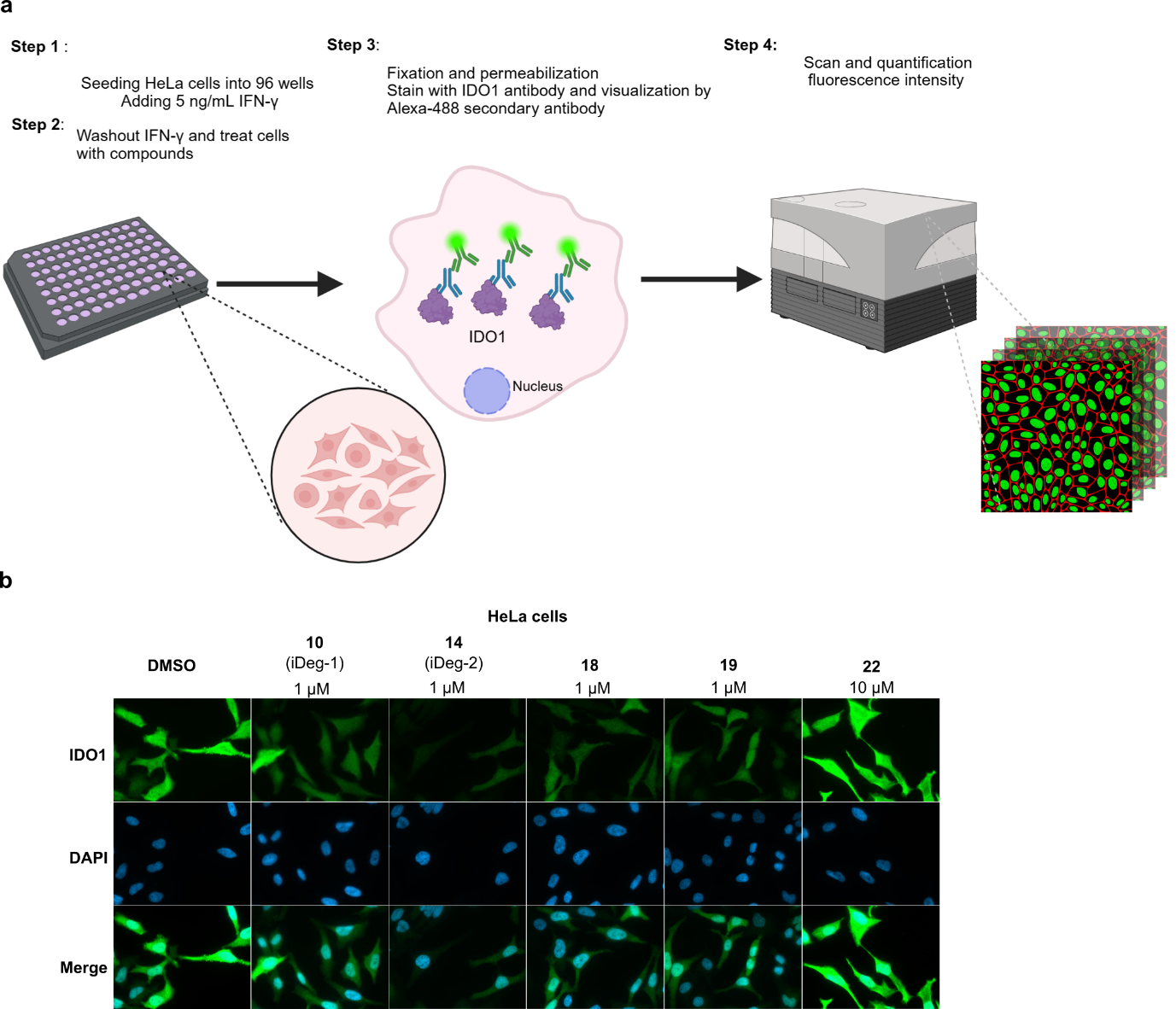


**Fig. S5**: Detection of IDO1 abundance by immunostaining.

a, Schematic representation of the used workflow, created with BioRender.com. b, Representative detection of IDO1 protein (green) and DAPI (blue) to visualize the nuclei in HeLa cells upon treatments with different iDegs, representative images of n = 3.


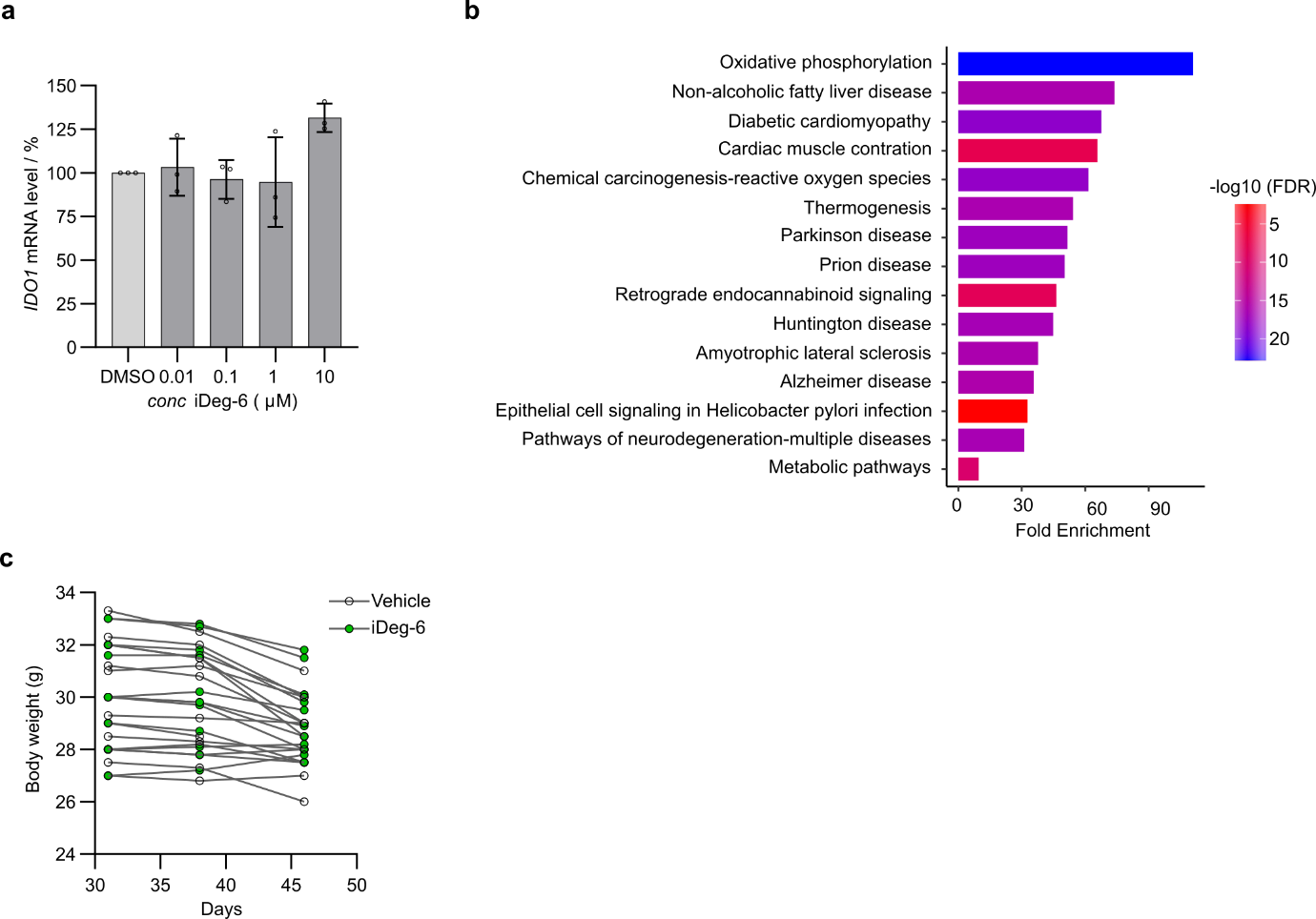


**Fig. S6**: Influence of iDeg-6 treatment in SKOV-3 cells.

a, IDO1 mRNA levels in SKOV-3 cells. SKOV-3 cells were treated with iDeg-6 for 24 h prior to RNA isolation and RT-qPCR. Data are mean values ± SD, n = 3 biological replicates. b, Influence of iDeg-6 on the global proteome after treatment of SKOV-3 cells with 10 µM iDeg-6 for 4 h. KEGG enrichment of upregulated genes, genes enriched by ShinyGO 0.8^[2]^ and ranked by FDR and fold change.


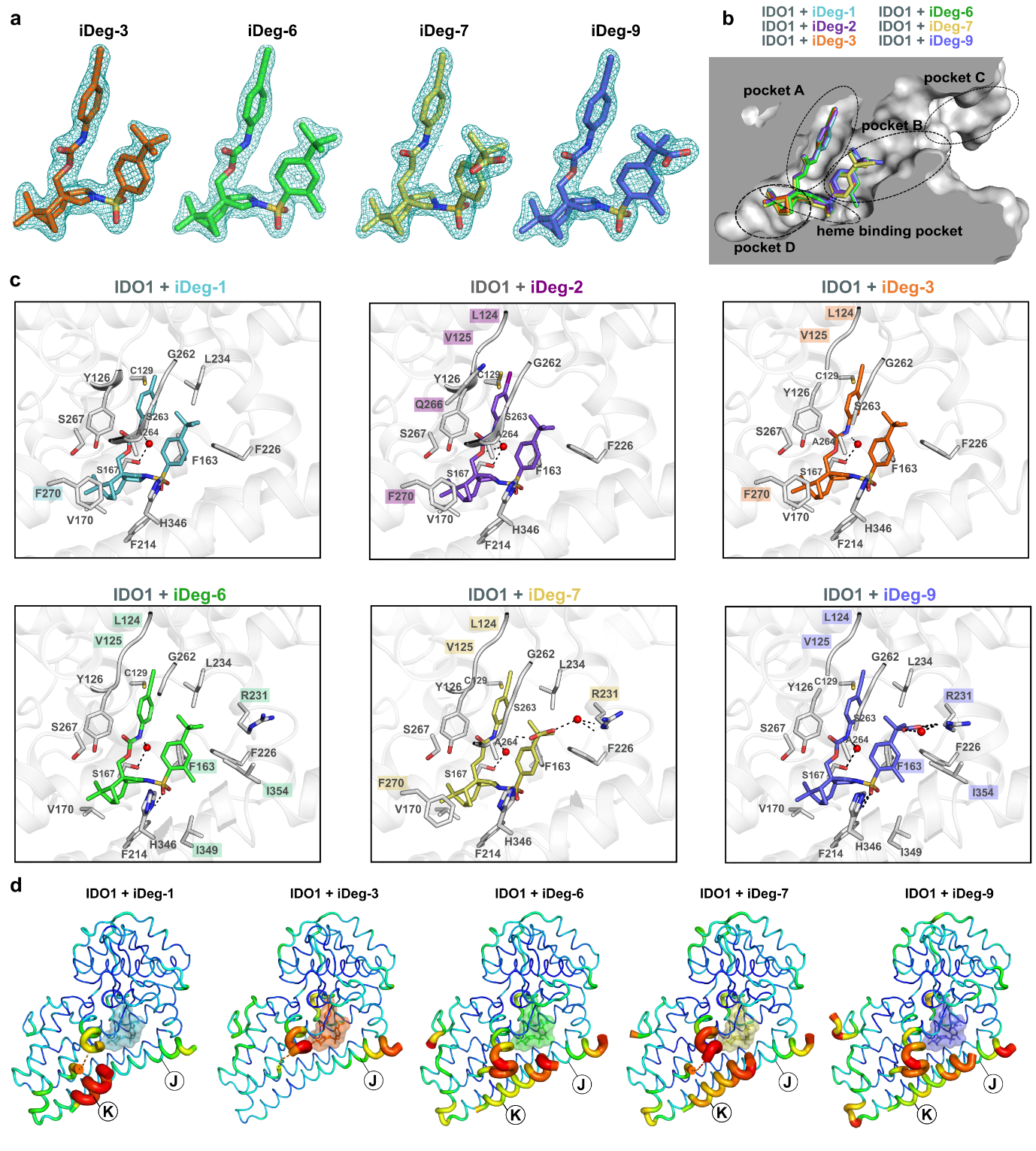


**Fig. S7**: Crystal structures of IDO1 and iDegs.

a, Electron density map (2Fo-Fc) contoured at 1.5  showing the refined iDegs structures in stick representation.b, Cross-sectional surface model representation illustrating the binding pose of iDegs within the active site of IDO1. Pockets A, B, C and D, and the heme binding site are indicated. c, Cartoon representation of the iDegs binding site. Residues involved in hydrogen bonding and hydrophobic interactions between iDegs and IDO1-protein within 4.0 Å are shown. Hydrogen bonds are indicated as black dashed lines and water molecules as red spheres. d, Relative changes in B-factor along the backbone of IDO1 in complex with iDeg-1, iDeg-3, iDeg-6, iDeg-7 and iDeg-9. Putty diagrams were generated in PyMOL. Blue colors with small diameter tubes indicate lower B-factors and less mobility, whereas orange to red colors with large diameter tubes indicate regions of higher B-factors and increased mobility within the proteins. Positions of J and K helices are indicated.


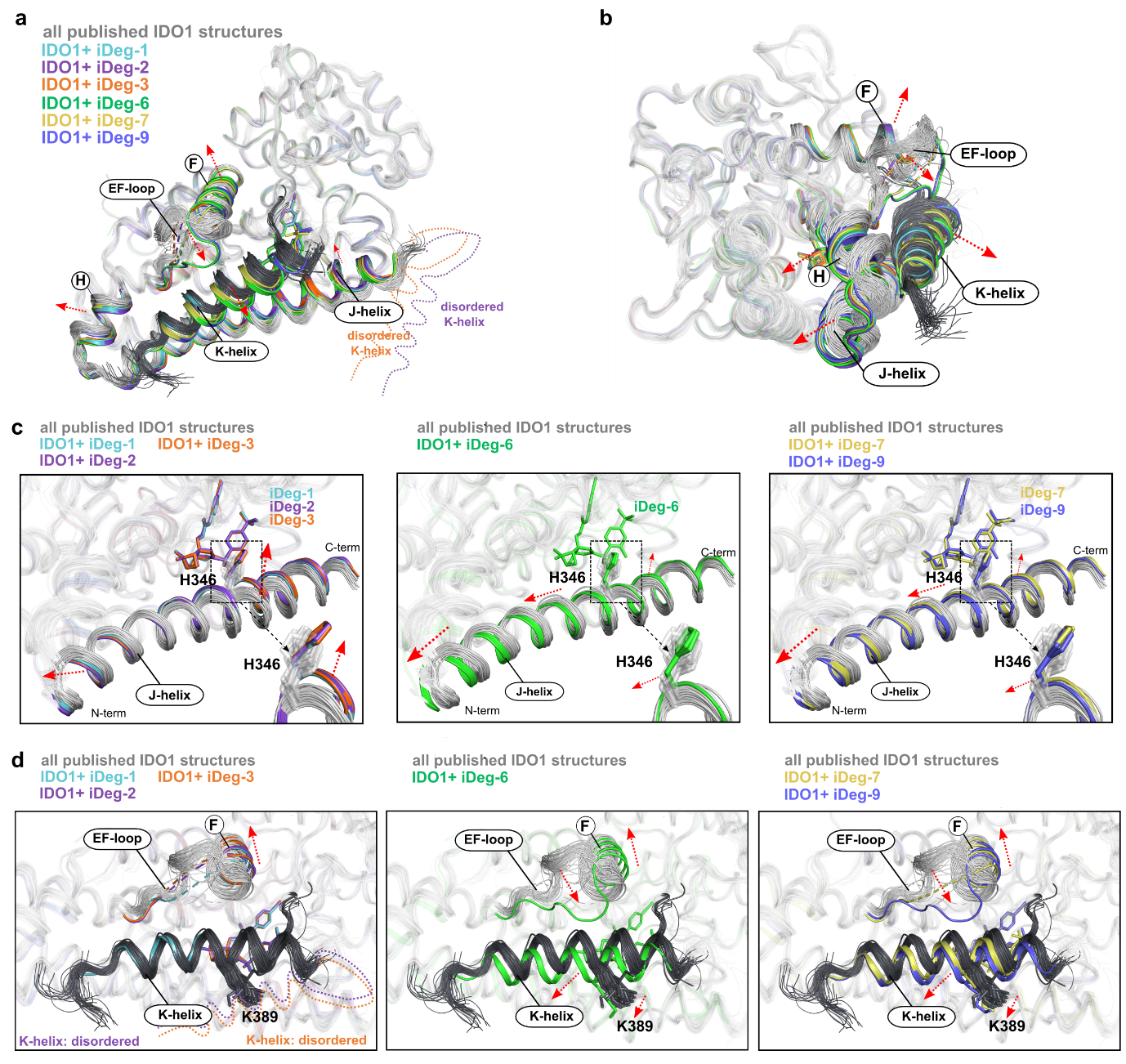


**Fig. S8**: iDeg binding induces distinct structural changes in the C-terminal J-helix, K-helix and EF-loop.

a-b*,* Overlay of the IDO1-iDegs structures with all published IDO1 structures (shown as thin cartoon) (excluding seven entries with high coordinate uncertainty). PDB IDs included: 7b1o, 7ah5, 7ah6, 7ah4, 6kof, 8i7l, 6o3i, 8fur, 7a62, 6mq6A, 6mq6B, 7p0n, 7p0r, 7m7d, 7nge, 6f0a, 6r63, 7e0t, 5wn8, 5whr, 6pu7, 5wmv, 5wmu, 5wmx, 5wmw, 6kps, 6x5y, 6wjy, 6ubp, 7e0p, 6pz1, 7e0u, 7e0o, 7e0s, 6v52, 6wpe, 7m63, 6kw7, 2d0u, 2d0t, 6e41, 7e0q, 4pk6, 5ek3, 6dpqA, 6dpqB, 6e43, 6e46, 4u74, 6cxv, 6azw, 5ek2, 5ek4, 5etw, 6e45, 6e44, 6cxu, 5xe1, 6e40, 6e35, 6dprA, 6dprB, 4pk5, 4u72, 6azv, 6azu, 6e42, 8abx, 7zv3, 7rrd, 7z2l, 7rrb, 7rrc, 7yxt and 8u5i. The absence of electron density for the K-helix in the iDeg-2 and iDeg-3 structures is represented by a dashed helix. The J-K loop is disordered in all structures except 7A62. Structural perturbations are indicated with red dotted arrows. The helices that are reoriented after iDegs-binding are indicated. Helix B is reorientated to a lesser extent. c, Same as in (a-b), showing the structures separately for better visualization. Zoomed-in view of J-helix and residue His346. The translation of the J-helix is indicated by red dotted arrows. d, Same as in (a-b), zoomed-in view of the F-helix, EF-loop and K-helix, including Lys389.


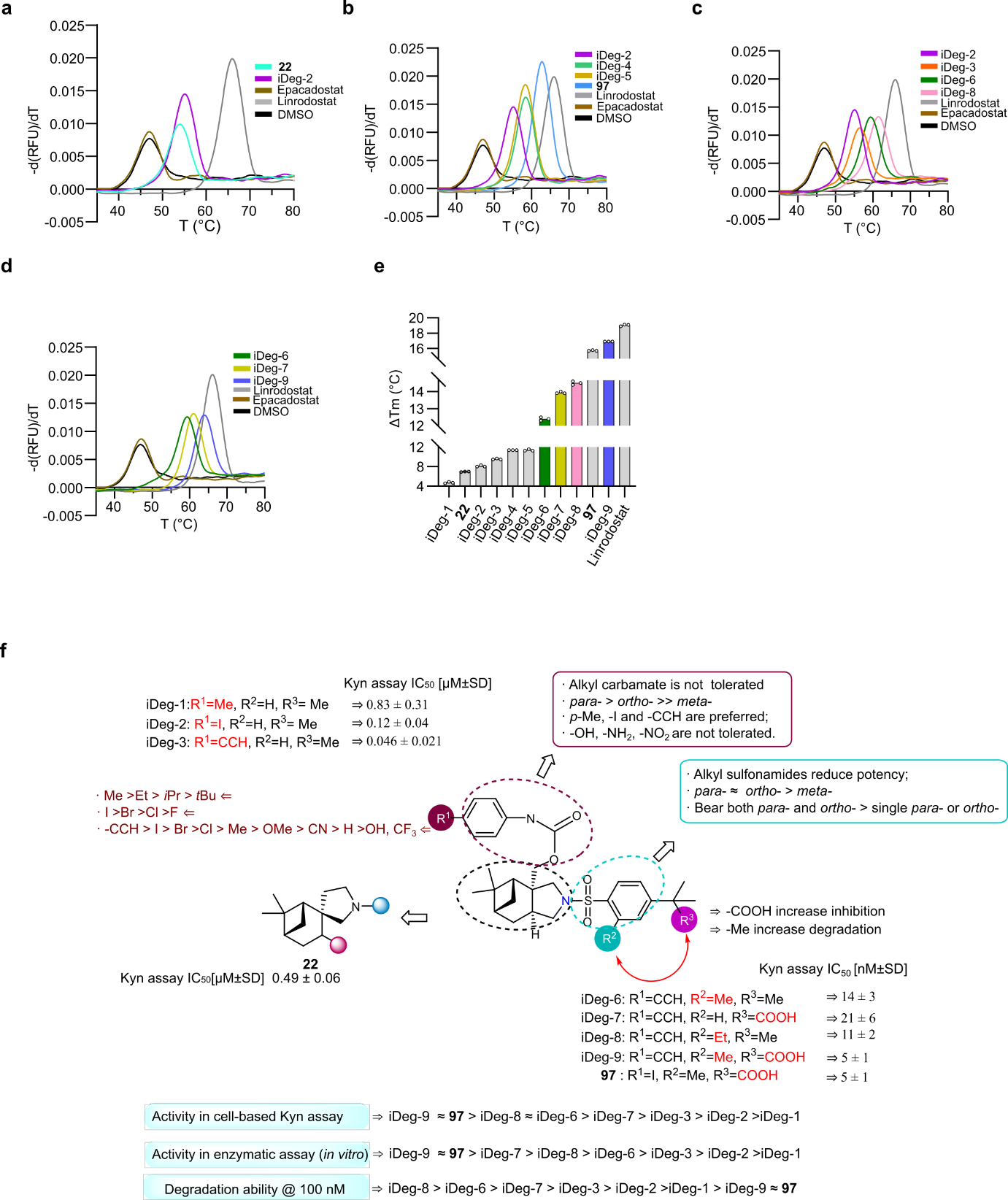


**Fig. S9**: Influence of iDegs on IDO1 melting temperature and SAR analysis.

a-d, Melting curves of IDO1 protein in presence of 50 µM iDegs, DMSO, or the apo-IDO1 inhibitor linrodostat (50 µM), measured by nano DSF. IDO1 and the compounds were incubated for 3 h at 37 °C prior to the measurement. Representative data for n = 3 biological replicates. e, Shift in melting temperature (ΔT_m_) for IDO1 from a-d. f, Summary of the SAR analysis.


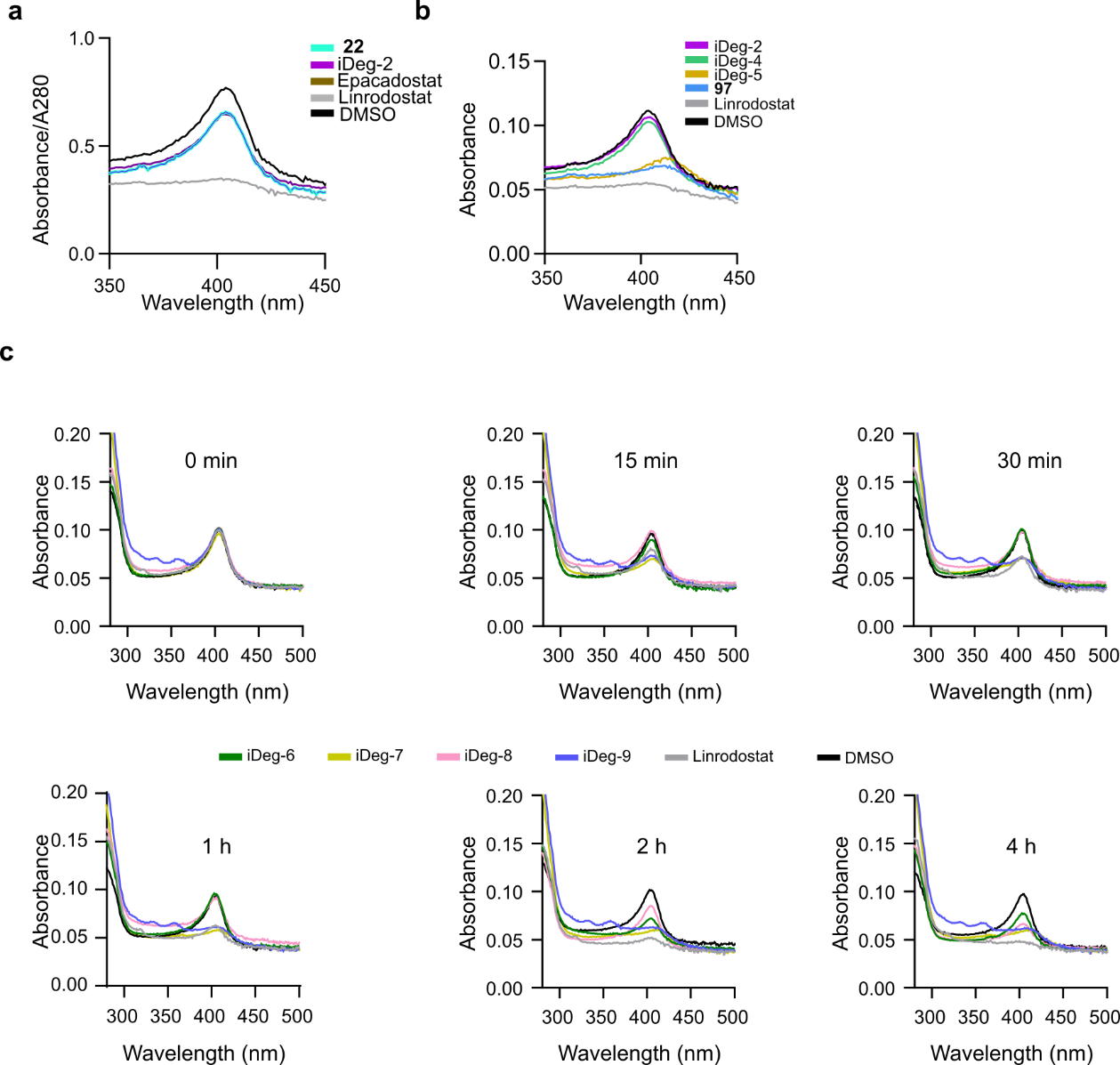


**Fig. S10**: iDegs promotes heme displacement from IDO1.

a-b, Detection of heme-bound IDO1 by UV/Vis spectroscopy in presence of iDegs (50 µM), DMSO or linrodostat (50 µM) after incubation at 37 °C for 3 h. Representative data of n=3 biological replicates. c, IDO1 was incubated with compounds (50 µM) or DMSO (control). UV/VIS spectra were acquired at five time points to monitor heme loss over time. Data are representative of two replicates.


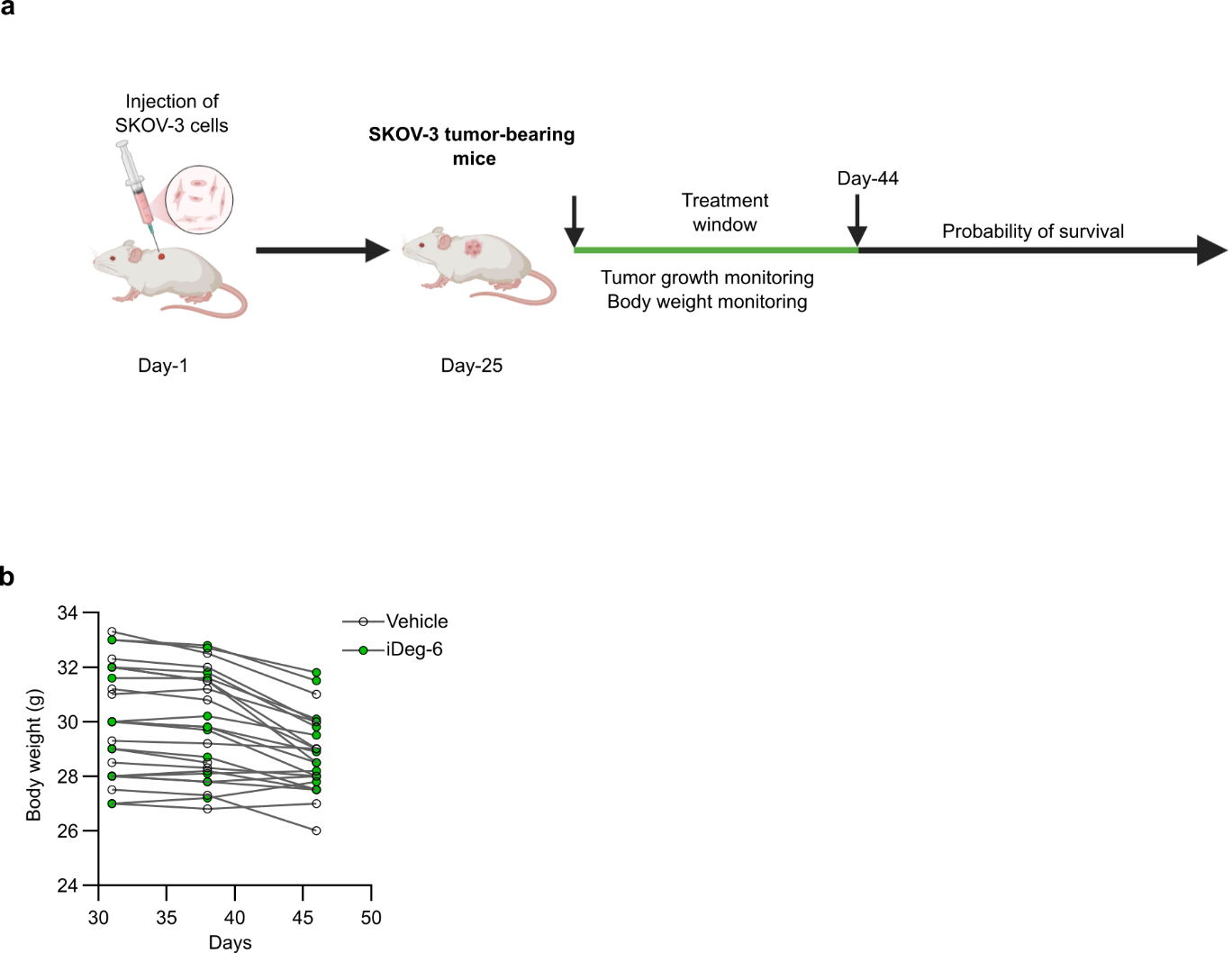


## **Fig. S11**: iDeg-6 inhibits tumor growth in mice bearing SKOV-3 xenograft.

a, Schematic representation of the experimental timeline. Created with BioRender.com. b, Effect of iDeg-6 (n = 13, green) or vehicle (n = 12, gray) treatment on body weight of SKOV-3 tumor bearing mice.


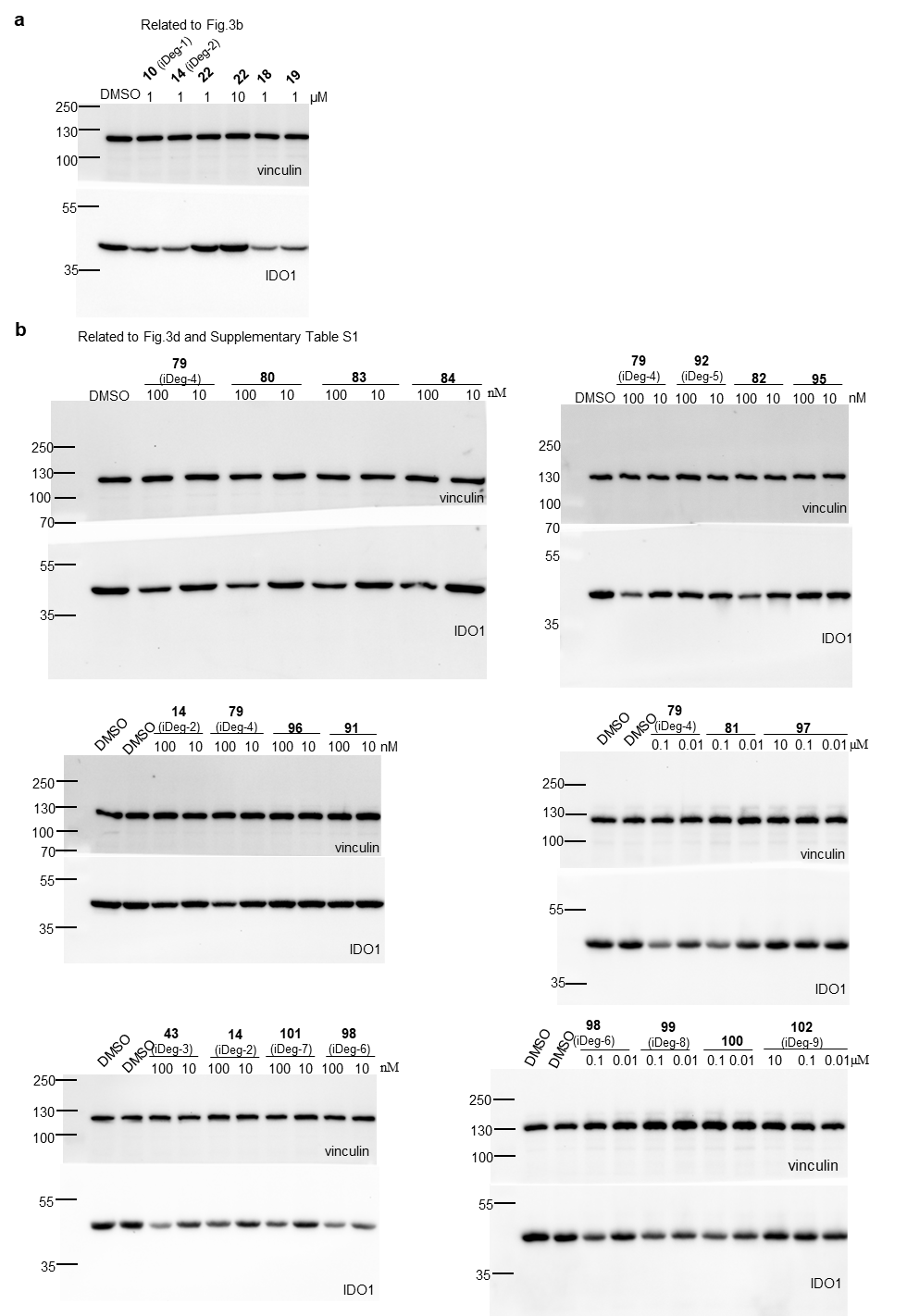


# **Fig. S12**: Uncropped immunoblots.Related to Figure 3b, 3d, and supplementary table S1.


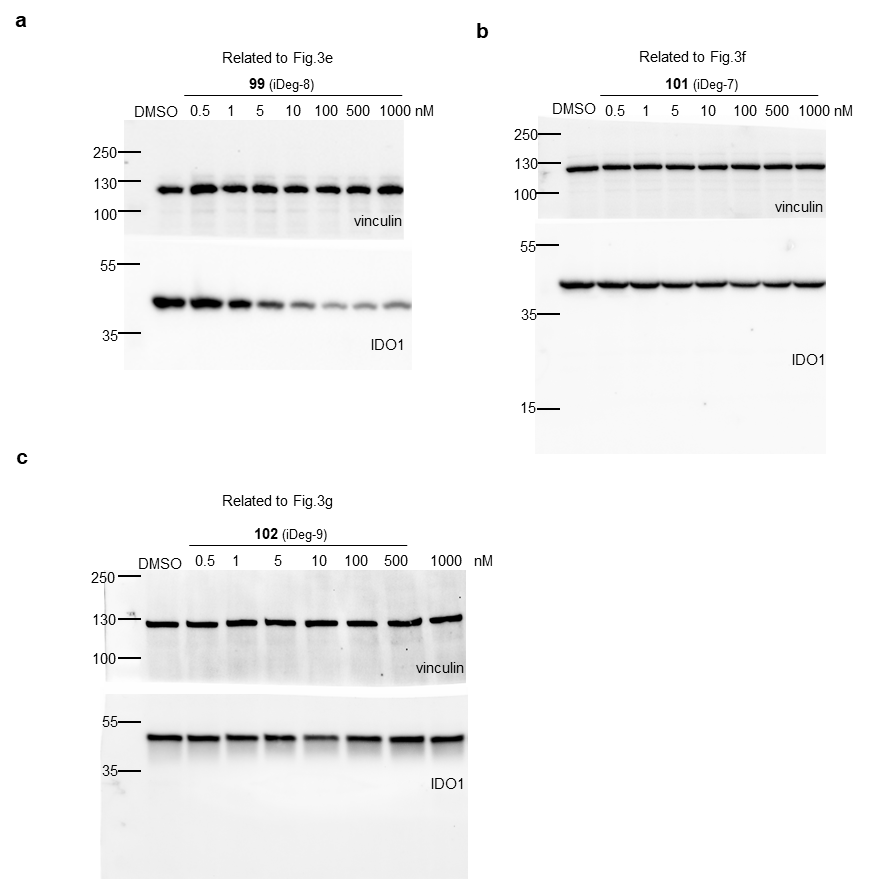


## **Fig. S13**: Uncropped immunoblots. Related to Figure 3e-g.

# **Supplementary Tables**

## **Table S1**: IDO1 degradation induced by iDegs at 10 nM or 100 nM after 24 h incubation.

| Entry | Cpd # | Max. degradation (%) (n)^a^ | | Entry | Cpd # | Max. degradation (%) (n)^a^ | |
| --- | --- | --- | --- | --- | --- | --- | --- |
|  |  | 10 nM | 100 nM |  |  | 10 nM | 100 nM |
| 1 | **14**  (iDeg-2) | 20 ± 9  (3) | 37 ± 14  (3) | 10 | **92**  (iDeg-5) | 23 ± 22  (5) | 46 ± 21  (5) |
| 2 | **43**  (iDeg-3) | 29 ± 16 (5) | 62 ± 10  (5) | 11 | **95** | Inactive  (5) | Inactive  (5) |
| 3 | **79**  (iDeg-4) | 31 ± 6  (3) | 65 ± 10  (14) | 12 | **96** | 15 ± 2  (3) | 20 ± 12  (3) |
| 4 | **80** | 25 ± 10 (3) | 56 ± 4  (3) | 13 | **97** | 11 ± 24  (3) | 18 ± 19  (3) |
| 5 | **81** | 31 ± 18 (3) | 55 ± 4  (3) | 14 | **98**  (iDeg-6) | 61 ± 12  (5) | 74 ± 13  (5) |
| 6 | **82** | 14 ± 15 (5) | 59 ± 16  (5) | 15 | **99**  (iDeg-8) | 66 ± 4  (3) | 75 ± 7  (3) |
| 7 | **83** | 24 ± 15 (3) | 56 ± 4  (3) | 16 | **100** | 61 ± 2  (3) | 71 ± 11  (3) |
| 8 | **84** | inactive | 55 ± 5  (3) | 17 | **101**  (iDeg-7) | 36 ± 10  (5) | 54 ± 8  (5) |
| 9 | **91** | 27 ± 5  (3) | 36 ± 2  (3) | 18 | **102**  (iDeg-9) | 20 ± 9  (9) | 21 ± 20  (9) |

*^a^* Data are mean values ± SD of n biological replicates (indicated in parenthesis). Inactive: no degradation (≥85% residual protein level) observed at a concentration of 10 nM or 100 nM after 24 h incubation.

**Table S2**: Changes in protein levels after treatment with iDeg-6.

SKOV-3 cells were treated with 10 µM iDeg-6 or DMSO for 4 h, followed by LC-MS/MS analysis of global protein abundance.

| **-Log(*P*-value)** | **Difference** | **Protein names** | **Gene names** |
| --- | --- | --- | --- |
| 1.14 | -0.53 | **Indoleamine 2,3-dioxygenase 1** | **IDO1** |
| 1.28 | 0.47 | V-type proton ATPase subunit d 1 | ATP6V0D1 |
| 0.97 | 0.74 | Cytochrome c oxidase subunit 7A2, mitochondrial | COX7A2 |
| 0.84 | 0.79 | Cytochrome c oxidase subunit 6C | COX6C |
| 1.56 | 0.6 | Cytochrome c oxidase subunit 7C, mitochondrial | COX7C |
| 1.09 | 0.69 | Erlin-2 | ERLIN2 |
| 1.26 | 0.44 | Cytochrome c oxidase subunit 2 | MT-CO2 |
| 1.0 | 0.52 | NADH-ubiquinone oxidoreductase chain 2 | MT-ND2 |
| 1.21 | 0.44 | NADH dehydrogenase [ubiquinone] 1 alpha subcomplex subunit 3 | NDUFA3 |
| 1.45 | 0.58 | NADH dehydrogenase [ubiquinone] 1 alpha subcomplex subunit 9, mitochondrial | NDUFA9 |
| 1.07 | 0.51 | NADH dehydrogenase [ubiquinone] 1 beta subcomplex subunit 11, mitochondrial | NDUFB11 |
| 1.00 | 0.53 | NADH dehydrogenase [ubiquinone] 1 beta subcomplex subunit 3 | NDUFB3 |
| 1.29 | 0.57 | NADH dehydrogenase [ubiquinone] 1 subunit C2 | NDUFC2 |
| 1.04 | 0.51 | NADH dehydrogenase [ubiquinone] iron-sulfur protein 5 | NDUFS5 |
| 1.14 | 0.45 | Prohibitin | PHB |
| 1.28 | 0.54 | Prohibitin-2 | PHB2 |
| 2.2 | 0.37 | V-type proton ATPase subunit a | TCIRG1 |
| 0.98 | 0.6 | Mitochondrial import receptor subunit TOM22 homolog | TOMM22 |
| 1.32 | 0.45 | Mitochondrial import receptor subunit TOM40 homolog | TOMM40 |
| 1.52 | 0.56 | Mitochondrial import receptor subunit TOM5 homolog | TOMM5 |
| 1.20 | 0.52 | Cytochrome b-c1 complex subunit 7 | UQCRB |
| 1.06 | 0.52 | Voltage-dependent anion-selective channel protein 3 | VDAC3 |

**Table S3**: Crystallographic data collection and refinement statistics.

Values in parentheses correspond to the highest-resolution shell**. ^a^** Asymmetric unit.

|  | IDO1-iDeg-3  (PDB: 9S1U) | IDO1-iDeg-6  (PDB: 9S1V) | IDO1-iDeg-7  (PDB: 9S1W) | IDO1-iDeg-9  (PDB: 9S1X) |
| --- | --- | --- | --- | --- |
|  |  |  |  |  |
| Crystal parameters |  |  |  |  |
| Space group | P 2_1_ 2_1_ 2 | P 2_1_ 2_1_ 2 | P 2_1_ 2_1_ 2 | P 2_1_ 2_1_ 2 |
| Cell dimensions |  |  |  |  |
| *a*, *b*, *c* (Å) | 104.72  110.09  36.13 | 106.79  109.91  36.15 | 106.02  110.38  36.18 | 106.75  109.65  36.01 |
|    γ (°) | 90 90 90 | 90 90 90 | 90 90 90 | 90 90 90 |
| Copies per AU^a^ | 1 | 1 | 1 | 1 |
|  |  |  |  |  |
| Data collection |  |  |  |  |
| Beamline | SLS X10SA | ESRF ID23-2 | ESRF ID30A-3 | ESRF ID30A-3 |
| Wavelength (Å) | 1.0 | 0.873 | 0.961 | 0.968 |
| Resolution range (Å) | 48.72 - 2.0 (2.07 - 2.00) | 36.15- 1.85 (1.9- 1.85) | 47.79 - 2.1 (2.15 - 2.1) | 48.77 - 2.0 (2.05 - 2.0) |
| Total reflections | 390,179 (37,651) | 496,568 (35,750) | 340,807 (23,746) | 393,655 (27,411) |
| Unique reflections | 54,475 (5,377) | 70,120 (5,095) | 47,907 (3,391) | 54,987 (3,837) |
| R_merge_ | 0.145 (2.049) | 0.221 (3.857) | 0.3905 (2.986) | 0.301 (2.546) |
| R_meas_ | 0.156 (2.213) | 0.238 (4.164) | 0.4204 (3.226) | 0.324 (2.745) |
| Mean * * ** | 8.33 (0.90) | 5.99 (0.64) | 5.84 (0.50) | 5.55 (0.67) |
| CC_1/2_ | 0.998 (0.413) | 0.997 (0.186) | 0.992 (0.187) | 0.991 (0.243) |
| Completeness (%) | 99.90 (99.89) | 99.84 (99.92) | 99.91 (99.72) | 99.43 (99.95) |
| Redundancy | 7.2 (7.0) | 7.1 (7.0) | 7.1 (7.0) | 7.2 (7.1) |
| Refinement |  |  |  |  |
| Resolution (Å) | 2.00 | 1.85 | 2.10 | 2.00 |
| No. reflections | 29,075 (2,808) | 37,269 (2,657) | 25,640 (1,807) | 29,294 (2,041) |
| *R*_work_ | 0.194 (0.339) | 0.185 (0.364) | 0.199 (0.364) | 0.183 (0.297) |
| R_free_ | 0.229 (0.370) | 0.222(0.380) | 0.237 (0.389) | 0.219 (0.305) |
| No. of non H-atoms | 3,021 | 3,276 | 3,189 | 3,236 |
| Protein | 2,877 | 3,078 | 3,045 | 3,054 |
| Ligand/ion | 73 | 76 | 68 | 69 |
| Water | 71 | 122 | 76 | 113 |
| Average B-factor (Å^2^) | 41.38 | 37.35 | 40.21 | 36.62 |
| Protein (Å^2^) | 41.22 | 37.05 | 40.41 | 36.56 |
| Ligand (Å^2^) | 48.78 | 44.87 | 35.47 | 38.37 |
| Water (Å^2^) | 40.10 | 40.42 | 36.36 | 36.99 |
| R.m.s.d. |  |  |  |  |
| Bond lengths (Å) | 0.004 | 0.008 | 0.009 | 0.008 |
| Bond angles (°) | 0.71 | 0.92 | 0.94 | 0.97 |
| Ramachandran |  |  |  |  |
| Favored (%) | 98.00 | 97.87 | 97.56 | 96.83 |
| Allowed (%) | 2.00 | 2.13 | 2.44 | 3.17 |
| Outliers (%) | 0.00 | 0.00 | 0.00 | 0.00 |
| Clashscore | 2.03 | 3.32 | 7.04 | 3.99 |
| Rotamer outliers (%) | 0.94 | 0.29 | 1.77 | 0.60 |

# **Methods**

**Materials**

BxPC3 and HeLa cell lines were obtained from DSMZ GmbH (Germany), SKOV3 cells were purchased from ATCC (USA). Fetal bovine serum (FBS), penicillin/streptomycin, and anti-IDO1 antibody (#14-9750-80) were ordered from Thermo Fisher Scientific (USA), and anti-vinculin antibody (#V9131) was sourced from Sigma-Aldrich. DMEM, McCoy’s 5A medium, RPMI-1640, sodium pyruvate, and non-essential amino acids were obtained from PAN-Biotech (Germany). Where specified, DMEM was purchased from Gibco (USA). L-tryptophan was obtained from Sigma-Aldrich. Recombinant human interferon-gamma (IFN-γ) was purchased from PeproTech. IRDye-conjugated secondary antibodies were obtained from LI-COR Biosciences. Anti-Mouse Alexa Fluor™ 488 was obtained from Invitrogen (#A-21202). Linrodostat (BMS-986205; HY-101560) was acquired from MedChemExpress. NSG mice (NOD.Cg-Prkdc^SCID^Il2rg^tm1Wjl^/SzJ) were purchased from Charles River (Calco, Italy).

**Cell Culture**

BxPC3 cells were cultured in RPMI-1640 medium (PAN-Biotech, Germany) supplemented with 10% heat-inactivated fetal bovine serum (FBS). HeLa cells were maintained in DMEM (PAN-Biotech, Germany) supplemented with 10% FBS, sodium pyruvate, and non-essential amino acids. SKOV3 cells were cultured in McCoy’s 5A medium containing 10% heat-inactivated FBS. All cell lines were maintained at 37 °C in a humidified incubator with 5% CO₂. Routine mycoplasma testing confirmed that all cell cultures were free of mycoplasma contamination.

**Automated Kyn Screening Assay**

The high-throughput kynurenine (Kyn) screening assay was conducted as described earlier^[3]^. BxPC3 cells were seeded in phenol red-free RPMI-1640 medium (PAN-Biotech, Germany) into 1536-well (Greiner Bio-One, #782086) or 384-well plates (Corning, #3770) 24 h prior to treatment with compounds, interferon-gamma (IFN-γ; 50 ng/mL, PeproTech), and L-tryptophan (L-Trp; 380 µM, Sigma-Aldrich). After 48 h of incubation, Hoechst 33342 was added for detecting cell viability. Subsequently, trichloroacetic acid (TCA; Sigma-Aldrich) was added to a final concentration of 7% and incubated for 10 minutes, followed by centrifugation at 1620 × *g* for 10 minutes. Kyn levels were quantified using the Kyn sensor^[3]^ at a final concentration of 17.5 µM in sensor buffer (50 mM H₃PO₄ and 120 mM NaCl, pH 1.0). SpectraMax Paradigm plate reader (Molecular Devices) was used to detect fluorescence intensity (excitation: 535 nm; emission: 595 nm). Data were normalized to DMSO-treated control wells. All high-throughput screening (HTS) data were analyzed using the Quattro Software Suite (Quattro Research GmbH).

**IDO1 Enzymatic Assay *in vitro***

The tested compounds were incubated with recombinant human full-length IDO1 protein (0.8 µM) in 50 mM potassium phosphate buffer (50 mM KH₂PO₄ and 50 mM K₂HPO₄, pH 6.5, 1% glycerol and 0.05% Tween-20) for 45 minutes at 37 °C. The reaction was initiated by the addition of 20 mM ascorbic acid, 20 µM methylene blue, 0.6 mM L-tryptophan (Trp), and 200 µg/mL catalase (Sigma-Aldrich, C100), followed by incubation for 60 minutes at 37 °C. Subsequently, trichloroacetic acid was added to a final concentration of 6%, and samples were further incubated for 30 minutes at 37 °C. Kynurenine (Kyn) levels were quantified by adding 0.08 mM Kyn sensor in acetic acid, and fluorescence intensity was measured at an excitation wavelength of 541 nm and emission at 595 nm using a SpectraMax Paradigm microplate reader (Molecular Devices). Data were normalized to the DMSO-treated control. Results are expressed relative to the DMSO control. Dose-response curves and IC₅₀ values were generated using GraphPad Prism version 9.0 (GraphPad Software, USA) and fitted with a four-parameter variable slope nonlinear regression model.

**Immunofluorescence**

IFN-γ-stimulated Hela cells (5 ng/mL IFN-γ) were seeded in 96-well plates and incubated for 24 h. Subsequently, IFN-γ was washed out and cells were treated with compounds for additional 24 h. Cells were then washed once with PBS and fixed with 3.7% paraformaldehyde for 10 min at room temperature. Cells were washed three times with ice-cold PBS. The fixed cells were permeabilized with 0.1% Triton-X100 for 15 min followed by blocking with 2% BSA in PBS-T (0.1%) for 1 h at room temperature. The primary antibody against IDO1 (1:1000) was diluted in 2% BSA in PBS-T (0.1%) and incubated with the cells at 4 °C overnight. Cells were then washed three times with PBS-T and once with PBS, followed by incubation with the secondary antibody, Alexa Fluor™ 488 (A-21202) for fluorescent labeling. Cell nuclei were stained using DAPI. Images were acquired at 10 x magnification using Axiovert 200M (Zeiss) microscope. Images were analyzed by SpectraMax Paradigm microplate reader (Molecular Devices) to extract IDO1 fluorescence intensity as well as the cell count. IDO1 fluorescence intensity was normalized to cell count. Data were plotted using Prism 9.0 (GraphPad, USA).

**Immunoblotting**

To analyze IDO1 protein expression, BxPC3 cells were stimulated with (IFN-γ; 50 ng/mL) and seeded into 12-well plates, followed by a 24 h incubation. Afterwards, IFN-γ was washed out, and cells were treated with test compounds for additional 24 h. Cells were then lysed in lysis buffer containing 150 mM NaCl, 50 mM Tris-HCl (pH 8.0), 1% NP-40 alternative, and protease/phosphatase inhibitors. Protein concentrations were determined using the DC Protein Assay (Bio-Rad). Equal amounts of protein were mixed with 4 × LDS sample buffer (NuPAGE^®^, Invitrogen) and separated on a 10% SDS-PAGE gel. Proteins were transferred to a polyvinylidene difluoride (PVDF) membrane using a wet-tank blotting system (Bio-Rad). Membranes were blocked with Intercept® blocking buffer (LI-COR Biosciences, #927-70001) for 1 h at room temperature. IDO1 and vinculin (loading control) were detected using primary antibodies: anti-IDO1 (1:2500; ab211017, Abcam or 14-9750-80, Thermo Fisher Scientific) and anti-vinculin (1:5000; V9131, Sigma-Aldrich), both diluted in Intercept® PBS blocking buffer. Membranes were incubated with primary antibodies overnight at 4 °C. The next day, membranes were incubated with IRDye-conjugated secondary antibodies (LI-COR Biosciences), and washing was performed using PBS-T (PBS + 0.1% Tween-20). Fluorescent signals were visualized by ChemiDoc™ MP Imaging System (Bio-Rad), and band intensities were quantified by Image Lab software (Bio-Rad).

**Reverse Transcription-Quantitative PCR**

To assess IDO1 gene expression in the SKOV3 cell line, SKOV3 cells were seeded into 24-well plates. After 24 h, the cells were treated with the indicated concentrations of compounds, followed by an additional 24 h incubation.

Total RNA was extracted using the RNeasy Mini Kit (Qiagen), according to the manufacturer’s protocol. RNA samples were reverse transcribed into complementary DNA (cDNA) using the QuantiTect Reverse Transcription Kit (Qiagen). Quantitative real-time PCR (qPCR) was subsequently performed using the QuantiFast SYBR Green PCR Kit (Bio-Rad) on a CFX96 Real-Time PCR Detection System (Bio-Rad). Gene-specific primers were used to amplify IDO1 (forward: 5’-GCCTGATCTCATAGAGTCTGGC-3’; reverse: 5’-TGCATCCCAGAACTAGACGTGC-3’) and GAPDH (forward: 5’-GTCTCCTCTGACTTCAACAGCG-3’; reverse: 5’-ACCACCCTGTTGCTGTAGCCAA-3’), which served as the internal control. Relative gene expression was calculated using the 2^−ΔΔCt^ method.^[4]^ Data were analyzed using the CFX Manager software (Bio-Rad), with IDO1 expression normalized to GAPDH.

**TMT-Based Global Proteome Profiling of SKOV3 Cells Treated with iDeg-6**

SKOV-3 cells were treated with 10 µM iDeg-6 or DMSO for 4 h. Cells were collected after trypsinization. The collected cell suspension was centrifugated and washed twice by cold PBS before lysate preparation by means of four freeze-thaw cycles in PBS containing 0.4% NP-40 alternative with protease (Roche, #5892970001) and phosphatase inhibitors (Roche, #4906845001). Protein concentration was determined by DC protein assay (BIO-RAD, #5000116) and 200 μg of protein lysates were subjected to the sample preparation and MS analysis. Protein lysates were dissolved in equal volume of 100 mM triethylammonium bicarbonate (TEAB) buffer supplemented with 7.5 µL of a 200 mM Tris(2-carboxyethyl) phosphine (TCEP) followed byan incubation step at 55 °C for 1 h. Afterwards, 7.5 µL of 375 mM iodoacetamide were added and samples were incubated for another 30 min at room temperature in the dark prior to acetone-based protein precipitation (pre-incubation at -20 °C overnight). On the next day, samples were centrifuged at 8,000 x *g* and 4 °C, the supernatant was aspired and protein pellets were dried at room temperature. Pellets were dissolved in digestion buffer (trypsin: protein ratio-1:50 (w/w)), in which trypsin was pre-dissolved in 10 mM HCl, and then diluted in 100 mM TEAB buffer to a final concentration of 0.4 µg/µL trypsin in 10 mM HCl, then the sample was incubated with shaking overnight at 37 °C. On the next day, samples were labelled with TMT10plex reagents (Thermo Scientific, WL320956) following the instruction of the TMT10plex Mass Tag Labeling Kit. Namely, 20 µL of the TMTpro Label reagent was added to each 100 µL sample (100 µg protein) followed by incubation for 2 h at room temperature while shaking. Afterwards, 5 µL of 5% hydroxylamine was added into each sample, and the samples were incubated for 15 min to quench the reaction. Equal amounts of each sample were combined in a new tube. The pooled samples were dried in a SpeedVac at 30 °C until the liquid evaporated completely.

**Mass Spectrometry (MS)**

All solvents used during MS measurements were LC-MS grade. The TMTpro labeled peptides were cleaned up and fractionated using a reversed-phase fractionation spin column (Thermo Fisher Scientific ^#^84868) following the instructions of the Pierce™ High pH Reversed-Phase Peptide Fractionation kit. Briefly, the combined labeled samples (40 µg) were fully dissolved in 300 μL of 0.1% TFA and loaded onto an activated spin column, followed by centrifugation at 3000 *g* for 2 minutes. The column was then transferred to a fresh 2.0 mL tube, washed with 300 μL of water and 300 μL of 5% acetonitrile in 0.1% triethylamine respectively, then centrifuged and the wash flow was discarded. Subsequently, eight fractions were collected by sequentially loading 300 μL of elution buffer containing increasing concentrations of acetonitrile (10.0%, 12.5%, 15.0%, 17.5%, 20.0%, 22.5%, 25.0%, and 50.0%) in 0.1% triethylamine. Each step was followed by centrifugation at 3000 g for 2 minutes to collect the eluates. All collected fractions were evaporated to dryness using a SpeedVac concentrator at 30 °C.

For nanoHPLC-MS/MS analysis, the dried samples were dissolved in 20 μl of 0.1% TFA in water prior to the injection of 10 μl onto an UltiMateTM 3000 RSLCnano system (Thermo Fisher Scientific, Germany) online coupled to a Q Exactive™ HF Hybrid Quadrupole-Orbitrap Mass Spectrometer equipped with a nanospray source (Nanospray Flex Ion Source, Thermo Fisher Scientific). For desalting, peptide samples were loaded onto a trap column (pre-column cartridge, 5 µm, 100 Å, 300 µm ID × 5 mm; Dionex, Germany) using 0.1% trifluoroacetic acid (TFA) in water as the loading solvent, with a flow rate of 30 µL/min. Desalting was carried out for 5 minutes with the flow directed to waste. Subsequently, peptides were back-flushed from the pre-column onto an analytical PepMap100 RSLC C18 nano-HPLC column (2 µm, 100 Å, 75 µm ID × 50 cm; nanoViper, Dionex, Germany). Chromatographic separation was performed using a linear gradient starting at 95% solvent A (water with 0.1% formic acid) and 5% solvent B (acetonitrile with 0.1% formic acid), increasing to 40% solvent B over 120 minutes at a flow rate of 300 nL/min. The column was subsequently washed reaching 95% solvent B and then re-equilibrated to initial conditions. Mass spectra were acquired in data-dependent acquisition (DDA) mode. For TMT10plex-labeled samples, full MS scans were recorded over an m/z range of 375–1500 with a resolution of 120,000, followed by up to 15 high energy collision dissociation (HCD) MS / MS scans of the most intense at least doubly charged ions using a resolution of 60000 and a normalized collision energy (NCE) of 35%. Raw data were processed using MaxQuant (version 2.4.14.0), with peptide identification performed via the integrated Andromeda search engine against the UniProt human reference proteome database. Trypsin was specified as the proteolytic enzyme, allowing for up to two missed cleavages. Carbamidomethylation of cysteine was set as a fixed modification, while oxidation of methionine and N-terminal acetylation were defined as variable modifications. For TMT10plex-based quantification, the "reporter ion MS2" quantification method was selected. TMT labeling was defined for lysine residues and peptide N-termini. Mass tolerances were set to 20 ppm for the first search, 4.5 ppm for the second search, and 20 ppm for MS/MS scans. The experiment included four biological replicates per condition.

**Data analysis of proteomics results**

A false discovery rate (FDR) of 1% was applied for both peptide and protein identification. Only proteins quantified with at least two peptides were considered for further analysis. Relative protein quantification was performed using the reporter ion MS2 algorithm implemented in MaxQuant. Proteins not identified by at least two razor and unique peptides in at least one biological replicate were excluded. Replicates were grouped by treatment condition (treated vs. control), and proteins not quantified in at least three replicates within either group were also removed from the dataset. Following data filtering, the report ion intensities were corrected calculation of log^2^ and normalization to median of each replicate. Statistical analysis was carried out using UMSAP version 2.3.3. Missing values were imputed as '0', and a t-test was conducted with parameters t₀ = 0.5 and s₀ = 0.5. The results were exported as Excel (.xlsx) files and visualized using UMSAP. Enrichment analysis of upregulated genes was performed using ShinyGO version 0.8. Proteomics results are supplied as Fig. 3j, Supplementary Fig. S6b and Supplementary Table S2. Proteomics raw data are available via ProteomeXchange (ID: PXD066412).

**Monitoring tumour growth in mice**

NSG female mice (NOD.Cg-PrkdcSCIDIl2rgtm1Wjl/SzJ) were purchased from Charles River (Calco, Italia) with 10 weeks age. 5x10^6^ SKOV-3 cells were inoculated subcutaneously into the left flank of mice. When tumor volume reached ~125 mm^3^, mice were separated in two groups: iDeg-6 treated (n = 12) and vehicle-treated (n = 13) mice. The pharmacological treatment was repeated 18 times by twice daily dosing (30 mg/kg). Tumor volume was determined weekly by digital caliper from 10 days after tumor challenging to the endpoint of the experiment (day 80). All mice were maintained under specific pathogen-free conditions in the animal facility of the University of Verona. Food and water were provided ad libitum. Animal experiments were performed according to national protocol number C46F4.26 approved by the Ministerial Decree Number 993/2020-PR of July 24, 2020, and protocol number C46F4.30 approved by the Ministerial Decree Number 227/2023-PR of March 14, 2023 [PI: Stefano Ugel] and European laws and regulations. All animal experiments were approved by the Animal Welfare Organization (OPBA) (https://www.univr.it/it/ateneo/organismo-preposto-al-benessere-degli-animali-opba) and conducted according to the guidelines of the Federation of European Laboratory Animal Science Association (FELASA). All animal experiments were under the Amsterdam Protocol on animal protection and welfare: mice were monitored daily and euthanized when displaying excessive discomfort.

**Cloning and Protein Purification of Recombinant IDO1**

IDO1 protein expression and purification was performed as previously described by Littlejohn et al^[5]^. Full length human IDO1 cDNA was cloned into the pGEX6p-2rbs using EcoRI and SalI restriction sitesIDO1 protein expression was induced with 200 µM IPTG and cells were incubated at 18 °C overnight. Harvested cells were collected and resuspended in lysis buffer, and the cells were lysed by sonication. The lysate was centrifuged at 13,000 x *g* for 35 min at 10 °C. The supernatant was loaded onto a GST Trap HP column to enrich GST-tagged IDO1, equilibrated in 50 mM Tris-HCl, 150 mM NaCl and 1 mM DTE, pH 7.0. The GST-tag of IDO1 was removed to obtain untagged full-length IDO1l using PreScission protease with overnight incubation. The eluted IDO1 protein was further purified using a size exclusion column (26/60 G75 HiLoad) to yield highly purified rhIDO1.

For hIDO1crystallization, a truncated version (5-400) of IDO1 was cloned into the pGEX6p-2rbs vector using the EcoRI and SalI restriction sites. Apo-IDO1 was obtained by removing heme through overnight incubation with 166 mM 2-mercaptoethanesulfonate (MESNA). Heme occupancy was determined by UV/VIS spectrophotometry.

**Differential Scanning Fluorimetry (nanoDSF)**

The test compounds or DMSO (vehicle control) were incubated with recombinant full-length human IDO1 (rhIDO1; 10 µM) in 50 mM potassium phosphate buffer (pH 6.5) for 3 h at 37 °C. Following incubation, samples were centrifuged at 5,000 × *g* for 10 minutes, then the protein-compound solutions were loaded into capillaries, and thermal unfolding was assessed using the Prometheus Panta system (NanoTemper® Technologies, Germany). Fluorescence intensities at 330 nm and 350 nm were recorded at temperature increments of 0.115 °C from 20 °C to 90 °C. Melting temperatures (T_m_) were automatically calculated using Prometheus NT.48 analysis software. The resulting thermal denaturation curves, with T_m_ defined as the temperature at the maximum derivative of the unfolding transition, were visualized and analysed using GraphPad Prism version 9.0 (GraphPad Software, USA).

**UV/VIS analysis**

The test compounds or DMSO (vehicle control) were incubated with recombinant full-length human IDO1 (rhIDO1; 10 µM) in 50 mM potassium phosphate buffer (pH 6.5) for 3 h at 37 °C. Following incubation, samples were centrifuged at 5,000 × *g* for 10 minutes. UV/Vis absorption spectra were then recorded from 250 nm to 650 nm in 1 nm increments using UV-transparent microplates (UV-STAR®, Greiner Bio-One, Austria) on a Spark® multimode microplate reader (Tecan, Austria). Absorbance values were analyzed at 404 nm (A404), and were plotted using GraphPad Prism version 9.0 (GraphPad Software, USA).

**Isothermal Titration Calorimetry**

ITC was performed using the MicroCal PEAQ-ITC system (Malvern) at 25 °C. Recombinant human full-length IDO1 (rhIDO1) was buffer-exchanged into 100 mM phosphate buffer (50 mM KH₂PO₄ and 50 mM K₂HPO₄, pH 6.57) with 1 mM TECP using a 10 K Amicon® Ultra centrifugal filter (Millipore). The buffer exchange was repeated ten times to ensure complete removal of interfering components. The final protein sample was diluted in the same phosphate buffer prior to titration.125 μM protein in 100 mM phosphate buffer was loaded into the syringe, while 25 μM compound (except for iDeg-8, 50 μM) in 100 mM phosphate buffer was loaded to the cell. All samples were adjusted to 25 °C and degassed before loading. Titrations were performed at 25 °C with one injection of 0.4 µL, followed by 18 injections of 2 µL. Experiments were performed with three biological replicates except for iDeg-9 with two biological replicates. Data were analyzed using the MicroCal PEAQ-ITC Analysis software and were plotted using GraphPad Prism 9.0 (GraphPad, USA).

**Co-crystallization of IDO1 protein with iDeg-3, iDeg-6, iDeg-7 and iDeg-9**

Apo-IDO1 protein (5-400) at 10 mg/mL (0.225 mM) was incubated with iDeg-3 (0.3 mM) (3.0% (v/v) DMSO), iDeg-6 (1.1 mM) (2.2% (v/v) DMSO), iDeg-7 (1.1 mM) (2.2% (v/v) DMSO) and iDeg-9 (1.1 mM) (2.2% (v/v) DMSO) in 25 mM Tris pH 8.0 and 100 μM TCEP at 37 °C (iDeg-3) or 42 °C (for iDeg-6, iDeg-7 and iDeg-9) for 2 h. 0.4 µL of the protein solution was mixed with 0.2 µL of reservoir solution in a sitting drop setup (iQ plates, SPT Labtech, UK) at 20 °C. Following incubation, the soluble protein fraction was separated from the precipitate by centrifugation at 20,000 × *g* for 15 minutes at 20 °C. Crystallization trials were performed using the sitting drop vapor diffusion method in 96 well iQ plates (SPT Labtech) at 20 °C. Crystallization drops were prepared by mixing 400 nl of the protein-iDeg solution with 200 nl of reservoir solution using a mosquito HTS robot (TTP Labtech). Crystals were obtained in wells with reservoir solution containing, 42% (iDeg-3), 43% (iDeg-6), 38% (iDeg-7) and 38% (iDeg-9) (v/v) PEG200 in 50 mM MES, pH 6.81 (iDeg-3), 7.0 (iDeg-6), 5.8 (iDeg-7) and 5.94 (iDeg-9) respectively. Thin bar-shaped crystals appeared within 24h and were harvested and frozen in liquid nitrogen using the corresponding reservoir solution as cryoprotectant after 5-73 days. X-ray diffraction data were collected at Swiss Light Source (SLS, Villigen, Switzerland) on beamline X10SA (iDeg-3) and at European Synchrotron Radiation Facility (ESRF Grenoble, France) on beamline ID23-2 (iDeg-6) and ID30A-3 (iDeg-7 and iDeg-9). Diffraction data sets were processed using XDS^[6]^ and scaled with XSCALE. The crystal structures were solved using Phaser^[7]^ from the Phenix suite^[8]^ with the IDO1-iDeg-2 structure (PDB ID: 9FOH) as initial model. Iterative refinement cycles were carried out using PHENIX and COOT^[9]^. Figures were generated using the PyMOL Molecular Graphics System (Version 3.13, Schrödinger, LLC). The PDB ID codes, data collection and refinement statistics are presented in Extended Data Table S3. The structures have been submitted to the Protein Data Bank (PDB) under the entries, 9S1U (iDeg-3), 9S1V(iDeg-6), 9S1W (iDeg-7), and (iDeg-9, 9S1X).

# **Chemistry**

**General Information**

Unless otherwise stated, all commercially available compounds were used without further purification. Chromatography solvents were of technical grade. Analytical thin-layer chromatography (TLC) was carried out on Merck aluminum-backed silica gel 60 F254 plates, using petroleum ether and ethyl acetate or dichloromethane and methanol as gradient eluents. Compounds were visualized under UV light. Column chromatography was performed using Merck silica gel 60 (particle size 0.040–0.063 mm). Optimized compounds were further purified via preparative HPLC using a NUCLEODUR C18 Gravity column (250 × 21 mm, 10 μm particle size).

¹H, ¹³C, and ¹⁹F NMR spectra were recorded on Bruker DRX400 (400 MHz), DRX500 (500 MHz), INOVA500 (500 MHz), and DRX700 spectrometers, using CD₂Cl₂, CDCl₃, DMSO as solvents. Chemical shifts (δ) are reported in ppm relative to the solvent peaks (CD₂Cl₂: δ = 5.32 ppm for ¹H, 53.84 ppm for ¹³C; CDCl₃: δ = 7.26 ppm for ¹H, 77.16 ppm for ¹³C; DMSO-*d*_6_: δ = 2.5 ppm for ¹H, 39.85 ppm for ¹³C). Multiplicities are noted as s (singlet), d (doublet), t (triplet), q (quartet), or m (multiplet), and coupling constants (*J*) are given in Hz.

High-resolution mass spectra (HRMS) were obtained using a LTQ Orbitrap mass spectrometer coupled to an Accela HPLC system with a Hypersyl GOLD column (50 × 1 mm, 1.9 μm particle size) and electron spray ionization (ESI).

Single-crystal X-ray diffraction data were collected on a Bruker D8 Venture four-circle diffractometer equipped with a PHOTON II CPAD detector. X-ray radiation was generated using an IµS 3.0 Mo microfocus source with HELIOS mirror optics and a single-hole collimator (Incoatec/Bruker AXS). Data collection and processing were performed using the APEX 3 Suite (v.2018.7-2), including SAINT for integration and SADABS for absorption correction (Bruker AXS GmbH).

(+)-3-Carene (#C0047), (-)-Perillaldehyde (#P086), (-)-β-Pinene (#P0441), (+)-α-Pinene (#1099) were purchased from TCI, Sabinene (W530597,) was purchased from Sigma-Aldrich and identified as having [α]^20^_D_ = -74.1° (CHCl_3_, *c* = 1.0). The terpenes were further functionalized to bicyclic monoterpenes 1a-1c according to the following reference.^[10]^ 1d(#218243)was purchased from Sigma-Aldrich without further purification. Compounds 1e, 1f, 1h, 1i were synthesized from related terpenes according to the following references.^[11-14]^ The arenesulfonyl chlorides were synthesized according to the following references,^[15-16]^ and applied into the next step immediately after simply purification by chromatography.

**The general procedure for the synthesis of 1a-1c**:

1) The terpene aldehyde (13.2 mmol) was stirred into a solution of sodium chlorite (92.4 mmol, 7 eq.), monobasic sodium phosphate (69.6 mmol, 5 eq.), and 2-methyl-2-butene (3.38 mL) in a 20:1 *tert*-butyl alcohol-water mixture (20 mL) at room temperature. After 30 min, TLC analysis indicated that no aldehyde remained. The solvents were evaporated, and the residue was dissolved in 1 M hydrochloric acid (20 mL) and extracted with ether twice. The combined extracts were washed with brine (50 mL), dried by Na_2_SO_4_, filtered through silica gel, and evaporated. The crude acid was used for next step without further purification.

2) To a solution of the crude terpene acid (6.6 mmol) in DCM (20 mL), DMF (1.32 mmol, 0.2 eq.), oxalyl dichloride (1.1 mL, 13.2 mmol, 2.0 eq.) was added into the reaction mixture in an ice-bath. The reaction mixture was stirred at room temperature for 3 h, and then concentrated in vacuo. The residue was dissolved in toluene (7.5 mL), *O*-alkyl hydroxylamine hydrochloride (7.26 mmol, 1.1 eq.), which was added to a solution of Na_2_CO_3_ (1.4 g, 13.2 mmol, 2.0 eq.) in toluene/H_2_O (30 mL, v/v = 1:1) slowly at 0˚C. The resulting mixture was stirred at room temperature overnight. The mixture was extracted with EtOAc for three times, and the combined organic phase was washed with brine, dried over anhydrous Na_2_SO_4_, filtered, and concentrated under reduced pressure. The crude residue was purified by silica gel chromatography (PE:EtOAc, 10:1, v/v) to yield the product **1a-c** as a light-yellow liquid.

**(1R,5S)-N-methoxy-6,6-dimethylbicyclo[3.1.1]hept-2-ene-2-carboxamide (1a):**

**Yield**: 85%.

**TLC**: (PE:EA=2:1, v/v): R*_f_*= 0.3, yellow liquid.

**^1^H NMR** (500 MHz, Chloroform-d) δ 6.36 (m, 1H), 3.79 (s, 3H), 2.99 (s, 1H), 2.61 (td, *J =* 5.6, 1.6 Hz, 1H), 2.47 (dt, *J =* 9.1, 5.6 Hz, 1H), 2.39 (dt, *J* = 14.4, 3.1 Hz, 2H), 2.13 (ttd, *J* = 5.6, 2.8, 1.3 Hz, 1H), 1.33 (s, 3H), 1.15 (d, *J* = 9.1 Hz, 1H), 0.82 (s, 3H).

**^13^C NMR** (126 MHz, CDCl_3_) δ 165.8, 141.1, 130.4, 64.4, 41.8, 40.5, 37.9, 32.0, 31.5, 26.0, 21.0.

**HRMS** (ESI) for C_11_H_17_NO_2_Na: calculated 218.1157 [M+Na]^+^, found: 218.1153.

**(1S,5R)-N-methoxy-6,6-dimethylbicyclo[3.1.1]hept-2-ene-2-carboxamide (1b):**

**Yield**: 82%.

**TLC**: (PE:EA=2:1, v/v): R*_f_*= 0.3, yellow liquid.

**^1^H NMR** (500 MHz, Chloroform-*d*) δ 6.37 (dt, *J* = 3.3, 1.7 Hz, 1H), 3.79 (s, 3H), 2.61 (td, *J* = 5.6, 1.6 Hz, 1H), 2.47 (dt, *J* = 9.1, 5.7 Hz, 1H), 2.39 (dt, *J* = 14.5, 3.1 Hz, 2H), 2.13 (m, 1H), 1.33 (s, 3H), 1.15 (d, *J* = 9.1 Hz, 1H), 0.82 (s, 3H).

**^13^C NMR** (126 MHz, CDCl_3_) δ 165.7, 141.1, 130.4, 64.4, 41.8, 40.5, 37.9, 32.0, 31.5, 26.0, 21.1.

**HRMS** (ESI) for C_11_H_17_NO_2_Na: calculated 218.1157 [M+Na]^+^, found: 218.1153.

**(1R,6S)-N-methoxy-7,7-dimethylbicyclo[4.1.0]hept-2-ene-3-carboxamide (1c):**

**Yield**: 85%.

**TLC**: (PE:EA=2:1, v/v): R*_f_*= 0.4, yellow liquid.

**^1^HNMR** (500 MHz, Chloroform-*d*) δ 6.82 – 6.78 (m, 1H), 3.76 (s, 3H), 2.36 – 2.25 (m, 1H), 1.93 – 1.72 (m, 3H), 1.18 (dd, *J* = 8.2, 5.4 Hz, 1H), 1.12 (s, 3H), 1.09 (td, *J* = 6.4, 3.2 Hz, 1H), 0.88 (s, 3H).

**^13^CNMR** (126 MHz, CDCl_3_) δ 167.2, 134.2, 128.5, 64.3, 29.1 28.33, 24.9, 23.4, 21.7, 17.1, 15.9.

**HRMS** (ESI) for C_11_H_17_NO_2_Na: calculated 218.1157 [M+Na]^+^, found: 218.1155.

## **General procedure A: C-H functionalization with 2-isocyano-2-methylpropane**

***N*-methoxyamide Terpene** (293 mg, 1.5 mmol, 1.0 eq.), **Pd_2_(dba)_3_** (68.7 mg, 0.0375 mmol, 0.5 mol%; ^#^328774, Sigma-Aldrich), ***t*-BuNC** (339 µL, 3 mmol, 2.0 eq; ^#^260630, Sigma-Aldrich), and **dioxane** (15 mL) were added into a 10 mL round-bottom flask equipped with a magnetic stir bar and condenser. The mixture was stirred in a pre-heated oil bath at 80 °C for 6–12 h. After completion, the reaction was cooled to room temperature, and the solvent was removed under reduced pressure. The crude residue was purified by silica gel chromatography (hexane/EtOAc = 30:1, v/v) to yield the product **2a-c** as a light-yellow liquid (61–74%).

To a 25 mL reaction vessel, **2a-c** (0.4 mmol) and Pd/C (11 mg, 0.2eq.) were dissolved into MeOH (5.0 mL). The reaction vessel was evacuated and backfilled with H_2_ (×3) and the mixture was stirred at room temperature for 24 h under 1 atm of H_2_. Upon completion, the mixture was filtered through a short celite-packed column and washed with MeOH. The solvents were removed under reduced pressure. The crude residue was purified by silica gel chromatography (hexane/EtOAc = 50:1, v/v) to yield the product **3a-c** as a light-yellow liquid **3a-c** (18-99%).

**(4R,6R)-2-(tert-butyl)-1-(methoxyimino)-5,5-dimethyl-1,2,4,5,6,7-hexahydro-3H-4,6-methanoisoindol-3-one (2a)**:

**Yield:** 61%.

**TLC**: (PE:EA=10:1): R*_f_*= 0.7, yellow liquid.

**^1^H NMR** (500 MHz, Chloroform-*d*) δ 3.91 (s, 3H), 2.86 (d, *J* = 2.9 Hz, 1H), 2.81 (d, *J* = 2.6 Hz, 1H), 2.66 (d, *J* = 5.4 Hz, 1H), 2.55 (ddd, *J* = 9.2, 6.0, 5.2 Hz, 1H), 2.25 – 2.20 (m, 1H), 1.64 (s, 9H), 1.35 (s, 3H), 1.15 (d, *J* = 9.1 Hz, 1H), 0.76 (s, 3H).

**^13^C NMR** (126 MHz, CDCl_3_) δ 168.8, 152.8, 148.0, 133.6, 63.0, 58., 41.5, 39.5, 38.0, 32.5, 31.4, 29.9, 26.0, 21.4.

**HRMS** (ESI) for C_16_H_25_N_2_O_2_: calculated 277.1911 [M+H]^+^, found: 277.1905.

[α]^20^_D_ = -60.3° (CHCl_3_, *c* = 0.25).

**(4S,6S)-2-(tert-butyl)-1-(methoxyimino)-5,5-dimethyl-1,2,4,5,6,7-hexahydro-3H-4,6-methanoisoindol-3-one (2b):**

**Yield:** 63%.

**TLC**: (PE:EA=10:1, v/v): R*_f_*= 0.7, yellow liquid.

**^1^H NMR** (600 MHz, Chloroform-*d*) δ 3.91 (s, 3H), 2.87 (dd, *J* = 19.5, 3.0 Hz, 1H), 2.79 (dd, *J* = 19.5, 2.6 Hz, 1H), 2.66 (d, *J* = 5.4 Hz, 1H), 2.55 (dt, *J* = 9.2, 5.6 Hz, 1H), 2.25 – 2.20 (m, 1H), 1.64 (s, 9H), 1.35 (s, 3H), 1.15 (d, *J* = 9.2 Hz, 1H), 0.76 (s, 3H).

**^13^C NMR** (151 MHz, CDCl_3_) δ 168.8, 152.8, 148.0, 133.6, 63.0, 58.0, 41.5, 39.5, 38., 32.5, 31.4, 29.9, 26.0, 21.4.

**HRMS** (ESI) for C_16_H_25_N_2_O_2_: calculated 277.1911 [M+H]^+^, found: 277.1907.

[α]^20^_D_ = +65.3° (CHCl_3_, *c* = 0.5).

**(5aS,6aR,Z)-2-(*tert*-butyl)-1-(methoxyimino)-6,6-dimethyl-1,4,5,5a,6,6a-hexahydrocyclopropa[e]isoindol-3(2H)-one (2c):**

**Yield:** 74%.

**TLC**: (PE:EA=10:1, v/v), R*_f_*= 0.8, yellow liquid.

**^1^H NMR** (700 MHz, Chloroform-*d*) δ 3.91 (s, 3H), 2.28 – 2.22 (m, 1H), 2.03 – 1.96 (m, 1H), 1.95 – 1.89 (m, 2H), 1.76 (ddd, *J* = 8.9, 6.0, 3.1 Hz, 1H), 1.63 (s, 9H), 1.18 (s, 3H), 1.14 (td, *J* = 7.6, 3.0 Hz, 1H), 0.83 (s, 3H).

**^13^C NMR** (176 MHz, CDCl_3_) δ 170.2, 152.4, 136.4, 135.2, 63.0, 57.8, 29.8, 28.6, 28.1, 24.5, 24.2, 18.0, 17.3, 15.8.

**HRMS** (ESI) for C_16_H_25_N_2_O_2_: calculated 277.1911 [M+H]^+^, found: 277.1906.

[α]^20^_D_ = +62.3° (CHCl_3_, *c* = 0.5).

**(4S,6R)-2-(tert-butyl)-1-(methoxyimino)-5,5-dimethyloctahydro-3H-4,6-methanoisoindol-3-one (3a)：**

**Yield:** 99%**.**

**TLC**: (PE:EA=10:1, v/v): R*_f_*= 0.8, yellow liquid.

**^1^H NMR** (500 MHz, Chloroform-*d*) δ 3.81 (s, 3H), 3.39 (td, *J* = 11.0, 6.1 Hz, 1H), 2.93 (dd, *J* = 10.8, 4.5 Hz, 1H), 2.53 (ddd, *J* = 14.4, 11.4, 4.6 Hz, 1H), 2.43 (dt, *J* = 5.7, 4.6 Hz, 1H), 2.38 – 2.32 (m, 1H), 1.96 – 1.93 (m, 1H), 1.93 – 1.89 (m, 1H), 1.62 (s, 9H), 1.19 (s, 3H), 1.17 (d, *J* = 10.0 Hz, 1H), 0.79 (s, 3H).

**^13^C NMR** (126 MHz, CDCl_3_) δ 178.8, 162.5, 62.0, 58.8, 46.9, 41.36, 40.4, 39.6, 30.3, 28.7, 28.4, 27.3, 26.5, 21.0.

**HRMS (ESI)** for C_16_H_27_N_2_O_2_: calculated 279.2067 [M+H]^+^, found 279.2067.

**(4R,6S)-2-(tert-butyl)-1-(methoxyimino)-5,5-dimethyloctahydro-3H-4,6-methanoisoindol-3-one (3b):**

**Yield:** 75%**.**

**TLC**: (PE:EA=10:1, v/v): R*_f_*= 0.8, yellow liquid.

**^1^H NMR** (500 MHz, Methylene Chloride-*d*_2_) δ 3.78 (s, 3H), 3.38 (d, *J* = 6.1 Hz, 1H), 2.91 (dd, *J* = 10.8, 4.2 Hz, 1H), 2.51 (ddd, *J* = 14.4, 11.3, 4.5 Hz, 1H), 2.39 – 2.31 (m, 2H), 1.95 – 1.89 (m, 2H), 1.60 (s, 9H), 1.19 (s, 3H), 1.16 (d, 1H), 0.77 (s, 3H).

**^13^C NMR** (126 MHz, CD_2_Cl_2_) δ 178.7, 162.9, 62.0, 58.8, 47.1, 41.8, 40.7, 39.8, 30.6, 28.8, 28.4, 27.4, 26.8, 21.2.

**HRMS (ESI)** for C_16_H_26_N_2_O_2_Na: calculated 301.1892 [M+Na]^+^, found 301.1879.

**(5aS,6aS,Z)-2-(tert-butyl)-1-(methoxyimino)-6,6-dimethyloctahydrocyclopropa[e]isoindol-3(2H)-one (3c):**

**Yield:** 18%**.**

**TLC**: (PE:EA=10:1, v/v): R*_f_*= 0.9, yellow liquid.

**^1^H NMR** (700 MHz, Methylene Chloride-*d*_2_) δ 3.83 (s, 3H), 3.64 (t, *J* = 11.0 Hz, 1H), 2.51 – 2.45 (m, 1H), 2.04 (t, *J* = 7.1 Hz, 1H), 1.91 (dd, *J* = 13.5, 3.0 Hz, 1H), 1.83 – 1.75 (m, 1H), 1.57 (s, 9H), 0.98 (s, 3H), 0.94 – 0.91 (m, 1H), 0.88 (d, *J* = 6.8 Hz, 1H), 0.86 (s, 3H), 0.76 – 0.72 (m, 1H).

**^13^C NMR** (176 MHz, CD_2_Cl_2_) δ 178.4, 161.1, 62.0, 58.9, 38.0, 29.4, 28.7, 28.5, 21.4, 20.7, 19.6, 18.4, 17.0, 16.1.

**HRMS (ESI)** for C_16_H_26_N_2_O_2_Na: calculated 301.1892 [M+Na]^+^, found 301.1880.

## **General procedure B: [3+2] cycloaddition for Terpene-Pyrrolidine Scaffold**

Procedure B1:

i) TFA (0.2 eq. diluted in DCM) was added dropwise into a solution of α, β-unsaturated terpene derived aldehyde or ketone (1.0 eq.) with *N*-(Methoxymethyl)-*N*-(trimethylsilylmethyl)benzylamine (1.2 eq., ^#^B1938, TCI) in dry DCM at 0 °C in 20 minutes and then the mixture was stirred at 0 °C over 16 h. A second portion of *N*-(Methoxymethyl)-*N*-(trimethylsilylmethyl) benzylamine (2.0 mL, 7.93 mol, 1.0 eq.) was slowly added dropwise (over 2 h) to the mixture until TLC monitoring showed complete consumption of the substrates. Sat. aq. NaHCO_3_ solution was added and continued to stir for 10 minutes The organic phase was separated, the aqueous phase extracted with DCM (2 × 50 mL), The combined organic phase was washed by sat. aq. NaCl, and then dried over Na_2_SO_4_ and the solvent was evaporated. Column chromatography (PE/EtOAc 15:1 → 5:1, v/v) afforded the related products.

**((3aS,4R,6R,7aS)-2-benzyl-5,5-dimethyloctahydro-3aH-4,6-methanoisoindol-3a-yl)methanol (8)：**

Following Method B1: From Myrtenal(2.0 g, 13.3 mmol, 1 eq.) and *N*-(methoxymethyl)-*N*-trimethylsilylmethyl) benzylamine (4.5 mL, 15.9 mmol, 1.2 eq., 90% pure) afforded a colorless solid **4a** (2.79 g, 9.8 mmol, 74%).

To a stirred solution of **4a** in MeOH (30 mL), NaBH₄ (451 mg, 11.9 mmol, 1.3 eq.) was added at 0 °C. The reaction mixture was stirred at room temperature for 3 h, then quenched with acetone. The solvent was removed under reduced pressure, and the residue was diluted with water. The mixture was extracted with ethyl acetate (2×), and the combined organic layers were dried over Mg_2_SO₄, filtered, and concentrated. The crude product was purified by column chromatography (R*_f_*= 0.2, PE/EtOAc = 4:1, v/v) to afford the liquid alcohol **8** (85%).

**^1^H NMR** (500 MHz, Chloroform-*d*) δ 7.32 (d, *J* = 4.5 Hz, 4H), 7.30 – 7.25 (m, 1H), 3.81 (d, *J* = 9.9 Hz, 1H), 3.68 (t, *J* = 10.8 Hz, 2H), 3.39 (d, *J* = 9.9 Hz, 1H), 3.36 (d, *J* = 8.6 Hz, 1H), 2.86 (d, *J* = 9.3 Hz, 1H), 2.59 – 2.52 (m, 1H), 2.50 (d, *J* = 9.5 Hz, 1H), 2.23 – 2.17 (m, 1H), 2.18 – 2.11 (m, 1H), 2.11 (d, *J* = 13.9 Hz, 1H), 1.89 (dq, *J* = 5.9, 3.0 Hz, 1H), 1.75 (dd, *J* = 6.4, 4.9 Hz, 1H), 1.56 (d, *J* = 13.5 Hz, 1H), 1.40 (d, *J* = 10.4 Hz, 1H), 1.22 (s, 3H), 0.95 (s, 3H).

**^13^C NMR** (126 MHz, CDCl_3_) δ 137.35, 129.01, 128.65, 127.65, 77.41, 77.16, 76.91, 71.37, 66.37, 64.24, 59.68, 50.95, 46.15, 41.26, 38.91, 32.91, 32.08, 27.62, 27.60, 24.36.

**HRMS** (ESI) for C_19_H_28_NO: calculated 286.2165 [M+H]^+^, found 286.2167.

[α]^20^_D_ = -53.7° (CHCl_3_, *c* = 1.0).

**(3aR,4S,6S,7aR)-2-benzyl-5,5-dimethyloctahydro-3aH-4,6-methanoisoindole-3a-carbaldehyde (4b):**

Following Method B1: From (*S*)-myrtenal^[13]^(2.0 g, 13.3 mmol, 1 eq) and *N*- methoxymethyl)-*N*-trimethylsilylmethyl) benzylamine (4.5 ml. 15.9 mmol, 1.2 eq., 90% pure) afforded a colorless liquid **4b** (R*_f_*= 0.6, PE:EA=7:1, v/v; 2.7 g, 10 mmol, 72%).

**^1^H NMR** (500 MHz, Methylene Chloride-*d*_2_) δ 9.64 (s, 1H), 7.28 (d, *J* = 6.2 Hz, 4H), 7.26 – 7.20 (m, 1H), 3.61 (d, *J* = 13.0 Hz, 1H), 3.50 (d, *J* = 13.0 Hz, 1H), 3.14 (t, *J* = 8.4 Hz, 1H), 2.90 – 2.85 (m, 1H), 2.66 (d, *J* = 10.2 Hz, 1H), 2.41 (d, *J* = 10.2 Hz, 1H), 2.32 – 2.26 (m, 2H), 2.26 – 2.20 (m, 1H), 2.19 – 2.10 (m, 1H), 1.87 (dq, *J* = 8.7, 3.0 Hz, 1H), 1.64 (d, *J* = 3.4 Hz, 1H), 1.62 (d, *J* = 3.3 Hz, 1H), 1.22 (s, 3H), 0.66 (s, 3H).

**^13^C NMR** (126 MHz, CD_2_Cl_2_)δ 205.9, 139.5, 128.9, 128.6, 127.3, 66.5, 61.3, 61.1, 59.8, 46.0, 40.8, 38.9, 32.2, 31.1, 26.6, 26.6, 23.6.

**HRMS** (ESI) for C_19_H_26_NO: calculated 284.2014 [M+H]^+^, found 284.2012.

[α]^20^_D_ = +56.1° (CHCl_3_, *c* = 1.0).

**(3aR,4aS,5aR,6aR)-2-benzyl-5,5-dimethyloctahydrocyclopropa[f]isoindole-3a(1H)-carbaldehyde (4c):**

Following Method B1: From 3-carene derived aldehyde^[14]^(274.9 mg, 1.83 mmol, 1 eq.) and *N*- methoxymethyl)-*N*-trimethylsilylmethyl) benzylamine (0.55 mL, 2.2 mmol, 1.2 eq., 90% pure) afforded a colorless liquid **4c** (R*_f_*= 0.7, PE:EA=7:1, v/v; 383.75 mg, 1.35 mmol, 74%).

**^1^H NMR** (500 MHz, Methylene Chloride-*d*_2_) δ 9.52 (s, 1H), 7.36 (s, 1H), 7.33 – 7.29 (m, 4H), 3.59 (d, *J* = 12.9 Hz, 1H), 3.52 (d, *J* = 12.9 Hz, 1H), 3.16 (d, *J* = 9.7 Hz, 1H), 3.06 (t, *J* = 8.1 Hz, 1H), 2.47 – 2.42 (m, 1H), 2.27 (d, *J* = 9.6 Hz, 1H), 2.19 (d, *J* = 9.1 Hz, 1H), 1.76 – 1.71 (m, 1H), 1.57 (dd, *J* = 14.9, 7.4 Hz, 1H), 1.07 (s, 3H), 1.01 (s, 3H), 0.97 (d, *J* = 9.0 Hz, 1H), 0.90 – 0.85 (m, 1H), 0.65 (ddd, *J* = 17.9, 9.1, 7.5 Hz, 2H).

**^13^C NMR** (126 MHz, CD_2_Cl_2_) δ 204.9, 139.7, 128.9, 128.6, 127.3, 60.2, 60.0, 59.9, 54.8, 37.2, 28.7, 20.1, 19.9, 19.4, 18.4, 17.8, 15.1.

**HRMS** (ESI) for C_19_H_26_NO: calculated 284.2009 [M+H]^+^, found: 284.2021.

[α]^20^_D_ = +26.2° (CHCl_3_, *c* = 1.0).

**((3aR,4aS,5aR,6aR)-2-benzyl-5,5-dimethyloctahydrocyclopropa[f]isoindol-3a(1H)-yl)methanol (S1):**

A stirred solution of compound **4c** (81.4 mg, 0.29 mmol) in MeOH (5 mL) was cooled to 0 °C, and NaBH₄ (17.3 mg, 0.45 mmol, 1.3 eq.) was added. The reaction mixture was stirred at room temperature for 1 h and then quenched with acetone. The solvent was removed under reduced pressure, and the residue was diluted with water. The aqueous layer was extracted with EtOAc (2×), and the combined organic extracts were dried over Na₂SO₄, filtered, and concentrated. The crude product was purified by column chromatography (petroleum ether/EtOAc = 5:1, v/v) to afford compound **S1** as a colorless liquid (R*_f_*= 0.2, PE:EA=3:1, v/v; 140 mg, 49%).

**^1^H NMR** (500 MHz, Chloroform-*d*) δ 7.32 – 7.25 (m, 4H), 7.23 (d, *J* = 6.8 Hz, 1H), 3.65 – 3.56 (m, 2H), 3.36 (d, *J* = 9.6 Hz, 1H), 3.24 (d, *J* = 9.8 Hz, 1H), 3.18 (t, *J* = 8.6 Hz, 1H), 2.92 (d, *J* = 9.3 Hz, 1H), 2.35 (d, *J* = 9.3 Hz, 1H), 2.29 (td, *J* = 9.1, 6.2 Hz, 1H), 2.12 (t, *J* = 6.0 Hz, 1H), 1.58 (ddd, *J* = 15.2, 6.9, 1.9 Hz, 1H), 1.47 – 1.42 (m, 1H), 0.99 (s, 3H), 0.91 (s, 3H), 0.89 (d, *J* = 5.9 Hz, 1H), 0.84 – 0.78 (m, 1H), 0.56 – 0.50 (m, 2H).

**^13^C NMR** (126 MHz, CDCl_3_) δ 129.9, 129.2, 128.7, 127.9, 72.7, 62.5, 60.3, 59.8, 44.5, 38.0, 28.8, 22.4, 19.2, 19.1, 18.5, 17.7, 15.1.

**HRMS** (ESI) for C_19_H_28_NO: calculated 286.2165 [M+H]^+^, found: 286.2199.

[α]^20^_D_ = +36.1° (CHCl_3_, *c* = 0.5).

**Methyl 2-benzylhexahydrocyclopenta[c]pyrrole-3a(1H)-carboxylate (4e):**

**Compound 4e** was synthesized according to the literature procedure^[1]^ (liquid, 1.87 g, 91%).

**^1^H NMR** (400 MHz, Chloroform-*d*) δ 7.30 – 7.22 (m, 4H), 7.21 – 7.15 (m, 1H), 3.62 (s, 3H), 3.53 (d, *J* = 13.2 Hz, 1H), 3.47 (d, *J* = 13.2 Hz, 1H), 2.87 (d, *J* = 9.3 Hz, 1H), 2.82 (dt, *J* = 7.9, 3.8 Hz, 1H), 2.62 (t, *J* = 8.2 Hz, 1H), 2.39 (d, *J* = 9.3 Hz, 1H), 2.26 (dd, *J* = 9.0, 4.3 Hz, 1H), 2.02 – 1.93 (m, 1H), 1.85 – 1.54 (m, 4H), 1.46 (t, *J* = 5.5 Hz, 1H).

**^13^C NMR** (101 MHz, CDCl_3_) δ 178.0, 139.3, 128.6, 128.3, 126.9, 63.7, 61.3, 59.7, 59.7, 52.1, 47.8, 38.4, 33.6, 26.9.

**HRMS** (ESI) for C_16_H_22_NO_2_: calculated 260.1645 [M+H]^+^, found 260.1664.

**(1R,2S,5R)-1'-benzyl-6,6-dimethylspiro[bicyclo [3.1.1]heptane-2,3'-pyrrolidin]-3-one (5a):**

Following Method B1: From pinocarvone^[13]^ (1.5 g, 10 mmol, 1 eq.) and *N*-methoxymethyl)-*N*-trimethylsilylmethyl) benzylamine **2** (3.4 mL, 12 mmol, 1.2 eq., 90% pure) afforded a colorless liquid **5a** (R*_f_*= 0.7, PE:EA=7:1, v/v; 2.3 g, 8.12 mmol, 81%).

**^1^H NMR** (500 MHz, Methylene Chloride-d_2_) δ 7.33 – 7.28 (m, 4H), 7.25 – 7.20 (m, 1H), 3.61 (d, *J* = 13.0 Hz, 1H), 3.52 (d, *J* = 13.0 Hz, 1H), 2.69 (dd, *J* = 8.5, 6.2 Hz, 1H), 2.65 (d, *J* = 9.2 Hz, 1H), 2.61 (td, *J* = 8.2, 5.7 Hz, 1H), 2.57 (t, *J* = 3.0 Hz, 1H), 2.52 – 2.48 (m, 1H), 2.47 (d, *J* = 2.7 Hz, 1H), 2.44 (d, *J* = 9.2 Hz, 1H), 2.25 (ddd, *J* = 12.5, 7.8, 6.1 Hz, 1H), 2.14 (t, *J* = 6.3 Hz, 1H), 2.06 (dq, *J* = 6.2, 3.1 Hz, 1H), 1.56 (ddd, *J* = 12.9, 7.2, 6.0 Hz, 1H), 1.31 (s, 3H), 1.14 (d, J = 10.8 Hz, 1H), 0.86 (s, 3H).

**^13^C NMR** (126 MHz, CD_2_Cl_2_) δ 217.1, 139.8, 129.0, 128.6, 127.2, 66.0, 60.6, 60.2, 55.4, 50.8, 44.7, 39.7, 38.7, 38.5, 31.8, 26.9, 22.5.

**HRMS** (ESI) for C_19_H_26_NO: calculated 284.2009 [M+H]^+^, found 284.2020.

[α]^20^_D_ = +18.1° (CHCl_3_, *c* = 1.0).

**(1S,2R,5S)-1'-benzyl-5-isopropylspiro[bicyclo[3.1.0]hexane-2,3'-pyrrolidin]-3-ol (S2):**

Following Method B1: From sabinene derived enone^[12]^(601 mg, 4 mmol, 1 eq.) and *N*-(methoxymethyl)-*N*-trimethylsilylmethyl) benzylamine (1.2 mL, 4.8 mmol, 1.2 eq., 90% pure) afforded a colorless liquid **5b** (1.2 g, 4 mmol, > 99%) with simply purification by column chromatography (R*_f_*= 0.15; PE:EA =15:1, v/v).

Then **5b** was dissolved into MeOH (5 mL), NaBH₄ (17.3 mg, 0.45 mmol, 1.3 eq.) was added into the reaction mixture at 0 °C. The reaction mixture was stirred at room temperature for 3 h, then quenched with acetone. The solvent was removed under reduced pressure, and the residue was diluted with water. The mixture was extracted with ethyl acetate (2×), and the combined organic layers were dried over Na_2_SO₄, filtered, and concentrated. The crude product was purified by column chromatography (PE/EA = 4:1, v/v) to afford **S2** as colorless liquid (R*_f_*= 0.2, PE:EA=3:1, v/v; 85.63 mg, 0.3 mmol, 75%).

**^1^H NMR** (400 MHz, Chloroform-*d*) δ 7.42 (t, *J* = 2.2 Hz, 5H), 5.61 (s, 1H), 4.29 (d, *J* = 12.7 Hz, 1H), 4.17 (d, *J* = 12.8 Hz, 1H), 3.87 – 3.62 (m, 2H), 3.20 (d, *J* = 9.8 Hz, 1H), 2.84 (d, *J* = 11.9 Hz, 1H), 2.56 (dd, *J* = 19.3, 2.8 Hz, 1H), 2.29 (d, *J* = 19.3 Hz, 1H), 2.25 – 2.09 (m, 2H), 1.75 – 1.64 (m, 1H), 1.50 – 1.35 (m, 1H), 0.95 (d, *J* = 6.7 Hz, 3H), 0.91 (d, *J* = 6.8 Hz, 3H), 0.85 (ddd, *J* = 8.5, 6.1, 2.7 Hz, 1H), 0.09 (dd, *J* = 6.1, 3.9 Hz, 1H).

**^13^C NMR** (101 MHz, CDCl_3_) δ 142.5, 130.5, 130.3, 129.6, 77.5, 77.4, 77.2, 76.8, 60.5, 58.7, 57.4, 53.4, 41.6, 32.3, 32.2, 29.5, 28.4, 19.9, 19.7, 17.4.

**HRMS** (ESI) for C_19_H_27_NO: calculated 286.2165 [M+H]^+^, found 286.2175.

**General procedure B2: [3+2] cycloaddition for Terpene-Pyrrolidine Scaffold**

Procedure B2:

PPh_3_ (17 mg, 6.5 mol%, 7.8 μmol) and AgOAc (10 mg, 6 mol%, 7.2 μmol) were dissolved in DCM and stirred at ambient temperature for 15 minutes. To the resulting solution were added iminoester (194 mg, 1.1 eq., 1.1 mmol), Cs_2_CO_3_ (130 mg, 40 mol%, 0.4 mmol), and (*R*)-myrtenal (0.15 mL, 1.0 eq., 1.0 mmol). The reaction mixture was stirred at ambient temperature for 12 h. The crude mixture was then directly loaded onto silica gel, and the product was purified by column chromatography using *n*-pentane/acetone (500:40) as eluent to afford the product as a solid (276.2 mg, 0.84 mmol).

**Methyl (4R,6R)-3a-formyl-5,5-dimethyl-3-phenyloctahydro-1H-4,6-methanoisoindole-1-carboxylate (4d):**

Following Method B2: From (*R*)-Myrtenal(1.5 g, 10 mmol, 1.0 eq) and iminoacetate (12 mmol, 1.2 eq., 90% pure) afforded a colorless solid **4d** (R*_f_*= 0.2, PE:EA=2:1, v/v; 2.52 g, 7.7 mmol, 77%).

**^1^H NMR** (600 MHz, Chloroform-*d*) δ 8.93 (s, 1H), 7.30 (d, *J* = 7.8 Hz, 2H), 7.26 – 7.23 (m, 1H), 7.19 – 7.15 (m, 2H), 4.36 (d, *J* = 2.6 Hz, 1H), 3.83 (d, *J* = 0.9 Hz, 3H), 3.68 (dt, *J* = 9.6, 1.9 Hz, 1H), 3.13 – 3.04 (m, 1H), 2.59 (dd, *J* = 6.3, 4.7 Hz, 1H), 2.54 – 2.46 (m, 1H), 2.22 – 2.13 (m, 1H), 2.02 – 1.92 (m, 2H), 1.62 (d, *J* = 10.8 Hz, 1H), 1.28 (s, 3H), 0.57 (s, 3H).

**^13^C NMR** (151 MHz, CDCl_3_) δ 207.2, 172.8, 135.6, 135.6, 128.7, 128.1, 127.3, 70.1, 69.5, 65.0, 52.4, 44.7, 40.4, 38.6, 38.0, 30.4, 26.7, 26.6, 22.9.

**HRMS (ESI)** for C_20_H_26_NO_3_: calculated 328.1907 [M+H]^+^, found 328.1904.

[α]^20^_D_ = -16.9° (CHCl_3_, *c* = 1.0).

## **General procedure C: Benzyl group cleavage**

**General procedure C1**

A degassed solution of the *N*-benzyl amine (1.0 eq.) in MeOH (0.1–0.2 M) was treated with ammonium formate (5.0 eq.) and Pd/C (10 wt%). The reaction mixture was stirred at 60 °C for 1 h. After cooling to room temperature, it was filtered through Celite, washed with EtOAc, and concentrated under reduced pressure to afford the corresponding amine.

**General procedure C2**

A degassed solution of the N-benzyl-protected amine (1.0 eq.) in EtOH/THF (4:1, 0.1–0.2 M) was treated with Pd/C (10 wt%) and stirred under a hydrogen atmosphere for 16–22 h. The reaction mixture was then filtered through Celite, washed with EtOAc, and concentrated under reduced pressure to afford the deprotected amine.

**General procedure C3**

A degassed solution of the N-benzyl-protected amine in MeOH (0.1 M) was treated with Pd/C (10 wt%, 0.2 eq.) and stirred under a hydrogen atmosphere for 16 h. The reaction mixture was then filtered through Celite, washed with MeOH, and concentrated under reduced pressure to yield the corresponding amine.

## **General procedure D: Sulfonamide formation**

**Procedure D1**

A solution of the amine (1.0 eq.) in DCM (0.2 M) was cooled to 0 °C, followed by the addition of NEt_3_ (2.0 eq.) and the respective sulfonyl chloride (1.0–1.2 eq.). The reaction mixture was stirred at room temperature for 2 h. The solvent was then removed under reduced pressure, and the residue was purified by column chromatography to afford the corresponding sulfonamide.

**Procedure D2**

A solution of the amine (1.0 eq.) in THF (0.1 M) was cooled to 0 °C, and NEt_3_ (1.2 eq.) followed by arenesulfonyl chloride (1.0 eq.) were added. The reaction mixture was stirred at 0 °C for 2 h to overnight. The solvent was removed under reduced pressure, and the residue was purified by column chromatography to afford the sulfonamide as a colorless solid.

**((3aS,4R,6R,7aS)-2-((4-(tert-butyl)phenyl)sulfonyl)-5,5-dimethyloctahydro-3aH-4,6-methanoisoindol-3a-yl)methanol (12):**

Following general procedure C3: From a degassed solution of **8** (1 eq.) in MeOH (0.1 M) was added Pd/C (10 wt%, 0.1 eq.) and it was stirred under a H_2_ atmosphere for 16 h. The reaction mixture was filtered over Celite, washed with MeOH and the solvent was evaporated to give a colorless solid (**11**, quant.).

Following general procedure D2: From **11** (242 mg, 1.24 mmol) and 4-*tert*-Butylbenzenesulfonyl chloride (288 mg, 1.24 mmol, 1.0 eq.) in THF (10 mL) at 0 °C afforded a colorless solid **12**^[17]^ withchromatography (PE/EtOAc 4:1 → 2:1, v/v) (R*_f_*= 0.2, PE:EA=2:1, v/v; 415 mg, 1.06 mmol, 86%).

**^1^H NMR** (500 MHz, Chloroform-*d*) δ 7.72 (d, *J* = 8.6 Hz, 2H), 7.53 (d, *J* = 8.6 Hz, 2H), 3.51 (d, *J* = 10.6 Hz, 1H), 3.42 (d, *J* = 10.6 Hz, 1H), 3.19 (dd, *J* = 9.1, 7.3 Hz, 1H), 3.06 (d, *J* = 9.7 Hz, 1H), 3.01 – 2.95 (m, 2H), 2.31 – 2.22 (m, 1H), 2.22 – 2.12 (m, 2H), 1.96 (dd, *J* = 6.5, 5.0 Hz, 1H), 1.86 (tt, *J* = 5.6, 2.9 Hz, 1H), 1.61 (ddd, *J* = 13.4, 4.4, 2.8 Hz, 1H), 1.35 (s, 9H), 1.31 (d, *J* = 10.4 Hz, 1H), 1.21 (s, 3H), 0.95 (s, 3H).

**^13^C NMR** (126 MHz, CDCl_3_) δ 156.6, 132.0, 128.1, 126.0, 77.4, 77.2, 76.9, 68.2, 58.5, 56.9, 52.3, 45.8, 40.4, 39.2, 35.3, 34.2, 34.1, 31.2, 27.5, 27.2, 23.7.

**HRMS** (ESI) for C_22_H_34_NO_3_S: calculated 392.2254 [M+H]^+^, found 392.2254.

[α]^20^_D_ = -26.1° (CHCl_3_, *c* = 1.0).

**((3aR,4S,6S,7aR)-2-((4-(tert-butyl)phenyl)sulfonyl)-5,5-dimethyloctahydro-3aH-4,6-methanoisoindol-3a-yl)methanol** (**S3**):

A stirred solution of **4b** (0.38 mmol, 1.0 eq.) in MeOH (4 mL) was cooled to 0 °C, and NaBH₄ (18.9 mg, 0.50 mmol, 1.3 eq.) was added portion wise. The reaction mixture was stirred at room temperature for 1 h before being quenched with acetone. The solvent was removed under reduced pressure, and the residue was diluted with water. The aqueous phase was extracted with ethyl acetate (2×), and the combined organic layers were dried over Na_2_SO_4_, filtered, and concentrated. The crude product was purified by column chromatography (petroleum ether/EtOAc = 4:1) to yield the corresponding alcohol as a colorless liquid (100 mg, >99%).

Following general procedure C3: From a degassed solution of the alcohol product obtained from the last step (0.2 mmol, 1 eq.) in MeOH (0.1 M) was added Pd/C (8 mg, 10 wt%, 0.1 eq.) and then the reaction mixture was stirred under a H_2_ atmosphere for 16 h. The reaction mixture was filtered over Celite, washed with MeOH and the solvent was evaporated to give a colorless solid without further purification for the next step.

Following general procedure D2: From the crude amine obtained from the last step (22 mg, 0.11 mmol) and 4-*tert*-Butylbenzenesulfonyl chloride (25.6 mg, 0.11 mmol, 1.0 eq.) in THF (2 mL) at 0 °C afforded a colorless solid **S3** withchromatography ((R*_f_*= 0.2, PE:EA=2:1, v/v), PE:EtOAc 4:1 → 2:1) (35.3 mg, 0.09 mmol, 80 %).

**^1^H NMR** (700 MHz, Chloroform-d) δ 7.72 (d, *J* = 8.5 Hz, 2H), 7.53 (d, *J* = 8.6 Hz, 2H), 3.51 (d, J = 10.5 Hz, 1H), 3.41 (d, *J* = 10.6 Hz, 1H), 3.20 (dd, *J* = 9.2, 7.3 Hz, 1H), 3.07 (d, *J* = 9.7 Hz, 1H), 3.00 – 2.95 (m, 2H), 2.26 (td, *J* = 11.5, 1.2 Hz, 1H), 2.20 – 2.13 (m, 2H), 1.96 (dd, *J* = 6.5, 5.0 Hz, 1H), 1.85 (tt, *J* = 5.6, 3.0 Hz, 1H), 1.60 (ddd, *J* = 13.5, 4.5, 2.9 Hz, 1H), 1.34 (s, 9H), 1.30 (d, *J* = 10.4 Hz, 1H), 1.21 (s, 3H), 0.94 (s, 3H).

**^13^C NMR** (176 MHz, CDCl_3_) δ 156.6, 132.1, 128.1, 126.0, 68.1, 58.5, 56.9, 52.3, 45.8, 40.4, 39.2, 35.3, 34.3, 34.1, 31.2, 27.5, 27.2, 23.7.

**HRMS** (ESI) for C_22_H_33_NO_3_SNa: calculated 414.2079 [M+Na]^+^, found 414.2068.

[α]^20^_D_ = +23.3° (CHCl_3_, *c* = 1.0).

**((3aR,4aS,5aR,6aR)-2-((4-(tert-butyl)phenyl)sulfonyl)-5,5-dimethyloctahydrocyclopropa[f]isoindol-3a(1H)-yl)methanol (S4):**

Following general procedure C3: From **S1** (40 mg, 0.14 mmol, 1 eq.) to afford a colorless liquid (6.3 mg, 0.13 mmol, 96%).

Following general procedure D2: From the above obtained amine(26.3 mg, 0.13 mmol 1 eq) and 4-*tert*-butylbenzenesulfonyl chloride (30.3 mg, 0.13 mmol, 1.0 eq.) to afford a colorless solid **S4** (R*_f_*= 0.2, PE:EA=2:1, v/v; 41 mg, 0.1 mmol, 77%).

**1H NMR** (500 MHz, Chloroform-d) δ 7.73 (d, *J* = 8.6 Hz, 2H), 7.54 (d, *J* = 8.5 Hz, 2H), 3.55 (t, *J* = 8.8 Hz, 1H), 3.43 (d, *J* = 10.4 Hz, 1H), 3.35 (d, *J* = 5.3 Hz, 1H), 3.33 (d, *J* = 4.6 Hz, 1H), 2.69 – 2.58 (m, 2H), 2.12 – 2.02 (m, 1H), 1.56 (ddt, *J* = 15.3, 5.0, 2.2 Hz, 2H), 1.34 (s, 9 H), 0.92 (s, 3H), 0.90 (s, 3H), 0.87 – 0.81 (m, 1H), 0.78 – 0.70 (m, 1H), 0.28 – 0.20 (m, 2H).

**^13^C NMR** (126 MHz, CDCl_3_) δ 156.8, 131.6, 128.1, 125.9, 70.2, 55.0, 53.7, 45.5, 38.2, 35.3, 31.2, 28.6, 23.3, 20.2, 19.1, 17.9, 17.6, 14.9.

**HRMS** (ESI) for C_22_H_34_NO_3_S: calculated 392.2254 [M+H]^+^, found: 392.2255.

[α]^20^_D_ = +9.3° (CHCl_3_, *c* = 0.5).

**(2-((4-(*tert*-butyl) phenyl) sulfonyl)hexahydrocyclopenta[c]pyrrol-3a(1H)-yl) methanol (S5):**

To a stirred suspension of LiAlH₄ (170 mg, 11.9 mmol, 1.3 eq.) in THF (20 mL) at 0 °C, a solution of compound **4e** (obtained from the previous step) in THF (0.3 M) was added dropwise. The reaction mixture was stirred at 0 °C for approximately 3 h, until complete consumption of the starting material. The reaction was then quenched with water and stirred for an additional 30 minutes The aqueous layer was extracted three times with diethyl ether, and the combined organic extracts were dried over anhydrous Na_2_SO_4_ and concentrated under reduced pressure. The resulting crude alcohol was used in the next step without further purification.

Following general procedure C3 to give a colorless liquid (126 mg, 0.645 mmol, 92 %). The crude amine was used in the next step without purification.

Following general procedure D2 to afforded a colorless solid **S5** (R*_f_*= 0.2, PE:EA=2:1, v/v; 785 mg, 2.33 mmol, 86%).

**^1^H NMR** (700 MHz, Methylene Chloride-d_2_) δ 7.69 (d, *J* = 8.5 Hz, 2H), 7.57 (d, *J* = 8.5 Hz, 2H), 3.40 (d, *J* = 2.3 Hz, 2H), 3.18 (dd, *J* = 9.6, 8.0 Hz, 1H), 3.09 (d, *J* = 9.7 Hz, 1H), 2.86 (d, *J* = 9.7 Hz, 1H), 2.82 (dd, *J* = 9.5, 4.5 Hz, 1H), 2.24 (tt, *J* = 8.1, 4.7 Hz, 1H), 1.81 – 1.73 (m, 1H), 1.64 – 1.56 (m, 2H), 1.55 – 1.47 (m, 2H), 1.43 – 1.37 (m, 1H), 1.35 (s, 9H).

**^13^C NMR** (176 MHz, CD_2_Cl_2_) δ 157.0, 132.4, 128.2, 126.3, 68.1, 57.2, 56.4, 55.0, 54.2, 54.0, 53.7, 53.5, 45.6, 35.8, 35.5, 32.9, 31.2, 25.8.

**HRMS** (ESI) for C_18_H_28_NO_3_S: calculated 338.1784 [M+H]^+^, found 338.1780.

**Methyl 2-((4-(tert-butyl)phenyl)sulfonyl)octahydro-3aH-isoindole-3a-carboxylate (S6):**

Following general procedure C3: A degassed solution of cyclohexane-pyrrolidine derived ester (2 mmol) that obtained according to the reference ^[1]^ in MeOH (10 mL) was added Pd/C (20%wt, 0.2 mmol). Then the reaction mixture was stirred under H_2_ atmosphere for 16 h. The reaction mixture was filtered over celite, washed with MeOH and the solvent was evaporated to give a colorless liquid. The crude alcohol was used in the next step without purification.

Following general procedure D2: From amine(241 mg, 1.56 mmol) and 4-*tert* butylbenzenesulfonyl chloride (1.56 mmol, 363 mg, 1.0 eq.) afforded a colorless solid **S6** (R*_f_*= 0.4, PE:EA=5:1, v/v; 415 mg, 1.06 mmol, 86%).

**^1^H NMR** (600 MHz, Chloroform-*d*) δ 7.74 (d, *J* = 8.6 Hz, 2H), 7.52 (d, *J* = 8.6 Hz, 2H), 3.52 (s, 3H), 3.47 (d, *J* = 10.1 Hz, 1H), 3.38 (d, *J* = 10.1 Hz, 1H), 3.30 (dd, *J* = 9.6, 7.6 Hz, 1H), 3.23 (dd, *J* = 9.7, 6.8 Hz, 1H), 2.56 – 2.50 (m, 1H), 1.75 – 1.69 (m, 1H), 1.65 (dt, *J* = 8.4, 5.3 Hz, 1H), 1.42 – 1.34 (m, 4H), 1.33 (s, 9H), 1.33 – 1.24 (m, 2H).

**^13^C NMR** (151 MHz, CDCl_3_) δ 175.0, 156.5, 134.3, 127.4, 126.1, 54.4, 52.3, 51.2, 51.0, 39.0, 35.3, 31.2, 28.9, 24.7, 22.1, 21.7.

**HRMS** (ESI) for C_20_H_30_NO_4_S: calculated 380.1890 [M+H]^+^, found 380.1888.

**(1R,2S,5R)-1'-((4-(tert-butyl)phenyl)sulfonyl)-6,6-dimethylspiro[bicyclo[3.1.1]heptane-2,3'-pyrrolidin]-3-ol (S7):**

To a stirred solution of **5a** (331 mg, 1.17 mmol, 1 eq.) in MeOH (10 mL), NaBH_4_ (94.5 mg, 2.5 mmol, 2.0 eq.) was added in portions at ice-bath. The reaction mixture was stirred at room temperature for 3 h, then quenched with acetone. The solvent was removed under reduced pressure, and the residue was diluted with water. The mixture was extracted with ethyl acetate (2×), and the combined organic layers were dried over Na_2_SO_4_, filtered, and concentrated. The crude product was purified by column chromatography (PE/EtOAc = 4:1, v/v) to afford the related alcohol product without further purification for next step (285 mg, 1 mmol, 85%).

Following general procedure C3: From the above obtained alcohol(285 mg, 1 mmol, 1 eq) to afford a colorless liquid without further purification for next step (180.9 mg, 0.93 mmol, 93%).

Following general procedure D2: From the above amine(180 mg, 0.92 mmol 1 eq.) and 4-*tert*-Butylbenzenesulfonyl chloride (214.1 mg, 0.92 mmol, 1.0 eq.) to afford a colorless solid **S7**(R*_f_*= 0.2, PE:EA=3:1, v/v; 79 mg, 0.2 mmol, 22% over three steps).

**^1^H NMR** (600 MHz, Chloroform-*d*) δ 7.72 (d, *J* = 8.5 Hz, 2H), 7.53 (d, *J* = 8.4 Hz, 2H), 4.07 (dd, *J* = 9.6, 6.8 Hz, 1H), 3.30 – 3.24 (m, 1H), 3.19 (d, *J* = 9.3 Hz, 1H), 3.10 (d, *J* = 4.6 Hz, 1H), 2.81 (d, *J* = 9.2 Hz, 1H), 2.54 (dt, *J* = 8.9, 4.6 Hz, 1H), 2.39 (td, *J* = 9.5, 4.8 Hz, 1H), 2.14 – 2.05 (m, 1H), 1.87 (t, *J* = 5.6 Hz, 1H), 1.69 (t, *J* = 6.0 Hz, 1H), 1.45 (ddd, *J* = 13.1, 9.7, 6.8 Hz, 2H), 1.35 (s, 9H), 1.16 (d, *J* = 10.4 Hz, 1H), 1.14 (s, 3H), 0.90 (s, 3H).

**^13^C NMR** (151 MHz, CDCl_3_) δ 156.5, 132.4, 127.8, 125.9, 70.3, 62.6, 51.9, 50.7, 48.6, 39.7, 39.6, 35.6, 35.2, 31.1, 30.5, 27.1, 27.0, 22.7.

**HRMS** (ESI) for C_22_H_34_NO_3_S: calculated 392.2254 [M+H]^+^, found 392.2252.

[α]^20^_D_ = -26.1° (CHCl_3_, *c* = 1.0).

**(1S,2R,3R,5S)-1'-((4-(tert-butyl)phenyl)sulfonyl)-5-isopropylspiro[bicyclo[3.1.0]hexane-2,3'-pyrrolidin]-3-ol (S8):**

Following general procedure C3: From **S2** (85.6 mg, 0.3 mmol, 1 eq.) to afford a colorless solid without purification (56.9 mg, 0.29 mmol, 97%).

Following general procedure D2: From the above obtained amine(56.9 mg, 0.29 mmol 1 eq.) and 4-*tert*-Butylbenzenesulfonyl chloride (67.5 mg, 0.29 mmol, 1.0 eq.) to afford a colorless solid **S8** and **S9** (R*_f_*= 0.2, PE:EA=3:1, v/v; 86 mg, 0.22 mmol, dr=1:1, 76% for two steps).

**^1^H NMR** (400 MHz, Chloroform-*d*) δ 7.76 (d, *J* = 8.5 Hz, 2H), 7.53 (d, *J* = 8.6 Hz, 2H), 3.80 – 3.71 (m, 1H), 3.38 (dd, *J* = 7.8, 6.1 Hz, 1H), 3.32 (dd, *J* = 7.8, 6.5 Hz, 1H), 3.08 – 2.98 (m, 2H), 2.04 (tdd, *J* = 14.1, 6.4, 1.6 Hz, 2H), 1.71 (ddd, *J* = 12.6, 7.8, 6.1 Hz, 1H), 1.63 (d, *J* = 14.2 Hz, 1H), 1.34 (s, 9H), 1.23 – 1.14 (m, 1H), 0.86 (d, *J* = 6.8 Hz, 3H), 0.84 (d, *J* = 3.8 Hz, 1H), 0.82 (d, *J* = 6.8 Hz, 3H), 0.59 (ddd, *J* = 8.5, 3.7, 1.3 Hz, 1H), 0.25 (ddd, *J* = 8.5, 4.8, 1.9 Hz, 1H).

**^13^C NMR** (101 MHz, CDCl_3_) δ 156.5, 134.1, 127.5, 126.1, 76.6, 58.4, 56.0, 47.6, 37.5, 35.3, 33.5, 32.6, 31.2, 30.2, 29.8, 20.1, 15.0.

**HRMS** (ESI) for C_22_H_34_NO_3_S: calculated 392.2254 [M+H]^+^, found 392.2247.

[α]^20^_D_ = +16.0° (CHCl_3_, *c* = 1.0).

**(1S,2R,3S,5S)-1'-((4-(*tert*-butyl)phenyl)sulfonyl)-5-isopropylspiro[bicyclo[3.1.0]hexane-2,3'-pyrrolidin]-3-ol (S9):**

**TLC**: (PE:EA=3:1, v/v): R*_f_*= 0.2.

**^1^H NMR** (400 MHz, Chloroform-*d*)δ 7.77 (d, *J* = 8.6 Hz, 2H), 7.51 (d, *J* = 8.6 Hz, 2H), 3.59 (dd, *J* = 9.4, 7.2 Hz, 1H), 3.49 (d, *J* = 9.8 Hz, 1H), 3.42 (ddd, *J* = 9.5, 8.2, 3.7 Hz, 1H), 3.28 (td, *J* = 9.3, 7.3 Hz, 1H), 3.01 – 2.93 (m, 1H), 1.97 (dd, *J* = 12.2, 7.2 Hz, 1H), 1.80 – 1.73 (m, 2H), 1.47 (ddd, *J* = 12.3, 9.4, 1.4 Hz, 1H), 1.32 (s, 9H), 1.18 – 1.05 (m, 1H), 0.87 (d, *J* = 6.7 Hz, 3H), 0.80 (d, *J* = 6.8 Hz, 3H), 0.61 (dd, *J* = 8.3, 3.8 Hz, 1H), 0.27 (dd, *J* = 5.5, 3.8 Hz, 1H), 0.12 (ddd, *J* = 8.4, 5.5, 1.3 Hz, 1H).

**^13^C NMR** (101 MHz, CDCl_3_) δ 156.4, 134.0, 127.6, 126.1, 74.6, 52.6, 52.3, 47.4, 35.3, 35.2, 33.4, 33.0, 31.2, 31.0, 29.8, 19.9, 19.8, 13.1.

**HRMS** (ESI) for C_22_H_34_NO_3_S: calculated 392.2254 [M+H]^+^, found 392.2250.

[α]^20^_D_ = -16.7° (CHCl_3_, *c* = 1.0).

**Methyl (4R,6R)-2-((4-(*tert*-butyl)phenyl)sulfonyl)-3a-formyl-5,5-dimethyl-3-phenyloctahydro-1H-4,6-methanoisoindole-1-carboxylate (S10):**

A mixture of **4d** (196 mg, 0.6 mmol), 4-(*tert*-butyl) benzenesulfonyl chloride (418.9 mg, 1.8 mmol) and K_2_CO_3_ (138.21 mg, 1.8 mmol) in dry CH_3_CN (10 mL) was stirred at 80 °C for 6 h. The reaction mixture was then concentrated under vacuum. The residue was dissolved in EtOAc and washed with H_2_O and brine. The organic layer was dried over Na_2_SO_4_, filtered, and concentrated. The crude residue was purified by flash column chromatography on silica gel (PE/EtOAc = 5:1) to afford **S10** (R*_f_*= 0.4, PE:EA=5:1, v/v; 197 mg, 0.38 mmol, 63%) as a yellow liquid.

**^1^H NMR** (500 MHz, Chloroform-*d*) δ 8.67 (s, 1H), 7.49 – 7.40 (m, 2H), 7.27 (d, *J* = 2.0 Hz, 1H), 7.26 (s, 1H), 7.20 – 6.76 (m, 5H), 4.70 (s, 1H), 4.42 (d, *J* = 8.0 Hz, 1H), 3.92 (s, 3H), 3.35 (ddd, *J* = 10.4, 8.0, 2.6 Hz, 1H), 2.46 (dd, *J* = 6.2, 4.8 Hz, 1H), 2.37 (dtd, *J* = 10.9, 6.1, 1.8 Hz, 1H), 2.24 (ddt, *J* = 14.0, 10.0, 2.0 Hz, 1H), 2.00 (dddd, *J* = 23.6, 10.9, 5.7, 3.3 Hz, 2H), 1.28 (s, 9H), 1.26 (d, *J* = 11.0 Hz, 1H), 1.20 (s, 3H), 0.48 (s, 3H).

**^13^C NMR** (126 MHz, CDCl_3_) δ 203.2, 172.3, 156.8, 134.6, 133.9, 128.3, 128.2, 128.2, 125.5, 72.8, 72.6, 64.6, 52.8, 45.9, 40.1, 37.8, 35.2, 34.2, 31.7, 31.2, 26.7, 26.5, 23.1.

**HRMS (ESI)** for C_30_H_38_NO_5_S: calculated 524.2465 [M+H]^+^, found 524.2470.

[α]^20^_D_ = -7.1° (CHCl_3_, *c* = 0.25).

**Methyl (4R,6R)-2-((4-(*tert*-butyl)phenyl)sulfonyl)-3a-(hydroxymethyl)-5,5-dimethyl-3-phenyloctahydro-1H-4,6-methanoisoindole-1-carboxylate (S11):**

A solution of **S10** (130.9 mg, 0.25 mmol, 1 eq.) in MeOH:THF=1:1 (4 mL) at 0 °C, NaBH_4_ (18.9 mg, 0.5 mmol, 2 eq.) was added in portions. After the mixture was stirred for 1 h, the reaction was quenched with acetone. The solvents were removed under reduced pressure and the residue was dissolved in water. The aqueous layer was extracted with ethyl acetate twice, the combined organic layers were dried over Na_2_SO_4_ and concentrated under reduced pressure. The crude product was purified by chromatography eluting with (PE:EtOAc=5:1, v/v) product obtained as colorless oil **S11** (R*_f_*= 0.3, PE:EA=3:1, v/v) with 41%.

**^1^H NMR** (600 MHz, Chloroform-*d*) δ 7.69 (d, *J* = 7.8 Hz, 1H), 7.56 (d, *J* = 8.6 Hz, 2H), 7.34 (d, *J* = 8.6 Hz, 2H), 7.25 – 7.22 (m, 1H), 7.16 – 7.13 (m, 1H), 7.07 – 7.03 (m, 1H), 6.96 (d, *J* = 7.6 Hz, 1H), 4.86 (s, 1H), 4.55 (d, *J* = 3.7 Hz, 1H), 3.82 (s, 3H), 3.40 (d, *J* = 12.4 Hz, 1H), 2.95 (d, *J* = 12.4 Hz, 1H), 2.80 – 2.74 (m, 1H), 2.58 – 2.50 (m, 1H), 2.24 (dtd, *J* = 10.6, 6.1, 2.2 Hz, 1H), 1.99 (dd, *J* = 6.4, 4.9 Hz, 1H), 1.91 (dq, *J* = 5.3, 2.7 Hz, 1H), 1.77 (ddd, *J* = 13.6, 5.6, 2.6 Hz, 1H), 1.30 (s, 9H), 1.22 (s, 3H), 0.97 (d, *J* = 2.2 Hz, 3H), 0.95 (s, 1H).

**^13^C NMR** (151 MHz, CDCl_3_) δ 173.2, 156.9, 138.6, 135.1, 128.8, 128.3, 128.1, 127.9, 127.7, 127.2, 125.6, 73.1, 72.7, 65.3, 58.8, 52.7, 47.3, 41.2, 39.4, 38.8, 36.3, 35.3, 31.2, 28.9, 27.9, 24.3.

**HRMS (ESI)** for C_30_H_40_NO_5_S: calculated 526.2549 [M+H]^+^, found 526.2470.

[α]^20^_D_ = -16.9° (CHCl_3_, *c* = 1.0).

**((3aS,4R,6R,7aS)-5,5-dimethyl-2-(pyridin-3-ylsulfonyl)octahydro-3aH-4,6-methanoisoindol-3a-yl)methanol (S12):**

Following general procedure D2: From **11** (58.59 mg, 0.3 mmol) and pyridine-3-sulfonyl chloride (53.2 mg, 1.0 eq.). Purification by column chromatography (PE/EtOAc = 4:1 to 2:1, v/v) afforded a colorless solid **S12** (R*_f_*= 0.2, PE:EA=1:1, v/v; 66.2 mg, 0.20 mmol, 66%).

**^1^H NMR** (700 MHz, Chloroform-*d*) δ 9.06 – 8.98 (m, 1H), 8.86 – 8.78 (m, 1H), 8.10 (dt, *J* = 8.0, 2.0 Hz, 1H), 7.54 – 7.46 (m, 1H), 3.45 (d, *J* = 2.2 Hz, 2H), 3.19 (dd, *J* = 9.1, 7.2 Hz, 1H), 3.10 – 3.03 (m, 3H), 2.33 – 2.27 (m, 1H), 2.25 – 2.18 (m, 2H), 1.94 (dd, *J* = 6.5, 5.0 Hz, 1H), 1.88 (dt, *J* = 5.4, 2.7 Hz, 1H), 1.61 (ddd, *J* = 13.4, 4.6, 2.8 Hz, 1H), 1.30 (d, *J* = 10.4 Hz, 1H), 1.22 (s, 3H), 0.95 (s, 3H).

**^13^C NMR** (176 MHz, CDCl_3_) δ 153.4, 148.9, 135.9, 132.0, 123.8, 67.9, 58.6, 56.8, 52.5, 46.2, 40.4, 39.2, 34.4, 34.4, 27.5, 27.4, 23.7.

**HRMS** (ESI) for C_17_H_25_N_2_O_3_S: calculated 337.1580 [M+H]^+^, found 337.1574.

[α]^20^_D_ = -19.2° (CHCl_3_, *c* = 0.25).

**((3aS,4R,6R,7aS)-5,5-dimethyl-2-((6-methylpyridin-3-yl)sulfonyl)octahydro-3aH-4,6-methanoisoindol-3a-yl)methanol (S13):**

Following general procedure D2: From **11** (39.1 mg, 0.2 mmol) and 6-methylpyridine-3-sulfonyl chloride (38.37 mg, 1.0 eq.). Purification by column chromatography (PE/EtOAc 4:1 to 2:1, v/v) afforded a colorless solid **S13** (R*_f_*= 0.2, PE:EA=1:1, v/v; 50 mg, 0.15 mmol, 75%).

**^1^H NMR** (700 MHz, Chloroform-*d*) δ 8.90 – 8.86 (m, 1H), 7.97 (dd, *J* = 8.1, 2.3 Hz, 1H), 7.32 (d, *J* = 8.1 Hz, 1H), 3.45 (q, *J* = 10.5 Hz, 2H), 3.17 (dd, *J* = 9.0, 7.2 Hz, 1H), 3.06 (d, *J* = 9.7 Hz, 1H), 3.03 (td, *J* = 5.2, 2.8 Hz, 2H), 2.65 (s, 3H), 2.29 (dddd, *J* = 13.5, 10.5, 3.3, 2.1 Hz, 1H), 2.21 (dqt, *J* = 10.4, 6.2, 2.6 Hz, 2H), 1.94 (dd, *J* = 6.5, 5.0 Hz, 1H), 1.87 (dt, *J* = 5.4, 2.7 Hz, 1H), 1.61 (ddd, *J* = 13.5, 4.6, 2.8 Hz, 1H), 1.31 (d, *J* = 10.4 Hz, 1H), 1.21 (s, 3H), 0.94 (s, 3H).

**^13^C NMR** (176 MHz, CDCl_3_) δ 163.4, 148.4, 136.2, 129.0, 123.4, 77.3, 77.2, 77.0, 67.9, 58.6, 56.8, 52.4, 46.1, 40.4, 39.2, 34.4, 34.3, 27.5, 27.4, 24.8, 23.7.

**HRMS** (ESI) for C_18_H_27_N_2_O_3_S: calculated 351.1737 [M+H]^+^, found 351.1739.

[α]^20^_D_ = -19.1° (CHCl_3_, *c* = 1.0).

**((3aS,4R,6R,7aS)-5,5-dimethyl-2-(thiophen-2-ylsulfonyl)octahydro-3aH-4,6-methanoisoindol-3a-yl)methanol (S14):**

Following general procedure D2: From **11** (58.59 mg, 0.3 mmol) and thiophene-2-sulfonyl chloride (54.8 mg, 1.0 eq.). Purification by column chromatography (PE/EtOAc 4:1 to 2:1, v/v) afforded a colorless solid **S14** (R*_f_*= 0.3, PE:EA=1:1, v/v; 95.5 mg, 0.28 mmol, 93%).

**^1^H NMR** (700 MHz, Chloroform-d) δ 7.62 (dd, *J* = 5.0, 1.3 Hz, 1H), 7.58 (dd, *J* = 3.8, 1.3 Hz, 1H), 7.16 (dd, *J* = 5.0, 3.7 Hz, 1H), 3.51 (d, *J* = 10.5 Hz, 1H), 3.45 (d, *J* = 10.5 Hz, 1H), 3.25 (dd, *J* = 9.2, 7.3 Hz, 1H), 3.14 (d, *J* = 9.8 Hz, 1H), 3.06 (d, *J* = 10.2 Hz, 1H), 3.05 – 3.03 (m, 1H), 2.29 (ddd, *J* = 13.2, 3.1, 2.0 Hz, 1H), 2.26 – 2.19 (m, 2H), 1.96 (dd, *J* = 6.5, 5.0 Hz, 1H), 1.88 (dt, *J* = 5.4, 2.7 Hz, 1H), 1.64 (ddd, *J* = 13.3, 4.4, 2.8 Hz, 1H), 1.34 (d, *J* = 10.4 Hz, 1H), 1.23 (s, 3H), 0.96 (s, 3H).

**^13^C NMR** (176 MHz, CDCl3) δ 135.2, 133.0, 132.2, 127.7, 68.2, 58.9, 57.2, 52.5, 46.1, 40.4, 39.1, 34.4, 34.3, 27.5, 27.4, 23.8.

HRMS (ESI) for C_16_H_24_NO_3_S_2_: calculated 342.1192 [M+H]^+^, found 342.1194.

[α]^20^_D_ = -98.3° (CHCl_3_, c = 0.1).

**((3a*S*,4*R*,6*R*,7a*S*)-5,5-dimethyl-2-(naphthalen-2-ylsulfonyl)octahydro-3a*H*-4,6-methanoisoindol-3a-yl)methanol (S15):**

Following general procedure D2: From **11** (329.3 mg, 1.69 mmol) and naphthalene-2-sulfonyl chloride (1.0 eq.). Purification by column chromatography (PE/EtOAc 4:1, v/v) afforded a colorless solid **S15** (R*_f_*= 0.3, PE:EA=2:1, v/v; 448 mg, 1.1 mmol, 65%).

**^1^H NMR** (700 MHz, CDCl_3_)δ 8.38 (s, 1H), 8.01 – 7.96 (m, 2H), 7.93 (d, *J* = 8.1 Hz, 1H), 7.81 (dd, *J* = 8.5, 1.8 Hz, 1H), 7.67 – 7.64 (m, 1H), 7.64 – 7.60 (m, 1H), 3.47 (d, *J* = 10.4 Hz, 1H), 3.39 (d, *J* = 10.4 Hz, 1H), 3.26 (dd, *J* = 9.2, 7.3 Hz, 1H), 3.12 (d, *J* = 9.7 Hz, 1H), 3.06 (dd, *J* = 9.4, 3.2 Hz, 2H), 2.30 – 2.24 (m, 1H), 2.20 – 2.14 (m, 2H), 1.95 (dd, *J* = 6.4, 5.0 Hz, 1H), 1.85 (tt, *J* = 5.7, 3.0 Hz, 1H), 1.61 (ddd, *J* = 13.5, 4.6, 2.9 Hz, 1H), 1.31 (d, *J* = 10.4 Hz, 1H), 1.21 (s, 3H), 0.93 (s, 3H).

**^13^C NMR** (176 MHz, CDCl_3_) δ 135.1, 132.4, 132.3, 129.5, 129.5, 129.2, 128.9, 128.1, 127.6, 123.6, 68.1, 58.6, 57.0, 52.4, 45.9, 40.4, 39.2, 34.3, 34.2, 27.5, 27.3, 23.7.

**HRMS** (ESI) for C_22_H_27_NO_3_SNa: calculated 408.1609 [M+Na]^+^, found 409.1591.

[α]^20^_D_ = -13.3° (CHCl_3_, *c* = 0.5).

**((3aS,4R,6R,7aS)-5,5-dimethyl-2-(phenylsulfonyl)octahydro-3a*H*-4,6-methanoisoindol-3a-yl)methanol (S16):**

Following general procedure D2: From **11** (39.6 mg, 0.2 mmol) and benzenesulfonyl chloride (1.0 eq.). Purification by column chromatography (PE/EtOAc 4:1, v/v) afforded a colorless solid **S16** (R*_f_*= 0.3, PE:EA=2:1, v/v; 90%).

**^1^H NMR** (500 MHz, CDCl_3_) δ 7.84 –7.79 (m, 2H), 7.64 – 7.57 (m, 1H), 7.58 – 7.51 (m, 2H), 3.49 (d, *J* = 10.5 Hz, 1H), 3.41 (d, *J* = 10.5 Hz, 1H), 3.19 (dd, *J* = 9.1, 7.3 Hz, 1H), 3.06 (d, *J* = 9.7 Hz, 1H), 3.02 – 2.95 (m, 2H), 2.31 – 2.23 (m, 1H), 2.21 – 2.14 (m, 2H), 1.98 – 1.92 (m, 1H), 1.86 (dt, *J* = 5.3, 2.7 Hz, 1H), 1.60 (ddd, *J* = 13.4, 4.4, 2.8 Hz, 1H), 1.29 (d, *J* = 10.5 Hz, 1H), 1.21 (s, 3H), 0.94 (s, 3H).

**^13^C NMR** (126 MHz, CDCl_3_) δ 135.0, 132.9, 129.0, 128.2, 68.1, 58.6, 56.9, 52.3, 45.9, 40.4, 39.2, 34.3, 34.1, 27.5, 27.2, 23.7.

**HRMS** (ESI) for C_18_H_25_NO_3_SNa: calculated 358.1453 [M+Na]^+^, found 358.1445.

[α]^20^_D_ = -18.7° (CHCl_3_, *c* = 0.5).

**((3a*S*,4*R*,6*R*,7a*S*)-5,5-dimethyl-2-(*o*-tolylsulfonyl)octahydro-3a*H*-4,6-methanoisoindol-3a-yl)methanol (S17):**

Following general procedure D2: From **11** (39.6 mg, 0.2 mmol) and 2-methylbenzenesulfonyl chloride (1.0 eq.). Purification by column chromatography (PE/EtOAc 4:1, v/v) afforded a colorless solid **S17** (R*_f_*= 0.3, PE:EA=2:1, v/v; 52.4 mg, 0.15 mmol, 75%).

**^1^H NMR** (700 MHz, CDCl_3_) δ 7.91 (dd, *J* = 8.2, 1.4 Hz, 1H), 7.45 (td, *J* = 7.5, 1.4 Hz, 1H), 7.33 – 7.29 (m, 2H), 3.54 (d, *J* = 10.6 Hz, 1H), 3.48 (d, *J* = 10.5 Hz, 1H), 3.41 (dd, *J* = 9.4, 7.3 Hz, 1H), 3.24 (d, *J* = 9.8 Hz, 1H), 3.03 (dd, *J* = 9.3, 3.6 Hz, 1H), 2.96 (d, *J* = 9.8 Hz, 1H), 2.67 (s, 3H), 2.43 (d, *J* = 3.1 Hz, 1H), 2.26 (dt, *J* = 12.9, 2.6 Hz, 1H), 2.23 – 2.20 (m, 1H), 2.17 (ddd, *J* = 10.5, 5.2, 3.2 Hz, 1H), 1.95 (dd, *J* = 6.5, 5.0 Hz, 1H), 1.88 – 1.84 (m, 1H), 1.58 – 1.55 (m, 1H), 1.27 (d, *J* = 10.4 Hz, 1H), 1.21 (s, 3H), 0.96 (s, 3H).

**^13^C NMR** (176 MHz, CDCl_3_) δ 138.4, 135.4, 133.0, 132.8, 130.4, 126.2, 68.1, 58.0, 56.2, 52.5, 45.9, 40.4, 39.2, 34.3, 34.2, 27.5, 27.3, 23.7, 21.1.

**HRMS** (ESI) for C_19_H_27_NO_3_SNa: calculated 372.1609 [M+Na]^+^, found 372.1591.

[α]^20^_D_ = -2.7° (CHCl_3_, *c* = 1.0).

**((3aS,4R,6R)-5,5-dimethyl-2-(m-tolylsulfonyl)octahydro-3aH-4,6-methanoisoindol-3a-yl)methanol (S18):**

Following general procedure D2: From **11** (39.6 mg, 0.2 mmol) and 3-methylbenzenesulfonyl chloride (1.0 eq.). Purification by column chromatography (PE/EtOAc 4:1, v/v) afforded a colorless solid **S18** (R*_f_*= 0.3, PE:EA=2:1, v/v; 53 mg, 0.15 mmol, 76%).

**^1^H NMR** (500 MHz, CDCl_3_) δ 7.61 (s, 1H), 7.59 (d, *J* = 7.0 Hz, 1H), 7.44 – 7.38 (m, 2H), 3.49 (d, *J* = 10.6 Hz, 1H), 3.40 (d, *J* = 10.6 Hz, 1H), 3.19 (dd, *J* = 9.1, 7.2 Hz, 1H), 3.05 (d, *J* = 9.7 Hz, 1H), 2.97 (d, *J* = 2.4 Hz, 1H), 2.96 (dd, *J* = 5.4, 3.7 Hz, 1H), 2.43 (s, 3H), 2.30 – 2.23 (m, 1H), 2.20 – 2.13 (m, 2H), 1.95 (dd, *J* = 6.5, 4.9 Hz, 1H), 1.85 (dq, *J* = 5.7, 2.9 Hz, 1H), 1.59 (ddd, *J* = 13.4, 4.5, 2.9 Hz, 1H), 1.29 (d, *J* = 10.4 Hz, 1H), 1.21 (s, 3H), 0.94 (s, 3H).

**^13^C NMR** (126 MHz, CDCl_3_) δ 139.2, 134.8, 133.8, 128.9, 128.5, 125.3, 68.0, 58.5, 56.9, 52.3, 45.9, 40.4, 39.2, 34.2, 34.1, 27.5, 27.2, 23.7, 21.6.

**HRMS** (ESI) for C_19_H_27_NO_3_SNa: calculated 372.1609 [M+Na]^+^, found 372.1598.

[α]^20^_D_ = -45.3° (CHCl_3_, *c* = 1.0).

**((3a*S*,4*R*,6*R*,7a*S*)-5,5-dimethyl-2-tosyloctahydro-3a*H*-4,6-methanoisoindol-3a-yl) methanol (S19):**

Following general procedure D2: From **11** (39.6 mg, 0.2 mmol) and 4-methylbenzenesulfonyl chloride (1.0 eq.). Purification by column chromatography (PE/EtOAc 4:1, v/v) afforded a colorless solid **S19** (R*_f_*= 0.3, PE:EA=2:1, v/v; 56 mg, 0.16 mmol, 80%).

**^1^H NMR** (700 MHz, CDCl_3_) δ 7.68 (d, *J* = 8.3 Hz, 2H), 7.32 (d, *J* = 8.1 Hz, 2H), 3.49 (d, *J* = 10.5 Hz, 1H), 3.40 (d, *J* = 10.5 Hz, 1H), 3.17 (dd, *J* = 9.1, 7.3 Hz, 1H), 3.04 (d, *J* = 9.7 Hz, 1H), 2.97 – 2.92 (m, 2H), 2.43 (s, 3H), 2.28 – 2.22 (m, 1H), 2.19 – 2.12 (m, 2H), 1.95 (dd, *J* = 6.4, 5.0 Hz, 1H), 1.84 (tt, *J* = 5.6, 3.0 Hz, 1H), 1.59 (ddd, *J* = 13.5, 4.5, 2.8 Hz, 1H), 1.29 (d, *J* = 10.4 Hz, 1H), 1.21 (s, 3H), 0.94 (s, 3H).

**^13^C NMR** (176 MHz, CDCl_3_) δ 143.7, 131.9, 129.7, 128.3, 68.1, 58.5, 56.9, 52.3, 45.9, 40.4, 39.2, 34.2, 34.1, 27.5, 27.2, 23.7, 21.7.

**HRMS** (ESI) for C_19_H_27_NO_3_SNa: calculated 372.1609 [M+Na]^+^, found 372.1603.

[α]^20^_D_ = -15.7° (CHCl_3_, *c* = 1.0).

**((3a*S*,4*R*,6*R*,7a*S*)-2-((4-isopropylphenyl)sulfonyl)-5,5-dimethyloctahydro-3a*H*-4,6-methanoisoindol-3a-yl)methanol (S20)：**

Following general procedure D2: From **11** (39.6 mg, 0.2 mmol) and 4-isopropylbenzenesulfonyl chloride (44.8 µl, 1.0 eq.). Purification by column chromatography (PE/EtOAc 10:1 to 4:1, v/v) afforded a colorless solid **S20** (R*_f_*= 0.32, PE:EA=2:1, v/v; 66 mg, 0.176 mmol, 88 %).

**1H NMR** (500 MHz, CDCl_3_) δ 7.72 (d, *J* = 8.4 Hz, 2H), 7.37 (d, *J* = 8.3 Hz, 2H), 3.50 (d, *J* = 10.5 Hz, 1H), 3.41 (d, *J* = 10.6 Hz, 1H), 3.20 (dd, *J* = 9.1, 7.3 Hz, 1H), 3.06 (d, *J* = 9.7 Hz, 1H), 3.01 – 2.96 (m, 2H), 2.95 (dd, *J* = 6.1, 3.1 Hz, 1H), 2.30 – 2.23 (m, 1H), 2.20 – 2.12 (m, 2H), 1.96 (dd, *J* = 6.5, 5.0 Hz, 1H), 1.85 (tt, *J* = 5.7, 2.9 Hz, 1H), 1.60 (ddd, *J* = 13.4, 4.4, 2.8 Hz, 1H), 1.29 (d, *J* = 9.1 Hz, 1H), 1.27 (d, *J* = 6.9 Hz, 6H), 1.21 (s, 3H), 0.94 (s, 3H).

**13C NMR** (126 MHz, CDCl_3_) δ 154.3, 132.3, 128.4, 127.1, 68.1 58.5, 56.9, 52.3, 45.8, 40.4, 39.2, 34.3, 34.2, 34.0, 27.5, 27.2, 23.8, 23.8, 23.7.

**HRMS** (ESI) for C_21_H_31_NO_3_SNa: calculated 400.1922 [M+Na]^+^, found 400.1917.

[α]^20^_D_ = -64.3° (CHCl_3_, *c* = 1.0).

**((3a*S*,4*R*,6*R*,7a*S*)-2-([1,1'-biphenyl]-4-ylsulfonyl)-5,5-dimethyloctahydro-3a*H*-4,6-methanoisoindol-3a-yl)methanol (S21)**

Following general procedure D2: From **11** (39.6 mg, 0.2 mmol) and biphenyl sulfonyl chloride (63.2 mg, 0.2 mmol, 1.0 eq.). Purification by column chromatography (PE/EtOAc 10 :1 to 4:1, v/v) afforded a colorless solid **S21** (R*_f_*= 0.2, PE:EA=2:1, v/v; 65.8 mg, 0.16 mmol, 80%).

**^1^H NMR** (500 MHz, CDCl_3_) δ 7.87 (dd, *J* = 8.5, 2.0 Hz, 2H), 7.75 (dd, *J* = 8.5, 2.1 Hz, 2H), 7.66 – 7.60 (m, 2H), 7.52 – 7.45 (m, 2H), 7.43 (dd, *J* = 7.6, 1.7 Hz, 1H), 3.54 – 3.48 (m, 1H), 3.43 (dd, *J* = 10.5, 4.1 Hz, 1H), 3.24 (dd, *J* = 9.1, 7.2 Hz, 1H), 3.11 (d, *J* = 9.7 Hz, 1H), 3.06 – 2.99 (m, 2H), 2.34 – 2.24 (m, 1H), 2.23 – 2.15 (m, 2H), 1.97 (dd, *J* = 6.4, 5.0 Hz, 1H), 1.87 (dq, *J* = 5.7, 2.9 Hz, 1H), 1.63 (ddd, *J* = 13.4, 4.5, 2.9 Hz, 2H), 1.34 (d, *J* = 10.4 Hz, 1H), 1.22 (s, 3H), 0.95 (s, 3H).

**^13^C NMR** (126 MHz, CDCl_3_) δ 145.7, 139.4, 133.7, 133.6, 129.2, 128.8, 128.6 127.6, 127.5, 68.1, 58.6, 57.0, 52.3, 45.9, 40.4, 39.2, 34.3, 34.2, 27.5, 27.3, 23.7.

**HRMS** (ESI) for C_24_H_29_NO_3_SNa: calculated 434.1766 [M+Na]^+^, found 434.1757.

[α]^20^_D_ = -17.3° (CHCl_3_, *c* = 1.0).

**((3a*S*,4*R*,6*R*,7a*S*)-2-((4-cyclohexylphenyl)sulfonyl)-5,5-dimethyloctahydro-3a*H*-4,6-methanoisoindol-3a-yl)methanol（S22）:**

Following general procedure D2: From **11** (29.3 mg, 0.15 mmol) and 4-cyclohexylbenzenesulfonyl chloride (42.7 mg,1.0 eq.). Purification by column chromatography (PE/EtOAc 10:1 to 4:1, v/v) afforded a colorless solid **S22** (R*_f_*= 0.3, PE:EA=2:1, v/v; 44.9 mg, 0.11 mmol, 72%).

**^1^H NMR** (700 MHz, CDCl_3_) δ 7.71 (d, *J* = 8.3 Hz, 2H), 7.36 (d, *J* = 8.3 Hz, 2H), 3.51 (d, *J* = 10.5 Hz, 1H), 3.42 (d, *J* = 10.5 Hz, 1H), 3.20 (dd, *J* = 9.1, 7.3 Hz, 1H), 3.06 (d, *J* = 9.7 Hz, 1H), 3.00 – 2.96 (m, 2H), 2.96 (d, *J* = 3.7 Hz, 1H), 2.58 (ddd, *J* = 11.5, 8.7, 3.3 Hz, 1H), 2.30 – 2.23 (m, 1H), 2.20 – 2.13 (m, 2H), 1.95 (dd, *J* = 6.4, 5.0 Hz, 1H), 1.92 – 1.83 (m, 5H), 1.77 (d, *J* = 13.3 Hz, 1H), 1.63 – 1.58 (m, 1H), 1.46 – 1.37 (m, 4H), 1.30 (d, *J* = 10.4 Hz, 1H), 1.27 (dd, *J* = 12.7, 3.6 Hz, 2H), 1.22 (s, 3H), 0.95 (s, 3H).

**^13^C NMR** (176 MHz, CDCl_3_) δ 153.5, 132.3, 128.4, 127.5, 68.2, 58.5, 56.9, 52.3, 45.9, 44.7, 40.4, 39.2, 34.3, 34.2, 34.1, 27.5, 27.2, 26.8, 26.1, 23.8.

**HRMS** (ESI) for C_24_H_35_NO_3_SNa: calculated 440.2235 [M+Na]^+^, found 440.2217.[α]^20^_D_ = -10.3° (CHCl_3_, *c* = 0.2).

**((3a*S*,4*R*,6*R*,7a*S*)-2-(mesitylsulfonyl)-5,5-dimethyloctahydro-3a*H*-4,6-methanoisoindol-3a-yl)methanol (S23):**

Following general procedure D2: From **11** (39.6 mg, 0.2 mmol) and mesitylenesulfonyl chloride (1.0 eq.). Purification by column chromatography (PE/EtOAc 10:1 to 5:1, v/v) afforded a colorless solid **S23** (R*_f_*= 0.3, PE:EA=2:1, v/v; 66.5 mg, 0.176 mmol, 88%).

**1H NMR** (700 MHz, CDCl_3_) δ 6.94 (s, 2H), 3.56 (d, *J* = 10.6 Hz, 1H), 3.50 (d, *J* = 10.6 Hz, 1H), 3.45 (dd, *J* = 9.1, 6.8 Hz, 1H), 3.28 (d, *J* = 9.6 Hz, 1H), 3.01 (dd, *J* = 9.1, 3.0 Hz, 1H), 2.88 (d, *J* = 9.6 Hz, 1H), 2.64 (s, 6H), 2.29 (s, 3H), 2.26 – 2.20 (m, 2H), 2.16 – 2.13 (m, 1H), 1.94 (dd, *J* = 6.4, 5.0 Hz, 1H), 1.85 (dt, *J* = 5.6, 2.7 Hz, 1H), 1.54 – 1.51 (m, 1H), 1.23 (d, *J* = 10.3 Hz, 1H), 1.20 (s, 3H), 0.96 (s, 3H).

**13C NMR** (176 MHz, CDCl_3_) δ 142.7, 140.4, 132.0, 131.7, 68.2, 57.1, 55.5, 52.4, 45.8, 40.5, 39.2, 34.2, 34.1, 27.5, 27.3, 23.7, 23.2, 21.1.

**HRMS** (ESI) for C_21_H_31_NO_3_SNa: calculated 400.1922 [M+Na]^+^, found 400.1912.

[α]^20^_D_ = -4.3° (CHCl_3_, *c* = 1.0).

**((3a*S*,4*R*,6*R*,7a*S*)-5,5-dimethyl-2-((5,5,8,8-tetramethyl-5,6,7,8-tetrahydronaphthalen-2-yl)sulfonyl)octahydro-3aH-4,6-methanoisoindol-3a-yl)methanol (S24) :**

Following general procedure D2: From **11** (48.8 mg, 0.25 mmol) and benzenesulfonyl chloride (71.7 mg, 0.25 mmol, 1.0 eq.). Purification by column chromatography (PE/EtOAc 10:1 to 4:1, v/v) afforded a colorless solid **S24** (R*_f_*= 0.35, PE:EA=2:1, v/v; 84.1 mg, 0.18 mmol, 75%).

**^1^H NMR** (500 MHz, CDCl_3_) δ 7.71 (d, *J* = 2.0 Hz, 1H), 7.53 (dd, *J* = 8.3, 2.0 Hz, 1H), 7.44 (d, *J* = 8.3 Hz, 1H), 3.51 (d, *J* = 10.5 Hz, 1H), 3.43 (d, *J* = 10.5 Hz, 1H), 3.22 (dd, *J* = 9.1, 7.3 Hz, 1H), 3.09 (d, *J* = 9.7 Hz, 1H), 2.96 (d, *J* = 9.8 Hz, 1H), 2.93 (dd, *J* = 9.2, 3.8 Hz, 1H), 2.30 – 2.22 (m, 1H), 2.20 – 2.13 (m, 2H), 1.95 (dd, *J* = 6.5, 5.0 Hz, 1H), 1.86 (td, *J* = 5.5, 2.9 Hz, 1H), 1.71 (s, 4H), 1.61 – 1.56 (m, 1H), 1.30 (s, 3H), 1.30 (s, 9H), 1.26 (d, *J* = 10.4 Hz, 1H), 1.21 (s, 3H), 0.95 (s, 3H).

**^13^C NMR** (126 MHz, CDCl_3_) δ 150.5, 146.1, 132.0, 127.4, 126.6, 125.1, 68.3, 58.5, 56.9, 52.3, 45.9, 40.4, 39.2, 34.8, 34.8, 34.8, 34.7, 34.2, 34.1, 31.9, 31.9, 31.8, 31.8, 27.5, 27.2, 23.7.

**HRMS** (ESI) for C_26_H_39_NO_3_SNa: calculated 468.2548 [M+Na]^+^, found 468.2535.

[α]^20^_D_ = -2.3° (CHCl_3_, *c* = 1.0).

**((3a*S*,4*R*,6*R*,7a*S*)-5,5-dimethyl-2-((4-neopentylphenyl)sulfonyl)octahydro-3a*H*-4,6-methanoisoindol-3a-yl)methanol（S25）:**

Following general procedure D2: From **11** (58.6 mg, 0.3 mmol) and 4-neopentylbenzenesulfonyl chloride (74.3 mg, 1.0 eq.). Purification by column chromatography (PE/EtOAc 10:1 to 4:1, v/v) afforded a colorless solid **S25** (R*_f_*= 0.3, PE:EA=2:1, v/v; 79.2 mg, 0.2 mmol, 65 %).

**^1^H NMR** (500 MHz, CDCl_3_) δ 7.70 (d, *J* = 8.4 Hz, 2H), 7.28 (d, *J* = 8.3 Hz, 2H), 3.49 (d, *J* = 10.6 Hz, 1H), 3.42 (d, *J* = 10.5 Hz, 1H), 3.20 (dd, *J* = 9.1, 7.2 Hz, 1H), 3.06 (d, *J* = 9.7 Hz, 1H), 2.98 – 2.92 (m, 2H), 2.57 (s, 2H), 2.30 – 2.23 (m, 1H), 2.20 – 2.13 (m, 2H), 1.94 (dd, *J* = 6.5, 5.0 Hz, 1H), 1.85 (tt, *J* = 5.6, 3.0 Hz, 1H), 1.59 (ddd, *J* = 13.4, 4.3, 3.0 Hz, 1H), 1.26 (d, *J* = 10.4 Hz, 1H), 1.21 (s, 3H), 0.94 (s, 3H), 0.90 (s, 9H).

**^13^C NMR** (126 MHz, CDCl_3_) δ 145.5, 132.1, 130.9, 127.7, 68.2, 58.7, 56.9, 52.3, 50.2, 45.9, 40.4, 39.1, 34.2, 34.0, 32.1, 29.5, 27.5, 27.2, 23.8.

**HRMS** (ESI) for C_23_H_36_NO_3_S: calculated 406.2410 [M+H]^+^, found 406.2411.[α]^20^_D_ = -4.3° (CHCl_3_, *c* = 0.5).

**((3a*S*,4*R*,6*R*,7a*S*)-5,5-dimethyl-2-((4-(*tert*-pentyl)phenyl)sulfonyl)octahydro-3a*H*-4,6-methanoisoindol-3a-yl)methanol (S26):**

Following general procedure D2: From **11** (58.59 mg, 0.3 mmol) and 4-(*tert*-pentyl) benzenesulfonyl chloride (74.03 mg, 1.0 eq.). Purification by column chromatography (PE/EtOAc 4:1, v/v) afforded a colorless solid **S26** (R*_f_*= 0.3, PE:EA=2:1, v/v; 86.4 mg, 0.21 mmol, 71%).

**^1^H NMR** (500 MHz, CDCl_3_) δ 7.72 (d, *J* = 8.5 Hz, 2H), 7.48 (d, *J* = 8.6 Hz, 2H), 3.50 (d, *J* = 10.5 Hz, 1H), 3.42 (d, *J* = 10.6 Hz, 1H), 3.21 (dd, *J* = 9.1, 7.3 Hz, 1H), 3.07 (d, *J* = 9.7 Hz, 1H), 2.96 (d, *J* = 4.0 Hz, 1H), 2.94 (d, *J* = 3.5 Hz, 1H), 2.30 – 2.22 (m, 1H), 2.20 – 2.13 (m, 2H), 1.94 (dd, *J* = 6.5, 5.0 Hz, 1H), 1.85 (tt, *J* = 5.7, 3.0 Hz, 1H), 1.67 (q, *J* = 7.5 Hz, 2H), 1.60 (ddd, *J* = 13.4, 4.3, 2.9 Hz, 1H), 1.31 (s, 6H), 1.27 (d, *J* = 10.5 Hz, 1H), 1.21 (s, 3H), 0.94 (s, 3H), 0.63 (t, *J* = 7.5 Hz, 3H).

**^13^C NMR** (126 MHz, CDCl_3_) δ 155.1, 131.7, 128.0, 126.7, 68.2, 58.6, 56.9, 52.3, 45.9, 40.4, 39.1, 38.6, 37.0, 34.2, 34.0, 28.3, 27.5, 27.2, 23.7, 9.2.

**HRMS** (ESI) for C_23_H_35_NO_3_SNa: calculated 428.2235 [M+Na]^+^, found 428.2227.[α]^20^_D_ = -15.3° (CHCl_3_, *c* = 1.0).

**((3a*S*,4*R*,6*R*,7a*S*)-2-((4'-(tert-butyl)-[1,1'-biphenyl]-4-yl)sulfonyl)-5,5-dimethyloctahydro-3a*H*-4,6-methanoisoindol-3a-yl)methanol (S27):**

Following general procedure D2: From **11** (39.1 mg, 0.2 mmol) and 4'-(*tert*-butyl)-[1,1'-biphenyl]-4-sulfonyl chloride (61.8 mg, 0.2 mmol, 1.0 eq.). Purification by column chromatography (PE/EtOAc 10:1 to 4:1, v/v) afforded a colorless solid **S27** (R*_f_*= 0.3, PE:EA=2:1, v/v; 93.2 mg, 0.20 mmol, 66%).

**^1^H NMR** (500 MHz, CDCl_3_)δ 7.85 (d, *J* = 8.5 Hz, 2H), 7.75 (d, *J* = 8.5 Hz, 2H), 7.58 (d, *J* = 8.7 Hz, 2H), 7.51 (d, *J* = 8.7 Hz, 2H), 3.51 (d, *J* = 10.5 Hz, 1H), 3.43 (d, *J* = 10.5 Hz, 1H), 3.23 (dd, *J* = 9.1, 7.2 Hz, 1H), 3.10 (d, *J* = 9.7 Hz, 1H), 3.03 (d, *J* = 7.2 Hz, 1H), 3.03 – 2.99 (m, 1H), 2.32 – 2.24 (m, 1H), 2.21 – 2.14 (m, 2H), 1.96 (dd, *J* = 6.5, 5.0 Hz, 1H), 1.86 (tt, *J* = 5.6, 3.0 Hz, 1H), 1.62 (ddd, *J* = 13.4, 4.3, 2.8 Hz, 1H), 1.37 (s, 9H), 1.32 (d, *J* = 10.4 Hz, 1H), 1.22 (s, 3H), 0.95 (s, 3H).

**^13^C NMR** (126 MHz, CDCl_3_) δ 151.9, 145.5, 136.4, 133.2, 128.7, 127.4, 127.1, 126.2, 68.1, 58.6, 57.0, 52.3, 45.9, 40.4, 39.2, 34.8, 34.3, 34.1, 31.4, 27.5, 27.2, 23.8.

**HRMS** (ESI) for C_28_H_38_NO_3_S: calculated 468.2567 [M+H]^+^, found 468.2568.[α]^20^_D_ = -11.3° (CHCl_3_, *c* = 1.0).

**((3a*S*,4*R*,6*R*,7a*S*)-5,5-dimethyl-2-((4-(trimethylsilyl)phenyl)sulfonyl)octahydro-3a*H*-4,6-methanoisoindol-3a-yl)methanol (S28):**

Following general procedure D2: From **11** (58.59, 0.3 mmol) and 4-(trimethylsilyl)benzenesulfonyl chloride (74.6 mg, 0.3 mmol, 1.0 eq.). Purification by column chromatography (PE/EtOAc 10:1 to 4:1, v/v) afforded a colorless solid **S28** (R*_f_*= 0.4, PE:EA=2:1, v/v; 72.6 mg, 0.18 mmol, 60%).

**^1^H NMR** (500 MHz, Chloroform-d)δ 7.75 (d, *J* = 8.2 Hz, 2H), 7.67 (d, *J* = 8.2 Hz, 2H), 3.51 (d, *J* = 10.6 Hz, 1H), 3.41 (d, *J* = 10.6 Hz, 1H), 3.20 (dd, *J* = 9.2, 7.2 Hz, 1H), 3.07 (d, *J* = 9.7 Hz, 1H), 3.03 – 2.97 (m, 2H), 2.27 (tdd, *J* = 13.5, 3.2, 2.0 Hz, 1H), 2.22 – 2.11 (m, 2H), 1.96 (dd, *J* = 6.5, 5.0 Hz, 1H), 1.86 (tt, *J* = 5.7, 3.0 Hz, 1H), 1.65 – 1.58 (m, 1H), 1.31 (d, *J* = 10.4 Hz, 1H), 1.22 (s, 3H), 0.95 (s, 3H), 0.30 (s, 9H).

**^13^C NMR** (126 MHz, CDCl_3_) δ 147.1, 135.3, 133.9, 127.1, 68.1, 58.5, 56.9, 52.3, 45.9, 40.4, 39.2, 34.3, 34.1, 27.5 27.2, 23.7, -1.2.

**HRMS** (ESI) for C_21_H_34_NO_3_SSi: calculated 408.2023 [M+H]^+^, found 408.2023.[α]^20^_D_ = -10.7° (CHCl_3_, *c* = 0.5).

**((3a*S*,4*R*,6*R*,7a*S*)-2-((4-(adamantan-1-yl)phenyl)sulfonyl)-5,5-dimethyloctahydro-3a*H*-4,6-methanoisoindol-3a-yl)methanol (S29):**

Following general procedure D2: From **11** (39.6 mg, 0.2 mmol) and 4-(adamantan-1-yl)benzenesulfonyl chloride (62.2 mg, 1.0 eq.). Purification by column chromatography (PE/EtOAc 4:1, v/v) afforded a colorless solid **S29** (R*_f_*= 0.15, PE:EA=2:1, v/v; 74.2 mg, 0.16 mmol, 79%).

**^1^H NMR** (700 MHz, CDCl_3_) δ 7.73 (d, *J* = 8.2 Hz, 2H), 7.51 (d, *J* = 8.4 Hz, 2H), 3.51 (d, *J* = 10.5 Hz, 1H), 3.41 (d, *J* = 10.5 Hz, 1H), 3.23 – 3.18 (m, 1H), 3.06 (d, *J* = 9.7 Hz, 1H), 2.97 (dt, *J* = 8.7, 4.1 Hz, 2H), 2.30 – 2.23 (m, 1H), 2.19 – 2.14 (m, 2H), 2.14 – 2.10 (m, 3H), 1.96 (dd, *J* = 6.5, 5.0 Hz, 1H), 1.92 (d, *J* = 3.0 Hz, 6H), 1.87 – 1.83 (m, 1H), 1.84 – 1.78 (m, 3H), 1.78 – 1.73 (m, 3H), 1.60 (dt, *J* = 13.7, 2.5 Hz, 2H), 1.29 (d, *J* = 10.4 Hz, 1H), 1.21 (s, 3H), 0.94 (s, 3H).

**^13^C NMR** (176 MHz, CDCl_3_) δ 156.7, 132.0, 128.1, 125.6, 68.2, 58.5, 56.9, 52.3, 52.3, 45.9, 43.0, 40.4, 39.2, 36.9, 36.7, 34.2, 34.1, 28.9, 27.5, 27.2, 23.7.

**HRMS** (ESI) for C_28_H_39_NO_3_SNa: calculated 492.2548 [M+Na]^+^, found 492.2534.[α]^20^_D_ = -12.3° (CHCl_3_, *c* = 1).

**((3a*S*,4*R*,6*R*,7a*S*)-2-((4-(*tert*-butyl)-2-methylphenyl)sulfonyl)-5,5-dimethyloctahydro-3a*H*-4,6-methanoisoindol-3a-yl)methanol (S30)：**

Following general procedure D2: From **11** (58.59 mg, 0.3 mmol) and 4-(*tert*-butyl)-2-methylbenzenesulfonyl chloride (74.0 mg, 0.3 mmol, 1.0 eq.). Purification by column chromatography (PE/EtOAc 10:1 to 4:1, v/v) afforded a colorless solid **S30** (R*_f_*= 0.3, PE:EA=2:1, v/v; 80 mg, 0.20 mmol, 66%).

**^1^H NMR** (500 MHz, CDCl_3_) δ 7.83 (d, *J* = 8.1 Hz, 1H), 7.33 – 7.28 (m, 2H), 3.57 (d, *J* = 10.6 Hz, 1H), 3.50 (d, *J* = 10.6 Hz, 1H), 3.42 (dd, *J* = 9.3, 7.0 Hz, 1H), 3.25 (d, *J* = 9.8 Hz, 1H), 3.03 (dd, *J* = 9.4, 3.7 Hz, 1H), 2.99 (d, *J* = 9.7 Hz, 1H), 2.66 (s, 3H), 2.27 (ddt, *J* = 12.1, 7.6, 2.1 Hz, 1H), 2.23 – 2.16 (m, 2H), 2.00 – 1.94 (m, 1H), 1.87 (dt, *J* = 5.4, 2.7 Hz, 1H), 1.59 (d, *J* = 3.2 Hz, 1H), 1.33 (s, 9H), 1.31 (d, *J* = 10.5 Hz, 1H), 1.22 (s, 3H), 0.97 (s, 3H).

**^13^C NMR** (126 MHz, CDCl_3_) δ 156.6, 138.0, 132.6, 130.4, 129.9, 123.2, 68.3, 58.0, 56.3, 52.4, 45.8, 40.5, 39.2, 35.0, 34.3, 34.1, 31.2, 27.6, 27.3, 23.8, 21.3.

**HRMS** (ESI) for C_23_H_36_NO_3_S: calculated 406.2410 [M+H]^+^, found 406.2408.[α]^20^_D_ = -88.3° (CHCl_3_, *c* = 0.5).

**((3a*S*,4*R*,6*R*,7a*S*)-2-((4-(*tert*-butyl)-2-ethylphenyl)sulfonyl)-5,5-dimethyloctahydro-3a*H*-4,6-methanoisoindol-3a-yl)methanol (S31)：**

Following general procedure D2: From **11** (58.59 mg, 0.3 mmol) and 4-(*tert*-butyl)-2-ethylbenzenesulfonyl chloride (78.2 mg, 0.3 mmol, 1.0 eq.). Purification by column chromatography (PE/EtOAc 10:1 to 4:1, v/v) afforded a colorless solid **S31** (R*_f_*= 0.3, PE:EA=2:1, v/v; 109.4 mg. 0.26 mmol, 87%).

**^1^H NMR** (500 MHz, CDCl_3_) δ 7.82 (d, *J* = 8.4 Hz, 1H), 7.36 (d, *J* = 2.1 Hz, 1H), 7.30 (dd, *J* = 8.4, 2.1 Hz, 1H), 3.56 (d, *J* = 10.5 Hz, 1H), 3.50 (d, *J* = 10.7 Hz, 1H), 3.40 (dd, *J* = 9.3, 7.0 Hz, 1H), 3.25 (d, *J* = 9.8 Hz, 1H), 3.10 – 3.02 (m, 3H), 2.99 (d, *J* = 9.8 Hz, 1H), 2.30 – 2.23 (m, 1H), 2.23 – 2.13 (m, 2H), 1.99 – 1.95 (m, 1H), 1.89 – 1.84 (m, 1H), 1.61 – 1.56 (m, 1H), 1.33 (s, 9H), 1.30 (d, *J* = 10.4 Hz, 1H), 1.28 (t, *J* = 7.5 Hz, 3H), 1.22 (s, 3H), 0.96 (s, 3H).

**^13^C NMR** (126 MHz, CDCl_3_) δ 156.6, 144.3, 132.1, 130.3, 128.1, 123.1, 68.3, 58.0, 56.3, 52.4, 45.8, 40.5, 39.2, 35.1, 34.3, 34.0 31.2, 27.6, 27.2, 26.8, 23.8, 16.3.

**HRMS** (ESI) for C_24_H_37_NO_3_SNa: calculated 442.2392 [M+Na]^+^, found 442.2379.[α]^20^_D_ = -32.1° (CHCl_3_, *c* = 1).

**((3a*S*,4*R*,6*R*,7a*S*)-2-((4-(*tert*-butyl)-2-methoxyphenyl)sulfonyl)-5,5-dimethyloctahydro-3a**H**-4,6-methanoisoindol-3a-yl)methanol (S32):**

Following general procedure D2: From **11** (58.59mg, 0.2 mmol) and 4-(*tert*-butyl)-2-methoxybenzenesulfonyl chloride (92.0 mg, 0.2 mmol, 1.0 eq.). Purification by column chromatography (PE/EtOAc 2:1, v/v) afforded a colorless solid **S32** (R*_f_*= 0.1, PE:EA=2:1, v/v; 75.6 mg, 0.18 mmol, 90%).

**^1^H NMR** (500 MHz, CDCl_3_) δ 7.80 (d, *J* = 8.3 Hz, 1H), 7.04 (dd, *J* = 8.3, 1.7 Hz, 1H), 6.99 (d, *J* = 1.7 Hz, 1H), 3.92 (s, 3H), 3.76 – 3.71 (m, 1H), 3.52 (d, *J* = 5.6 Hz, 1H), 3.51 – 3.47 (m, 1H), 3.29 (d, *J* = 10.0 Hz, 1H), 3.16 – 3.11 (m, 2H), 2.30 – 2.16 (m, 3H), 1.95 (dd, *J* = 6.5, 4.9 Hz, 1H), 1.89 – 1.85 (m, 1H), 1.64 – 1.59 (m, 1H), 1.39 (d, *J* = 10.3 Hz, 1H), 1.33 (s, 9H), 1.22 (s, 3H), 0.96 (s, 3H).

**^13^C NMR** (126 MHz, CDCl_3_) δ 159.0, 157.1, 131.6, 123.2, 117.7, 109.8, 68.6, 58.4, 56.5, 56.1, 52.4, 45.9, 40.5, 39.2, 35.5, 34.3, 34.0, 31.2, 27.6, 27.2, 23.8.

**HRMS** (ESI) for C_23_H_35_NO_4_SNa: calculated 444.2184 [M+Na]^+^, found 444.2180.[α]^20^_D_ = -24.0° (CHCl_3_, *c* = 1.0).

**((3a*S*,4*R*,6*R*,7a*S*)-2-((2-bromo-4-(*tert*-butyl)phenyl)sulfonyl)-5,5-dimethyloctahydro-3a*H*-4,6-methanoisoindol-3a-yl)methanol (S33):**

Following general procedure D2: From **11** (29.3 mg, 0.15 mmol) and arenesulfonyl chloride (46.7 mg, 0.15 mmol, 1.0 eq.). Purification by column chromatography (PE/EtOAc 10:1 to 4:1, v/v) afforded a colorless solid **S33** (R*_f_*= 0.3, PE:EA=2:1, v/v; 70.6 mg, quant.).

**^1^H NMR** (500 MHz, CDCl_3_) δ 7.98 (d, *J* = 8.4 Hz, 1H), 7.71 (d, *J* = 1.9 Hz, 1H), 7.42 (dd, *J* = 8.3, 1.9 Hz, 1H), 3.60 (dd, *J* = 9.7, 7.1 Hz, 1H), 3.56 (d, *J* = 10.5 Hz, 1H), 3.52 (d, J = 10.7 Hz, 1H), 3.36 (d, *J* = 10.1 Hz, 1H), 3.19 (dd, *J* = 9.5, 3.5 Hz, 1H), 3.10 (d, *J* = 10.0 Hz, 1H), 2.30 – 2.15 (m, 3H), 1.97 (dd, *J* = 6.5, 5.0 Hz, 1H), 1.88 (dq, *J* = 5.5, 2.4 Hz, 1H), 1.68 – 1.61 (m, 1H), 1.35 (d, *J* = 10.5 Hz, 6H), 1.32 (s, 9H), 1.22 (s, 3H), 0.97 (s, 3H).

**^13^C NMR** (126 MHz, CDCl_3_) δ 158.0, 134.6, 133.0, 132.2, 124.7, 120.7, 68.1, 58.6, 56.4, 52.8, 45.7, 40.5, 39.1, 35.2, 34.5, 33.9, 31.0, 27.5, 27.3, 23.8.

**HRMS** (ESI) for C_22_H_32_BrNO_3_SNa: calculated 492.1184 [M+Na]^+^, found 492.1167.

[α]^20^_D_ = -12.1° (CHCl_3_, *c* = 1.0).

**((3a*S*,4*R*,6*R*,7a*S*)-2-((4-(tert-butyl)-2-iodophenyl)sulfonyl)-5,5-dimethyloctahydro-3a*H*-4,6-methanoisoindol-3a-yl)methanol (S34):**

Following general procedure D2: From **11** (29.3 mg, 0.15 mmol) and 4-(*tert*-butyl)-2-iodobenzenesulfonyl chloride (46.7 mg, 0.15 mmol, 1.0 eq.). Purification by column chromatography (PE/EtOAc 10: 1 to 4:1, v/v) afforded a colorless solid **S34** (R*_f_*= 0.25, PE:EA=2:1, v/v; 70.6 mg, 0.15 mmol, quant.).

**^1^H NMR** (500 MHz, CDCl_3_) δ 8.06 (d, *J* = 1.9 Hz, 1H), 8.00 (d, *J* = 8.3 Hz, 1H), 7.46 (dd, *J* = 8.4, 1.9 Hz, 1H), δ 3.79 – 3.70 (m, 1H).3.65 – 3.59 (m, 1H), 3.59 (d, *J* = 10.7 Hz, 1H), 3.54 (d, *J* = 10.6 Hz, 1H), 3.37 (d, *J* = 10.1 Hz, 1H), 3.19 (dd, *J* = 9.7, 3.6 Hz, 1H), 3.07 (d, *J* = 10.0 Hz, 1H), 2.30 – 2.17 (m, 3H), 1.98 (dd, *J* = 6.5, 5.0 Hz, 1H), 1.89 (dt, *J* = 5.6, 2.8 Hz, 1H), 1.69 (dd, *J* = 9.2, 2.9 Hz, 1H), 1.38 (d, *J* = 10.4 Hz, 1H), 1.32 (s, 9H), 1.22 (s, 3H), 0.97 (s, 3H).

**^13^C NMR** (126 MHz, CDCl_3_) δ 157.6, 140.5, 137.7, 131.6, 125.5, 93.1, 68.2, 58.6, 56.5, 52.8, 45.7, 40.5, 39.1, 35.0, 34.4, 33.7, 31.0, 27.6, 27.4, 23.8.

**HRMS** (ESI) for C_22_H_32_INO_3_SNa: calculated 540.1045 [M+Na]^+^, found 540.1034.

[α]^20^_D_ = -9.0° (CHCl_3_, *c* = 1.0).

**((3a*S*,4*R*,6*R*,7a*S*)-2-((4-(1-(2-methoxyethoxy)-2-methylpropan-2-yl)phenyl)sulfonyl)-5,5-dimethyloctahydro-3a*H*-4,6-methanoisoindol-3a-yl)methanol (S35):**

Following general procedure D2: From **11** (48.83 mg, 0.25 mmol) and arenesulfonyl chloride (1.0 eq.). Purification by column chromatography (PE/EtOAc 4:1, v/v) afforded a colorless solid **S35** (R*_f_*= 0.3, PE:EA=1:1, v/v; 104 mg, 0.225 mmol, 90 %).

**^1^H NMR** (700 MHz, CDCl_3_) δ 7.73 (d, J = 8.8 Hz, 2H), 7.55 (d, J = 8.8 Hz, 2H), 3.52 (d, J = 9.3 Hz, 1H), 3.48 (d, J = 9.4 Hz, 1H), 3.47 – 3.43 (m, 4H), 3.39 (d, J = 2.9 Hz, 2H), 3.32 (s, 3H), 3.14 (dd, J = 9.1, 7.1 Hz, 1H), 3.06 – 3.02 (m, 1H), 3.02 (d, J = 9.9 Hz, 1H), 2.97 (d, J = 9.7 Hz, 1H), 2.31 – 2.24 (m, 1H), 2.19 (dtd, J = 14.9, 8.0, 7.6, 5.1 Hz, 2H), 1.91 (t, J = 5.8 Hz, 1H), 1.86 (dt, J = 5.2, 2.6 Hz, 1H), 1.64 – 1.60 (m, 1H), 1.38 (s, 1H), 1.36 (s, 6H), 1.21 (s, 3H), 0.94 (s, 3H).

**^13^C NMR** (176 MHz, CDCl_3_) δ 153.2, 132.1, 128.0, 126.9, 81.1, 72.0, 71.1, 68.1, 59.2, 58.7, 56.9, 52.4, 46.3, 40.4, 39.8, 39.2, 34.4, 34.4, 27.6, 27.4, 26.0, 26.0, 23.8.

**HRMS** (ESI) for C_25_H_39_NO_5_SNa: calculated: 488.2447 [M+Na]^+^, found 488.2445.

[α]^20^_D_ = +12.0° (CHCl_3_, *c* = 0.5).

**2-(4-(((3a*S*,4*R*,6*R*,7a*S*)-3a-(hydroxymethyl)-5,5-dimethyloctahydro-2*H*-4,6-methanoisoindol-2-yl)sulfonyl)phenyl)-2-methyl-1-morpholinopropan-1-one (S36):**

Following general procedure D2: From **11** (58.6 mg, 0.3 mmol) and arenesulfonyl chloride (1.0 eq.). Purification by column chromatography (PE/EtOAc 2:1, v/v) afforded a colorless solid **S36** (R*_f_*= 0.3, PE:EA=1:1, v/v; 104.8 mg, 0.21 mmol, 71%).

**^1^H NMR** (400 MHz, CDCl_3_) δ 7.79 (d, *J* = 8.5 Hz, 2H), 7.41 (d, *J* = 8.5 Hz, 2H), 3.60 (s, 2H), 3.47 – 3.39 (m, 3H), 3.25 (d, *J* = 6.3 Hz, 3H), 3.14 (dd, *J* = 9.0, 7.0 Hz, 2H), 3.05 – 2.95 (m, 4H), 2.34 – 2.24 (m, 1H), 2.18 (tdd, *J* = 8.4, 6.6, 3.9 Hz, 2H), 1.93 (dd, *J* = 6.5, 5.0 Hz, 1H), 1.86 (tt, *J* = 7.0, 3.1 Hz, 1H), 1.64 – 1.59 (m, 1H), 1.56 (s, 6H), 1.30 (d, *J* = 10.4 Hz, 1H), 1.21 (s, 3H), 0.94 (s, 3H).

**^13^C NMR** (101 MHz, CDCl_3_) δ 174.1, 151.5, 133.3, 129.0, 125.6, 68.0, 58.7, 56.8, 52.6, 47.3, 46.2, 40.4, 39.2, 34.3, 34.3, 28.3, 28.2, 27.5, 27.2, 23.7.

**HRMS** (ESI) for C_26_H_38_N_2_O_5_SNa: calculated: 513.2399 [M+Na]^+^, found 513.2388.

[α]^20^_D_ = -3.1° (CHCl_3_, *c* = 1.0).

**2-(4-(((3a*S*,4*R*,6*R*,7a*S*)-3a-(hydroxymethyl)-5,5-dimethyloctahydro-2*H*-4,6-methanoisoindol-2-yl)sulfonyl)phenyl)-*N,N,2*-trimethylpropanamide (S37):**

Following general procedure D2: From **11** (39.6 mg, 0.2 mmol) and arenesulfonyl chloride (57.8 mg, 1.0 eq.). Purification by column chromatography (PE/EtOAc 2:1, v/v) afforded a colorless solid **S37** (R*_f_*= 0.2, PE:EA=1:1, v/v; 73 mg, 0.16 mmol, 81%).

**^1^H NMR** (600 MHz, CDCl_3_) δ 7.78 (d, *J* = 8.5 Hz, 2H), 7.39 (d, *J* = 8.5 Hz, 2H), 3.48 (d, *J* = 10.5 Hz, 1H), 3.43 (d, *J* = 10.4 Hz, 1H), 3.20 (dd, *J* = 9.1, 7.3 Hz, 1H), 3.08 (d, *J* = 9.7 Hz, 1H), 3.01 – 2.96 (m, 1H), 2.97 (d, *J* = 9.6 Hz, 1H), 2.94 (s, 3H), 2.43 (s, 3H), 2.31 – 2.24 (m, 1H), 2.22 – 2.13 (m, 2H), 1.94 (dd, *J* = 6.4, 5.0 Hz, 1H), 1.86 (tt, *J* = 5.6, 3.0 Hz, 1H), 1.60 (ddd, *J* = 13.5, 4.4, 2.9 Hz, 1H), 1.57 (s, 6H), 1.26 (d, *J* = 10.4 Hz, 1H), 1.21 (s, 3H), 0.95 (s, 3H).

**^13^C NMR** (151 MHz, CDCl_3_)δ 175.2, 152.0, 133.0, 129.0, 125.5, 68.1, 58.7, 56.9, 52.3, 47.4, 46.1, 40.4, 39.2, 38.2, 37.4, 34.3, 34.1, 28.2, 27.5, 27.2, 23.7.

**HRMS** (ESI) for C_24_H_36_N_2_O_4_SNa: calculated: 471.2293 [M+Na]^+^, found 471.2269.

[α]^20^_D_ = -12.7° (CHCl_3_, *c* = 1.0).

**Methyl 2-(4-(((3aS,4R,6R,7aS)-3a-(hydroxymethyl)-5,5-dimethyloctahydro-2*H*-4,6-methanoisoindol-2-yl)sulfonyl)phenyl)-2-methylpropanoate (S38):**

Following general procedure D2: From **11** (78.1 mg, 0.4 mmol) and arenesulfonyl chloride (110.7 mg, 0.4 mmol, 1.0 eq.). Purification by column chromatography (PE/EtOAc 4:1, v/v) afforded a colorless solid **S38** (R*_f_*= 0.15, PE:EA=2:1, v/v; 129 mg, 0.3 mmol, 75%).

**^1^H NMR** (500 MHz, CDCl_3_) δ 7.75 (d, *J* = 8.5 Hz, 2H), 7.49 (d, *J* = 8.5 Hz, 2H), 3.66 (s, 3H), 3.49 (d, *J* = 10.5 Hz, 1H), 3.41 (d, *J* = 10.6 Hz, 1H), 3.20 (dd, *J* = 9.1, 7.2 Hz, 1H), 3.07 (d, *J* = 9.7 Hz, 1H), 2.99 (dd, *J* = 9.4, 2.8 Hz, 2H), 2.30 – 2.24 (m, 1H), 2.20 – 2.14 (m, 2H), 1.95 (dd, *J* = 6.5, 5.0 Hz, 1H), 1.87 – 1.84 (m, 1H), 1.60 (s, 6H), 1.29 (d, *J* = 10.4 Hz, 1H), 1.21 (s, 3H), 0.94 (s, 3H).

**^13^C NMR** (126 MHz, CDCl_3_) δ 176.5, 149.8, 133.6, 128.3 126.5, 68.1, 58.5, 56.9, 52.6, 52.4, 47.0, 45.9, 40.4, 39.2, 34.3, 34.1, 27.5, 27.2, 26.5, 23.7.

**HRMS** (ESI) for C_23_H_33_NO_5_SNa: calculated: 458.1977 [M+Na]^+^, found 458.1971.

[α]^20^_D_ = -16.0° (CHCl_3_, *c* = 1.0).

**Methyl 2-(4-(((3a*S*,4*R*,6*R*,7a*S*)-3a-(hydroxymethyl)-5,5-dimethyloctahydro-2*H*-4,6-methanoisoindol-2-yl)sulfonyl)-3-methoxyphenyl)-2-methylpropanoate (S39)**:

Following general procedure D2: From **11** (58.6 mg, 0.3 mmol) and arenesulfonyl chloride (92 mg, 1.0 eq.). Purification by column chromatography (PE/EtOAc 10:1 to 4:1, v/v) afforded a colorless solid **S39** (R*_f_*= 0.15, PE:EA=1:1, v/v; 122.7 mg, 0.26 mmol, 88%).

**^1^H NMR** (600 MHz, CDCl_3_)δ 7.84 (d, *J* = 8.3 Hz, 1H), 6.99 (dd, *J* = 8.3, 1.7 Hz, 1H), 6.94 (d, *J* = 1.8 Hz, 1H), 3.91 (s, 3H), 3.67 (s, 3H), 3.53 (d, *J* = 10.7 Hz, 1H), 3.52 – 3.47 (m, 2H), 3.30 (d, *J* = 10.1 Hz, 1H), 3.14 (d, *J* = 9.9 Hz, 2H), 2.29 – 2.16 (m, 3H), 1.95 (dd, *J* = 6.4, 4.9 Hz, 1H), 1.87 (tt, *J* = 5.5, 2.9 Hz, 1H), 1.64 – 1.59 (m, 1H), 1.58 (s, 6H), 1.37 (d, *J* = 10.4 Hz, 1H), 1.22 (s, 3H), 0.96 (s, 3H).

**^13^C NMR** (151 MHz, CDCl_3_) δ 176.5, 157.2, 151.9, 131.9, 124.9, 117.9, 110.2, 68.5, 58.4, 56.5, 56.1, 52.6, 52. 5, 47.1, 46.0, 40.5, 39.2, 34.4, 34.1, 27.6, 27.2, 26.5, 26.5, 23.8.

**HRMS** (ESI) for C_24_H_35_NO_6_SNa: calculated: 488.2083 [M+Na]^+^, found 488.2068.

[α]^20^_D_ = +10.1° (CHCl_3_, *c* = 0.2).

**Methyl 2-(3-chloro-4-(((3aS,4R,6R)-3a-(hydroxymethyl)-5,5-dimethyloctahydro-2*H*-4,6-methanoisoindol-2-yl)sulfonyl)phenyl)-2-methylpropanoate (S40):**

Following general procedure D2: From **11** (25.4 mg, 0.13 mmol) and arenesulfonyl chloride (39 mg, 1.0 eq.). Purification by column chromatography (PE/EtOAc 10:1 to 4:1, v/v) afforded a colorless solid **S40** (R*_f_*= 0.25, PE:EA=2:1, v/v; 47.5 mg, 0.1 mmol, 78%).

**^1^H NMR** (500 MHz, CDCl_3_) δ 7.99 (d, *J* = 8.3 Hz, 1H), 7.47 (d, *J* = 1.9 Hz, 1H), 7.33 (dd, *J* = 8.3, 1.9 Hz, 1H), 3.68 (s, 3H), 3.60 (dd, *J* = 9.7, 7.1 Hz, 1H), 3.56 (d, *J* = 10.6 Hz, 1H), 3.50 (d, *J* = 10.6 Hz, 1H), 3.36 (d, *J* = 10.1 Hz, 1H), 3.21 (dd, *J* = 9.7, 3.6 Hz, 1H), 3.13 (d, *J* = 10.1 Hz, 1H), 2.27 (dd, *J* = 13.5, 1.6 Hz, 2H), 2.26 – 2.18 (m, 2H), 1.97 (dd, *J* = 6.5, 5.0 Hz, 1H), 1.89 (dq, *J* = 5.4, 2.6 Hz, 1H), 1.63 (s, 2H), 1.59 (s, 6H), 1.33 (d, *J* = 10.5 Hz, 1H), 1.22 (s, 3H), 0.97 (s, 3H).

**^13^C NMR** (126 MHz, CDCl_3_) δ 176.0, 151.1, 134.5, 132.8, 132.1, 129.8, 124.7, 68.1, 58.6, 56.4, 52.8, 46.8, 45.8, 40.5, 39.2, 34.6, 34.0, 27.6, 27.3, 26.4, 26.4, 23.8.

**HRMS** (ESI) for C_23_H_32_ClNO_5_SNa: calculated: 492.1587 [M+Na]^+^, found 492.1580.

[α]^20^_D_ = -12.0° (CHCl_3_, *c* = 1.0).

**Methyl 2-(4-(((3aS,4R,6R,7aS)-3a-(hydroxymethyl)-5,5-dimethyloctahydro-2*H*-4,6-methanoisoindol-2-yl) sulfonyl)-3-methylphenyl)-2-methylpropanoate (S41):**

Following general procedure D2: From **11** (117.2 mg, 0.6 mmol) and arenesulfonyl chloride (174.58 mg, 0.6 mmol, 1.0 eq.). Purification by column chromatography (PE/EtOAc 10:1 to 4:1, v/v) afforded a colorless solid **S41** (R*_f_*= 0.25, PE:EA=2:1, v/v; 246.5 mg, 0.55 mmol, 91 %).

**^1^H NMR** (500 MHz, DMSO-d6) δ 7.83 (d, *J* = 8.9 Hz, 1H), 7.33 – 7.23 (m, 2H), 3.65 (s, 3H), 3.52 (d, *J* = 10.3 Hz, 1H), 3.46 (dd, *J* = 10.6, 3.7 Hz, 1H), 3.37 (dd, *J* = 9.3, 7.1 Hz, 1H), 3.21 (d, *J* = 9.7 Hz, 1H), 3.02 (dd, *J* = 9.2, 3.5 Hz, 1H), 2.96 (d, *J* = 9.7 Hz, 1H), 2.64 (s, 3H), 2.32 – 2.25 (m, 1H), 2.25 – 2.15 (m, 2H), 1.95 (dd, *J* = 6.5, 5.0 Hz, 1H), 1.86 (tt, *J* = 5.7, 3.0 Hz, 1H), 1.57 (d, *J* = 2.3 Hz, 9H), 1.56 – 1.54 (m, 1H), 1.29 (s, 1H), 1.22 (s, 3H), 0.96 (s, 3H).

**^13^C NMR** (126 MHz, DMSO) δ 176.7, 150.2, 138.7, 134.3, 130.6, 130.6, 123.9, 68.3, 58.3, 56.5, 52.7, 52.6, 47.0, 46.3, 40.8, 39.4, 34.7, 34.4, 27.6, 27.5, 26.5, 23.7, 21.3.

**HRMS** (ESI) for C_24_H_35_NO_5_SNa: calculated: 472.2134 [M+Na]^+^, found 472.2124.

[α]^20^_D_ = -28.1° (CHCl_3_, *c* = 0.5).

## **General procedure E: Carbamate formation**

**Procedure E1**

To a solution of the alcohol (1.0 eq.) in anhydrous THF (0.2 m) was added NEt_3_ (2.0 eq.) and the respective isocyanate (1.3 eq.). The solution was stirred at room temperature for 2 h. The solvent was removed under reduced pressure. Column chromatography afforded the carbamate.

**Procedure E2**

To a solution of the alcohol (1.0 eq.) in anhydrous THF (0.1−0.2 m) was added DBU (1.5 eq.) and the respective isocyanate (1.2 eq.) at 0 °C. The solution was stirred at room temperature for 18‑22 h. The solvent was removed under reduced pressure. Column chromatography afforded the carbamate.

**Procedure E3**

To a solution of the alcohol (1.0 eq.) in anhydrous DCM (0.2 m) was added NEt_3_ (3.0 eq.) and the respective isocyanate (1.3 eq.). The solution was stirred at room temperature for 24 h and then water was added. The mixture was then extracted with DCM (3 times) and the combined organic phases was dried over anhydrous MgSO_4_, filtered and concentrated under reduced pressure. Column chromatography afforded the carbamate.

**((3a*S*,4*R*,6*R*,7a*S*)-2-benzyl-5,5-dimethyloctahydro-3a*H*-4,6-methanoisoindol-3a-yl)methyl (3-nitrophenyl)carbamate (S42):**

Following general procedure E1: From **8** (72.0 mg, 0.252 mmol) and 3-nitrophenyl isocyanate. Purification by column chromatography (PE/EtOAc 9:1, v/v) afforded a yellow oil **S42** (R*_f_*= 0.4, PE:EA=3:1, v/v; 98.0 mg, 0.218 mmol, 86%).

**^1^H NMR** (CDCl_3_, 500 MHz): δ = 8.22–8.34 (m, 1 H, Ar-H), 7.88–7.92 (m, 1 H, Ar-H), 7.71–7.81 (m, 1 H, Ar-H), 7.46 (t, *J* = 8.2 Hz, 1 H, Ar-H), 7.19–7.40 (m, 5 H, 5 × Ar-H), 6.85–6.96 (m, 1 H, NH), 4.19–4.29 (m, 1 H, CH), 4.06–4.15 (m, 1 H, CH), 3.52–3.67 (m, 2 H, 2 × CH), 2.60–2.71 (m, 1 H, CH), 2.46–2.59 (m, 2 H, 2 × CH), 2.32–2.42 (m, 1 H, CH), 2.22–2.29 (m, 1 H, CH), 2.06–2.21 (m, 2 H, 2 × CH), 1.91–1.97 (m, 1 H, CH), 1.78–1.90 (m, 2 H, 2 × CH), 1.62 (dt, *J* = 3.5, 13.3 Hz, 1 H, CH), 1.03 (s, 3 H, CH_3_), 1.24 (s, 3 H, CH_3_).

**^13^C NMR** (CDCl_3_, 125 MHz):δ = 153.5, 148.8, 139.6, 130.0, 128.6, 128.4, 127.0, 124.2, 120.7, 118.0, 113.4, 71.6, 64.4, 64.0, 60.6, 49.9, 46.2, 40.5, 39.4, 34.8, 34.3, 27.6, 27.1, 26.7, 23.6.

**HRMS** (ESI) for C_26_H_32_N_3_O_4_: calculated 450.2387 [M+H]^+^, found 450.2386.

[α]^20^_D_ = -18.1° (CHCl_3_, *c* = 0.5).

**((3a*S*,4*R*,6*R*,7a*S*)-2-benzyl-5,5-dimethyloctahydro-3a*H*-4,6-methanoisoindol-3a-yl) methyl (4-methoxyphenyl) carbamate (S43):**

Following general procedure E1: From **8** (285 mg, 1.00 mmol) and 4-methoxyphenyl isocyanate. Purification by column chromatography (PE/EtOAc= 8:1, v/v) afforded a slightly yellow semicrystalline solid **S43** (R*_f_*= 0.6, PE:EA=3:1, v/v; 314 mg, 0.723 mmol, 72%).

**^1^H NMR** (CDCl_3_, 500 MHz): δ = 7.20–7.39 (m, 7 H, 7 × Ar‑H), 6.82–6.88 (m, 2 H, 2 × Ar‑H), 6.44 (s_br_, 1 H, NH), 4.19 (d, *J* = 10.3 Hz, 1 H, CH_B’_), 4.04 (d, *J* = 10.4 Hz, 1 H, CH_A’_), 3.78 (s, 3 H, OMe), 3.63 (d, *J* = 13.4 Hz, 1 H, CH_B_), 3.55 (d, *J* = 13.1 Hz, 1 H, CH_A_), 2.45–2.64 (m, 3 H, 3 × CH), 2.33 (d, *J* = 9.4 Hz, 1 H, CH), 2.21–2.28 (m, 1 H, CH), 2.06–2.17 (m, 2 H, 2 × CH), 1.91–1.98 (m, 1 H, CH), 1.83–1.90 (m, 2 H, 2 × CH), 1.60 (dt, *J* = 3.5, 13.3 Hz, 1 H, CH), 1.23 (s, 3 H, CH_3_), 1.03 (s, 3 H, CH_3_),

**^13^C NMR** (CDCl_3_, 125 MHz):δ = 153.9, 139.9, 128.6, 128.3, 126.8, 120.6, 114.3, 64.2 (2C), 60.0, 55.6, 50.0, 46.3, 40.6, 39.4, 34.8, 34.6, 29.8, 27.6, 27.1, 26.7, 23.6.

**HRMS** (ESI) for C_27_H_35_N_2_O_3_: calculated 435.2642 [M+H]^+^, found 435.2640.

[α]^20^_D_ = -13.5° (CHCl_3_, *c* = 1.0).

**((3a*S*,4*R*,6*R*,7a*S*)-2-benzyl-5,5-dimethyloctahydro-3a*H*-4,6-methanoisoindol-3a-yl) methyl (2-nitrophenyl) carbamate (S44):**

Following general procedure E1: From **8** (75.0 mg, 0.263 mmol) and 2-nitrophenyl isocyanate. Purification by column chromatography (PE/EtOAc 9:1, v/v) afforded a yellow oil **S44** (R*_f_*= 0.6, PE:EA=3:1, v/v; 98.0 mg, 0.218 mmol, 83%).

**^1^H NMR** (CDCl_3_, 500 MHz): δ = 9.81 (s, 1 H, NH), 8.57 (dd, *J* = 1.3, 8.6 Hz, 1 H, Ar-H), 8.21 (dd, *J* = 1.6, 8.5 Hz, 1 H, Ar-H), 7.64 (ddd, *J* = 1.6, 7.2, 8.8 Hz, 1 H, Ar-H), 7.37–7.33 (m, 2 H, 2 × Ar‑H), 7.32–7.27 (m, 2 H, 2 × Ar‑H), 7.24–7.20 (m, 1 H, Ar-H), 7.13 (ddd, *J* = 1.3, 7.2, 8.5 Hz, 1 H, Ar-H), 4.26 (d, *J* = 10.4 Hz, 1 H, CH_B’_), 4.13 (d, *J* = 10.4 Hz, 1 H, CH_A’_), 3.64 (d, *J* = 13.2 Hz, 1 H, CH_B_), 3.57 (d, *J* = 13.2 Hz, 1 H, CH_A_), 2.63 (dd, *J* = 6.6, 8.8 Hz, 1 H, CH), 2.59 (d, *J* = 9.5 Hz, 1 H, CH), 2.49 (dd, *J* = 3.0, 8.9 Hz, 1 H, CH), 2.37 (d, *J* = 9.5 Hz, 1 H, CH), 2.30–2.22 (m, 1 H, CH), 2.20–2.15 (m, 1 H, CH), 2.14–2.08 (m, 1 H, CH), 1.99–1.96 (m, 1 H, CH), 1.90–1.83 (m, 2 H, 2 × CH), 1.65–1.58 (m, 1 H, CH), 1.24 (s, 3 H, CH_3_),1.04 (s, 3 H, CH_3_),

**^13^C NMR** (CDCl_3_, 125 MHz):δ = 153.5, 139.8, 136.1 (2C), 135.7, 128.6, 128.3, 126.8, 126.0, 122.4, 120.9, 71.7, 64.3, 64.0, 60.0, 50.0, 46.2, 40.5, 39.4, 34.9, 34.4, 27.6, 27.1, 26.8, 23.6.

**HRMS** (ESI) for C_26_H_32_N_3_O_4_: calculated 450.2387 [M+H]^+^, found 450.2386.

[α]^20^_D_ = -15.3° (CHCl_3_, *c* = 0.1).

**((3a*S*,4*R*,6*R*,7a*S*)-2-((4-(tert-butyl) phenyl) sulfonyl)-5,5-dimethyloctahydro-3a*H*-4,6-methanoisoindol-3a-yl) methyl (2-aminophenyl) carbamate (S45):**

Following general procedure C2: From **S44** (100 mg, 0.222 mmol) afforded an orange oil which was used in next step without further purification (50.0 mg, 0.152 mmol, 68%).

Following general procedure D1: From the crude amine obtained from above step (50.0 mg, 0.152 mmol) and 4-*tert*-butylbenzenesulfonyl chloride. Purification by column chromatography (PE/EtOAc = 3:1, v/v) afforded a colorless oil **S45** (R*_f_*= 0.1, PE:EA=1:1, v/v; 41.0 mg, 78.0 µmol, 51%).

**^1^H NMR** (CDCl_3_, 500 MHz): δ 7.68 (d, *J* = 8.1 Hz, 2H), 7.48 (d, *J* = 8.6 Hz, 2H), 7.17 (m, 1H), 6.98 (t, *J* = 7.7 Hz, 1H), 6.78 (m, 2H), 6.25 (s, 1H), 4.00 (d, *J* = 8.7 Hz, 2H), 3.17 (s, 1H), 3.09 – 2.88 (m, 3H), 2.22 (m, 2H), 2.14 – 2.06 (m, 1H), 1.86 (s_br_, 1H), 1.81 (s_br_, 1H), 1.58 – 1.50 (m, 1H), 1.23 (s, 9H), 1.21 (d, *J* = 4.9 Hz, 1H), 1.14 (s, 3H), 0.93 (s, 3H).

**^13^C NMR** (CDCl_3_, 125 MHz):δ 156.8, 145.3, 132.3, 128.1, 126.1, 124.9, 120.8, 115.6, 70.5, 58.3, 56.9, 50.8, 46.6, 40.2, 39.1, 35.3, 34.5, 34.1, 31.2, 27.4, 27.1, 23.5.

**HRMS** (ESI) for C_29_H_40_N_3_O_4_S: calculated 526.2734 [M+H]^+^, found 526.2733.

[α]^20^_D_ = -16.5° (CHCl_3_, *c* = 1.0).

**((3a*S*,4*R*,6*R*,7a*S*)-2-benzyl-5,5-dimethyloctahydro-3a*H*-4,6-methanoisoindol-3a-yl) methyl *p*-tolylcarbamate (9):**

Following general procedure E1: From **8** (100 mg, 0.350 mmol) and *p*-tolyl isocyanate. Purification by column chromatography (PE/EtOAc 9:1, v/v) afforded a colorless foam **9** (R*_f_*= 0.6, PE:EA=3:1, v/v;122 mg, 0.291 mmol, 83 %)^[17]^.

**^1^H NMR** (CDCl_3_, 500 MHz): δ = 7.20–7.39 (m, 7 H, 7 × Ar‑H), 7.10 (d, *J* = 8.1 Hz, 2 H, 2 × Ar‑H), 6.46 (s_br_, 1 H, NH), 4.21 (d, *J* = 10.3 Hz, 1 H, CH_B’_), 4.05 (d, *J* = 10.3 Hz, 1 H, CH_A’_), 3.63 (d, *J* = 13.3 Hz, 1 H, CH_B_), 3.55 (d, *J* = 13.1 Hz, 1 H, CH_A_), 2.62–2.57 (m, 1 H, CH), 2.55 (d, *J* = 9.5 Hz, 1 H, CH), 2.52–2.46 (m, 1 H, CH), 2.34 (d, *J* = 9.4 Hz, 1 H, CH), 2.30 (s, 3 H, CH_3_), 2.28–2.21 (m, 1 H, CH), 2.19–2.06 (m, 2 H, 2 × CH), 1.95 (t, *J* = 5.7 Hz, 1 H, CH), 1.89–1.81 (m, 2 H, 2 × CH), 1.60 (dt, *J* = 3.5, 13.3 Hz, 1 H, CH), 1.23 (s, 3 H, CH_3_), 1.03 (s, 3 H, CH_3_).

**^13^C NMR** (CDCl_3_, 125 MHz):δ = 154.0, 139.9, 135.5, 133.01, 129.7, 128.6, 128.3, 126.8, 118.7, 71.0, 64.2, 50.5, 46.3, 40.6, 39.5, 34.9, 34.6, 29.9, 27.6, 27.1, 26.7, 23.6, 20.9.

**HRMS** (ESI) for C_27_H_35_N_2_O_2_: calculated 419.2693 [M+H]^+^, found 419.2692.

**((3a*S*,4*R*,6*R*,7a*S*)-2-((4-(*tert*-butyl)phenyl)sulfonyl)-5,5-dimethyloctahydro-3a*H*-4,6-methanoisoindol-3a-yl)methyl *p*-tolylcarbamate (7) (iDeg-1):**

Following general procedure C1: From **9** (1.0 g, 2.39 mmol) afforded a colorless solid amine without further purification(745.5 mg, 2.27 mmol, 95%).

Following general procedure D1: From the crude amine (81.0 mg, 0.247 mmol) and 4-*tert*-butylbenzenesulfonyl chloride. Purification by column chromatography (PE/EtOAc 8:1 → 4:1, v/v) afforded a colorless foam **1 (iDeg-1)**^[17]^ (R*_f_*= 0.8, PE:EA=3:1, v/v; 115 mg, 0.219 mmol, 89%).

**^1^H NMR** (600 MHz, CD_2_Cl_2_) δ 7.71 (d, *J* = 8.5 Hz, 2H), 7.56 (d, *J* = 8.6 Hz, 2H), 7.19 (d, *J* = 7.6 Hz, 2H), 7.10 (d, *J* = 7.9 Hz, 2H), 6.36 (s, 1H), 4.06 (d, *J* = 10.7 Hz, 1H), 3.92 (d, *J* = 10.6 Hz, 1H), 3.15 (dd, *J* = 9.2, 7.1 Hz, 1H), 3.05 (d, *J* = 9.9 Hz, 1H), 3.02 (dd, *J* = 9.2, 3.1 Hz, 1H), 2.99 (d, *J* = 9.9 Hz, 1H), 2.33 (ddd, *J* = 10.5, 3.3, 2.1 Hz, 1H), 2.30 (s, 3H), 2.25 (tdd, *J* = 7.4, 4.2, 2.2 Hz, 1H), 2.20 (ddt, *J* = 8.4, 6.2, 3.1 Hz, 1H), 1.98 (dd, *J* = 6.5, 5.0 Hz, 1H), 1.87 (tt, *J* = 5.6, 3.0 Hz, 1H), 1.63 – 1.59 (m, 1H), 1.32 (d, *J* = 10.4 Hz, 1H), 1.30 (s, 9H), 1.22 (s, 3H), 1.01 (s, 3H).

**^13^C** **NMR** (151 MHz, CD_2_Cl_2_) δ 157.2, 153.7, 135.6, 133.6, 132.4, 129.9, 128.3, 126.4, 119.2, 70.1, 58.5, 57.4, 54.2, 54.0, 53.8, 53.7, 53.5, 50.9, 46.7, 40.6, 39.4, 35.4, 34.7, 34.5, 31.2, 27.4, 27.3, 23.5, 20.8.

**HRMS** (ESI) for C_30_H_41_N_2_O_4_S: calculated 525.2782 [M+H]^+^, found 525.2785.

[α]^20^_D_ = -16.0° (CHCl_3_, *c* = 0.5).

**((3aR,4S,6S,7aR)-2-((4-(tert-butyl)phenyl)sulfonyl)-5,5-dimethyloctahydro-3aH-4,6-methanoisoindol-3a-yl)methyl (4-iodophenyl)carbamate (20):**

Following general procedure E2: From **S3** (45.9 mg, 0.12 mmol 1 eq) and 4-Iodophenyl isocyanate (0.144 mmol, 35.3 mg, 1.2 eq.) to afford a colorless solid **20** (R*_f_*= 0.7, PE:EA=3:1, v/v; 111.8 mg, 0.18 mmol, 88%).

**^1^H NMR** (700 MHz, Chloroform-*d*) δ 7.73 (d, *J* = 8.5 Hz, 2H), 7.59 – 7.55 (m, 2H), 7.53 (d, *J* = 8.5 Hz, 2H), 7.17 – 7.10 (m, 2H), 6.71 (s, 1H), 4.10 (d, *J* = 10.6 Hz, 1H), 3.98 – 3.92 (m, 1H), S3.22 (dd, *J* = 9.3, 7.0 Hz, 1H), 3.06 (d, *J* = 3.8 Hz, 2H), 3.02 (dd, *J* = 9.3, 3.2 Hz, 1H), 2.33 – 2.23 (m, 2H), 2.22 – 2.15 (m, 1H), 1.95 (dd, *J* = 6.5, 4.9 Hz, 1H), 1.87 (t, *J* = 2.8 Hz, 1H), 1.60 (dd, *J* = 13.2, 3.4 Hz, 1H), 1.33 (d, *J* = 13.4 Hz, 1H), 1.30 (d, *J* = 1.2 Hz, 9H), 1.21 (s, 3H), 1.00 (s, 3H).

**^13^C NMR** (176 MHz, CDCl_3_) δ 156.9, 153.2, 138.0, 137.7, 132.3, 132.3, 128.0, 126.1, 86.5, 70.2, 58.2, 57.0, 50.6, 46.3, 40.1, 39.1, 35.3, 34.4, 34.1, 31.2, 27.4, 27.0, 23.5.

**HRMS** (ESI) for C_29_H_38_IN_2_O_4_S: calculated 637.1592 [M+H]^+^, found 637.1596.

[α]^20^_D_ = +8.1° (CHCl_3_, *c* = 1).

**((3aR,4aS,5aR,6aR)-2-((4-(tert-butyl)phenyl)sulfonyl)-5,5-dimethyloctahydrocyclopropa[f]isoindol-3a(1H)-yl)methyl (4-iodophenyl)carbamate (21):**

Following general procedure E2: From **S4** (31.3 mg, 0.08 mmol 1 eq.) and 4-Iodophenyl isocyanate (29.4 mg, 1.2 eq.) to afford a colorless solid **21** (R*_f_*= 0.8, PE:EA=3:1, v/v; 43.6 mg, 0.07 mmol, 90%).

**^1^H NMR** (700 MHz, Chloroform-*d*) δ 7.74 (d, *J* = 8.5 Hz, 2H), 7.59 (d, *J* = 8.8 Hz, 2H), 7.54 (d, *J* = 8.6 Hz, 2H), 7.17 (s, 2H), 6.77 (s, 1H), 3.93 (d, *J* = 10.5 Hz, 1H), 3.90 (d, *J* = 10.4 Hz, 1H), 3.57 (t, *J* = 8.9 Hz, 1H), 3.44 (d, *J* = 10.0 Hz, 1H), 2.76 (d, *J* = 10.0 Hz, 1H), 2.69 (t, *J* = 9.1 Hz, 1H), 2.12 – 2.05 (m, 1H), 1.65 (dd, *J* = 14.7, 7.1 Hz, 2H), 1.63 – 1.57 (m, 1H), 1.33 (s, 9H), 0.92 (s, 3H), 0.91 (s, 3H), 0.74 – 0.69 (m, 1H), 0.32 – 0.25 (m, 2H).

**^13^C NMR** (176 MHz, CDCl_3_) δ 156.9, 153.3, 138.1, 137.7, 132.1, 128.0, 125.9, 120.7, 86.6, 71.5, 55.2, 53.5, 44.0, 38.6, 35.3, 31.2, 28.6, 23.8, 20.0, 19.1, 17.6, 17.6, 14.9.

**HRMS** (ESI) for C_29_H_38_IN_2_O_4_S: calculated 659.1416 [M+H]^+^, found 659.1418.

[α]^20^_D_ = -12.3° (CHCl_3_, *c* = 0.5).

**(1R,2S,5R)-1'-((4-(tert-butyl)phenyl)sulfonyl)-6,6-dimethylspiro[bicyclo[3.1.1]heptane-2,3'-pyrrolidin]-3-yl (4-iodophenyl)carbamate (22):**

Following general procedure E2: From **S7** (58.74 mg, 0.15 mmol 1 eq.) and 4-Iodophenyl isocyanate (0.18 mmol, 44.4 mg, 1.2 eq.) to afford a colorless solid **22** (R*_f_*= 0.8, PE:EA=3:1, v/v; 57.5 mg, 0.09 mmol, 60%).

**^1^H NMR** (700 MHz, Methylene Chloride-*d*_2_) δ 7.68 (d, *J* = 8.5 Hz, 2H), 7.60 (d, *J* = 8.7 Hz, 2H), 7.51 (d, *J* = 8.5 Hz, 2H), 7.17 (d, *J* = 8.3 Hz, 2H), 6.71 (s, 1H), 5.01 (dd, *J* = 10.0, 6.1 Hz, 1H), 3.24 – 3.20 (m, 1H), 3.19 (d, *J* = 2.3 Hz, 2H), 3.11 – 3.05 (m, 1H), 2.52 (ddd, *J* = 14.3, 10.0, 4.6 Hz, 1H), 2.26 – 2.20 (m, 1H), 2.12 – 2.08 (m, 1H), 1.91 – 1.86 (m, 1H), 1.76 (ddt, *J* = 14.1, 6.3, 1.8 Hz, 1H), 1.67 (ddd, *J* = 13.8, 8.3, 6.3 Hz, 1H), 1.54 (t, *J* = 5.8 Hz, 1H), 1.30 (s, 9H), 1.19 (d, *J* = 10.7 Hz, 1H), 1.11 (s, 3H), 0.93 (s, 3H).

**^13^C NMR** (176 MHz, CD_2_Cl_2_) δ 156.9, 152.9, 138.3, 138.3, 133.6, 127.8, 126.3. 120.8, 86.4, 71.7, 61.0, 51.3, 49.9, 48.3, 39.9, 39.2, 35.4, 33.9, 31.6, 31.2, 27.5, 27.2, 22.7.

**HRMS** (ESI) for C_29_H_38_IN_2_O_4_S: calculated 637.1592 [M+H]^+^, found: 637.1602.

[α]^20^_D_ = -10.1° (CHCl_3_, *c* = 1.0).

**(1S,2R,3R,5S)-1'-((4-(tert-butyl)phenyl) sulfonyl)-5-isopropylspiro[bicyclo[3.1.0]hexane-2,3'-pyrrolidin]-3-yl (4-iodophenyl) carbamate (23):**

Following general procedure E2: From **S8** (45.9 mg, 0.12 mmol 1 eq.) and 4-Iodophenyl isocyanate (0.144 mmol, 35.3 mg, 1.2 eq.) to afford a colorless solid **23** (R*_f_*= 0.8, PE:EA=3:1, v/v; 25.6 mg, 0.04 mmol, 34%).

**^1^H NMR** (500 MHz, Chloroform-*d*) δ 7.76 (d, *J* = 8.6 Hz, 2H), 7.57 (d, *J* = 8.7 Hz, 2H), 7.53 (d, *J* = 8.5 Hz, 2H), 7.12 (d, *J* = 8.3 Hz, 2H), 6.51 (s, 1H), 4.81 (d, *J* = 6.8 Hz, 1H), 3.35 (dd, *J* = 8.1, 6.1 Hz, 2H), 3.19 (d, *J* = 9.5 Hz, 1H), 3.11 (d, *J* = 9.6 Hz, 1H), 2.10 (ddd, *J* = 14.9, 7.0, 1.9 Hz, 1H), 1.88 (dt, *J* = 12.6, 8.0 Hz, 1H), 1.79 (d, *J* = 14.8 Hz, 1H), 1.77 – 1.72 (m, 1H), 1.34 (s, 9H), 1.24 – 1.20 (m, 1H), 0.88 (d, *J* = 6.8 Hz, 3H), 0.81 (d, *J* = 6.9 Hz, 3H), 0.75 – 0.69 (m, 1H), 0.65 (t, *J* = 4.5 Hz, 1H), 0.30 (ddd, *J* = 8.4, 5.0, 1.8 Hz, 1H).

**^13^C NMR** (126 MHz, CDCl_3_) δ 156.6, 152.5, 138.1, 137.6, 133.9, 127.4, 126.3, 120.5, 86.6, 79.7, 58.3, 55.5, 47.3, 35.4, 35.3, 33.5, 32.4, 31.2, 30.6, 30.4, 20.1, 20.0, 14.3.

**HRMS** (ESI) for C_29_H_38_IN_2_O_4_S: calculated 659.1416 [M+H]^+^, found 659.1407.

[α]^20^_D_ = +4.3° (CHCl_3_, *c* = 0.5).

**(1S,2R,5S)-1'-((4-(tert-butyl)phenyl)sulfonyl)-5-isopropylspiro[bicyclo[3.1.0]hexane-2,3'-pyrrolidin]-3-yl (4-iodophenyl)carbamate (24):**

Following general procedure E2: From **S9** (45.9 mg, 0.12 mmol 1 eq) and 4-Iodophenyl isocyanate (0.144 mmol, 35.3 mg, 1.2 eq.) to afford a colorless solid **24** (R*_f_*= 0.8, PE:EA=3:1, v/v; 25.6 mg, 0.04 mmol, 34%).

**^1^H NMR** (500 MHz, Methylene Chloride-*d*_2_)δ 7.77 (d, *J* = 8.5 Hz, 2H), 7.60 (d, *J* = 8.7 Hz, 2H), 7.55 (d, *J* = 8.5 Hz, 2H), 7.19 (d, *J* = 8.8 Hz, 2H), 7.14 (s, 1H), 4.60 (d, *J* = 1.9 Hz, 1H), 3.68 (d, *J* = 10.0 Hz, 1H), 3.40 (s, 1H), 3.15 (d, *J* = 9.3 Hz, 1H), 2.97 (d, *J* = 9.9 Hz, 1H), 2.21 (dd, *J* = 12.4, 7.3 Hz, 1H), 1.92 (s, 2H), 1.65 (s, 1H), 1.32 (s, 9H), 1.17 (s, 1H), 0.90 (d, *J* = 6.8 Hz, 3H), 0.84 (d, *J* = 6.9 Hz, 3H), 0.63 – 0.57 (m, 1H), 0.45 (d, *J* = 2.0 Hz, 1H), 0.29 – 0.21 (m, 1H).

**^13^C NMR** (126 MHz, CD_2_Cl_2_) δ 157.0, 153.1, 138.4, 138.3, 134.9, 127.6, 126.5, 120.9, 86.3, 78.1, 53.5, 52.1, 47.5, 35.4, 34.1, 33.2, 32.9, 31.5, 31.2, 29.3, 19.9, 19.9, 13.4.

**HRMS** (ESI) for C_29_H_38_IN_2_O_4_S: calculated 659.1416 [M+H]^+^, found 659.1397.

[α]^20^_D_ = -4.0° (CHCl_3_, *c* = 0.5).

**(2-((4-(*tert*-butyl) phenyl) sulfonyl) octahydro-3aH-isoindol-3a-yl) methyl (4-iodophenyl) carbamate (25):**

To a stirred suspension of LiAlH_4_ (5 mg, 100 mol%) in THF (1 mL) at 0 ºC, the product obtained above **S6** dissolved in THF (0.1 mmol, 0.1 M) was added dropwise to the LiAlH_4_ solution. The reaction mixture was stirred at 0 ºC for 1 - 2 h until complete consumption of starting material. The reaction was quenched with water and stirred for 30 minutes The aqueous layer was extracted three times with Et_2_O and the combined organic layers were dried over anhydrous Na_2_SO_4_ and concentrated under reduced pressure. The crude product was used in the next step without purification.

Following general procedure E2: From the above crude product(35.15 mg, 0.1 mmol) and isocyanate (29.4 mg. 1.2 eq.). Purification by column chromatography (PE/EtOAc 4:1,v/v) afforded a colorless solid **25** (R*_f_*= 0.8, PE:EA=3:1, v/v; 37 mg, 0.06 mmol, 60%).

**^1^H NMR** (500 MHz, Chloroform-*d*) δ 7.76 (d, *J* = 8.6 Hz, 2H), 7.60 (d, *J* = 8.8 Hz, 2H), 7.53 (d, *J* = 8.6 Hz, 2H), 7.16 (d, *J* = 8.2 Hz, 2H), 6.68 (s, 1H), 4.04 (d, *J* = 10.9 Hz, 1H), 3.71 (d, *J* = 10.9 Hz, 1H), 3.41 (dd, *J* = 9.7, 7.6 Hz, 1H), 3.28 (d, *J* = 10.1 Hz, 1H), 3.24 (dd, *J* = 9.8, 6.6 Hz, 1H), 3.20 (d, *J* = 10.1 Hz, 1H), 1.98 – 1.91 (m, 1H), 1.51 (q, *J* = 3.9 Hz, 1H), 1.46 – 1.34 (m, 4H), 1.32 (s, 9H), 1.32 – 1.21 (m, 3H).

**^13^C NMR** (126 MHz, CDCl_3_) δ 156.7, 153.1, 138.1, 137.6, 134.3, 127.3, 126.2, 120.6, 86.6, 68.5, 55.1, 51.2, 43.6, 38.9, 35.3, 31.2, 27.5, 25.0, 22.0, 21.5.

**HRMS** (ESI) for C_26_H_34_IN_2_O_4_S: calculated 619.1103 [M+H]^+^, found: 619.1109.

**(2-((4-(tert-butyl)phenyl) sulfonyl) hexahydrocyclopenta [c]pyrrol-3a(1H)-yl) methyl (4-iodophenyl) carbamate (26):**

Following general procedure E2: From **S5** (272 mg, 0.69 mmol) and isocyanate (202.3 mg, 0.828 mmol, 1.2 eq.) afforded a colorless solid (280 mg, 0.44 mmol, 63 %). chromatography (PE/EtOAc 20:1 → 10 :1, v/v; R*_f_*= 0.7, PE:EA=3:1, v/v).

**^1^H NMR** (500 MHz, Chloroform-*d*) δ 7.71 (d, *J* = 8.6 Hz, 2H), 7.59 (d, *J* = 8.8 Hz, 2H), 7.54 (d, *J* = 8.5 Hz, 2H), 7.15 (d, *J* = 7.7 Hz, 2H), 6.68 (s, 1H), 4.02 (d, *J* = 10.6 Hz, 1H), 3.92 (d, *J* = 10.6 Hz, 1H), 3.22 (dd, *J* = 9.6, 7.9 Hz, 1H), 3.11 (d, *J* = 9.8 Hz, 1H), 3.01 (d, *J* = 9.8 Hz, 1H), 2.91 (dd, *J* = 9.6, 4.3 Hz, 1H), 2.29 (tt, *J* = 8.0, 4.7 Hz, 1H), 1.87 – 1.78 (m, 1H), 1.68 – 1.58 (m, 3H), 1.56 (dd, *J* = 6.6, 3.4 Hz, 1H), 1.50 – 1.43 (m, 1H), 1.34 (s, 9H).

**^13^C NMR** (126 MHz, CDCl_3_) δ 156.8, 153.3, 138.1, 137.6, 132.3, 128.0, 126.1, 120.6, 86.6, 69.9, 57.2, 54.4, 54.1, 45.9, 35.9, 35.3, 32.7, 31.2, 25.6.

**HRMS** (ESI) for C_25_H_32_IN_2_O_4_S: calculated 583.1122 [M+H]^+^, found 583.1130.

**Methyl (4R,6R)-2-((4-(tert-butyl) phenyl) sulfonyl)-3a-((((4-iodophenyl) carbamoyl) oxy) methyl)-5,5-dimethyl-3-phenyloctahydro-1H-4,6-methanoisoindole-1-carboxylate (27):**

To a solution of the **S11** (52.6 mg, 0.1 mmol, 1.0 eq.) in THF (0.1 M) was added DBU (26.9 µL, 2.0 eq.) and the respective isocyanate (49 mg, 2.0 eq.) and the solution was stirred at room temperature for 2 h, and then stir at 50 °C overnight. The solvent was removed under reduced pressure. Column chromatography (PE:EA=10:1, v/v) afforded the desired product **27** (R*_f_*= 0.6, PE:EA=3:1, v/v; 43.6 mg, 0.06 mmol, 57%).

**^1^H NMR** (500 MHz, Chloroform-*d*) δ 7.62 (d, *J* = 8.6 Hz, 3H), 7.58 (d, *J* = 8.8 Hz, 2H), 7.37 (d, *J* = 8.6 Hz, 2H), 7.27 (s, 1H), 7.18 – 7.13 (m, 3H), 7.06 – 7.02 (m, 1H), 6.84 (td, *J* = 7.6, 1.4 Hz, 2H), 4.90 (s, 1H), 4.64 (d, *J* = 3.1 Hz, 1H), 3.84 (s, 3H), 3.77 (d, *J* = 10.8 Hz, 1H), 3.06 (d, *J* = 10.8 Hz, 1H), 2.90 (d, *J* = 3.7 Hz, 1H), 2.62 – 2.54 (m, 1H), 2.28 – 2.20 (m, 1H), 1.90 (d, *J* = 6.2 Hz, 2H), 1.70 (dd, *J* = 13.5, 6.2 Hz, 1H), 1.29 (s, 9H), 1.23 (s, 4H), 0.99 (s, 3H), 0.88 (t, *J* = 7.1 Hz, 1H), 0.82 (d, *J* = 10.9 Hz, 1H).

**^13^C NMR** (126 MHz, CDCl_3_) δ 174.3, 157.0, 152.4, 137.9, 137.4, 134.5, 128.4, 128.1, 127.6, 127.4, 127.3, 126.9, 125.6, 120.1, 72.9, 72.2, 67.0, 56.6, 52.7, 47.2, 41.3, 39.6, 38.7, 37.2, 35.1, 31.1, 29.3, 27.7, 24.5.

**HRMS** (ESI) for C_37_H_44_IN_2_O_6_S: calculated 793.1784 [M+H]^+^, found 793.1766.

[α]^20^_D_ = +108.1º (CHCl_3_, *c* = 1.0).

**((3a*S*,4*R*,6*R*,7a*S*)-2-((4-(*tert*-butyl)phenyl)sulfonyl)-5,5-dimethyloctahydro-3a*H*-4,6-methanoisoindol-3a-yl)methyl (4-ethylphenyl)carbamate (28):**

Following general procedure E2: From **12** (20.0 mg, 51.1 µmol) and 4-ethylphenyl isocyanate. Purification by column chromatography (PE/EtOAc 15:1, v/v) afforded a colorless solid **28** (R*_f_*= 0.8, PE:EA=3:1, v/v; 21.0 mg, 40.0 µmol, 78%).

**^1^H NMR** (CDCl_3_, 500 MHz): δ 7.74 (d, *J* = 8.5 Hz, 2H), 7.53 (d, *J* = 8.6 Hz, 2H), 7.22 (m, 2H), 7.13 (d, *J* = 8.5 Hz, 2H), 6.32 (s, 1H), 4.05 (d, *J* = 10.6 Hz, 1H), 3.97 (d, *J* = 10.6 Hz, 1H), 3.17 – 3.04 (m, 3H), 3.01 (d, *J* = 9.8 Hz, 1H), 2.60 (q, *J* = 7.6 Hz, 2H), 2.35 – 2.23 (m, 2H), 2.23 – 2.16 (m, 1H), 1.96 (dd, *J* = 6.4, 4.9 Hz, 1H), 1.88 (dd, *J* = 5.5, 2.9 Hz, 1H), 1.65 (t, *J* = 3.4 Hz, 1H), 1.38 (d, *J* = 10.4 Hz, 1H), 1.30 (s, 9H), 1.24 – 1.19 (m, 7H), 1.01 (s, 3H).

**^13^C NMR** (CDCl_3_, 125 MHz):δ 156.8, 153.5, 135.2, 132.2, 128.7, 128.5, 128.1, 126.0, 119.1, 70.0, 58.2, 57.1, 50.6, 46.5, 40.2, 39.2, 35.3, 34.5, 34.3, 31.2, 28.4, 27.4, 27.1, 23.5, 15.9.

**HRMS** (ESI) for C_31_H_43_N_2_O_4_S: calculated 539.2938 [M+H]^+^, found 539.2941.

[α]^20^_D_ = -14.7° (CHCl_3_, *c* = 0.5).

**((3a*S*,4*R*,6*R*,7a*S*)-2-((4-(*tert*-butyl)phenyl)sulfonyl)-5,5-dimethyloctahydro-3a*H*-4,6-methanoisoindol-3a-yl)methyl (4-isopropylphenyl)carbamate (29):**

Following general procedure E2: From **12** (20.0 mg, 51.1 µmol) and 4-isopropylphenyl isocyanate. Purification by column chromatography (PE/EtOAc= 15:1, v/v) afforded a colorless solid **29** (R*_f_*= 0.8, PE:EA=3:1, v/v; 22.0 mg, 40.0 µmol, 78 %).

**^1^H NMR** (CDCl_3_, 500 MHz): δ 7.74 (d, *J* = 8.5 Hz, 2H), 7.53 (d, *J* = 8.5 Hz, 2H), 7.25 – 7.19 (m, 2H), 7.16 (d, *J* = 8.5 Hz, 2H), 6.31 (s, 1H), 4.06 (d, *J* = 10.7 Hz, 1H), 3.97 (d, *J* = 10.7 Hz, 1H), 3.18 – 3.05 (m, 3H), 3.00 (d, *J* = 9.8 Hz, 1H), 2.90 – 2.83 (m, 1H), 2.32 (ddd, *J* = 10.5, 3.2, 2.0 Hz, 1H), 2.31 – 2.22 (m, 2H), 2.20 (dtd, *J* = 10.4, 6.2, 1.7 Hz, 1H), 1.96 (dd, *J* = 6.5, 5.0 Hz, 1H), 1.88 (dd, *J* = 5.5, 2.9 Hz, 1H), 1.65 (d, *J* = 3.7 Hz, 1H), 1.38 (d, *J* = 10.4 Hz, 1H), 1.29 (s, 9H), 1.23 (s, 9H), 1.22 (d, *J* = 1.1 Hz, 6H), 1.01 (s, 3H).

**^13^C NMR** (CDCl_3_, 125 MHz):δ 156.8, 153.4, 135.3, 132.2, 128.1, 127.3, 127.1, 126.0, 119.0, 70.0, 58.2, 57.1, 50.7, 46.5, 40.2, 39.2, 35.3, 34.5, 34.4, 33.6, 31.2, 27.4, 27.1, 24.2, 23.5.

**HRMS** (ESI) for C_32_H_45_N_2_O_4_S: calculated 553.3095 [M+H]^+^, found 553.3100.

[α]^20^_D_ = -14.2° (CHCl_3_, *c* = 1).

**((3a*S*,4*R*,6*R*,7a*S*)-2-((4-(*tert*-butyl)phenyl)sulfonyl)-5,5-dimethyloctahydro-3a*H*-4,6-methanoisoindol-3a-yl)methyl (4-(*tert*-butyl)phenyl)carbamate (30):**

Following general procedure E2: From **12** (20.0 mg, 51.1 µmol) and 4-(*tert*-butyl) phenyl isocyanate. Purification by column chromatography (PE/EtOAc 6:1, v/v) afforded an orange oil **30** (R*_f_*= 0.8, PE:EA=3:1, v/v; 26.0 mg, 45.9 µmol, 90%).

**^1^H NMR** (CDCl_3_, 400 MHz): δ 7.74 (d, *J* = 8.5 Hz, 2H), 7.53 (d, *J =* 8.6 Hz, 2H), 7.32 (d, *J =* 8.7 Hz, 2H), 7.23 (d, *J =* 9.1 Hz, 2H), 6.33 (s, 1H), 4.10 – 4.03 (m, 1H), 3.97 (d, *J =* 10.7 Hz, 1H), 3.17 – 3.05 (m, 3H), 3.01 (d, *J =* 9.8 Hz, 1H), 2.37 – 2.25 (m, 2H), 2.26 – 2.18 (m, 1H), 1.99 – 1.95 (m, 1H), 1.89 (d, *J =* 6.1 Hz, 1H), 1.67 – 1.61 (m, 1H), 1.38 (d, *J =* 10.4 Hz, 1H), 1.30 (s, 9H), 1.29 (s, 9H), 1.22 (s, 3H), 1.01 (s, 3H).

**^13^C NMR** (CDCl_3_, 100 MHz):δ 156.8, 153.5, 135.0, 132.3, 128.1, 126.4, 126.0, 121.1, 118.8, 77.5, 70.0, 60.5, 58.2, 57.1, 50.7, 46.5, 40.2, 39.2, 35.3, 34.5, 34.4, 34.4, 31.5, 31.2, 27.4, 27.1, 23.5.

**HRMS** (ESI) for C_33_H_47_N_2_O_4_S: calculated 567.3251 [M+H]^+^, found 567.3257.

[α]^20^_D_ = -12.2° (CHCl_3_, *c* = 1.0).

**((3a*S*,4*R*,6*R*,7a*S*)-2-((4-(*tert*-butyl)phenyl)sulfonyl)-5,5-dimethyloctahydro-3a*H*-4,6-methanoisoindol-3a-yl)methyl [1,1'-biphenyl]-4-ylcarbamate (31):**

Following general procedure E2: From **12** (39.2 mg, 0.1 mmol) and 4-diphenyl isocyanate (23.4 mg, 1.2 eq.). Purification by column chromatography (PE/EtOAc 6:1, v/v) afforded an orange oil **31** (R*_f_*= 0.7, PE:EA=3:1, v/v; 26.0 mg, 45.9 µmol, 90%).

**^1^H NMR** (500 MHz, CDCl_3_) δ 7.75 (d, *J =* 8.5 Hz, 2H), 7.59 – 7.56 (m, 2H), 7.56 – 7.52 (m, 4H), 7.46 – 7.42 (m, 2H), 7.42 – 7.38 (m, 2H), 7.37 – 7.31 (m, 1H), 6.47 (s, 1H), 4.10 (d, *J =* 10.5 Hz, 1H), 4.01 (d, *J =* 10.7 Hz, 1H), 3.23 – 3.17 (m, 1H), 3.11 (d, *J =* 9.8 Hz, 1H), 3.09 – 3.03 (m, 2H), 2.35 – 2.26 (m, 2H), 2.25 – 2.17 (m, 1H), 1.98 (dd, *J =* 6.5, 5.0 Hz, 1H), 1.90 (dd, *J =* 5.5, 3.1 Hz, 1H), 1.67 – 1.62 (m, 1H), 1.37 (d, *J =* 10.6 Hz, 1H), 1.31 (s, 9H), 1.24 (s, 3H), 1.02 (s, 3H).

**^13^C NMR** (126 MHz, CDCl_3_) δ 156.9, 153.4, 143.3, 140.6, 137.0, 132.3, 128.9, 128.1, 127.9, 127.2, 126.9, 126.1, 119.2, 70.2, 58.2, 57.1, 50.7, 46.5, 40.2, 39.2, 35.3, 34.5, 34.3, 31.2, 27.4, 27.1, 23.5.

**HRMS** (ESI) for C_35_H_42_N_2_O_4_SNa: calculated 609.2763 [M+Na]^+^, found 609.2754.

[α]^20^_D_ = -6.2° (CHCl_3_, *c* = 0.5).

**((3a*S*,4*R*,6*R*,7a*S*)-2-((4-(*tert*-butyl)phenyl)sulfonyl)-5,5-dimethyloctahydro-3a*H*-4,6-methanoisoindol-3a-yl)methyl phenylcarbamate (32)：**

Following general procedure E2: From **12** (20.0 mg, 51.1 µmol) and phenyl isocyanate. Purification by column chromatography (PE/EtOAc 4:1, v/v) afforded a colorless solid **32** (R*_f_*= 0.7, PE:EA=3:1, v/v; 23.0 mg, 45.0 µmol, 88 %).

**^1^H-NMR** (CDCl_3_, 500 MHz): δ 7.74 (d, *J =* 8.5 Hz, 2H), 7.54 (d, *J =* 8.5 Hz, 2H), 7.37 – 7.26 (m, 4H), 7.08 (d, *J =* 6.0 Hz, 1H), 6.42 (s, 1H), 4.08 (d, *J =* 10.6 Hz, 1H), 3.98 (d, *J =* 10.6 Hz, 1H), 3.16 (dd, *J =* 9.2, 6.7 Hz, 1H), 3.13 – 3.04 (m, 2H), 3.02 (d, *J =* 9.8 Hz, 1H), 2.37 – 2.17 (m, 3H), 1.97 (dd, *J =* 6.5, 5.0 Hz, 1H), 1.89 (dd, *J =* 5.5, 2.9 Hz, 1H), 1.64 (td, *J =* 6.7, 3.4 Hz, 2H), 1.37 (d, *J =* 10.5 Hz, 1H), 1.30 (s, 9H), 1.22 (s, 4H), 1.01 (s, 3H).

**^13^C-NMR** (CDCl_3_, 125 MHz): δ 156.83, 153.42, 137.64, 132.18, 129.21, 128.10, 126.04, 123.80, 118.84, 70.05, 58.16, 57.06, 50.61, 46.44, 40.15, 39.17, 35.29, 34.49, 34.28, 31.16, 27.38, 27.08, 23.49.

**HRMS** (ESI) for C_29_H_39_N_2_O_4_S: calculated 511.2625 [M+H]^+^, found 511.2628.

[α]^20^_D_ = -18.2° (CHCl_3_, *c* = 0.5).

**((3a*S*,4*R*,6*R*,7a*S*)-2-((4-(*tert-*butyl)phenyl)sulfonyl)-5,5-dimethyloctahydro-3a*H*-4,6-methanoisoindol-3a-yl)methyl (2,4-dimethylphenyl)carbamate (33):**

Following general procedure E2: From **12** (39.2 mg, 0.1 mmol) and 1-isocyanato-2,4-dimethylbenzene (17.7 mg, 1.2 eq.). Purification by column chromatography (PE/EtOAc 10:1, v/v) afforded a colorless solid **33** (R*_f_*= 0.8, PE:EA=3:1, v/v; 27 mg, 50.0 µmol, 50%).

**^1^H NMR** (500 MHz, , CDCl_3_) δ 7.74 (d, *J =* 8.1 Hz, 2H), 7.53 (d, *J =* 8.5 Hz, 2H), 7.47 (s, 1H), 6.98 (d, *J =* 7.3 Hz, 2H), 6.03 (s, 1H), 4.08 (d, *J =* 10.6 Hz, 1H), 4.04 – 3.91 (m, 1H), 3.22 – 2.89 (m, 4H), 2.31 (dd, *J =* 7.7, 5.3 Hz, 1H), 2.29 (s, 3H), 2.25 (s, 1H), 2.23 – 2.19 (m, 1H), 2.18 (s, 3H), 1.95 (t, *J =* 5.7 Hz, 1H), 1.88 (dt, *J =* 5.4, 2.7 Hz, 1H), 1.66 – 1.59 (m, 2H), 1.36 (d, *J =* 10.5 Hz, 1H), 1.29 (s, 9H), 1.22 (s, 3H), 1.01 (s, 3H).

**^13^C NMR** (126 MHz, CDCl_3_) δ 156.6, 153.9, 137.6, 132.7, 132.1, 131.1, 127.9, 127.3, 125.9, 122.0 118.7, 69.9, 58., 57.0, 50.5, 46.3, 40.0, 39.0, 35.1, 34.3, 34.1, 31.0, 27.2, 26.9, 23.3, 20.8, 17.7.

**HRMS** (ESI) for C_31_H_43_N_2_O_4_S: calculated 539.2938 [M+H]^+^, found 539.2942.

[α]^20^_D_ = -14.7° (CHCl_3_, *c* = 0.5).

**((3a*S*,4*R*,6*R*,7a*S*)-2-((4-(*tert*-butyl)phenyl)sulfonyl)-5,5-dimethyloctahydro-3a*H*-4,6-methanoisoindol-3a-yl)methyl (4-fluorophenyl)carbamate (34):**

Following general procedure E2: From **12** (20.0 mg, 51.1 µmol) and 4-fluorophenyl isocyanate. Purification by column chromatography (PE/EtOAc 15:1, v/v) afforded a colorless solid **34** (R*_f_*= 0.7, PE:EA=3:1, v/v; 22.0 mg, 41.6 µmol, 81%).

**^1^H NMR** (CDCl_3_, 500 MHz): δ 7.74 (d, *J =* 8.5 Hz, 2H), 7.54 (d, *J =* 8.5 Hz, 2H), 7.28 (s_br_, 2H), 7.05 – 6.98 (m, 2H), 6.39 (s, 1H), 4.07 (d, *J =* 10.7 Hz, 1H), 4.01 (d, *J =* 10.7 Hz, 1H), 3.26 – 3.19 (m, 1H), 3.11 – 3.02 (m, 3H), 2.36 – 2.23 (m, 2H), 2.23 – 2.18 (m, 1H), 1.95 (t, *J =* 5.7 Hz, 1H), 1.89 (dd, *J =* 5.6, 3.1 Hz, 1H), 1.65 – 1.60 (m, 1H), 1.34 (d, *J =* 10.6 Hz, 1H), 1.31 (s, 9H), 1.23 (s, 3H), 1.01 (s, 3H).

**^13^C NMR** (CDCl_3_, 125 MHz):δ 156.9, 153.6, 133.7, 132.4, 128.1, 126.0, 120.8, 115.8, 70.3, 58.2, 57.0, 50.7, 46.5, 40.2, 39.2, 35.3, 34.5, 34.2, 31.2, 27.4, 27.1, 23.5.

**^19^F NMR** (CDCl_3_, 470 MHz) δ -119.20.

**HRMS** (ESI) for C_29_H_38_FN_2_O_4_S: calculated 529.2531 [M+H]^+^, found 529.2535.

[α]^20^_D_ = -4.3° (CHCl_3_, *c* = 1.0).

**((3a*S*,4*R*,6*R*,7a*S*)-2-((4-(*tert*-butyl)phenyl)sulfonyl)-5,5-dimethyloctahydro-3a*H*-4,6-methanoisoindol-3a-yl)methyl (4-chlorophenyl)carbamate (35):**

Following general procedure E2: From **12** (20.0 mg, 51.1 µmol) and 4-chlorophenyl isocyanate. Purification by column chromatography (PE/EtOAc 15:1, v/v) afforded a colorless solid **35** (R*_f_*= 0.6, PE:EA=3:1, v/v; 26.0 mg, 47.7 µmol, 93%).

**^1^H NMR** (CDCl_3_, 500 MHz): δ 7.74 (d, *J =* 8.5 Hz, 2H), 7.53 (d, *J =* 8.5 Hz, 2H), 7.32 – 7.23 (m, 4H), 6.55 (s, 1H), 4.10 (d, *J =* 10.6 Hz, 1H), 3.98 (d, *J =* 10.7 Hz, 1H), 3.21 (dd, *J =* 9.2, 6.7 Hz, 1H), 3.08 – 2.99 (m, 3H), 2.34 – 2.22 (m, 2H), 2.19 (ddd, *J =* 10.3, 5.0, 2.9 Hz, 1H), 1.95 (dd, *J =* 6.5, 5.0 Hz, 1H), 1.88 (dd, *J =* 5.7, 3.1 Hz, 1H), 1.64 – 1.58 (m, 1H), 1.34 (m, 1H), 1.31 (s, 9H), 1.22 (s, 3H), 1.00 (s, 3H).

**^13^C NMR** (CDCl_3_, 125 MHz):δ 156.9, 153.3, 136.4, 132.3, 129.2, 129.2, 128.1, 126.1, 120.0, 70.2, 58.2, 57.0, 50.6, 46.3, 40.1, 39.1, 35.3, 34.5, 34.1, 31.2, 27.4, 27.1, 23.5.

**HRMS** (ESI) for C_29_H_38_ClN_2_O_4_S: calculated 545.2235 [M+H]^+^, found 545.2243.

[α]^20^_D_ = -13.3° (CHCl_3_, *c* = 0.5).

**((3a*S*,4*R*,6*R*,7a*S*)-2-((4-(*tert*-butyl)phenyl)sulfonyl)-5,5-dimethyloctahydro-3a*H*-4,6-methanoisoindol-3a-yl)methyl (4-bromophenyl)carbamate (36):**

Following general procedure E2: From **12** (20.0 mg, 51.1 µmol) and 4-bromophenyl isocyanate. Purification by column chromatography (PE/EtOAc 15:1, v/v) afforded a colorless solid **36** (R*_f_*= 0.6, PE:EA=3:1,v/v; 22.0 mg, 37.7 µmol, 73%).

**^1^H NMR** (CDCl_3_, 700 MHz): δ = 1.01 (s, 3 H, CH_3_), 1.22 (s, 3 H, CH_3_), 1.31 (s, 9 H, 3 × CH_3_), 1.32–1.36 (m, 1 H, CH), 1.59–1.64 (m, 1 H, CH), 1.86–1.90 (m, 1 H, CH), 1.95 (t, *J =* 5.8 Hz, 1 H, CH), 2.16–2.22 (m, 1 H, CH), 2.24–2.32 (m, 2 H, 2 × CH), 3.02 (dd, *J =* 9.4, 3.2 Hz, 1 H, CH), 3.07 (s, 2 H, 2 × CH), 3.23 (t, *J =* 8.2 Hz, 1 H, CH), 3.98 (d, *J =* 10.6 Hz, 1 H, CH_A_), 4.10 (d, *J =* 10.6 Hz, 1 H, CH_B_), 6.57 (s, 1 H, NH), 7.20–7.28 (m, 2 H, 2 × Ar-H), 7.40 (d, *J =* 8.5 Hz, 2 H, 2 × Ar-H), 7.53 (d, *J =* 8.3 Hz, 2 H, 2 × Ar-H), 7.74 (d, *J =* 8.1 Hz, 2 H, 2 × Ar-H).

**^13^C NMR** (CDCl_3_, 175 MHz):δ = 23.5, 27.1, 27.4, 31.2, 34.1, 34.5, 35.2, 39.1, 40.2, 46.4, 50.6, 57.0, 58.2, 70.3, 116.3, 120.4, 126.1, 128.1, 132.1, 132.4, 136.9, 153.3, 156.9.

**HRMS** (ESI) for C_29_H_38_BrN_2_O_4_S: calculated 589.1730 [M+H]^+^, found 589.1740.

[α]^20^_D_ = -12.1° (CHCl_3_, *c* = 0.5).

**((3a*S*,4*R*,6*R*,7a*S*)-2-((4-(*tert*-butyl)phenyl)sulfonyl)-5,5-dimethyloctahydro-3a*H*-4,6-methanoisoindol-3a-yl)methyl (2-iodophenyl)carbamate (37):**

Following general procedure E2: From **12** (20.0 mg, 51.1 µkmol) and 2-iodophenyl isocyanate. Purification by column chromatography (PE/EtOAc 15:1, v/v) afforded a colorless solid **37** (R*_f_*= 0.7, PE:EA=3:1, v/v; 23.0 mg, 36.1 µmol, 71%).

**^1^H NMR** (CDCl_3_, 500 MHz): δ = 1.01 (s, 3 H, CH_3_), 1.23 (m, 3 H, CH_3_), 1.26 (s, 9 H, 3 × CH_3_), 1.41 (d, *J =* 10.5 Hz, 1 H, CH), 1.63–1.70 (m, 1 H, CH), 1.86–1.92 (m, 1 H, CH), 1.95–2.00 (m, 1 H, CH), 2.19–2.37 (m, 3 H, 3 × CH), 2.97 (d, *J =* 9.8 Hz,1 H, CH), 3.09–3.20 (s, 3 H, 3 × CH), 3.97 (d, *J =* 10.7 Hz, 1 H, CH_A_), 4.09 (d, *J =* 10.7 Hz, 1 H, CH_B_), 6.66 (s, 1 H, NH), 6.82 (td, *J =* 7.9, 1.5 Hz, 1 H, Ar-H), 7.30–7.34 (m, 1 H, Ar-H), 7.51–7.56 (m, 2 H, 2 × Ar-H), 7.72–7.78 (m, 3 H, 3 × Ar-H), 7.85–7.93 (m, 1 H, Ar-H).

**^13^C NMR** (CDCl_3_, 125 MHz):δ = 23.5, 27.1, 27.4, 31.1, 34.3, 34.5, 35.3, 39.2, 40.1, 46.4, 50.6, 57.0, 58.2, 70.3, 98.7, 125.7, 126.1, 128.1, 129.5, 129.6, 132.0, 138.1, 139.0, 153.3, 156.7.

**HRMS** (ESI) for C_29_H_38_IN_2_O_4_S: calculated 637.1592 [M+H]^+^, found 637.1600.

[α]^20^_D_ = -10.1° (CHCl_3_, *c* = 0.5).

**((3a*S*,4*R*,6*R*,7a*S*)-2-((4-(*tert*-butyl)phenyl)sulfonyl)-5,5-dimethyloctahydro-3a*H*-4,6-methanoisoindol-3a-yl)methyl (2-bromophenyl)carbamate (38):**

Following general procedure E2: From **12** (20.0 mg, 51.1 µmol) and 2-bromophenyl isocyanate. Purification by column chromatography (PE/EtOAc 15:1, v/v) afforded a colorless oil **38** (R*_f_*= 0.6, PE:EA=3:1,v/v; 18.0 mg, 30.5 µmol, 60%).

**^1^H NMR** (CDCl_3_, 500 MHz): δ = 1.01 (s, 3 H, CH_3_), 1.23 (m, 3 H, CH_3_), 1.27 (s, 9 H, 3 × CH_3_), 1.40 (d, *J =* 10.4 Hz, 1 H, CH), 1.63–1.70 (m, 1 H, CH), 1.86–1.92 (m, 1 H, CH), 1.95–2.01 (m, 1 H, CH), 2.18–2.38 (m, 3 H, 3 × CH), 2.97 (d, *J =* 9.8 Hz,1 H, CH), 3.07–3.20 (s, 3 H, 3 × CH), 3.97 (d, *J =* 10.7 Hz, 1 H, CH_A_), 4.11 (d, *J =* 10.6 Hz, 1 H, CH_B_), 6.85 (s, 1 H, NH), 6.92–6.97 (m, 1 H, Ar-H), 7.27–7.32 (m, 1 H, Ar-H), 7.49–7.56 (m, 3 H, 3 × Ar-H), 7.71–7.76 (m, 2 H, 2 × Ar-H), 7.96–8.06 (m, 1 H, Ar-H).z

**^13^C NMR** (CDCl_3_, 125 MHz):δ 156.9, 153.1, 139.1, 132.4, 130.5, 128.1, 126.7, 126.1, 122.9, 121.7, 117.3, 70.3, 58.2, 57.0, 50.7, 46.4, 40.2, 39.2, 35.3, 34.5, 34.2, 31.2, 27.4, 27.1, 23.5.

**HRMS** (ESI) for C_29_H_38_BrN_2_O_4_S: calculated 591.1710 [M+H]^+^, found 591.1716.

[α]^20^_D_ = -9.1° (CHCl_3_, *c* = 0.5).

**((3a*S*,4*R*,6*R*,7a*S*)-2-((4-(*tert*-butyl)phenyl)sulfonyl)-5,5-dimethyloctahydro-3a*H*-4,6-methanoisoindol-3a-yl)methyl (3-bromophenyl)carbamate (39):**

Following general procedure E2: From **39** (20.0 mg, 51.1 µmol) and 3-bromophenyl isocyanate. Purification by column chromatography (PE/EtOAc 15:1, v/v) afforded a colorless oil **39** (R*_f_*= 0.7, PE:EA=3:1,v/v; 25.0 mg, 42.4 µmol, 83%).

**^1^H NMR** (CDCl_3_, 700 MHz): δ = 1.01 (s, 3 H, CH_3_), 1.23 (m, 3 H, CH_3_), 1.31 (s, 9 H, 3 × CH_3_), 1.34 (d, *J =* 10.5 Hz, 1 H, CH), 1.60–1.65 (m, 1 H, CH), 1.87–1.91 (m, 1 H, CH), 1.93–1.97 (m, 1 H, CH), 2.18–2.22 (m, 1 H, CH), 2.24–2.33 (m, 2 H, 2 × CH), 3.02–3.07 (s, 2 H, 2 × CH), 3.09 (d, *J =* 9.9 Hz, 1 H, CH), 3.21 (dd, *J =* 9.3, 7.0 Hz, 1 H, CH), 4.00 (d, *J =* 10.6 Hz, 1 H, CH_A_), 4.09 (d, *J =* 10.7 Hz, 1 H, CH_B_), 6.55 (s, 1 H, NH), 7.15 (t, *J =* 8.0 Hz, 1 H, Ar-H), 7.18–7.20 (m, 1 H, Ar-H), 7.22–7.26 (m, 1 H, Ar-H), 7.52–7.56 (m, 2 H, 2 × Ar-H), 7.59 (s_br_, 1 H, Ar-H), 7.72–7.76 (m, 2 H, 2 × Ar-H).

**^13^C NMR** (CDCl_3_, 175 MHz):δ = 23.5, 27.1, 27.4, 31.2, 34.2, 34.5, 35.4, 39.2, 40.2, 46.4, 50.7, 57.0, 58.2, 70.3, 122.9, 126.1, 126.7, 128.1, 130.5, 132.4, 139.1, 153.1, 156.9.

**HRMS** (ESI) for C_29_H_38_BrN_2_O_4_S: calculated 589.1730 [M+H]^+^, found 589.1738.

[α]^20^_D_ = -12.3° (CHCl_3_, *c* = 0.5).

**((3a*S*,4*R*,6*R*,7a*S*)-2-((4-(*tert*-butyl)phenyl)sulfonyl)-5,5-dimethyloctahydro-3a*H*-4,6-methanoisoindol-3a-yl)methyl (3-aminophenyl)carbamate (40):**

Following general procedure C2: From **S42** (85.0 mg, 0.139 mmol) afforded an orange oil**,** which was used in next step without further purification (58.0 mg, 0.176 mmol, 93 %yield).

Following general procedure D1: From the crude amine obtained from above step (58.0 mg, 0.176 mmol) and 4-*tert*-butylbenzenesulfonyl chloride. Purification by column chromatography (PE/EtOAc 3:1, v/v) afforded a colorless oil **40** (R*_f_*= 0.3, PE:EA=1:1, v/v; 43.0 mg, 81.8 µmol, 46 %).

**^1^H NMR** (CDCl_3_, 500 MHz): δ 7.67 (d, *J =* 8.5 Hz, 2H), 7.47 (d, *J =* 8.6 Hz, 2H), 6.99 (t, *J =* 8.0 Hz, 1H), 6.88 (s, 1H), 6.58 – 6.52 (m, 1H), 6.44 – 6.35 (m, 2H), 4.00 (d, *J =* 10.7 Hz, 1H), 3.89 (d, *J =* 10.7 Hz, 1H), 3.13 (dd, *J =* 9.2, 6.7 Hz, 1H), 2.98 (dd, *J =* 10.8, 7.3 Hz, 3H), 2.26 – 2.15 (m, 2H), 2.11 (dt, *J =* 10.8, 3.8 Hz, 1H), 1.88 (dd, *J =* 6.5, 5.0 Hz, 1H), 1.81 (dd, *J =* 6.0, 3.5 Hz, 1H), 1.55 (dd, *J =* 12.7, 3.5 Hz, 1H), 1.23 (m, 10H), 1.15 (s, 3H), 0.93 (s, 3H).

**^13^C NMR** (CDCl_3_, 125 MHz):δ 156.9, 153.4, 142.2, 138.8, 132.2, 1130.0, 128.1, 127.1, 126.1, 111.1, 109.7, 69.9, 58.2, 57.0, 50.6, 46.4, 40.2, 39.1, 35.3, 34.4, 34.2, 27.4, 27.1, 23.5.

**HRMS** (ESI) for C_29_H_40_N_3_O_4_S: calculated 526.2734 [M+H]^+^, found 526.2732.

[α]^20^_D_ = -10.3° (CHCl_3_, *c* = 0.5).

**((3a*S*,4*R*,6*R*,7a*S*)-2-((4-(*tert*-butyl)phenyl)sulfonyl)-5,5-dimethyloctahydro-3a*H*-4,6-methanoisoindol-3a-yl)methyl (4-methoxyphenyl)carbamate (44)：**

Following general procedure C1: From **S43** (100 mg, 0.230 mmol) afforded a colorless solid **-**which was used in next stepwithout further purification (79.0 mg, 0.229 mmol, quant.).

Following general procedure D1: From the crude amine obtained from above step (79.0 mg, 0.229 mmol) and 4-*tert*-butylbenzenesulfonyl chloride. Purification by column chromatography (PE/EtOAc 10:1,v/v) afforded a colorless solid **44** (R*_f_*= 0.5, PE:EA=3:1, v/v; 90.0 mg, 0.166 mmol, 72%).

**^1^H NMR** (CDCl_3_, 600 MHz): δ = 1.00 (s, 3 H, CH_3_), 1.22 (s, 3 H, CH_3_), 1.30 (s, 9 H, 3 × CH_3_), 1.37 (d, *J =* 10.4 Hz, 1 H, CH), 1.60–1.67 (m, 2 H, 2 × CH), 1.86–1.90 (m, 1 H, CH), 1.92–1.97 (m, 1 H, CH), 2.16–2.34 (m, 3 H, 3 × CH), 2.97–3.21 (m, 4 H, 4 × CH), 3.79 (s, 3 H, OMe), 3.95–4.06 (m, 2 H, 2 × CH), 6.23 (s, 1 H, NH), 6.82–6.87 (m, 2 H, 2 × Ar-H), 7.17–7.25 (s_br_, 2 H, 2 × Ar-H), 7.51–7.56 (m, 2 H, 2 × Ar-H), 7.74 (d, *J =* 8.1 Hz, 2 H, 2 × Ar-H).

**^13^C NMR** (CDCl_3_, 150 MHz):δ = 23.5, 27.2, 27.4, 29.8, 31.2, 34.4, 34.5, 35.3, 39.2, 40.2, 46.6, 50.7, 55.7, 57.1, 58.2, 70.1, 114.4, 121.0, 126.0, 128.1, 130.7, 132.3, 153.8, 156.3, 156.8.

**HRMS** (ESI) for C_30_H_41_N_2_O_5_S: calculated 541.2731 [M+H]^+^, found 541.2736.

[α]^20^_D_ = -14.3° (CHCl_3_, *c* = 1).

**((3a*S*,4*R*,6*R*,7a*S*)-2-((4-(*tert*-butyl)phenyl)sulfonyl)-5,5-dimethyloctahydro-3a*H*-4,6-methanoisoindol-3a-yl)methyl (4-(trifluoromethyl)phenyl)carbamate (45):**

Following general procedure E2: From **12** (20.0 mg, 51.1 µmol) and 4-(trifluoromethyl)phenyl isocyanate. Purification by column chromatography (PE/EtOAc 4:1, v/v) afforded a colorless solid **45** (R*_f_*= 0.6, PE:EA=3:1, v/v; 27.0 mg, 46.7 µmol, 91%).

**^1^H NMR** (CDCl_3_, 400 MHz): δ 7.74 (d, *J =* 8.6 Hz, 2H), 7.56 (d, *J =* 8.5 Hz, 2H), 7.54 (d, *J =* 8.5 Hz, 2H), 7.47 (d, *J =* 8.6 Hz, 2H), 6.74 (s, 1H), 4.15 (d, *J =* 10.6 Hz, 1H), 4.02 (d, *J =* 10.7 Hz, 1H), 3.28 (dd, *J =* 9.3, 7.0 Hz, 1H), 3.11 (d, *J =* 9.8 Hz, 1H), 3.07 (d, *J =* 9.9 Hz, 1H), 3.01 (dd, *J =* 9.3, 3.0 Hz, 1H), 2.35 – 2.25 (m, 2H), 2.20 (ddd, *J =* 6.0, 4.3, 1.5 Hz, 1H), 1.96 (dd, *J =* 6.4, 5.0 Hz, 1H), 1.89 (dd, *J =* 5.6, 3.4 Hz, 1H), 1.62 (dd, *J =* 9.2, 2.7 Hz, 1H), 1.32-1.30 (m, 1H), 1.31 (s, 9H), 1.23 (s, 3H), 1.02 (s, 3H).

**^13^C NMR** (CDCl_3_, 100 MHz):δ 156.9, 153.1, 141.0, 132.5, 128.1, δ 126.5 (q, *J =* 3.7 Hz), 126.1, 118.3, 70.5, 58.2, 57.0, 50.7, 46.4, 40.2, 39.1, 35.3, 34.5, 34.0, 31.2, 27.4, 27.1, 23.6.

**^19^F NMR** (470 MHz, CDCl_3_) δ -62.05.

**HRMS** (ESI) for C_30_H_38_F_3_N_2_O_4_S: calculated 579.2499 [M+H]^+^, found 579.2501.

[α]^20^_D_ = -22.3° (CHCl_3_, *c* = 0.5).

**((3a*S*,4*R*,6*R*,7a*S*)-2-((4-(*tert-*butyl)phenyl)sulfonyl)-5,5-dimethyloctahydro-3a*H*-4,6-methanoisoindol-3a-yl)methyl (4-nitrophenyl)carbamate (46):**

Following general procedure E2: From **12** (20.0 mg, 51.1 µmol) and 4-nitrophenyl isocyanate. Purification by column chromatography (PE/EtOAc 15:1, v/v) afforded a colorless solid **46** (R*_f_*= 0.6, PE:EA=3:1, v/v; 24.0 mg, 43.2 µmol, 85%).

**^1^H NMR** (CDCl_3_, 400 MHz): δ = 1.02 (s, 3 H, CH_3_), 1.23 (s, 3 H, CH_3_), 1.32 (s, 9 H, 3 × CH_3_),1.30–1.36 (m, 1 H, CH), 1.55–1.63 (m, 1 H, CH), 1.86–1.92 (m, 1 H, CH), 1.94–1.99 (m, 1 H, CH), 2.15–2.23 (m, 1 H, CH), 2.25–2.34 (m, 2 H, 2 × CH), 2.97 (dd, *J =* 9.7, 3.3 Hz, 1 H, CH), 3.05 (d, *J =* 9.9 Hz, 1 H, CH), 3.19 (d, *J =* 9.9 Hz, 1 H, CH), 3.37 (dd, *J =* 9.4, 6.6 Hz, 1 H, CH), 4.03 (d, *J =* 10.7 Hz, 1 H, CH_A_), 4.17 (d, *J =* 10.6 Hz, 1 H, CH_B_), 7.27 (s, 1 H, NH), 7.52–7.59 (m, 4 H, 4 × Ar-H), 7.72–7.78 (m, 2 H, 2 × Ar-H), 8.16–8.22 (m, 2 H, 2 × Ar-H).

**^13^C NMR** (CDCl_3_, 100 MHz):δ = 23.6, 27.4, 31.2, 33.7, 34.3, 35.3, 39.1, 40.2, 46.2, 50.7, 57.0, 58.3, 70.7, 118.0, 125.3, 126.1, 128.0, 132.6, 143.2, 144.0, 152.9, 157.0.

**HRMS** (ESI) for C_29_H_38_N_3_O_6_S: calculated 556.2476 [M+H]^+^, found 556.2476.

[α]^20^_D_ = -9.3° (CHCl_3_, *c* = 1.0).

**((3a*S*,4*R*,6*R*,7a*S*)-2-((4-(*tert*-butyl)phenyl)sulfonyl)-5,5-dimethyloctahydro-3a*H*-4,6-methanoisoindol-3a-yl)methyl (4-cyanophenyl)carbamate (47):**

Following general procedure E2: From **12** (20.0 mg, 51.1 µmol) and 4-cyanophenyl isocyanate. Purification by column chromatography (PE/EtOAc 10:1, v/v) afforded a colorless solid **47** (R*_f_*= 0.7, PE:EA=3:1, v/v; 26.0 mg, 48.5 µmol, 95%).

**^1^H NMR** (CDCl_3_, 400 MHz): δ 7.74 (d, *J =* 8.6 Hz, 2H), 7.58 (d, *J =* 8.8 Hz, 2H), 7.54 (d, *J =* 8.6 Hz, 2H), 7.50 (d, *J =* 8.8 Hz, 2H), 7.10 (s, 1H), 4.17 (d, *J =* 10.7 Hz, 1H), 4.01 (d, *J =* 10.7 Hz, 1H), 3.34 (dd, *J =* 9.3, 6.6 Hz, 1H), 3.16 (d, *J =* 9.9 Hz, 1H), 3.04 (d, *J =* 9.9 Hz, 1H), 2.97 (dd, *J =* 9.5, 3.2 Hz, 1H), 2.28 (dt, *J =* 5.6, 2.5 Hz, 2H), 2.22 – 2.15 (m, 1H), 1.96 (t, *J =* 5.7 Hz, 1H), 1.92 – 1.86 (m, 1H), 1.61 – 1.56 (m, 1H), 1.30-1.34 (m, 1H), 1.31 (s, 9H), 1.22 (s, 3H), 1.01 (s, 3H).

**^13^CNMR** (CDCl_3_, 100 MHz):δ 157.0, 152.9, 142.2, 133.5, 132.5, 128.0, 126.1, 119.0, 118.5, 106.5, 70.6, 58.2, 57.0, 50.6, 46.2, 40.2, 39.1, 35.3, 34.4, 33.8, 31.2, 27.4, 27.0, 23.5.

**HRMS** (ESI) for C_30_H_38_N_3_O_4_S: calculated 536.2578 [M+H]^+^, found 536.2578.

[α]^20^_D_ = -8.3° (CHCl_3_, *c* = 1.0).

**((3a*S*,4*R*,6*R*,7a*S*)-2-((4-(*tert*-butyl)phenyl)sulfonyl)-5,5-dimethyloctahydro-3a*H*-4,6-methanoisoindol-3a-yl)methyl benzylcarbamate (48):**

Following general procedure E2: From **12** (15.0 mg, 38.3 µmol) and benzyl isocyanate. Purification by column chromatography (PE/EtOAc 15:1, v/v) afforded a colorless solid **48** (R*_f_*= 0.8, PE:EA=3:1, v/v; 14.0 mg, 26.7 µmol, 70%).

**^1^H NMR** (CDCl_3_, 500 MHz): δ = 1.00 (s, 3 H, CH_3_), 1.21 (s, 3 H, CH_3_), 1.30 (s, 9 H, 3 × CH_3_), 1.37 (d, *J =* 10.5 Hz, 1 H, CH), 1.58–1.66 (m, 2 H, 2 × CH), 1.82–1.92 (m, 2 H, 2 × CH), 2.12–2.35 (m, 3 H, 3 × CH), 2.97 (d, *J =* 9.7, 1 H, CH), 3.02–3.10 (m, 2 H, 2 × CH), 3.90 (d, *J =* 10.6 Hz, 1 H, CH_A_), 4.02 (d, *J =* 10.8 Hz, 1 H, CH_B_), 4.24 (dd, *J =* 14.9, 5.6 Hz, 1 H, CH_A_), 4.35 (dd, *J =* 14.9, 6.4 Hz, 1 H, CH_B_), 4.62 (t_br_, *J =* 6.2 Hz, 1 H, NH), 7.22–7.26 (m, 2 H, 2 × Ar-H), 7.27–7.32 (m, 1 H, Ar-H), 7.32–7.38 (m, 2 H, 2 × Ar-H), 7.46–7.51 (m, 2 H, 2 × Ar-H), 7.67–7.72(m, 2 H, 2 × Ar-H).

**^13^C NMR** (CDCl_3_, 125 MHz):δ = 23.5, 27.2, 27.4, 31.2, 34.5, 35.3, 39.2, 40.2, 45.2, 46.6, 50.8, 57.0, 58.2, 70.0, 125.9, 127.6, 127.8, 128.2, 128.9, 132.0, 138.4, 156.5, 156.7.

**HRMS** (ESI) for C_30_H_41_N_2_O_4_S: calculated 525.2782 [M+H]^+^, found 525.2782.

[α]^20^_D_ = -18.3° (CHCl_3_, *c* = 1).

**3aS,4R,6R,7aS)-2-((4-(tert-butyl)phenyl)sulfonyl)-5,5-dimethyloctahydro-3aH-4,6-methanoisoindol-3a-yl)methyl thiophen-2-ylcarbamate (49):**

Following general procedure E2: From **12** (19.6.0 mg, 50 µmol) and thiophen isocyanate. Purification by column chromatography (PE/EtOAc 15:1, v/v) afforded a colorless solid **49** (R*_f_*= 0.7, PE:EA=3:1, v/v; 25.0 mg, 50 µmol, quant.).

**^1^H NMR** (700 MHz, Methylene Chloride-d2) δ 7.71 (d, *J =* 8.1 Hz, 2H), 7.63 – 7.54 (m, 2H), 6.87 (s_br_, 2H), 6.82 (dd, *J =* 5.5, 3.7 Hz, 1H), 6.57 (s, 1H), 4.14 – 4.05 (m, 1H), 3.97 (d, *J =* 10.8 Hz, 1H), 3.15 (s, 1H), 3.05 (d, *J =* 9.9 Hz, 1H), 3.00 (dd, *J =* 25.0, 8.9 Hz, 2H), 2.36 – 2.29 (m, 1H), 2.26 (d, *J =* 11.8 Hz, 1H), 2.21 (dtt, *J =* 13.7, 7.4, 3.7 Hz, 1H), 1.97 (dd, *J =* 6.5, 4.9 Hz, 1H), 1.88 (tt, *J =* 5.6, 2.8 Hz, 1H), 1.64 – 1.57 (m, 1H), 1.33 (s, 1H), 1.32 (s, 9H), 1.22 (s, 3H), 1.00 (s, 3H).

**^13^C NMR** (176 MHz, CD_2_Cl_2_) δ 157.2, 153.5, 140.1, 132.4, 128.3, 126.4, 125.1, 118.1, 113.0, 70.9, 58.5, 57.3, 50.8, 46.7, 40.5, 39.4, 35.5, 34.7, 34.5, 31.2, 27.4, 27.3, 23.5.

**HRMS** (ESI) for C_27_H_37_N_2_O_4_S_2_: calculated 539.2014 [M+H]^+^, found 539.2001.

[α]^20^_D_ = -139.1° (CHCl_3_, *c* = 1.0).

**((3aS,4R,6R,7aS)-2-((4-(tert-butyl)phenyl)sulfonyl)-5,5-dimethyloctahydro-3aH-4,6-methanoisoindol-3a-yl)methyl pyridin-2-ylcarbamate (50):**

Following general procedure E2: From **12** (19.6.0 mg, 50 µmol) and thiophen isocyanate. Purification by column chromatography (PE/EtOAc 15:1, v/v) afforded a colorless solid **50** (R*_f_*= 0.5, PE:EA=3:1, v/v; 20.0 mg, 40 µmol, quant.).

**^1^H NMR** (500 MHz, Methylene Chloride-*d*_2_) δ 8.28 – 8.19 (m, 1H), 7.89 (d, *J =* 8.4 Hz, 1H), 7.71 (d, *J =* 8.5 Hz, 3H), 7.66 (s, 1H), 7.58 (d, *J =* 8.5 Hz, 2H), 7.05 – 6.99 (m, 1H), 4.07 (d, *J =* 10.7 Hz, 1H), 3.97 (d, *J =* 10.7 Hz, 1H), 3.16 (dd, *J =* 9.3, 7.0 Hz, 1H), 3.10 – 3.00 (m, 2H), 2.94 (d, *J =* 9.8 Hz, 1H), 2.38 – 2.29 (m, 1H), 2.24 (dddd, *J =* 24.8, 10.4, 5.2, 2.5 Hz, 2H), 1.98 (dd, *J =* 6.5, 5.0 Hz, 1H), 1.91 – 1.86 (m, 1H), 1.63 (ddd, *J =* 13.1, 4.2, 2.7 Hz, 1H), 1.35 (d, *J =* 10.4 Hz, 1H), 1.30 (s, 9H), 1.23 (s, 3H), 1.01 (s, 3H).

**^13^C NMR** (126 MHz, CD_2_Cl_2_) δ 157.2, 153.2, 151.8, 147.8, 139.0, 132.1, 128.3, 126.4, 119.3, 112.5 70.41, 58.6, 57.3, 50.8, 46.7, 40.6, 39.4, 35.4, 34.8, 34.5, 31.1, 27.4, 27.3, 23.5.

**HRMS** (ESI) for C_28_H_38_N_3_O_4_S: calculated 512,2578 [M+H]^+^, found 512.2578.

[α]^20^_D_ = -139.1° (CHCl_3_, *c* = 1.0).

**((3a*S*,4*R*,6*R*,7a*S*)-2-((4-(*tert*-butyl)phenyl)sulfonyl)-5,5-dimethyloctahydro-3a*H*-4,6-methanoisoindol-3a-yl)methyl cyclohexylcarbamate (51):**

Following general procedure E2: From **8** (15.0 mg, 38.2 µmol) and cyclohexyl isocyanate. Purification by column chromatography (PE/EtOAc 15:1, v/v) afforded a colorless oil **51** (R*_f_*= 0.8, PE:EA=3:1, v/v; 7.0 mg, 13.6 µmol, 35%).

**^1^H NMR** (CDCl_3_, 500 MHz): δ 7.73 (d, *J =* 8.6 Hz, 2H), 7.54 (d, *J =* 8.6 Hz, 2H), 4.29 (d, *J =* 8.3 Hz, 1H), 3.90 (m, 2H), 3.44 – 3.32 (m, 1H), 3.32 – 3.22 (m, 1H), 3.13 – 3.02 (m, 3H), 2.94 (d, *J =* 9.7 Hz, 1H), 2.28 (d, *J =* 12.3 Hz, 1H), 2.26 – 2.14 (m, 2H), 1.93 – 1.84 (m, 4H), 1.74 – 1.65 (m, 3H), 1.60 (dt, *J =* 13.2, 3.8 Hz, 2H), 1.35 (s, 9H), 1.32 – 1.28 (m, 1H), 1.21 (s, 3H), 1.06 (mf, *J =* 11.4 Hz, 2H), 0.99 (s, 3H).

**^13^C NMR** (CDCl_3_, 125 MHz): δ 156.7, 132.4, 128.1, 126.0, 69.4, 58.1, 57.0, 50.7, 50.0, 46.5, 40.2, 39.2, 35.3, 34.4, 33.6, 31.3, 27.4, 27.1, 25.6, 25.0, 24.0, 23.4.

**HRMS** (ESI) for C_29_H_45_N_2_O_4_S: calculated 517.3095 [M+H]^+^, found 517.3093.

[α]^20^_D_ = -39.5° (CHCl_3_, *c* = 1.0).

**((3a*S*,4*R*,6*R*,7a*S*)-2-((4-(*tert*-butyl) phenyl) sulfonyl)-5,5-dimethyloctahydro-3a*H*-4,6-methanoisoindol-3a-yl)methyl naphthalen-1-ylcarbamate (52):**

Following general procedure E2: From **12** (15.0 mg, 38.3 µmol) and 1-naphthyl isocyanate. Purification by column chromatography (PE/EtOAc 10:1 to 4:1, v/v) afforded a colorless solid **52** (R*_f_*= 0.7, PE:EA=3:1, v/v; 14.0 mg, 25.0 µmol, 65%).

**^1^H NMR** (CDCl_3_, 700 MHz): δ = 7.64–7.93 (m, 5 H, 5 × Ar-H), 7.41–7.62 (m, 5 H, 5 × Ar-H), 7.22–7.32 (m, 1 H, Ar-H), 6.58–6.71 (m, 1 H, NH), 3.98–4.21 (m, 2 H, 2 × CH), 2.98–3.32 (m, 3 H, 3 × CH), 2.12–2.45 (m, 3 H, 3 × CH), 1.81–2.07 (m, 2 H, 2 × CH), 1.52–1.72 (m, 2 H, 2 × CH), 1.33–1.43 (m, 1 H, CH), 1.14–1.30 (m, 12 H, 4 × CH_3_), 0.97–1.07 (s, 3 H, CH_3_).

**^13^C NMR** (CDCl_3_, 175 MHz): δ 156.8, 134.3, 132.4, 132.3, 128.9, 128.1, 126.5, 126.3, 126.1, 126.1, 125.9, 120.7, 70.5, 58.3, 57.1, 50.8, 46.6, 40.2, 39.2, 35.2, 34.6, 34.3, 31.1, 31.1, 27.4,

**HRMS** (ESI) for C_33_H_41_N_2_O_4_S: calculated 561.2782 [M+H]^+^, found 561.2790.

[α]^20^_D_ = -23.1° (CHCl_3_, *c* = 1.0).

**((3a*S*,4*R*,6*R*,7a*S*)-2-(isopropylsulfonyl)-5,5-dimethyloctahydro-3a*H*-4,6-methanoisoindol-3a-yl) methyl *p*-tolylcarbamate (54):**

Following general procedure C1: From **9** (1.0 g, 2.39 mmol) afforded a colorless solid amine without further purification(745.5 mg, 2.27 mmol, 95%).

Following general procedure D1: From the crude amine (50 mg, 0.15 mmol) and propane-2-sulfonyl chloride. Purification by column chromatography (PE/EtOAc 15:1, v/v) afforded a colorless solid **54** (R*_f_*= 0.7, PE:EA=3:1, v/v; 39.7 mg, 0.09 mmol, 60%).

**^1^H NMR** (CDCl_3_, 400 MHz): δ = 7.31 – 7.21 (m, 2H), 7.10 (d, *J =* 8.5 Hz, 2H), 6.80 (s, 1H), 4.22 (d, *J =* 10.7 Hz, 1H), 4.12 (dd, *J =* 14.1, 8.9 Hz, 1H), 3.68 (s, 1H), 3.52 (d, *J =* 10.3 Hz, 1H), 3.27 – 3.18 (m, 1H), 3.14 (dd, *J =* 9.9, 3.4 Hz, 1H), 2.39 – 2.24 (m, 5H), 2.00 – 1.96 (m, 1H), 1.93 (dd, *J =* 8.5, 4.9 Hz, 1H), 1.60 (dd, *J =* 9.2, 2.8 Hz, 1H), 1.37 (dt, *J =* 6.8, 3.4 Hz, 6H), 1.25 (s, 3H), 1.06 (s, 3H).

**^13^C NMR** (CDCl_3_, 100 MHz):δ = 153.8, 135.3, 129.7, 119.0, 70.2, 58.9, 57.2, 53.7, 51.1, 46.6, 40.4, 39.2, 34.9, 34.2, 27.5, 27.4, 23.7, 20.9, 16.9, 16.8.

**HRMS** (ESI) for C_23_H_35_O_4_N_2_S: calculated 435.2318 [M+H]^+^, found 435.2330.

[α]^20^_D_ = -105.1° (CHCl_3_, *c* = 1.0).

**((3a*S*,4*R*,6*R*,7a*S*)-2-(cyclohexylsulfonyl)-5,5-dimethyloctahydro-3a*H*-4,6-methanoisoindol-3a-yl)methyl *p*-tolylcarbamate (55):**

Following general procedure C1: From **9** (1.0 g, 2.39 mmol) afforded a colorless solid amine without further purification(745.5 mg, 2.27 mmol, 95%).

Following general procedure D1: From the crude amine (50 mg, 0.150 mmol) and cyclohexylsulfonyl chloride. Purification by column chromatography (PE/EtOAc 15:1, v/v) afforded a colorless foam **55** (R*_f_*= 0.8, PE:EA=3:1, v/v; 65 mg, 0.140 mmol, 90%).

**^1^H NMR** (CDCl_3_, 400 MHz): δ = 7.26 – 7.13 (m, 2H), 7.04 (d, *J =* 8.5 Hz, 2H), 6.69 (s, 1H), 4.16 (d, *J =* 10.7 Hz, 1H), 4.04 (d, *J =* 10.6 Hz, 1H), 3.61 (s, 1H), 3.45 (d, *J =* 10.3 Hz, 1H), 3.14 (d, *J =* 10.4 Hz, 1H), 3.07 (dd, *J =* 9.7, 3.1 Hz, 1H), 2.87 (tt, *J =* 12.0, 3.2 Hz, 1H), 2.32 – 2.16 (m, 6H), 2.08 (s, 2H), 1.92 (t, *J =* 5.6 Hz, 1H), 1.84 (dd, *J =* 18.7, 8.1 Hz, 3H), 1.63 (d, *J =* 11.6 Hz, 1H), 1.57 – 1.40 (m, 4H), 1.31 (d, *J =* 10.4 Hz, 1H), 1.25 – 1.07 (m, 7H), 0.99 (s, 3H).

**^13^C NMR** (CDCl_3_, 100 MHz):δ = 153.8, 135.3, 129.7, 119.0, 70.3, 61.7, 58.8, 57.2, 51.1, 46.6, 40.4, 39.2, 34.9, 34.1, 27.5, 27.4, 26.7, 26.6, 25.4, 25.4, 23.7, 20.9.

**HRMS** (ESI) for C_26_H_39_N_2_O_4_S: calculated 475.2625 [M+H]^+^, found 475.2650.

[α]^20^_D_ = -97.2° (CHCl_3_, *c* = 1.0).

**((3a*S*,4*R*,6*R*,7a*S*)-5,5-dimethyl-2-((4-(trifluoromethyl)phenyl)sulfonyl)octahydro-3a*H*-4,6-methanoisoindol-3a-yl)methyl *p*-tolylcarbamate (56):**

Following general procedure C1: From **9** (1.0 g, 2.39 mmol) afforded a colorless solid amine without further purification(745.5 mg, 2.27 mmol, 95%).

Following general procedure D1: From the crude amine (50 mg, 0.15 mmol) and 4-(trifluoromethyl) phenyl) sulfonyl chloride, Purification by column chromatography (PE/EtOAc 15:1, v/v) afforded a colorless solid **56** (R*_f_*= 0.7, PE:EA=3:1,v/v; 73.52 mg, 0.137 mmol, 90%).

**^1^H NMR** (500 MHz, Methylene Chloride-*d*_2_) δ 7.94 (d, *J =* 8.1 Hz, 2H), 7.82 (d, *J =* 8.2 Hz, 2H), 7.18 (d, *J =* 8.0 Hz, 2H), 7.11 (d, *J =* 8.2 Hz, 2H), 6.37 (s, 1H), 4.09 (d, *J =* 10.8 Hz, 1H), 3.93 (d, *J =* 10.7 Hz, 1H), 3.17 (dd, *J =* 9.3, 6.8 Hz, 1H), 3.10 (d, *J =* 9.9 Hz, 1H), 3.08 – 3.04 (m, 1H), 3.01 (d, *J =* 9.9 Hz, 1H), 2.39 – 2.31 (m, 2H), 2.30 (s, 3H), 2.23 (dtd, *J =* 10.4, 6.2, 1.9 Hz, 1H), 2.00 (t, *J =* 5.7 Hz, 1H), 1.89 (dt, *J =* 5.4, 2.7 Hz, 1H), 1.63 – 1.58 (m, 1H), 1.29 (d, *J =* 10.4 Hz, 1H), 1.23 (s, 3H), 1.02 (s, 3H).

**^13^C NMR** (126 MHz, CD_2_Cl_2_) δ 153.6, 139.4, 135.5, 134.6 (q, *J =* 32.8 Hz), 133.6, 129.9, 128.9, 126.6 (q, *J =* 3.7 Hz), 123.8 (q, *J =* 272.8 Hz), 119.0 (m), 69.9, 58.5, 57.3, 51.1, 46.7, 40.5, 39.4, 34.8, 34.6, 27.4, 27.3, 23.5, 20.8.

**^19^F NMR** (470 MHz, CD_2_Cl_2_) δ -63.37.

**HRMS** (ESI) for C_27_H_32_O_4_N_2_F_3_S: calculated 537.2029 [M+H]^+^, found 537.2050.

[α]^20^_D_ = -11.8° (CHCl_3_, *c* = 0.5).

**((3a*S*,4*R*,6*R*,7a*S*)-2-((4-methoxyphenyl)sulfonyl)-5,5-dimethyloctahydro-3a*H*-4,6-methanoisoindol-3a-yl)methyl *p*-tolylcarbamate (57):**

Following general procedure C1: From **9** (1.0 g, 2.39 mmol) afforded a colorless solid amine without further purification(745.5 mg, 2.27 mmol, 95%).

Following general procedure D1: From the crude amine (130 mg, 0.396 mmol) and 4-methoxybenzenesulfonyl chloride. Purification by column chromatography (PE/EtOAc 15:1, v/v) afforded a colorless solid **57** (R*_f_*= 0.6, PE:EA=3:1, v/v; 153 mg, 0.307 mmol, 78%).

**^1^H NMR** (CDCl_3_, 400 MHz): δ = 7.73–7.79 (m, 2 H, 2 × Ar-H), 7.15–7.21 (m, 2 H, 2 × Ar-H), 7.08–7.13 (m, 2 H, 2 × Ar-H), 6.96–7.02 (m, 2 H, 2 × Ar-H), 6.06 (s_br_, 1 H, NH), 3.92–4.03 (m, 2 H, 2 × CH), 3.75 (s, 3 H, OMe), 3.07–3.14 (m, 2 H, 2 × CH), 3.00–3.06 (m, 1 H, CH), 2.93 (d, *J =* 9.8 Hz, 1 H, CH), 2.31 (s, 3 H, CH_3_), 2.19–2.34 (m, 3 H, 3 × CH), 1.87–1.94 (m, 2 H, 2 × CH), 1.63–1.68 (m, 1 H, CH), 1.43 (d, *J =* 10.3 Hz, 1 H, CH), 1.23 (s, 3 H, CH_3_), 1.00 (s, 3 H, CH_3_).

**^13^C NMR** (CDCl_3_, 100 MHz):δ = 163.1, 135.0, 133.3, 130.6, 129.7, 126.4, 118.8, 114.1, 70.2, 58.5, 57.0, 55.6, 50.7, 46.9, 40.2, 39.1, 34.8, 34.7, 27.4 (2), 27.1, 23.6, 20.9.

**HRMS** (ESI) for C_27_H_35_N_2_O_5_S: calculated 499.2261 [M+H]^+^, found 499.2264.

[α]^20^_D_ = -12.8° (CHCl_3_, *c* = 0.5).

**((3a*S*,4*R*,6*R*,7a*S*)-5,5-dimethyl-2-((4-(trifluoromethoxy)phenyl)sulfonyl)octahydro-3a*H*-4,6-methanoisoindol-3a-yl)methyl *p*-tolylcarbamate (58):**

Following general procedure C1: From **9** (1.0 g, 2.39 mmol) afforded a colorless solid amine without further purification(745.5 mg, 2.27 mmol, 95%).

Following general procedure D1: From the crude amine (50 mg, 0.15 mmol) and 4-(trifluoromethoxy)phenylsulfonyl chloride. Purification by column chromatography (PE/EtOAc 15:1, v/v) afforded a colorless solid **58** (R*_f_*= 0.6, PE:EA=3:1, v/v; 77.00 mg, 0.140 mmol, 91%).

**^1^H NMR** (CDCl_3_, 400 MHz): δ = 7.78 (d, *J =* 8.6 Hz, 2H), 7.26 (d, *J =* 8.0 Hz, 2H), 7.10 (s, 2H), 7.03 (d, *J =* 8.5 Hz, 2H), 6.27 (s, 1H), 4.01 (d, *J =* 10.8 Hz, 1H), 3.91 (d, *J =* 10.7 Hz, 1H), 3.02 (dd, *J =* 13.8, 9.6 Hz, 3H), 2.93 (d, *J =* 9.7 Hz, 1H), 2.32 – 2.20 (m, 5H), 2.17 – 2.09 (m, 1H), 1.88 (t, *J =* 5.7 Hz, 1H), 1.84 – 1.76 (m, 1H), 1.62 – 1.45 (m, 1H), 1.26 (d, *J =* 10.5 Hz, 1H), 1.15 (s, 3H), 0.94 (s, 3H).

**^13^C NMR** (CDCl_3_, 100 MHz):δ = 153.5, 152.5, 152.4, 135.0, 133.7, 133.4, 130.3, 129.7, 121.6, 120.8, 119.1, 118.9, 69.9, 58.3, 57.0, 50.9, 46.7, 40.2, 39.2, 34.6, 34.4, 27.4, 27.2, 23.5, 20.9.

**HRMS** (ESI) for C_27_H_32_O_5_N_2_F_3_S: calculated 553.1979 [M+H]^+^, found 553.2000.

[α]^20^_D_ = -139.1° (CHCl_3_, *c* = 1.0).

**((3a*S*,4*R*,6*R*,7a*S*)-2-((2-bromophenyl)sulfonyl)-5,5-dimethyloctahydro-3a*H*-4,6-methanoisoindol-3a-yl)methyl *p*-tolylcarbamate (59):**

Following general procedure C1: From **9** (1.0 g, 2.39 mmol) afforded a colorless solid amine without further purification(745.5 mg, 2.27 mmol, 95%).

Following general procedure D1: From the crude amine (50 mg, 0.15 mmol) and 2-bromophenyl sulfonyl chloride. Purification by column chromatography (PE/EtOAc 15:1, v/v) afforded a colorless solid **59** (R*_f_*= 0.7, PE:EA=3:1, v/v; 70.85 mg, 0.130 mmol, 85%).

**^1^H NMR** (CDCl_3_, 400 MHz): δ = 8.03 (dd, *J =* 7.9, 1.7 Hz, 1H), 7.65 (dd, *J =* 7.8, 1.3 Hz, 1H), 7.36 (ddd, *J =* 7.9, 7.5, 1.3 Hz, 1H), 7.27 (td, *J =* 7.7, 1.8 Hz, 1H), 7.16 (d, *J =* 7.5 Hz, 2H), 7.03 (d, *J =* 8.2 Hz, 2H), 6.55 (s, 1H), 4.03 (dd, *J =* 34.5, 10.7 Hz, 2H), 3.53 (t, *J =* 9.0 Hz, 1H), 3.30 (d, *J =* 10.2 Hz, 1H), 3.17 – 3.08 (m, 2H), 2.34 – 2.09 (m, 6H), 1.88 – 1.80 (m, 2H), 1.61 – 1.54 (m, 1H), 1.28 (d, *J =* 10.5 Hz, 1H), 1.16 (s, *J =* 6.5 Hz, 3H), 0.96 (s, 3H).

**^13^C-NMR** (CDCl_3_, 100 MHz):δ = 153.6, 137.6, 135.9, 135.3, 133.9, 132.4, 129.7, 127.7, 120.9, 119.0, 70.3, 58.5, 56.8, 51.2, 46.7, 40.4, 39.1, 34.9, 34.1, 27.5, 27.4, 23.7, 20.9.

**HRMS** (ESI) for C_26_H_32_O_4_N_2_BrS: calculated 547.1261 [M+H]^+^, found 547.1289.

[α]^20^_D_ = -12.3° (CHCl_3_, *c* = 0.5).

**((3aS,4R,6R,7aS)-5,5-dimethyl-2-(pyridin-3-ylsulfonyl)octahydro-3aH-4,6-methanoisoindol-3a-yl)methyl (4-iodophenyl)carbamate (61):**

Following general procedure E2: From **S12** (42 mg, 0.1 mmol) and 1-iodo-4-isocyanatobenzene (29.4 mg, 0.12 mmol, 1.2 eq.). Purification by column chromatography (PE/EtOAc 10:1, v/v) afforded a colorless solid **61** (R*_f_*= 0.5, PE:EA=3:1, v/v; 54 mg, 0.09 mmol, 98%).

**^1^H NMR (**500 MHz, Methylene Chloride-*d*_2_) δ 9.01 (dd, *J =* 2.3, 0.9 Hz, 1H), 8.72 (dd, *J =* 4.9, 1.7 Hz, 1H), 8.08 (dt, *J =* 8.0, 2.0 Hz, 1H), 7.62 (d, *J =* 8.8 Hz, 2H), 7.47 (ddd, *J =* 8.0, 4.9, 0.9 Hz, 1H), 7.17 (d, *J =* 8.5 Hz, 2H), 6.51 (s, 1H), 4.04 (d, *J =* 10.8 Hz, 1H), 3.97 (d, *J =* 10.8 Hz, 1H), 3.18 (dd, *J =* 9.2, 6.8 Hz, 1H), 3.11 (td, *J =* 5.9, 3.1 Hz, 2H), 2.94 (d, *J =* 9.9 Hz, 1H), 2.34 (ddt, *J =* 9.2, 4.0, 2.4 Hz, 2H), 2.28 – 2.21 (m, 1H), 1.95 (dd, *J =* 6.5, 5.0 Hz, 1H), 1.90 (dt, *J =* 5.4, 2.8 Hz, 1H), 1.63 (d, *J =* 5.6 Hz, 1H), 1.30 (d, *J =* 10.5 Hz, 1H), 1.23 (s, 3H), 1.01 (s, 3H).

**^13^C NMR** (126 MHz, CD_2_Cl_2_) δ 153.9, 153.2, 149.0, 138.3, 138.2, 136.2, 131.9, 124.2, 86.5, 70.4, 58.8, 57.3, 51.1, 46.9, 40.5, 39.3, 34.9, 34.7, 27.6, 27.4, 23.5.

**HRMS** (ESI) for C_24_H_28_IN_3_O_4_SNa: calculated 604.0743 [M+Na]^+^, found 604.0738.

[α]^20^_D_ = 6.6° (CHCl_3_, *c* = 0.25).

**((3aS,4R,6R,7aS)-5,5-dimethyl-2-((6-methylpyridin-3-yl)sulfonyl)octahydro-3aH-4,6-methanoisoindol-3a-yl)methyl (4-iodophenyl)carbamate (62):**

Following general procedure E2: From **S13** (42 mg, 0.1 mmol) and 1-iodo-4-isocyanatobenzene (29.4 mg, 0.12 mmol, 1.2 eq.). Purification by column chromatography (PE/EtOAc 10:1, v/v) afforded a colorless solid **62** (R*_f_*= 0.5, PE:EA=3:1, v/v; 46.5 mg, 0.078 mmol, 78%).

**^1^H NMR** (500 MHz, Methylene Chloride-*d*_2_) δ 8.88 (d, *J =* 2.4 Hz, 1H), 7.95 (dd, *J =* 8.1, 2.4 Hz, 1H), 7.67 – 7.59 (m, 2H), 7.31 (d, *J =* 8.2 Hz, 1H), 7.17 (d, *J =* 8.3 Hz, 2H), 6.51 (s, 1H), 4.05 (d, *J =* 10.7 Hz, 1H), 3.97 (d, *J =* 10.7 Hz, 1H), 3.21 – 3.15 (m, 1H), 3.09 (td, *J =* 6.3, 3.2 Hz, 2H), 2.93 (d, *J =* 9.9 Hz, 1H), 2.52 (s, 3H), 2.39 – 2.29 (m, 2H), 2.24 (dtd, *J =* 10.5, 6.3, 1.7 Hz, 1H), 1.96 (dd, *J =* 6.5, 4.9 Hz, 1H), 1.90 (tt, *J =* 5.3, 2.6 Hz, 1H), 1.66 – 1.59 (m, 2H), 1.32 (d, *J =* 10.5 Hz, 1H), 1.23 (s, 3H), 1.01 (s, 3H).

**^13^C NMR** (126 MHz, CD_2_Cl_2_) δ 164.1, 153.2, 148.6, 138.3, 138.2, 136.4, 129.1, 123.7, 120.8, 86.5, 70.5, 58.8, 57.3, 51.1, 46.9, 40.6, 39.3, 34.8, 34.6, 27.6, 27.4, 24.8, 23.5.

**HRMS** (ESI) for C_25_H_31_IN_3_O_4_S: calculated 596.1074 [M+H]^+^, found 596.1084.

[α]^20^_D_ = 2.5° (CHCl_3_, *c* = 0.25).

**((3aS,4R,6R,7aS)-5,5-dimethyl-2-(thiophen-2-ylsulfonyl)octahydro-3aH-4,6-methanoisoindol-3a-yl)methyl (4-iodophenyl)carbamate (63):**

Following general procedure E2: From **S14** (71.6 mg, 0.21 mmol) and 1-iodo-4-isocyanatobenzene (77.2 mg, 0. 315 mmol, 1.5 eq.). Purification by column chromatography (PE/EtOAc 20:1 to 10:1, v/v) afforded a colorless solid **63** (R*_f_*= 0.7, PE:EA=3:1, v/v; 117.6 mg, 0.21 mmol, 78%).

**^1^H NMR** (700 MHz, Chloroform-*d*) δ 7.61 (s, 2H), 7.58 (dd, *J =* 3.7, 1.3 Hz, 1H), 7.56 (dd, *J =* 5.0, 1.4 Hz, 1H), 7.13 (dd, *J =* 5.0, 3.7 Hz, 2H), 7.12 – 7.08 (m, 1H), 6.34 (s, 1H), 4.06 (d, *J =* 10.7 Hz, 1H), 4.02 (d, *J =* 10.7 Hz, 1H), 3.18 (dd, *J =* 9.2, 6.8 Hz, 1H), 3.16 (d, *J =* 10.0 Hz, 1H), 3.12 (dd, *J =* 9.3, 2.3 Hz, 1H), 3.07 (d, *J =* 10.0 Hz, 1H), 2.38 – 2.31 (m, 2H), 2.25 (dtd, *J =* 10.6, 6.3, 1.6 Hz, 1H), 1.96 – 1.93 (m, 1H), 1.93 – 1.89 (m, 1H), 1.66 (dd, *J =* 8.8, 2.7 Hz, 1H), 1.37 (d, *J =* 10.5 Hz, 1H), 1.24 (s, 3H), 1.01 (s, 3H).

**^13^C NMR** (176 MHz, CDCl_3_) δ 153.0, 138.1, 137.6, 135.1, 133.2, 132.2, 127.7, 120.6, 86.6, 70.4, 58.8, 57.3, 50.9, 46.8, 40.2, 39.1, 34.7, 34.5, 27.4, 27.4, 23.6.

**HRMS** (ESI) for C_23_H_27_IN_2_O_4_S_2_Na: calculated 609.0355 [M+Na]^+^, found 609.0350.

[α]^20^_D_ = -25.8° (CHCl_3_, *c* = 0.49).

**((3a*S*,4*R*,6*R*,7a*S*)-5,5-dimethyl-2-(naphthalen-2-ylsulfonyl)octahydro-3a*H*-4,6-methanoisoindol-3a-yl)methyl (4-iodophenyl)carbamate (64):**

Following general procedure E2: From **S15** (38.5 mg, 0.1 mmol) and 1-iodo-4-isocyanatobenzene (29.4 mg, 1.2 eq.). Purification by column chromatography (PE/EtOAc 20:1 to 10:1, v/v) afforded a colorless solid **64** (R*_f_*= 0.7, PE:EA=3:1, v/v; 42.5 mg, 0.065 mmol, 65%).

**^1^H NMR** (500 MHz, Chloroform-*d*) δ 8.39 (s, 1H), 8.00 – 7.97 (m, 1H), 7.95 (d, *J =* 8.5 Hz, 1H), 7.86 – 7.79 (m, 2H), 7.62 (ddd, *J =* 7.0, 4.6, 1.8 Hz, 2H), 7.51 (d, *J =* 8.8 Hz, 2H), 6.80 (d, *J =* 8.6 Hz, 2H), 5.96 (s, 1H), 4.02 (d, *J =* 10.7 Hz, 1H), 3.95 (d, *J =* 10.8 Hz, 1H), 3.19 (d, *J =* 9.9 Hz, 1H), 3.17 – 3.14 (m, 2H), 3.07 (d, *J =* 9.8 Hz, 1H), 2.31 (dd, *J =* 4.9, 3.0 Hz, 2H), 2.26 – 2.17 (m, 1H), 1.89 (d, *J =* 6.2 Hz, 2H), 1.67 – 1.63 (m, 1H), 1.39 (d, *J =* 10.5 Hz, 1H), 1.22 (s, 3H), 0.99 (s, 3H).

**^13^C NMR** (126 MHz, CDCl_3_) δ 152.8, 138.8, 137.9, 137.3, 134.9, 132.1, 132.1, 130.3, 129.6, 129.4, 129.1, 128.1, 127.8, 123.7, 120.5, 86.5, 70.4, 58.6, 56.9, 50.8, 46.9, 40.2, 39.1, 34.7, 34.5, 27.4, 27.4, 23.6.

**HRMS** (ESI) for C_29_H_31_IN_2_O_4_SNa: calculated 653.0947 [M+Na]^+^, found 653.0946.

[α]^20^_D_ = -6.23º (CHCl_3_, *c* = 1.0).

**((3aS,4R,6R,7aS)-5,5-dimethyl-2-(phenylsulfonyl)octahydro-3aH-4,6-methanoisoindol-3a-yl)methyl (4-iodophenyl)carbamate (65):**

Following general procedure E2: From **S16** (38.5 mg, 0.1 mmol) and 1-iodo-4-isocyanatobenzene (29.4 mg, 1.2 eq.). Purification by column chromatography (PE/EtOAc 20:1 to 10:1) afforded a colorless solid **65** (R*_f_*= 0.7, PE:EA=3:1, v/v; 41 mg, 0.068 mmol, 68%).

**^1^H NMR** (500 MHz, CDCl_3_) δ 7.85 – 7.79 (m, 2H), 7.61 (d, *J =* 8.8 Hz, 2H), 7.57 – 7.51 (m, 3H), 7.14 – 7.06 (m, 2H), 6.17 (s, 1H), 4.01 (s, 2H), 3.14 – 3.05 (m, 3H), 2.98 (d, *J =* 9.8 Hz, 1H), 2.31 (dd, *J =* 6.8, 3.8 Hz, 2H), 2.27 – 2.18 (m, 1H), 1.90 (d, *J =* 6.6 Hz, 2H), 1.64 (dd, *J =* 8.8, 2.3 Hz, 1H), 1.36 (d, *J =* 10.5 Hz, 1H), 1.23 (s, 3H), 1.00 (s, 3H).

**^13^C NMR** (126 MHz, CDCl_3_) δ 152.9, 138.8, 138.1, 137.5, 134.7, 133.0, 130.3, 129.0, 128.4, 120.6, 86.6, 70.4, 58.5, 56.9, 50.72, 46.8, 40.2, 39.1, 34.7, 34.4, 27.4, 27.3, 23.6.

**HRMS** (ESI) for C_25_H_29_IN_2_O_4_SNa: calculated 603.0790 [M+Na]^+^, found 603.0792.

[α]^20^_D_ = -5.3° (CHCl_3_, *c* = 0.5).

**((3a*S*,4*R*,6*R*,7a*S*)-5,5-dimethyl-2-(o-tolylsulfonyl)octahydro-3a*H*-4,6-methanoisoindol-3a-yl)methyl (4-iodophenyl)carbamate (66)：**

Following general procedure E2: From **S17** (52.4 mg, 0.15 mmol) and 1-iodo-4-isocyanatobenzene (66.2 mg, 1.2 eq.). Purification by column chromatography (PE:EtOAc 20:1 to 10:1, v/v) afforded a colorless solid **66** (R*_f_*= 0.7, PE:EA=3:1, v/v; 60 mg, 0.1 mmol, 67%).

**1H NMR** (700 MHz, CDCl_3_) δ 7.93 (d, *J =* 7.0 Hz, 1H), 7.60 (d, *J =* 8.7 Hz, 2H), 7.44 (td, *J =* 7.5, 1.4 Hz, 1H), 7.32 (t, *J =* 7.5 Hz, 2H), 7.15 (d, *J =* 7.9 Hz, 2H), 6.69 (s, 1H), 4.15 (d, *J =* 10.6 Hz, 1H), 4.03 (d, *J =* 10.6 Hz, 1H), 3.41 (t, *J =* 8.3 Hz, 1H), 3.29 (d, *J =* 10.0 Hz, 1H), 3.08 – 3.01 (m, 2H), 2.67 (s, 3H), 2.35 (dd, *J =* 7.5, 3.8 Hz, 1H), 2.33 – 2.26 (m, 1H), 2.21 (dtd, *J =* 10.5, 6.3, 1.9 Hz, 1H), 1.93 – 1.87 (m, 2H), 1.59 – 1.54 (m, 1H), 1.30 (d, *J =* 10.5 Hz, 1H), 1.23 (s, 3H), 1.02 (s, 3H).

**^13^C NMR** (176 MHz, CDCl_3_) δ 153.2, 138.5, 138.1, 137.7, 135.5, 133.1, 132.9, 130.4, 126.2, 120.7, 86.5, 70.6, 57.9, 56.3, 50.9, 46.7, 40.3, 39.1, 34.7, 34.3, 27.4, 27.3, 23.6, 21.0.

**HRMS** (ESI) for C_26_H_31_IN_2_O_4_SNa: calculated 617.0942 [M+Na]^+^, found 617.0942.

[α]^20^_D_ = + 4.3° (CHCl_3_, *c* = 1.0).

**((3a*S*,4*R*,6*R*,7a*S*)-5,5-dimethyl-2-(*m*-tolylsulfonyl)octahydro-3a*H*-4,6-methanoisoindol-3a-yl)methyl (4-iodophenyl)carbamate (67):**

Following general procedure E2: From **S18** (52.4 mg, 0.15 mmol) and 1-iodo-4-isocyanatobenzene (29.4 mg, 1.2 eq.). Purification by column chromatography (PE/EtOAc 20:1 to 10:1, v/v) afforded a colorless solid **67** (R*_f_*= 0.7, PE:EA=3:1, v/v; 54 mg, 0.09 mmol, 60%).

**1H NMR** (700 MHz, CDCl_3_)δ 7.65 – 7.59 (m, 4H), 7.43 – 7.39 (m, 1H), 7.33 (d, *J =* 7.5 Hz, 1H), 7.09 (d, *J =* 8.2 Hz, 2H), 6.19 (s, 1H), 4.02 (s, 2H), 3.12 – 3.08 (m, 2H), 3.07 (dd, *J =* 9.3, 2.4 Hz, 1H), 2.99 (d, *J =* 9.9 Hz, 1H), 2.39 (s, 3H), 2.31 (ddt, *J =* 9.4, 4.9, 2.5 Hz, 2H), 2.26 – 2.21 (m, 1H), 1.94 – 1.88 (m, 2H), 1.64 (dd, *J =* 8.8, 2.6 Hz, 1H), 1.38 (d, *J =* 10.5 Hz, 1H), 1.23 (s, 3H), 1.00 (s, 3H).

**^13^C NMR** (176 MHz, CDCl_3_) δ 152.9, 139.3, 138.1, 137.6, 134.7, 133.8, 128.8, 128.7, 125.5, 120.6, 86.7, 70.4, 58.5, 56.9, 50.8, 46.8, 40.2, 39.1, 34.7, 34.4, 27.4, 27.3, 23.6, 21.6.

**HRMS** (ESI) for C_26_H_32_IN_2_O_4_S: calculated 595.1122 [M+H]^+^, found 595.1114.

[α]^20^_D_ = - 8.3° (CHCl_3_, *c* = 1.0).

**((3a*S*,4*R*,6*R*,7a*S*)-5,5-dimethyl-2-tosyloctahydro-3a*H*-4,6-methanoisoindol-3a-yl)methyl (4-iodophenyl)carbamate (68):**

Following general procedure E2: From **S19** (104.8 mg, 0.3 mmol) and 1-iodo-4-isocyanatobenzene (88 mg, 1.2 eq.). Purification by column chromatography (PE/EtOAc 20:1 to 10:1, v/v) afforded a colorless solid **68** (R*_f_*= 0.7, PE:EA=3:1, v/v; 135 mg, 0.23 mmol, 76%).

**^1^H NMR** (500 MHz, CDCl_3_) δ 7.70 (d, *J =* 8.2 Hz, 2H), 7.61 (d, *J =* 8.8 Hz, 2H), 7.32 (d, *J =* 7.7 Hz, 2H), 7.10 (d, *J =* 8.3 Hz, 2H), 6.24 (s, 1H), 4.04 (d, *J =* 10.7 Hz, 1H), 3.98 (d, *J =* 10.7 Hz, 1H), 3.12 (dd, *J =* 9.2, 6.8 Hz, 1H), 3.09 (d, *J =* 9.8 Hz, 1H), 3.06 (dd, *J =* 9.0, 2.5 Hz, 1H), 2.98 (d, *J =* 9.9 Hz, 1H), 2.34 (s, 3H), 2.33 – 2.27 (m, 2H), 2.22 (dtd, *J =* 10.5, 6.3, 1.7 Hz, 1H), 1.94 – 1.90 (m, 1H), 1.89 (dt, *J =* 5.1, 2.7 Hz, 1H), 1.66 – 1.62 (m, 1H), 1.37 (d, *J =* 10.5 Hz, 1H), 1.23 (s, 3H), 1.00 (s, 3H).

**^13^C NMR** (126 MHz, CDCl_3_) δ 153.0, 143.9, 138.1, 137.6, 132.0, 129.6, 128.4, 120.6, 86.7, 70.4, 58.4, 57.0, 50.7, 46.7, 40.2, 39.1, 34.7, 34.4, 27.4, 27.3, 23.6, 21.7.

**HRMS** (ESI) for C_26_H_32_IN_2_O_4_S: calculated 595.1122 [M+H]^+^, found 595.1131.

[α]^20^_D_ = - 5.3° (CHCl_3_, *c* = 1.0).

**((3a*S*,4*R*,6*R*,7a*S*)-2-((4-isopropylphenyl)sulfonyl)-5,5-dimethyloctahydro-3a*H*-4,6-methanoisoindol-3a-yl)methyl (4-iodophenyl)carbamate (69):**

Following general procedure E2: From **S20** (56.63 mg, 0.15 mmol) and 1-iodo-4-isocyanatobenzene (66.2 mg, 1.2 eq.). Purification by column chromatography (PE/EtOAc 20:1 to 10:1, v/v) afforded a colorless solid **69** (R*_f_*= 0.7, PE:EA=3:1, v/v; 66.3 mg, 0.11 mmol, 71%).

**^1^H NMR** (700 MHz, CDCl_3_) δ 7.73 (d, *J =* 8.4 Hz, 2H), 7.59 (d, *J =* 8.7 Hz, 2H), 7.37 (d, *J =* 8.3 Hz, 2H), 7.12 (d, *J =* 8.2 Hz, 2H), 6.45 (s, 1H), 4.07 (d, *J =* 10.6 Hz, 1H), 4.00 (d, *J =* 10.6 Hz, 1H), 3.19 (dd, *J =* 9.3, 6.9 Hz, 1H), 3.07 (d, *J =* 9.9 Hz, 1H), 3.06 – 3.02 (m, 2H), 2.93 (p, *J =* 6.9 Hz, 1H), 2.33 – 2.28 (m, 1H), 2.28 – 2.24 (m, 1H), 2.20 (ddt, *J =* 10.6, 6.4, 3.2 Hz, 1H), 1.94 (dd, *J =* 6.5, 4.9 Hz, 1H), 1.88 (dt, *J =* 5.4, 2.7 Hz, 1H), 1.64 – 1.61 (m, 1H), 1.33 (d, *J =* 10.5 Hz, 1H), 1.24 (d, *J =* 6.9 Hz, 3H), 1.23 – 1.21 (d, *J =* 6.9 Hz, s, 6H), 1.00 (s, 3H).

**^13^C NMR** (176 MHz, CDCl_3_) δ 154.6, 153.1, 138.1, 137.6, 132.6, 128.4, 127.2, 120.7, 86.6, 70.3, 58.3, 57.0, 50.7, 46.5, 40.2, 39.1, 34.5, 34.3, 34.2, 27.4, 27.1, 23.8, 23.7 23.5.

**HRMS** (ESI) for C_28_H_37_IN_2_O_4_SNa: calculated 623.1435 [M+Na]^+^, found 623.1443.

[α]^20^_D_ = -6.0° (CHCl_3_, *c* = 1.0).

**((3a*S*,4*R*,6*R*,7a*S*)-2-([1,1'-biphenyl]-4-ylsulfonyl)-5,5-dimethyloctahydro-3a*H*-4,6-methanoisoindol-3a-yl)methyl (4-iodophenyl)carbamate (70):**

Following general procedure E2: From **S21** (61.7 mg, 0.15 mmol) and 1-iodo-4-isocyanatobenzene (66.2 mg, 1.2 eq.). Purification by column chromatography (PE/EtOAc 20:1 to 10:1, v/v) afforded a colorless solid **70** (R*_f_*= 0.6, PE:EA=3:1, v/v; 91 mg, 0.14 mmol, 92%).

**^1^H NMR** (700 MHz, CDCl_3_) δ 7.88 (d, *J =* 8.4 Hz, 2H), 7.75 (d, *J =* 8.4 Hz, 2H), 7.54 (dd, *J =* 7.6, 2.0 Hz, 2H), 7.46 – 7.43 (m, 3H), 7.41 (d, *J =* 8.7 Hz, 2H), 6.93 (d, *J =* 8.2 Hz, 2H), 6.18 (s, 1H), 4.04 (d, *J =* 10.6 Hz, 1H), 4.00 (d, *J =* 10.8 Hz, 1H), 3.16 (d, *J =* 10.0 Hz, 1H), 3.15 – 3.12 (m, 2H), 3.02 (d, *J =* 10.0 Hz, 1H), 2.36 – 2.30 (m, 2H), 2.24 (dtd, *J =* 10.5, 6.2, 1.5 Hz, 1H), 1.95 – 1.89 (m, 2H), 1.70 – 1.63 (m, 1H), 1.42 (d, *J =* 10.5 Hz, 1H), 1.23 (s, 3H), 1.00 (s, 3H).

**^13^C NMR** (176 MHz, CDCl_3_) δ 153.0, 145.8, 138.9, 138.0, 137.3, 133.4, 129.3, 128.9, 128.8, 127.5, 127.3, 120.5, 86.7, 70.5, 58.6, 57.0, 50.8, 46.9, 40.2, 39.1, 34.8, 34.5, 27.4, 27.4, 23.6.

**HRMS** (ESI) for C_31_H_33_IN_2_O_4_SNa: calculated 679.1103 [M+Na]^+^, found 679.1102.[α]^20^_D_ = -6.0° (CHCl_3_, *c* = 1.0).

**((3a*S*,4*R*,6*R*,7a*S*)-2-((4-cyclohexylphenyl)sulfonyl)-5,5-dimethyloctahydro-3a*H*-4,6-methanoisoindol-3a-yl)methyl (4-iodophenyl)carbamate (71):**

Following general procedure E2: From **S22** (41.8 mg, 0.1 mmol) and 1-iodo-4-isocyanatobenzene (29.4 mg, 1.2 eq.). Purification by column chromatography (PE/EtOAc 20:1 to 10:1, v/v) afforded a colorless solid **71** (R*_f_*= 0.7, PE:EA=3:1, v/v; 61 mg, 0.09 mg, 92%).

**^1^H NMR** (700 MHz, CD_2_Cl_2_**)** δ 7.69 (d, *J =* 8.3 Hz, 2H), 7.61 (d, *J =* 8.8 Hz, 2H), 7.36 (d, *J =* 8.2 Hz, 2H), 7.13 (d, *J =* 8.2 Hz, 2H), 6.41 (s, 1H), 4.06 (d, *J =* 10.6 Hz, 1H), 3.92 (d, *J =* 10.6 Hz, 1H), 3.13 (dd, *J =* 9.2, 7.1 Hz, 1H), 3.05 (d, *J =* 9.9 Hz, 1H), 3.03 (dd, *J =* 9.2, 3.0 Hz, 1H), 2.93 (d, *J =* 9.9 Hz, 1H), 2.52 – 2.46 (m, 1H), 2.32 (tdd, *J =* 12.7, 3.0, 1.9 Hz, 1H), 2.29 – 2.25 (m, 1H), 2.21 (dtd, *J =* 10.4, 6.3, 2.0 Hz, 1H), 1.95 (dd, *J =* 6.5, 5.0 Hz, 1H), 1.88 (dt, *J =* 5.4, 2.7 Hz, 1H), 1.83 – 1.77 (m, 3H), 1.76 – 1.71 (m, 2H), 1.61 (ddd, *J =* 13.1, 4.3, 2.8 Hz, 1H), 1.40 – 1.31 (m, 5H), 1.27 – 1.23 (m, 1H), 1.22 (s, 3H), 1.00 (s, 3H).

**^13^C NMR** (176 MHz, CD_2_Cl_2_) δ 154.1, 153.3, 138.3, 138.2, 132.4, 128.6, 127.7, 120.8, 86.5, 70.4, 58.7, 57.3, 50.8, 46.8, 44.8, 40.6, 39.3, 34.8, 34.6, 34.5, 34.3, 27.4, 27.4, 27.1, 27.0, 26.3, 23.5.

**HRMS** (ESI) for C_31_H_39_IN_2_O_4_SNa: calculated 685.1572 [M+Na]^+^, found 685.1568.

[α]^20^_D_ = -4.3° (CHCl_3_, *c* = 1.0).

**((3a*S*,4*R*,6*R*,7a*S*)-2-(mesitylsulfonyl)-5,5-dimethyloctahydro-3a*H*-4,6-methanoisoindol-3a-yl)methyl (4-iodophenyl)carbamate (72):**

Following general procedure E2: From **S23** (30.2 mg, 0.08 mmol) and 1-iodo-4-isocyanatobenzene (23.5 mg, 1.2 eq.). Purification by column chromatography (PE/EtOAc 20:1 to 10:1, v/v) afforded a colorless solid **72** (R*_f_*= 0.7, PE:EA=3:1, v/v; 26 mg, 52 %).

**^1^H NMR** (700 MHz, CDCl_3_)δ 7.60 (d, *J =* 8.8 Hz, 2H), 7.22 – 7.14 (m, 2H), 6.95 (s, 2H), 6.94 (s, 1H), 4.20 (d, *J =* 10.6 Hz, 1H), 4.04 (d, *J =* 10.6 Hz, 1H), 3.50 (t, *J =* 8.4 Hz, 1H), 3.39 (d, *J =* 9.8 Hz, 1H), 2.99 (dd, *J =* 9.4, 3.4 Hz, 1H), 2.96 (d, *J =* 9.8 Hz, 1H), 2.65 (s, 6H), 2.40 – 2.34 (m, 1H), 2.30 (m, 1H), 2.28 (s, 3H), 2.22 – 2.16 (m, 1H), 1.89 (d, *J =* 6.4 Hz, 2H), 1.53 (ddd, *J =* 13.0, 4.5, 2.2 Hz, 1H), 1.25 (d, *J =* 10.4 Hz, 1H), 1.22 (s, 3H), 1.03 (s, 3H).

**^13^C NMR** (176 MHz, CDCl_3_) δ 153.4, 142.9, 140.4, 138.1, 137.8, 132.1, 131.9, 120.7, 86.5, 70.7, 57.1, 55.6, 50.8, 46.7, 40.3, 39.1, 34.6, 34.4, 27.4, 27.3, 23.6, 23.2, 21.1.

**HRMS** (ESI) for C_28_H_36_IN_2_O_4_S: calculated 623.1435 [M+H]^+^, found 623.1443.

[α]^20^_D_ = +10.3° (CHCl_3_, *c* = 1.0).

**((3a*S*,4*R*,6*R*,7a*S*)-5,5-dimethyl-2-((5,5,8,8-tetramethyl-5,6,7,8-tetrahydronaphthalen-2-yl)sulfonyl)octahydro-3aH-4,6-methanoisoindol-3a-yl)methyl (4-iodophenyl)carbamate）(73):**

Following general procedure E2: From **S24** (66.85 mg, 0.15 mmol) and 1-iodo-4-isocyanatobenzene (45 mg, 0.18 mmol, 1.2 eq.). Purification by column chromatography (PE/EtOAc 20:1 to 10:1, v/v) afforded a colorless solid **73** (R*_f_*= 0.7, PE:EA=3:1, v/v; 81 mg, 78%).

**^1^H NMR** (700 MHz, CDCl_3_) δ 7.71 (d, *J =* 2.1 Hz, 1H), 7.61 – 7.58 (m, 2H), 7.53 (dd, *J =* 8.3, 2.0 Hz, 1H), 7.44 (d, *J =* 8.3 Hz, 1H), 7.13 (d, *J =* 7.5 Hz, 2H), 6.54 (s, 1H), 4.10 (d, *J =* 10.6 Hz, 1H), 3.97 (d, *J =* 10.6 Hz, 1H), 3.19 (dd, *J =* 9.4, 7.2 Hz, 1H), 3.05 (s_br_, 2H), 2.99 (dd, *J =* 9.4, 3.4 Hz, 1H), 2.30 (m, 1H), 2.24 (m, 1H), 2.18 (ddd, *J =* 10.4, 5.2, 1.9 Hz, 1H), 1.96 (dd, *J =* 6.5, 5.0 Hz, 1H), 1.88 (dt, *J =* 5.8, 3.0 Hz, 1H), 1.68 (s_br_, 4H), 1.61 (d, *J =* 1.3 Hz, 1H), 1.29 (m, 4H), 1.27 (m, 9H), 1.22 (s, 3H), 1.00 (s, 3H).

**^13^C NMR (**176 MHz, CDCl_3_) δ 153.2, 150.7, 146.3, 138.1, 137.6, 132.2, 127.4, 126.6, 125.0, 120.7, 86.7, 70.3, 58.1, 57.1, 50.5, 46.4, 40.2, 39.2, 34.8, 34.7, 34.7, 34.7, 34.4, 34.1, 31.9, 31.8, 31.7, 27.4, 27.0, 23.5.

**HRMS** (ESI) for C_33_H_43_IN_2_O_4_SNa: calculated 691.2061 [M+Na]^+^, found 691.2071.[α]^20^_D_ = -12.3° (CHCl_3_, *c* = 1).

**((3a*S*,4*R*,6*R*,7a*S*)-5,5-dimethyl-2-((4-neopentylphenyl)sulfonyl)octahydro-3a*H*-4,6-methanoisoindol-3a-yl)methyl (4-iodophenyl)carbamate (74):**

Following general procedure E2: From **S25** (60.84 mg, 0.15 mmol) and 1-iodo-4-isocyanatobenzene (45 mg, 0.18 mmol, 1.2 eq.). Purification by column chromatography (PE/EtOAc 20:1 to 10:1, v/v) afforded a colorless solid **74** (R*_f_*= 0.7, PE:EA=3:1, v/v; 80 mg, 0.12 mmol, 82%).

**^1^H NMR** (500 MHz, CDCl_3_) δ 7.71 (d, *J =* 8.2 Hz, 2H), 7.60 (d, *J =* 8.7 Hz, 2H), 7.26 (d, *J =* 8.2 Hz, 2H), 7.10 (d, *J =* 8.2 Hz, 2H), 6.37 (s, 1H), 4.07 (d, *J =* 10.7 Hz, 1H), 3.99 (d, *J =* 10.6 Hz, 1H), 3.18 (dd, *J =* 9.2, 6.5 Hz, 1H), 3.10 – 2.99 (m, 3H), 2.45 (s, 2H), 2.33 – 2.27 (m, 2H), 2.23 – 2.16 (m, 1H), 1.92 (dd, *J =* 6.5, 5.0 Hz, 1H), 1.91 – 1.86 (m, 1H), 1.64 – 1.58 (m, 4H), 1.30 (d, *J =* 10.4 Hz, 1H), 1.22 (s, 3H), 1.00 (s, 3H), 0.87 (s, 9H).

**^13^C NMR** (126 MHz, CDCl_3_) δ 153.0, 145.7, 138.1, 137.6, 132.4, 130.9, 127.7, 120.6, 86.6, 70.3, 58.4, 57.0, 50.7, 50.1, 46.6, 40.2, 39.1, 34.5, 34.2, 32.1, 29.4, 27.4, 27.1, 23.6.

**HRMS** (ESI) for C_30_H_40_IN_2_O_4_S: calculated 651.1748 [M+H]^+^, found 651.1759.[α]^20^_D_ = -4.3° (CHCl_3_, *c* = 0.5).

**((3a*S*,4*R*,6*R*,7a*S*)-5,5-dimethyl-2-((4-(*tert*-pentyl)phenyl)sulfonyl)octahydro-3a*H*-4,6-methanoisoindol-3a-yl)methyl (4-iodophenyl)carbamate (75):**

Following general procedure E2: From **S26** (60.8 mg, 0.15 mmol) and 1-iodo-4-isocyanatobenzene (45 mg, 1.2 eq.). Purification by column chromatography (PE/EtOAc 20:1 to 10:1, v/v) afforded a colorless solid **75** (R*_f_*= 0.7, PE:EA=3:1, v/v; 62.2 mg, 0.1 mmol, 64%).

**^1^H NMR** (500 MHz, CDCl_3_)δ 7.74 (d, *J =* 8.5 Hz, 2H), 7.59 (d, *J =* 8.9 Hz, 2H), 7.48 (d, *J =* 8.5 Hz, 2H), 7.13 (d, *J =* 8.3 Hz, 2H), 6.53 (s, 1H), 4.09 (d, *J =* 10.7 Hz, 1H), 4.00 (d, *J =* 10.6 Hz, 1H), 3.25 (dd, *J =* 9.2, 6.8 Hz, 1H), 3.10 – 3.03 (m, 2H), 3.03 – 2.98 (m, 1H), 2.28 (qdt, *J =* 10.4, 7.0, 3.0 Hz, 2H), 2.22 – 2.14 (m, 1H), 1.94 (dd, *J =* 6.4, 4.9 Hz, 1H), 1.87 (dt, *J =* 5.5, 2.7 Hz, 1H), 1.63 (q, *J =* 7.6 Hz, 2H), 1.59 (d, *J =* 3.0 Hz, 1H), 1.29 – 1.28 (m, 6H), 1.26 (m, 1H), 1.22 (s, 3H), 1.00 (s, 3H), 0.62 (t, *J =* 7.4 Hz, 3H).

**^13^C NMR** (126 MHz, CDCl_3_) δ 155.5, 153.2, 138.1, 137.6, 132.1, 128.0, 126.7, 120.7, 93.1, 70.2, 58.3, 57.0, 50.6, 46.3, 40.1, 39.1, 38.6, 37.0, 34.4, 34.0, 28.3, 28.2, 27.4, 27.0, 23.5, 9.2.

**HRMS** (ESI) for C_30_H_40_IN_2_O_4_S: calculated 651.1748 [M+H]^+^, found 651.1757.[α]^20^_D_ = -3.3° (CHCl_3_, *c* = 1.0).

**((3a*S*,4*R*,6*R*,7a*S*)-2-((4'-(*tert*-butyl)-[1,1'-biphenyl]-4-yl)sulfonyl)-5,5-dimethyloctahydro-3a*H*-4,6-methanoisoindol-3a-yl)methyl (4-iodophenyl)carbamate (76):**

Following general procedure E2: From **S27** (70.15 mg, 0.15 mmol) and 1-iodo-4-isocyanatobenzene (45 mg, 0.18 mmol, 1.2 eq.). Purification by column chromatography (PE/EtOAc 20:1 to 10:1, v/v) afforded a colorless solid **76** (R*_f_*= 0.7, PE:EA=3:1, v/v; 95.7 mg, 0.13 mmol, 90%).

**^1^H NMR** (500 MHz, CDCl_3_) δ 7.87 (d, *J =* 8.5 Hz, 2H), 7.75 (d, *J =* 8.5 Hz, 2H), 7.53 – 7.45 (m, 4H), 7.39 (d, *J =* 8.6 Hz, 2H), 6.90 (d, *J =* 8.7 Hz, 2H), 6.06 (s, 1H), 4.08 (d, *J =* 10.7 Hz, 1H), 3.94 (d, *J =* 10.7 Hz, 1H), 3.16 (d, *J =* 9.9 Hz, 2H), 3.15 – 3.07 (m, 2H), 3.02 (d, *J =* 9.8 Hz, 1H), 2.39 – 2.29 (m, 2H), 2.29 – 2.20 (m, 1H), 1.91 (d, *J =* 6.2 Hz, 2H), 1.72 – 1.62 (m, 1H), 1.43 (d, *J =* 10.5 Hz, 1H), 1.39 (s, 9H), 1.23 (s, 3H), 1.00 (s, 3H).

**^13^C NMR** (126 MHz, CDCl_3_) δ 152.2, 151.1, 145.6, 137.9, 137.3, 135.9, 132.2, 129.0, 127.2, 127.1, 126.3, 120.7, 99.7, 70.6, 58.6, 56.9, 50.7, 47.0, 40.2, 39.1, 34.9, 34.8, 34.6, 31.5, 27.4, 27.3, 23.6.

**HRMS** (ESI) for C_35_H_42_IN_2_O_4_S: calculated 735.1729 [M+H]^+^, found 735.1726.[α]^20^_D_ = +13.3° (CHCl_3_, *c* = 1.0).

**((3a*S*,4*R*,6*R*,7a*S*)-5,5-dimethyl-2-((4-(trimethylsilyl)phenyl)sulfonyl)octahydro-3a*H*-4,6-methanoisoindol-3a-yl)methyl (4-iodophenyl)carbamate (77)：**

Following general procedure E2: From **S28** (61.15 mg, 0.15 mmol) and 1-iodo-4-isocyanatobenzene (73.5 mg, 1.2 eq.). Purification by column chromatography (PE/EtOAc 20:1 to 10:1, v/v) afforded a colorless solid **77** (R*_f_*= 0.8, PE:EA=3:1, v/v; 63 mg, 0.10 mmol, 64%).

**^1^H NMR** (700 MHz, CD_2_Cl_2_) δ 7.75 (d, *J =* 6.7 Hz, 2H), 7.70 (d, *J =* 6.6 Hz, 2H), 7.62 – 7.58 (m, 2H), 7.14 (d, *J =* 8.2 Hz, 2H), 6.53 (d, *J =* 6.9 Hz, 1H), 4.10 (d, *J =* 10.6 Hz, 1H), 3.93 (d, *J =* 10.6 Hz, 1H), 3.20 (t, *J =* 8.3 Hz, 1H), 3.08 – 2.98 (m, 3H), 2.35 – 2.29 (m, 1H), 2.25 (tt, *J =* 7.0, 3.7 Hz, 1H), 2.20 (dt, *J =* 11.4, 6.4 Hz, 1H), 1.98 (t, *J =* 5.8 Hz, 1H), 1.88 (q, *J =* 5.0, 4.3 Hz, 1H), 1.60 (d, *J =* 13.4 Hz, 1H), 1.29 (d, *J =* 10.4 Hz, 1H), 1.22 (s, 3H), 1.00 (s, 3H), 0.27 (s, 9H).

**^13^C NMR** (176 MHz, CD_2_Cl_2_) δ 153.4, 147.7 138.3 138.2, 135.6, 134.2, 127.2, 120.9, 86.5, 70.4, 58.5, 57.3, 50.9, 46.6, 40.6, 39.4, 34.7, 34.4, 27.4, 27.3, 23.5, -1.4.

**HRMS** (ESI) for C_28_H_37_IN_2_O_4_SSiNa: calculated 675.1186 [M+Na]^+^, found 675.1176.[α]^20^_D_ = -2.3° (CHCl_3_, *c* = 1.0).

**((3a*S*,4*R*,6*R*,7a*S*)-2-((4-(adamantan-1-yl)phenyl)sulfonyl)-5,5-dimethyloctahydro-3a*H*-4,6-methanoisoindol-3a-yl)methyl (4-iodophenyl)carbamate (78):**

Following general procedure E2: From **S29** (47 mg, 0.1 mmol) and 1-iodo-4-isocyanatobenzene (29.4 mg, 1.2 eq.). Purification by column chromatography (PE/EtOAc 20:1 to 10:1, v/v) afforded a colorless solid **78** (R*_f_*= 0.7, PE:EA=3:1, v/v; 68%).

**^1^H NMR** (700 MHz, CDCl_3_) δ 7.74 (d, *J =* 8.6 Hz, 2H), 7.59 (d, *J =* 8.7 Hz, 2H), 7.50 (d, *J =* 8.6 Hz, 2H), 7.11 (d, *J =* 7.8 Hz, 2H), 6.35 (s, 1H), 4.06 (d, *J =* 10.7 Hz, 1H), 3.98 (d, *J =* 10.7 Hz, 1H), 3.18 (dd, *J =* 9.2, 6.8 Hz, 1H), 3.09 (d, *J =* 9.8 Hz, 1H), 3.06 (dd, *J =* 9.3, 2.9 Hz, 1H), 3.01 (d, *J =* 9.8 Hz, 1H), 2.34 – 2.25 (m, 2H), 2.21 (dtd, *J =* 10.5, 6.3, 1.8 Hz, 1H), 2.08 (s, 3H), 1.93 (dd, *J =* 6.5, 4.9 Hz, 1H), 1.89 (tt, *J =* 5.4, 2.9 Hz, 1H), 1.85 (s, 6H), 1.81 – 1.75 (m, 3H), 1.73 – 1.68 (m, 3H), 1.64 (dt, *J =* 12.3, 3.3 Hz, 1H), 1.37 (d, *J =* 10.5 Hz, 1H), 1.23 (s, 3H), 1.00 (s, 3H).

**^13^C NMR** (176 MHz, CDCl_3_) δ 157.0, 153.1, 138.1, 137.5, 132.2, 128.1, 125.7, 120.8, 86.7, 70.4, 58.3, 57.0, 50.6, 46.6, 43.0, 40.2, 39.2, 36.9, 36.7, 34.6, 34.2, 28.8, 27.4, 27.2, 23.5.

**HRMS** (ESI) for C_35_H_43_IN_2_O_4_SNa: calculated 737.1886 [M+Na]^+^, found 737.1890.[α]^20^_D_ = -6.3° (CHCl_3_, *c* = 1.0).

**((3a*S*,4*R*,6*R*,7a*S*)-2-((4-(*tert*-butyl)-2-methylphenyl)sulfonyl)-5,5-dimethyloctahydro-3a*H*-4,6-methanoisoindol-3a-yl)methyl (4-iodophenyl)carbamate (79):**

Following general procedure E2: From **S30** (60.84 mg, 0.15 mmol) and 1-iodo-4-isocyanatobenzene (45 mg, 0.18 mmol, 1.2 eq.). Purification by column chromatography (PE/EtOAc 20:1 to 10:1, v/v) afforded a colorless solid **79** (R*_f_*= 0.8, PE:EA=3:1, v/v; 69.6 mg, 0.11 mmol, 71%).

**^1^H NMR** (500 MHz, CD_2_Cl_2_) δ 7.85 (d, *J =* 8.7 Hz, 1H), 7.60 (d, *J =* 8.8 Hz, 2H), 7.33 – 7.28 (m, 2H), 7.18 (d, *J =* 8.4 Hz, 2H), 6.90 (s, 1H), 4.18 (d, *J =* 10.7 Hz, 1H), 4.05 (d, *J =* 10.6 Hz, 1H), 3.48 (t, *J =* 8.4 Hz, 1H), 3.37 (d, *J =* 10.0 Hz, 1H), 3.04 (d, *J =* 10.0 Hz, 1H), 3.00 (dd, *J =* 9.5, 3.5 Hz, 1H), 2.66 (s, 3H), 2.35 (dt, *J =* 7.3, 3.5 Hz, 1H), 2.34 – 2.26 (m, 1H), 2.21 (dtd, *J =* 10.4, 6.3, 1.9 Hz, 1H), 1.95 – 1.87 (m, 2H), 1.57 – 1.53 (m, 1H), 1.31 (s, 9H), 1.29 (d, *J =* 8.5 Hz, 1H), 1.23 (s, 3H), 1.03 (s, 3H).

**^13^C NMR** (176 MHz, CD_2_Cl_2_) δ 157.1, 153.5, 138.4, 138.3, 138.3, 133.0, 130.4, 130.3, 123.5, 120.8, 86.4, 70.6, 58.0, 56.6, 51.0, 46.7, 40.6, 39.3, 35.2, 34.8, 34.4, 31.1, 27.4, 27.4, 23.6, 21.2.

**HRMS** (ESI) for C_30_H_39_IN_2_O_4_SNa: calculated 651.1748 [M+Na]^+^, found 651.1752.

[α]^20^_D_ = +10.7° (CHCl_3_, *c* = 0.5).

**((3a*S*,4*R*,6*R*,7a*S*)-2-((4-(tert-butyl)-2-ethylphenyl)sulfonyl)-5,5-dimethyloctahydro-3a*H*-4,6-methanoisoindol-3a-yl)methyl (4-iodophenyl)carbamate (80):**

Following general procedure E2: From **S31** (42 mg, 0.1 mmol) and 1-iodo-4-isocyanatobenzene (29.4 mg, 0.12 mmol, 1.2 eq.). Purification by column chromatography (PE/EtOAc 20:1 to 10:1, v/v) afforded a colorless solid **80** (R*_f_*= 0.7, PE:EA=3:1, v/v; 78%).

**^1^H NMR** (500 MHz, CD_2_Cl_2_)δ 7.80 (d, *J =* 8.4 Hz, 1H), 7.61 (d, *J =* 8.7 Hz, 2H), 7.40 (d, *J =* 2.1 Hz, 1H), 7.33 (dd, *J =* 8.4, 2.1 Hz, 1H), 7.19 (d, *J =* 8.4 Hz, 2H), 6.87 (s, 1H), 4.09 (d, *J =* 2.1 Hz, 2H), 3.41 (dd, *J =* 9.4, 6.7 Hz, 1H), 3.26 (d, *J =* 10.0 Hz, 1H), 3.08 – 3.01 (m, 2H), 3.01 – 2.96 (m, 2H), 2.30 (dt, *J =* 9.5, 2.9 Hz, 2H), 2.20 (dtd, *J =* 10.4, 6.2, 1.5 Hz, 1H), 1.98 – 1.93 (m, 1H), 1.91 – 1.86 (m, 1H), 1.55 (d, *J =* 2.7 Hz, 1H), 1.31 (s, 8H), 1.29 (s, 3H), 1.31 – 1.24 (m, 4H), 1.22 (s, 3H), 1.02 (s, 3H).

**^13^C NMR** (126 MHz, CD_2_Cl_2_) δ 157.2, 153.5, 144.6 138.4, 138.3, 132.5, 130.5, 128.6, 123.4, 120.9, 86.6, 70.6, 58.1, 56.6, 51.0, 46.7, 40.6, 39.3, 35.3, 34.9, 34.3, 31.2, 27.4, 27.3, 27.00, 23.6, 16.3.

**HRMS** (ESI) for C_31_H_41_IN_2_O_4_SNa: calculated 687.1729 [M+Na]^+^, found 687.1727.[α]^20^_D_ = -8.3° (CHCl_3_, *c* = 1.0).

**((3a*S*,4*R*,6*R*,7a*S*)-2-((4-(*tert*-butyl)-2-methoxyphenyl)sulfonyl)-5,5-dimethyloctahydro-3a*H*-4,6-methanoisoindol-3a-yl)methyl (4-iodophenyl)carbamate (82):**

Following general procedure E2: From **S32** (39.4 mg, 0.1 mmol) and 1-iodo-4-isocyanatobenzene (29.4 mg, 1.2 eq.). Purification by column chromatography (PE/EtOAc 20:1 to 10:1, v/v) afforded a colorless solid **82** (R*_f_*= 0.6, PE:EA=3:1, v/v; 60%).

**^1^H NMR** (500 MHz, CDCl_3_) δ 7.81 (d, *J =* 8.2 Hz, 1H), 7.62 – 7.57 (m, 2H), 7.15 (d, *J =* 8.3 Hz, 2H), 7.04 (dd, *J =* 8.3, 1.7 Hz, 1H), 6.98 (d, *J =* 1.8 Hz, 1H), 6.71 (s, 1H), 4.11 (s, 2H), 3.91 (s, 3H), 3.55 (dd, *J =* 9.8, 6.3 Hz, 1H), 3.35 (d, *J =* 10.2 Hz, 1H), 3.18 (d, *J =* 10.2 Hz, 1H), 3.15 (dd, *J =* 10.0, 2.9 Hz, 1H), 2.34 – 2.26 (m, 2H), 2.21 (dtd, *J =* 10.4, 6.1, 1.5 Hz, 1H), 1.96 (dd, *J =* 6.5, 5.0 Hz, 1H), 1.92 – 1.88 (m, 1H), 1.62 (dd, *J =* 9.0, 2.8 Hz, 1H), 1.39 (d, *J =* 10.5 Hz, 1H), 1.32 (s, 9H), 1.23 (s, 3H), 1.03 (s, 3H).

**^13^C NMR** (126 MHz, CDCl_3_) δ 159.1, 157.1, 153.4, 138.1, 137.8, 131.5, 123.6, 120.7, 117.8, 109.8, 86.5, 70.6, 58.1, 56.6, 56.1, 50.8, 46.5, 40.3, 39.1, 35.6, 34.8, 34.2, 31.2, 27.5, 27.0, 23.6.

**HRMS** (ESI) for C_30_H_39_IN_2_O_5_SNa: calculated 689.1522 [M+Na]^+^, found 689.1537.[α]^20^_D_ = -12.1° (CHCl_3_, *c* = 0.5).

**((3a*S*,4*R*,6*R*,7a*S*)-2-((2-bromo-4-(*tert*-butyl)phenyl)sulfonyl)-5,5-dimethyloctahydro-3a*H*-4,6-methanoisoindol-3a-yl)methyl (4-iodophenyl)carbamate (83):**

Following general procedure E2: From **S33** (47 mg, 0.1 mmol) and 1-iodo-4-isocyanatobenzene (29.4 mg, 1.2 eq.). Purification by column chromatography (PE/EtOAc 20:1 to 10:1, v/v) afforded a colorless solid **83** (R*_f_*= 0.7, PE:EA=3:1, v/v; 47 mg, 0.07 mmol, 66%).

**^1^H NMR** (600 MHz, CDCl_3_)δ 8.02 (d, *J =* 8.3 Hz, 1H), 7.72 (d, *J =* 1.9 Hz, 1H), 7.59 (d, *J =* 8.8 Hz, 2H), 7.42 (dd, *J =* 8.3, 1.9 Hz, 1H), 7.18 (d, *J =* 8.2 Hz, 2H), 6.97 (s, 1H), 4.24 (d, *J =* 10.6 Hz, 1H), 4.05 (d, *J =* 10.6 Hz, 1H), 3.76 – 3.66 (m, 1H), 3.49 (d, *J =* 10.3 Hz, 1H), 3.19 (d, *J =* 10.3 Hz, 1H), 3.14 (dd, *J =* 10.0, 4.4 Hz, 1H), 2.41 (dd, *J =* 11.9, 6.5 Hz, 1H), 2.29 (dt, *J =* 13.5, 2.5 Hz, 1H), 2.26 – 2.19 (m, 1H), 1.92 (dd, *J =* 6.2, 2.6 Hz, 2H), 1.64 (ddd, *J =* 13.5, 4.1, 2.5 Hz, 1H), 1.36 (d, *J =* 10.5 Hz, 1H), 1.31 (s, 10H), 1.24 (s, 3H), 1.03 (s, 3H).

**^13^C NMR** (151 MHz, CDCl_3_) δ 158.2, 153.4, 138.1, 137.9, 135.0, 133.0, 132.2, 124.8, 120.7, 120.7, 86.4, 70.8, 58.5, 56.8, 51.2, 46.5, 40.3, 39.0, 35.3, 34.8, 33.7, 31.0, 27.4, 27.3, 23.7.

**HRMS** (ESI) for C_29_H_36_BrIN_2_O_4_SNa: calculated 737.0522 [M+Na]^+^, found 737.0516.

[α]^20^_D_ = -36.1° (CHCl_3_, *c* = 0.5).

**((3a*S*,4*R*,6*R*,7a*S*)-2-((4-(tert-butyl)-2-iodophenyl)sulfonyl)-5,5-dimethyloctahydro-3aH-4,6-methanoisoindol-3a-yl)methyl (4-iodophenyl)carbamate (84):**

Following general procedure E2: From **S34** (51.75 mg, 0.1 mmol) and 1-iodo-4-isocyanatobenzene (29.4 mg, 1.2 eq.). Purification by column chromatography (PE/EtOAc 20:1 to 10:1, v/v) afforded a colorless solid **84** (R*_f_*= 0.7, PE:EA=3:1, v/v; 32 mg, 42 %).

**^1^H NMR** (600 MHz, CDCl_3_) δ 8.07 (d, *J =* 1.9 Hz, 1H), 8.04 (d, *J =* 8.3 Hz, 1H), 7.60 (d, *J =* 8.8 Hz, 2H), 7.47 (dd, *J =* 8.3, 1.9 Hz, 1H), 7.19 (d, *J =* 8.2 Hz, 2H), 6.99 (s, 1H), 4.28 (d, *J =* 10.5 Hz, 1H), 4.05 (d, *J =* 10.5 Hz, 1H), 3.70 (t, *J =* 8.9 Hz, 1H), 3.54 (d, *J =* 10.3 Hz, 1H), 3.20 (d, *J =* 10.4 Hz, 1H), 3.11 (dd, *J =* 10.0, 4.7 Hz, 1H), 2.43 (d, *J =* 9.9 Hz, 1H), 2.27 (t, *J =* 10.8 Hz, 1H), 2.25 (dd, *J =* 8.3, 2.6 Hz, 1H), 1.93 (d, *J =* 6.1 Hz, 2H), 1.71 – 1.65 (m, 1H), 1.40 (d, *J =* 10.6 Hz, 1H), 1.31 (s, 9H), 1.24 (s, 3H), 1.04 (s, 3H).

**^13^C NMR** (151 MHz, CDCl_3_) δ 157.9, 153.5, 140.5, 138.1, 138.1, 138.0, 131.7, 125.6, 120.7, 93.3, 86.4, 71.0, 58.6, 57.2, 51.4, 46.6, 40.5, 39.0, 35.1, 34.9, 33.7, 31.1, 27.6, 27.5, 23.8.

**HRMS** (ESI) for C_29_H_36_I_2_N_2_O_4_SNa: calculated 785.0383 [M+Na]^+^, found 785.0385.

[α]^20^_D_ = -80.0° (CHCl_3_, *c* = 0.5).

**((3a*S*,4*R*,6*R*,7a*S*)-2-((4-(1-(2-methoxyethoxy)-2-methylpropan-2-yl)phenyl)sulfonyl)-5,5-dimethyloctahydro-3a*H*-4,6-methanoisoindol-3a-yl)methyl (4-iodophenyl)carbamate (85):**

Following general procedure E2: From **S35** (46.57 mg, 0.1 mmol) and 1-iodo-4-isocyanatobenzene (29.4 mg, 1.2 eq.). Purification by column chromatography (PE/EtOAc 20:1 to 10:1, v/v) afforded a colorless solid **85** (R*_f_*= 0.6, PE:EA=3:1, v/v; 54.9 mg, 0.075 mmol, 75%).

**^1^H NMR** (700 MHz, CDCl_3_) δ 7.71 (d, *J =* 8.1 Hz, 2H), 7.60 (d, *J =* 7.2 Hz, 2H), 7.58 – 7.55 (m, 2H), 7.15 (d, *J =* 8.2 Hz, 2H), 6.67 (s, 1H), 4.07 (d, *J =* 10.8 Hz, 1H), 3.98 (d, *J =* 10.7 Hz, 1H), 3.52 (d, *J =* 9.2 Hz, 1H), 3.50 – 3.46 (m, 3H), 3.42 (t, *J =* 4.8 Hz, 2H), 3.26 (d, *J =* 1.4 Hz, 3H), 3.15 (t, *J =* 8.2 Hz, 1H), 3.04 (d, *J =* 9.9 Hz, 1H), 3.00 (dd, *J =* 9.5, 3.6 Hz, 2H), 2.32 (t, *J =* 11.9 Hz, 1H), 2.28 (dd, *J =* 7.3, 3.7 Hz, 1H), 2.20 (dt, *J =* 11.6, 6.5 Hz, 1H), 1.96 (t, *J =* 5.8 Hz, 1H), 1.87 (d, *J =* 5.7 Hz, 1H), 1.62 – 1.59 (m, 1H), 1.33 (s, 1H), 1.31 (s, 3H), 1.30 (s, 3H), 1.22 (s, 3H), 1.01 (s, 3H).

**^13^C NMR** (176 MHz, CDCl_3_) δ 153.9, 153.5, 138.3, 138.3, 132.7, 128.1, 127.3, 121.1, 86.4, 80.9, 72.2, 71.3, 70.3, 59.0, 58.6, 57.2, 51.0, 46.8, 40.6, 40.0, 39.4, 34.7, 34.5, 27.4, 27.3, 26.1, 23.5.

**HRMS** (ESI) for C_32_H_43_IN_2_O_6_SNa: calculated: 733.1784 [M+Na]^+^, found 733.1777.

[α]^20^_D_ = +12.0° (CHCl_3_, *c* = 0.5).

**((3a*S*,4*R*,6*R*,7a*S*)-5,5-dimethyl-2-((4-(2-methyl-1-morpholino-1-oxopropan-2-yl)phenyl)sulfonyl)octahydro-3a*H*-4,6-methanoisoindol-3a-yl)methyl (4-iodophenyl)carbamate (86):**

Following general procedure E2: From **S36** (73.6 mg, 0.15 mmol) and 1-iodo-4-isocyanatobenzene (45 mg, 0.18 mmol, 1.2 eq.). Purification by column chromatography (PE/EtOAc 20:1 to 10:1, v/v) afforded a colorless solid **86** (R*_f_*= 0.6, PE:EA=3:1, v/v; 73.5 mg, 67%).

**^1^H NMR** (400 MHz, CD_2_Cl_2_) δ 7.77 (d, *J =* 8.5 Hz, 2H), 7.61 (d, *J =* 8.8 Hz, 2H), 7.42 (d, *J =* 8.5 Hz, 2H), 7.16 (d, *J =* 8.4 Hz, 2H), 6.74 (s, 1H), 4.16 (d, *J =* 10.8 Hz, 1H), 3.89 (d, *J =* 10.7 Hz, 1H), 3.64 (d, *J =* 3.1 Hz, 1H), 3.35 (s, 4H), 3.24 (dd, *J =* 9.4, 7.2 Hz, 2H), 2.99 (d, *J =* 1.9 Hz, 3H), 2.33 – 2.27 (m, 1H), 2.24 (dq, *J =* 6.9, 3.5 Hz, 1H), 2.18 (dt, *J =* 10.6, 5.9 Hz, 1H), 2.03 – 1.98 (m, 1H), 1.88 (q, *J =* 3.2, 2.8 Hz, 1H), 1.61 – 1.58 (m, 1H), 1.51 (s, 6H), 1.25 (d, *J =* 13.3 Hz, 1H), 1.22 (s, 3H), 1.01 (s, 3H).

**^13^C NMR** (101 MHz, CD_2_Cl_2_) δ 173.9, 156.8, 152.2, 139.3, 138.3, 133.6, 129.1, 126.1, 120.9, 85.7, 70.1, 58.4, 57.4, 50.9, 47.4, 46.4, 40.5, 39.4, 34.6, 34.2, 28.4, 28.2, 27.4, 27.2, 23.4.

**HRMS** (ESI) for C_33_H_42_IN_3_O_6_SNa: calculated: 758.1737 [M+Na]^+^, found 758.1735.

[α]^20^_D_ = -4.0° (CHCl_3_, *c* = 0.5).

**((3a*S*,4*R*,6*R*,7a*S*)-2-((4-(1-(dimethylamino)-2-methyl-1-oxopropan-2-yl)phenyl)sulfonyl)-5,5-dimethyloctahydro-3aH-4,6-methanoisoindol-3a-yl)methyl (4-iodophenyl)carbamate (87)**

Following general procedure E2: From **S37** (44.9 mg, 0.1 mmol) and 1-iodo-4-isocyanatobenzene (29.4 mg, 0.12 mmol, 1.2 eq.). Purification by column chromatography (PE/EtOAc 20:1 to 10:1, v/v) afforded a colorless solid **87** (R*_f_*= 0.6, PE:EA=3:1, v/v; 58.9 mg, 0.09 mmol, 85%).

**^1^H NMR** (700 MHz, CDCl_3_)δ 7.78 (d, *J =* 8.5 Hz, 2H), 7.60 (d, *J =* 8.8 Hz, 2H), 7.39 (d, *J =* 8.5 Hz, 2H), 7.14 (d, *J =* 8.2 Hz, 2H), 6.59 (s, 1H), 4.13 (d, *J =* 10.6 Hz, 1H), 3.96 (d, *J =* 10.6 Hz, 1H), 3.29 (dd, *J =* 9.4, 7.1 Hz, 1H), 3.08 (d, *J =* 9.9 Hz, 1H), 3.02 (d, *J =* 9.9 Hz, 1H), 3.00 – 2.98 (m, 1H), 2.92 (s, 3H), 2.42 (s, 3H), 2.33 – 2.28 (m, 1H), 2.26 (ddt, *J =* 10.5, 6.9, 3.7 Hz, 1H), 2.19 – 2.14 (m, 1H), 1.97 (dd, *J =* 6.5, 4.9 Hz, 1H), 1.88 (dq, *J =* 5.4, 2.6 Hz, 1H), 1.59 (dt, *J =* 12.9, 3.4 Hz, 1H), 1.54 (s, 3H), 1.53 (s, 3H), 1.24 (d, *J =* 17.2 Hz, 1H), 1.22 (s, 3H), 1.01 (s, 3H).

**^13^C NMR** (176 MHz, CDCl_3_) δ 175.0, 153.2, 152.3, 138.1, 137.6, 133.4, 128.9, 125.6, 120.6, 86.7, 70.1, 58.3, 57.1, 50.7, 47.4, 46.2, 40.1, 39.1, 38.2, 37.3, 34.4, 33.9, 28.4, 28.0, 27.4, 27.0, 23.5.

**HRMS** (ESI) for C_31_H_40_IN_3_O_5_SNa: calculated: 716.1631 [M+Na]^+^, found 716.1630.

[α]^20^_D_ = -8.1° (CHCl_3_, *c* = 0.5).

**((3a*S*,4*R*,6*R*,7a*S*)-2-((4-(1-(dimethylamino)-2-methylpropan-2-yl)phenyl)sulfonyl)-5,5-dimethyloctahydro-3a*H*-4,6-methanoisoindol-3a-yl)methyl (4-iodophenyl)carbamate (88):**

A solution of **S37** (89.7 mg, 0.2 mmol) in THF (3 mL) was treated dropwise with lithium aluminum hydride (8 mg) while the mixture was stirred under a nitrogen atmosphere. The reaction was stirred for 5 h and then quenched with water (1 mL). The reaction was filtered through celite, and the filtrate was evaporated. The residue was partitioned between 2 N aqueous NaOH and ethyl acetate. The organic phase was dried (Na_2_SO_4_), evaporated, and afforded crude oil, which was used in next step without further purification (54 mg, 0.12 mmol, 62%).

Following general procedure E2: From the crude oil obtained from above step (52.16 mg, 0.12 mmol) and 1-iodo-4-isocyanatobenzene (35.3 mg, 1.2 eq.). Purification by column chromatography (PE/EtOAc 15:1 to 10:1 to 5:1, v/v) afforded a colorless solid **88** (R*_f_*= 0.5, PE:EA=3:1, v/v; 62 mg, 0.09 mmol, 76%).

**^1^H NMR** (500 MHz, CDCl_3_) δ 7.73 (d, *J =* 8.5 Hz, 2H), 7.60 (d, *J =* 8.7 Hz, 2H), 7.56 (d, *J =* 8.5 Hz, 2H), 7.14 (d, *J =* 8.2 Hz, 2H), 6.54 (s, 1H), 4.08 (d, *J =* 10.7 Hz, 1H), 3.99 (d, *J =* 10.7 Hz, 1H), 3.26 – 3.19 (m, 1H), 3.08 – 3.01 (m, 2H), 3.01 – 2.96 (m, 1H), 2.46 (d, *J =* 6.7 Hz, 2H), 2.32 – 2.23 (m, 2H), 2.16 (ddt, *J =* 7.8, 6.2, 3.2 Hz, 1H), 2.00 (s, 6H), 1.93 (dd, *J =* 6.4, 4.9 Hz, 1H), 1.87 (dd, *J =* 5.5, 3.2 Hz, 1H), 1.60 – 1.56 (m, 1H), 1.33 (s, 3H), 1.32 (s, 3H), 1.28 (d, *J =* 4.8 Hz, 1H), 1.21 (s, 3H), 1.01 (s, 3H).

**^13^C NMR** (126 MHz, CDCl_3_) δ 154.7, 153.2, 138.1, 137.7, 132.2, 127.9, 127.0, 120.8, 86.7, 72.6, 70.2, 58.3, 57.0, 50.7, 48.3, 46.4, 40.2, 40.0, 39.1, 34.4, 34.0, 27.4, 27.3, 27.1, 27.0, 23.5.

**HRMS** (ESI) for C_31_H_42_IN_3_O_4_SNa: calculated: 680.2013 [M+Na]^+^, found 680.2012.

[α]^20^_D_ = 16.0° (CHCl_3_, *c* = 0.5).

**Ethyl 2-(4-(((3a*S*,4*R*,6*R*,7a*S*)-3a-((((4-iodophenyl)carbamoyl)oxy)methyl)-5,5-dimethyloctahydro-2*H*-4,6-methanoisoindol-2-yl)sulfonyl)phenyl)-2-methylpropanoate (89):**

Following general procedure E2: From **S37** (13.5 mg, 0.03 mmol) and 1-iodo-4-isocyanatobenzene (21.7 mg, 0.06 mmol, 2.0 eq.). Purification by column chromatography (PE/EtOAc 20:1 to 10:1, v/v) afforded a colorless solid **87** (R*_f_*= 0.6, PE:EA=3:1, v/v; 58.9 mg, 0.09 mmol, 85%).

**^1^H NMR** (500 MHz, CDCl_3_)δ 7.77 (d, *J =* 8.5 Hz, 2H), 7.59 (d, *J =* 8.8 Hz, 2H), 7.51 (d, *J =* 8.6 Hz, 2H), 7.13 (d, *J =* 8.3 Hz, 2H), 6.58 (s, 1H), 4.16 – 4.06 (m, 3H), 3.99 (d, *J =* 10.9 Hz, 1H), 3.23 – 3.12 (m, 1H), 3.09 – 3.02 (m, 3H), 2.36 – 2.26 (m, 2H), 2.20 (dtd, *J =* 10.6, 6.4, 1.5 Hz, 1H), 1.92 (dd, *J =* 6.5, 5.0 Hz, 1H), 1.89 (dq, *J =* 5.4, 2.6 Hz, 1H), 1.60 (s, 1H), 1.59 (s, 3H), 1.59 (s, 3H), 1.33 (d, *J =* 10.5 Hz, 1H), 1.22 (s, 3H), 1.17 (t, *J =* 7.1 Hz, 3H), 1.02 (s, 3H).

**^13^C NMR** (126 MHz, CDCl_3_) δ 176.0, 153.2, 150.1, 137.9, 137.5, 133.4, 128.1, 126.5, 120.9, 86.6, 70.1, 61.3, 58.2, 56.8, 50.7, 46.8, 46.5, 40.1, 39.0, 34.2, 34.1, 27.3, 27.0, 26.4, 26.2, 23.4, 14.0.

**HRMS** (ESI) for C_31_H_39_IN_2_O_6_SNa: calculated 695.1657 [M+Na]^+^, found 695.1646.

[α]^20^_D_ = +8.1° (CHCl_3_, *c* = 1.0).

**2-(4-(((3a*S*,4*R*,6*R*,7a*S*)-3a-((((4-iodophenyl)carbamoyl)oxy)methyl)-5,5-dimethyloctahydro-2*H*-4,6-methanoisoindol-2-yl)sulfonyl)phenyl)-2-methylpropanoic acid (92):**

The solution of sodium hydroxide (32 mg, 0.24 mmol, 3 eq.) in ethanol (0.1 M) was added dropwise to a solution of **S37** (0.24 mmol, 1.0 eq.) in ethanol (0.1 M) at ice bath. The resulting mixture was refluxed for 2-6 h, and then the solution was acidified with 2 N HCl to pH = 2. Extraction of the resulting mixture with diethyl ether, drying of the combined organic extracts over magnesium sulfate and evaporation of the solvent afforded carboxylic acid without purification for next step.

Following general procedure E3: From the crude carboxylic acid (63.2 mg, 0.15 mmol) and 1-iodo-4-isocyanatobenzene (44.1 mg, 0.18 mmol, 1.2 eq). Purification by column chromatography (DCM:MeOH=50:1 to 20:1, v/v) afforded a colorless solid **92** (R*_f_*= 0.3, DCM:MeOH=20:1, v/v; 45.6 mg, 0.07 mmol, 46% for two steps).

**^1^H NMR** (400 MHz, CD_2_Cl_2_) δ 7.76 (d, *J =* 8.5 Hz, 2H), 7.60 (d, *J =* 8.8 Hz, 2H), 7.57 (d, *J =* 8.5 Hz, 2H), 7.14 (d, *J =* 8.3 Hz, 2H), 6.69 (s, 1H), 4.06 (d, *J =* 10.8 Hz, 1H), 3.96 (d, *J =* 10.8 Hz, 1H), 3.17 (dd, *J =* 9.3, 6.9 Hz, 1H), 3.06 – 2.97 (m, 3H), 2.37 – 2.16 (m, 3H), 2.01 – 1.96 (m, 1H), 1.88 (dq, *J =* 5.4, 2.6 Hz, 1H), 1.61 (m, 1H), 1.60 (s, 6H), 1.28 (d, *J =* 10.4 Hz, 1H),1.22 (s, 3H), 1.00 (s, 3H)

**^13^C NMR** (101 MHz, CD_2_Cl_2_) δ 179.9, 152.7, 149.7, 138.3, 138.3, 134.0, 128.5, 127.1, 121.1, 86.6, 70.3, 58.6, 57.3, 50.9, 46.9, 46.6, 40.5, 39.3, 34.6, 34.3, 27.4, 27.3, 26.3, 23.5.

**HRMS** (ESI) for C_29_H_35_IN_2_O_6_SNa: calculated: 689.1158 [M+Na]^+^, found 689.1155.

[α]^20^_D_ = +6.0° (CHCl_3_, *c* = 1.0).

**Methyl 2-(4-(((3a*S*,4*R*,6*R*,7a*S*)-3a-((((4-iodophenyl)carbamoyl)oxy)methyl)-5,5-dimethyloctahydro-2*H*-4,6-methanoisoindol-2-yl)sulfonyl)-3-methoxyphenyl)-2-methylpropanoate (93)：**

Following general procedure E2: From **S39** (67.7 mg, 0.15 mmol) and 1-iodo-4-isocyanatobenzene (73.5 mg, 1.5 eq.). Purification by column chromatography (PE/EtOAc 10:1, v/v) afforded a colorless solid **93** (R*_f_*= 0.4, PE:EA=3:1, v/v; 83.2 mg, 0.117 mmol, 78%).

**^1^H NMR** (500 MHz, CDCl_3_) δ 7.90 (d, *J =* 8.9 Hz, 1H), 7.60 (d, *J =* 8.7 Hz, 2H), 7.20 (d, *J =* 8.3 Hz, 2H), 7.07 (d, *J =* 2.6 Hz, 1H), 7.01 (s, 1H), 6.85 (dd, *J =* 8.9, 2.6 Hz, 1H), 4.22 (d, *J =* 10.6 Hz, 1H), 4.02 (d, *J =* 10.6 Hz, 1H), 3.84 (s, 3H), 3.65 (s, 3H), 3.55 (t, *J =* 8.6 Hz, 1H), 3.40 (d, *J =* 10.1 Hz, 1H), 3.10 (d, *J =* 10.1 Hz, 1H), 3.04 (dd, *J =* 9.7, 3.8 Hz, 1H), 2.37 (tt, *J =* 7.8, 4.1 Hz, 1H), 2.30 (dt, *J =* 13.3, 2.5 Hz, 1H), 2.27 – 2.21 (m, 1H), 1.92 (dd, *J =* 7.3, 2.5 Hz, 2H), 1.70 (s, 6H), 1.57 – 1.53 (m, 1H), 1.34 (d, *J =* 10.5 Hz, 1H), 1.24 (s, 3H), 1.03 (s, 3H).

**^13^C NMR** (126 MHz, CDCl_3_) δ 177.2, 162.6, 153.4, 146.7, 138.0, 137.9, 133.5, 129.5, 120.7, 115.2, 110.7, 86.4, 70.8, 58.1, 56.6, 55.6, 52.2, 50.9, 47.3, 46.8, 40.3, 39.1, 34.7, 34.1, 28.6, 28.6, 27.4, 27.4, 23.7.

**HRMS** (ESI) for C_31_H_39_IN_2_O_7_SNa: calculated: 711.1595 [M+Na]^+^, found 711.1607.

[α]^20^_D_ = -30.0° (CHCl_3_, *c* = 1.0).

**Methyl 2-(3-chloro-4-(((3aS,4R,6R,7aS)-3a-((((4-iodophenyl)carbamoyl)oxy)methyl)-5,5-dimethyloctahydro-2*H*-4,6-methanoisoindol-2-yl)sulfonyl)phenyl)-2-methylpropanoate (94)：**

Following general procedure E2: From **S40** (14.1 mg, 0.03 mmol) and 1-iodo-4-isocyanatobenzene (12 mg, 0.05 mmol, 1.2 eq.). Purification by column chromatography (PE/EtOAc 20:1 to 10:1, v/v) afforded a colorless solid **94** (R*_f_*= 0.6, PE:EA=3:1, v/v; 10 mg, 0.01 mmol, 47 %).

**^1^H NMR** (600 MHz, CDCl_3_) δ 8.01 (d, *J =* 8.3 Hz, 1H), 7.60 (d, *J =* 8.7 Hz, 2H), 7.48 (d, *J =* 1.9 Hz, 1H), 7.34 (dd, *J =* 8.4, 1.9 Hz, 1H), 7.18 (d, *J =* 8.2 Hz, 2H), 6.87 (s, 1H), 4.23 (d, *J =* 10.6 Hz, 1H), 4.05 (d, *J =* 10.6 Hz, 1H), 3.67 (s, 3H), 3.46 (d, *J =* 10.3 Hz, 1H), 3.21 (d, *J =* 10.3 Hz, 1H), 3.16 (dd, *J =* 10.0, 4.3 Hz, 1H), 2.39 (ddt, *J =* 11.1, 7.8, 4.0 Hz, 1H), 2.29 (ddt, *J =* 13.0, 10.4, 2.4 Hz, 1H), 2.24 (dtd, *J =* 10.5, 6.2, 1.9 Hz, 1H), 1.93 (t, *J =* 5.5 Hz, 2H), 1.66 – 1.61 (m, 2H), 1.58 (s, 9H), 1.33 (d, *J =* 10.6 Hz, 2H), 1.24 (s, 5H), 1.04 (s, 3H).

**^13^C NMR** (151 MHz, CDCl_3_) δ 176.0, 153.4, 151.3, 138.1, 137.8, 134.8, 132.7, 132.1, 129.9, 124.8, 120.7, 86.5, 70.7, 58.5, 56.8, 52.8, 51.2, 46.8, 46.5, 40.3, 39.0, 34.8, 33.8, 29.9, 27.4, 27.3, 26.4, 26.4, 23.7.

**HRMS** (ESI) for C_30_H_36_ClIN_2_O_6_S: calculated: 737.0925 [M+Na]^+^, found 737.0932.

[α]^20^_D_ = -16.2° (CHCl_3_, *c* = 0.25).

**2-(4-(((3a*S*,4*R*,6*R*,7a*S*)-3a-((((4-iodophenyl)carbamoyl)oxy)methyl)-5,5-dimethyloctahydro-2*H*-4,6-methanoisoindol-2-yl)sulfonyl)-3-methoxyphenyl)-2-methylpropanoic acid (95)：**

To a solution of **S39** (60.5 mg, 0.13 mmol, 1.0 eq.) in ethanol (0.1 M), cooled in an ice bath, a solution of sodium hydroxide (17.3 mg, 0.43 mmol, 3 eq.) in ethanol (0.1 M) was added dropwise. The aqueous phase was extracted with diethyl ether, and the combined organic layers were dried over anhydrous magnesium sulfate. After solvent removal under reduced pressure, the resulting carboxylic acid was obtained and used directly in the next step without further purification.

Following general procedure E3: From the crude carboxylic acid (45.2 mg, 0.1 mmol) and 1-iodo-4-isocyanatobenzene (29.4 mg, 0.12 mmol, 1.2 eq). Purification by column chromatography (DCM:MeOH=50:1 to 20:1, v/v) afforded a colorless solid **95**  (R*_f_*= 0.3, DCM:MeOH=20:1, v/v; 50.2 mg, 0.07 mmol, two steps, totally 72%).

**^1^H NMR** (500 MHz, CD_2_Cl_2_) δ 7.81 (d, *J =* 8.2 Hz, 1H), 7.60 (d, *J =* 8.8 Hz, 2H), 7.16 (d, *J =* 8.6 Hz, 2H), 7.06 (dd, *J =* 8.2, 1.7 Hz, 1H), 7.01 (d, *J =* 1.8 Hz, 1H), 6.84 (s, 1H), 4.12 (d, *J =* 10.6 Hz, 1H), 4.01 (d, *J =* 10.7 Hz, 1H), 3.89 (s, 3H), 3.50 (dd, *J =* 9.9, 6.8 Hz, 1H), 3.27 (d, *J =* 10.3 Hz, 1H), 3.15 (d, *J =* 10.4 Hz, 1H), 3.14 – 3.10 (m, 1H), 2.36 – 2.31 (m, 1H), 2.28 (td, *J =* 6.6, 3.1 Hz, 1H), 2.25 – 2.19 (m, 1H), 1.99 – 1.97 (m, 1H), 1.88 (dt, *J =* 5.4, 2.7 Hz, 1H), 1.61 (d, *J =* 3.5 Hz, 1H), 1.59 (s, 6H), 1.34 (d, *J =* 10.4 Hz, 1H), 1.23 (s, 3H), 1.02 (s, 3H).

**^13^C NMR** (126 MHz, CD_2_Cl_2_)δ 170.5, 157.5, 151.8, 145.4, 138.4, 138.3, 131.9, 125.3, 120.9, 118.3, 110.7, 87.3, 70.5, 58.3, 56.9, 56.3, 51.1, 46.6, 40.6, 39.4, 35.0, 34.3, 30.1, 27.4, 27.2, 26.4, 26.4, 23.5.

**HRMS** (ESI) for C_30_H_37_IN_2_O_7_SNa: calculated: 719.1264 [M+Na]^+^, found 719.1261.

[α]^20^_D_ = -12.3° (CHCl_3_, *c* = 0.5).

**2-(3-chloro-4-(((3a*S*,4*R*,6*R*,7a*S*)-3a-((((4-iodophenyl)carbamoyl)oxy)methyl)-5,5-dimethyloctahydro-2H-4,6-methanoisoindol-2-yl)sulfonyl)phenyl)-2-methylpropanoic acid (96):**

To a solution of **S40** (23.5 mg, 0.05 mmol, 1.0 eq.) in ethanol (0.1 M), cooled in an ice bath, a solution of sodium hydroxide (7 mg, 0.17 mmol, 3 eq.) in ethanol (0.1 M) was added dropwise. The aqueous phase was extracted with diethyl ether, and the combined organic layers were dried over anhydrous magnesium sulfate. After solvent removal under reduced pressure, the resulting carboxylic acid was obtained and used directly in the next step without further purification.

Following general procedure E3: From the crudecarboxylic acid (20.6 mg, 0.05 mmol) and 1-iodo-4-isocyanatobenzene (15 mg, 1.2 eq.). Purification by column chromatography (DCM: MeOH=50:1 to 20:1, v/v) afforded a colorless solid **96** (R*_f_*= 0.3, DCM:MeOH=20:1, v/v; 7 mg, 0.01 mmol, 20% for two steps).

**^1^H NMR** (600 MHz, CDCl_3_) δ 8.03 (d, *J =* 8.4 Hz, 1H), 7.60 (d, *J =* 8.7 Hz, 2H), 7.53 (d, *J =* 1.9 Hz, 1H), 7.40 (dd, *J =* 8.4, 1.9 Hz, 1H), 7.17 (s, 2H), 6.88 (s, 1H), 4.20 (d, *J =* 10.8 Hz, 1H), 4.05 (d, *J =* 10.7 Hz, 1H), 3.68 (s, 1H), 3.44 (s, 1H), 3.21 (d, *J =* 10.3 Hz, 1H), 3.17 (dd, *J =* 10.0, 4.3 Hz, 1H), 2.37 (d, *J =* 8.4 Hz, 1H), 2.33 – 2.26 (m, 2H), 2.27 – 2.20 (m, 2H), 1.93 (d, *J =* 6.3 Hz, 3H), 1.64 (d, *J =* 4.2 Hz, 1H), 1.65 – 1.58 (m, 6H), 1.33 (d, *J =* 10.6 Hz, 1H), 1.24 (s, 3H), 1.03 (s, 3H).

**^13^C NMR** (151 MHz, CDCl_3_) δ 177.5, 153.4, 150.6, 138.1, 137.8, 135.0, 132.8, 132.2, 130.0, 124.8, 120.7, 83.6, 70.7, 58.5, 56.7, 51.2, 46.5, 46.3, 40.3, 39.0, 34.8, 33.8, 29.9, 27.4, 27.3, 26.2, 23.7.

**HRMS** (ESI) for C_29_H_34_ClIN_2_O_6_SNa: calculated: 723.0728 [M+Na]^+^, found 723.0772.

[α]^20^_D_ = -24.1° (CHCl_3_, *c* = 0.5).

**2-(4-(((3a*S*,4*R*,6*R*,7a*S*)-3a-((((4-iodophenyl)carbamoyl)oxy)methyl)-5,5-dimethyloctahydro-2H-4,6-methanoisoindol-2-yl)sulfonyl)-3-methylphenyl)-2-methylpropanoic acid (97):**

The solution of sodium hydroxide (48 mg, 1.2 mmol, 3 eq.) in ethanol (0.1 M) was added dropwise to a solution of **S41** (179.8 mg, 0.4 mmol, 1.0 eq.) in ethanol (0.1 M) at 0 ºC. The resulting mixture was refluxed for 2-6 h, and then the solution was acidified with 2 N HCl to pH = 2. The resulting mixture was extracted by ethyl acetate and the combined organic extracts was dried by Na_2_SO_4._ Evaporation of the solvent afforded carboxylic acid without purification for next step.

Following general procedure E3: From the crude carboxylic acid (196 mg, 0.4 mmol) and 1-iodo-4-isocyanatobenzene (118 mg, 0.48 mmol, 1.2 eq.). Purification by column chromatography (DCM:MeOH=50:1 to 20:1, v/v) afforded a colorless solid **97** (R*_f_*= 0.3, DCM:MeOH=20:1, v/v; 153 mg, 0.22 mmol, 50% for two steps).

**^1^H NMR** (600 MHz, CD_2_Cl_2_)δ 7.86 (d, *J =* 8.9 Hz, 1H), 7.61 (d, *J =* 8.8 Hz, 2H), 7.37 – 7.32 (m, 2H), 7.22 – 7.12 (m, 2H), 6.98 (s, 1H), 4.14 – 4.03 (m, 2H), 3.42 (d, *J =* 12.2 Hz, 1H), 3.29 – 3.21 (m, 1H), 3.04 (d, *J =* 10.0 Hz, 2H), 3.03 – 2.99 (m, 1H), 2.64 (s, 3H), 2.36 – 2.27 (m, 1H), 2.24 – 2.18 (m, 1H), 1.95 (dd, *J =* 6.5, 5.0 Hz, 1H), 1.91 – 1.87 (m, 1H), 1.59 (s, 6H), 1.58 – 1.53 (m, 1H), 1.28 (d, *J =* 10.5 Hz, 1H), 1.23 (s, 3H), 1.02 (s, 3H).

**^13^C NMR** (151 MHz, CD_2_Cl_2_) δ 179.8, 153.6, 149.7, 138.8, 138.4, 138.3, 134.6, 130.8, 130.7, 124.0, 121.0, 86.4, 70.6, 58.1, 56.6, 51.1, 46.7, 46.7, 40.6, 39.3, 34.8, 34.4, 27.4, 26.4, 26.3, 23.6, 21.2.

**HRMS** (ESI) for C_30_H_37_IN_2_O_6_SNa: calculated: 703.1315 [M+Na]^+^, found 703.1316.

[α]^20^_D_ = -8.1° (CHCl_3_, *c* = 0.5).

## **General procedure F: Ethynyl installation**

To a degassed solution of Ar-I (1.0 eq.) in THF (0.1 M) was added NEt_3_ (5.0 eq.), trimethylsilylacetylene (2.0 eq.), CuI (0.2 eq.) and Pd(PPh_3_)_4_ (0.1 eq.). The solution was stirred at room temperature for 22 h. The reaction mixture was poured into sat. aq. NaCl solution, extracted with DCM twice, dried over Na_2_SO_4_ and the solvent was removed under reduced pressure. Column chromatography afforded the product.

To a solution of the above alkynylated product in THF (0.1 M) was added TBAF (1.2 eq.), and the mixture was stirred at room temperature for 1 h. The solvent was removed under reduced pressure. Column chromatography afforded a colorless solid.

**((3aS,4R,6R,7aS)-2-((4-(tert-butyl)phenyl)sulfonyl)-5,5-dimethyloctahydro-3aH-4,6-methanoisoindol-3a-yl)methyl (4-ethynylphenyl)carbamate^[17]^:**

Following general procedure F: From **iDeg-2** (19.1 mg, 0.03 mmol) and trimethylsilylacetylene (9 µl, 2.0 eq.). Purification by column chromatography (PE/EtOAc 15:1 to 10:1 to 5:1, v/v) afforded a colorless solid (R*_f_*= 0.6, PE:EA=3:1, v/v; 58%).

**1H NMR** (500 MHz, Methylene Chloride-d2) δ 7.72 (d, *J =* 8.6 Hz, 2H), 7.56 (d, *J =* 8.6 Hz, 2H), 7.43 (d, *J =* 8.7 Hz, 2H), 7.33 (d, *J =* 8.4 Hz, 2H), 6.63 (s, 1H), 4.12 (d, *J =* 10.7 Hz, 1H), 3.94 (d, *J =* 10.7 Hz, 1H), 3.21 (dd, *J =* 9.3, 7.1 Hz, 1H), 3.09 (s, 1H), 3.05 – 2.97 (m, 3H), 2.32 (tdd, *J =* 13.0, 3.1, 2.0 Hz, 1H), 2.26 (tt, *J =* 7.0, 3.6 Hz, 1H), 2.20 (dtd, *J =* 10.4, 6.3, 2.0 Hz, 1H), 1.98 (dd, *J =* 6.5, 5.0 Hz, 1H), 1.88 (tt, *J =* 5.7, 3.1 Hz, 1H), 1.63 – 1.59 (m, 1H), 1.31 (s, 9H), 1.28 (d, *J =* 9.9 Hz, 1H), 1.23 (s, 3H), 1.01 (s, 3H).

**13C NMR** (126 MHz, CD_2_Cl_2_)δ 157.2, 153.3, 142.6, 138.9, 133.3, 132.5, 128.3, 126.4, 118.5, 117.1, 83.6, 76.8, 70.4, 58.5, 57.3, 50.8, 46.6, 40.6, 39.4, 35.5, 34.7, 34.3, 31.2, 27.4, 27.2, 23.5.

**HRMS** (ESI) for C_31_H_39_N_2_O_4_S: calculated 535.2625 [M+H]^+^, found 535.2627.

[α]^20^_D_ = -12.4° (CHCl_3_, *c* = 0.5).

**((3a*S*,4*R*,6*R*,7a*S*)-2-((4-(*tert*-butyl)-2-methylphenyl) sulfonyl)-5,5-dimethyloctahydro-3a*H*-4,6-methanoisoindol-3a-yl)methyl (4-ethynylphenyl)carbamate (98)^[17]^:**

Following general procedure F: From **79** (89 mg, 0.14 mmol) and trimethylsilylacetylene (42 µl,0.28 mmol, 2.0 eq.). Purification by column chromatography (PE/EtOAc 15:1 to 10:1 to 5:1, v/v) afforded a colorless solid (R*_f_*= 0.6, PE:EA=3:1, v/v; 53%).

**^1^HNMR** (500 MHz, DMSO-*d*_6_) δ 9.75 (s, 1H), 7.72 (d, *J =* 8.3 Hz, 1H), 7.52 – 7.42 (m, 3H), 7.39 (d, *J =* 8.6 Hz, 3H), 4.21 (d, *J =* 10.9 Hz, 1H), 4.04 (s, 1H), 3.84 (d, *J =* 10.9 Hz, 1H), 3.06 (d, *J =* 9.9 Hz, 1H), 2.99 (dd, *J =* 9.4, 2.7 Hz, 1H), 2.90 (d, *J =* 9.9 Hz, 1H), 2.58 (s, 3H), 2.39 – 2.22 (m, 2H), 2.14 (dt, *J =* 10.9, 6.1 Hz, 1H), 1.96 (t, *J =* 5.7 Hz, 1H), 1.86 – 1.80 (m, 1H), 1.53 – 1.44 (m, 1H), 1.25 (s, 9H), 1.18 (d, *J =* 6.1 Hz, 4H), 0.99 (s, 3H).

**^13^CNMR** (126 MHz, DMSO-*d*_6_) δ 156.2, 153.3, 139.7, 137.2, 132.5, 132.3, 129.8, 129.6, 123.2, 118.1, 115.4, 83.6, 79.6, 68.5, 57.0, 56.0, 50.2, 45.5, 34.7, 33.8, 33.4, 30.7, 27.1, 26.3, 22.9, 20.5.

**HRMS** (ESI) for C_32_H_40_N_2_O_4_SNa: calculated: 571.2606 [M+Na]^+^, found 571.2602.

[α]^20^_D_ = +12.3° (CHCl_3_, *c* = 0.5).

**((3a*S*,4*R*,6*R*,7a*S*)-2-((4-(*tert*-butyl)-2-ethylphenyl)sulfonyl)-5,5-dimethyloctahydro-3a*H*-4,6-methanoisoindol-3a-yl)methyl (4-ethynylphenyl)carbamate (99)**:

Following general procedure F: From **80** (29.9 mg, 0.04 mmol) and trimethylsilylacetylene (42 µL,0.28 mmol, 2.0 eq.). Purification by column chromatography (PE/EtOAc 15:1 to 10:1 to 5:1, v/v) afforded a colorless solid (R*_f_*= 0.6, PE:EA=3:1, v/v; 53%).

**^1^H NMR** (500 MHz, CD_2_Cl_2_) δ 7.83 (d, *J =* 8.4 Hz, 1H), 7.47 – 7.43 (m, 2H), 7.42 (d, *J =* 2.1 Hz, 1H), 7.38 (d, *J =* 8.5 Hz, 2H), 7.35 (dd, *J =* 8.4, 2.0 Hz, 1H), 6.93 (s, 1H), 4.14 (d, *J =* 10.6 Hz, 1H), 4.09 (d, *J =* 10.7 Hz, 1H), 3.43 (dd, *J =* 9.5, 6.7 Hz, 1H), 3.28 (d, *J =* 10.1 Hz, 1H), 3.11 (s, 1H), 3.09 – 3.00 (m, 4H), 2.38 – 2.31 (m, 2H), 2.26 – 2.18 (m, 1H), 1.99 (dd, *J =* 6.5, 4.9 Hz, 1H), 1.91 (dq, *J =* 5.5, 3.0 Hz, 1H), 1.60 – 1.56 (m, 1H), 1.33 (s_br_, 10H), 1.31 (t, *J =* 5.4 Hz, 3H), 1.25 (s, 3H), 1.05 (s, 3H).

**^13^C NMR** (151 MHz, CD_2_Cl_2_) δ 157.2, 153.4, 144.6, 139.1, 133.3, 132.5, 130.5, 128.6, 123.4, 118.5, 117.0, 83.7, 76.8, 70.6, 58.1, 56.6, 51.0, 46.7, 40.6, 39.4, 35.3, 34.9, 34.3, 31.2, 27.4, 27.3, 27.0, 23.6, 16.3.

**HRMS** (ESI) for C_33_H_42_N_2_O_4_SNa: calculated: 585.2763 [M+Na]^+^, found 585.2753.

[α]^20^_D_ = +12.0° (CHCl_3_, *c* = 0.5).

**2-(4-(((3a*S*,4*R*,6*R*,7a*S*)-3a-((((4-ethynylphenyl)carbamoyl)oxy)methyl)-5,5-dimethyloctahydro-2*H*-4,6-methanoisoindol-2-yl)sulfonyl)phenyl)-2-methylpropanoic acid (101):**

Following general procedure F: From **92** (46.8 mg, 0.07 mmol) and trimethylsilylacetylene (11.3 µL, 0.1 mmol, 2.0 eq.). Purification by column chromatography (DCM:MeOH =50:1 to 20:1, v/v) afforded a colorless solid (R*_f_*= 0.5, DCM:MeOH=20:1, v/v; 45%).

**^1^H NMR** (700 MHz, CDCl_3_) δ 7.78 (d, *J =* 8.4 Hz, 2H), 7.55 (d, *J =* 8.6 Hz, 2H), 7.43 (d, *J =* 8.7 Hz, 2H), 7.29 (s, 2H), 6.60 (s, 1H), 4.03 (d, *J =* 12.7 Hz, 2H), 3.22 – 3.14 (m, 1H), 3.09 (d, *J =* 9.9 Hz, 1H), 3.07 – 3.00 (m, 3H), 2.32 (dt, *J =* 10.3, 2.6 Hz, 1H), 2.30 – 2.26 (m, 1H), 2.20 (dtd, *J =* 10.4, 6.2, 1.8 Hz, 1H), 1.95 (dd, *J =* 6.5, 4.9 Hz, 1H), 1.89 (dq, *J =* 5.5, 2.7 Hz, 1H), 1.64 – 1.62 (m, 1H), 1.62 (d, *J =* 1.4 Hz, 6H), 1.33 (d, *J =* 10.6 Hz, 1H), 1.23 (s, 3H), 1.01 (s, 3H).

**^13^C NMR** (176 MHz, CDCl_3_) δ 179.5, 153.2, 149.2, 138.2, 134.0, 133.2, 130.1, 128.4, 126.7, 125.9, 118.5, 117.3, 83.4, 70.2, 58.3, 57.0, 50.8, 46.6, 46.5, 40.2, 39.2, 34.4, 34.1, 27.4, 27.1, 26.3, 23.5.

**HRMS** (ESI) for C_31_H_36_N_2_O_6_S: calculated: 587.2192 [M+Na]^+^, found 587.2179.

[α]^20^_D_ = -136.1° (CHCl_3_, *c* = 0.25).

**2-(4-(((3a*S*,4*R*,6*R*,7a*S*)-3a-((((4-ethynylphenyl)carbamoyl)oxy)methyl)-5,5-dimethyloctahydro-2*H*-4,6-methanoisoindol-2-yl)sulfonyl)-3-methylphenyl)-2-methylpropanoic acid (102):**

Following general procedure F: From **97** (110 mg, 0.16 mmol) and trimethylsilylacetylene (45.2 µL,0.32 mmol, 2.0 eq.). Purification by column chromatography (DCM:MeOH =50:1 to 20:1, v/v ) afforded a colorless solid (R*_f_*= 0.5, DCM:MeOH=20:1, v/v; 67%).

**^1^H NMR** (700 MHz, CD_2_Cl_2_)δ 7.86 (d, *J =* 8.8 Hz, 1H), 7.43 (d, *J =* 8.7 Hz, 2H), 7.36 (s, 1H), 7.34 (d, *J =* 6.4 Hz, 2H), 6.93 (s, 1H), 4.10 (s, 2H), 3.41 (s, 1H), 3.26 (d, *J =* 10.2 Hz, 1H), 3.09 (s, 1H), 3.07 – 3.00 (m, 2H), 2.64 (s, 3H), 2.32 (td, *J =* 7.3, 3.1 Hz, 2H), 2.21 (dtd, *J =* 10.5, 6.4, 1.6 Hz, 1H), 1.96 (dd, *J =* 6.4, 5.0 Hz, 1H), 1.91 – 1.88 (m, 1H), 1.59 (s, 6H), 1.56 (s, 1H), 1.29 (d, *J =* 10.4 Hz, 1H), 1.23 (s, 3H), 1.03 (s, 3H).

**^13^C NMR** (151 MHz, CD_2_Cl_2_) δ 178.1, 153.5, 149.7, 139.0, 138.8, 134.6, 133.3, 130.7, 130.7, 126.1, 124.0, 118.6, 117.1, 83.6, 76.8, 70.6, 58.1, 56.7, 51.1, 46.7, 46.6, 40.6, 39.3 34.8, 34.4, 27.4, 26.4, 26.4, 23.6, 21.2.

**HRMS** (ESI) for C_32_H_38_N_2_O_6_SNa: calculated: 601.2348 [M+Na]^+^, found 601.2341.

[α]^20^_D_ = -72.4° (CHCl_3_, *c* = 0.25).

**General synthesis of 6-7:**

**General procedure a:**

To a solution of substrate **4a** (1.0 g, 3.63 mmol) in 320 mL sd. NH_3_-*i*PrOH solution was slowly added Ti(O*i*Pr)_4_ (1.28 mL, 4.33 mmol) at -15 °C. The mixture was warmed to room temperature and stirred for 4 h. Then NaBH_4_ (193 mg, 5.08 mmol) was added and stirring was continued for another 30 minutes The reaction was then quenched with 100 mL *con*. ammonia solution. then diluted with 70 mL water and extracted with EtOAc. The organics were combined and dried over Na_2_SO_4_ and concentrated. The residue was simply purified by silica gel chromatography with CHCl_3_-CH_3_OH (20: 1, v/v) to get amino product (R*_f_*= 0.5, DCM:MeOH=20:1, v/v; light yellow liquid, 50%).

**General procedure b:**

To a solution of amino (54.4 mg, 0.19 mmol) in toluene (0.1 mol/L) at ice bath. 1-isocyanato-4-methylbenzene (0.19 mmol, 1.0 eq.) was added to the above solution, and then the reaction mixture was stirred about 24 h, and then purified by column and gave the desired product **6** (R*_f_*= 0.6, DCM:MeOH=20:1, v/v; light yellow liquid; 68.3 mg, 0.16 mmol, 86%).

**1-(((3aR,4R,6R,7aS)-2-benzyl-5,5-dimethyloctahydro-3aH-4,6-methanoisoindol-3a-yl) methyl)-3-(p-tolyl) urea (6):**

**^1^H NMR** (500 MHz, Methylene Chloride-*d*_2_) δ 7.24 (ddd, *J =* 13.5, 7.7, 6.0 Hz, 3H), 7.19 (d, *J =* 8.4 Hz, 2H), 7.10 (dd, *J =* 8.3, 6.5 Hz, 4H), 6.49 (s, 1H), 6.20 (s, 1H), 3.60 (d, *J =* 12.7 Hz, 1H), 3.56 (d, *J =* 12.3 Hz, 1H), 3.37 (d, *J =* 12.7 Hz, 1H), 3.17 (t, *J =* 8.4 Hz, 1H), 2.91 (d, *J =* 12.4 Hz, 1H), 2.43 (d, *J =* 9.4 Hz, 1H), 2.38 (dd, *J =* 9.4, 1.4 Hz, 1H), 2.34 – 2.29 (m, 1H), 2.27 (s, 3H), 2.22 – 2.13 (m, 3H), 1.89 – 1.85 (m, 1H), 1.78 (dd, *J =* 6.3, 4.9 Hz, 1H), 1.59 – 1.52 (m, 2H), 1.23 (s, 3H), 1.02 (s, 3H).

**^13^C NMR** (126 MHz, CD_2_Cl_2_) δ 156.5, 139.5, 137.1, 133.3, 129.9, 129.0, 128.6, 127.3, 121.1, 67.0, 64.8, 60.1, 50.6, 49.4, 48.0, 41.3, 39.2, 34.3, 32.7, 27.6, 27.4, 24.2, 20.8.

**HRMS (ESI)** for C_27_H_36_N_3_O: calculated 418.2853 [M+H]^+^, found 418.2866.

[α]^20^_D_ = -8.2° (CHCl_3_, *c* = 0.5).

**1-(((3aR,4R,6R,7aS)-2-((4-(tert-butyl) phenyl) sulfonyl)-5,5-dimethyloctahydro-3aH-4,6-methanoisoindol-3a-yl) methyl)-3-(p-tolyl) urea (7):**

Following general procedure C3: From **6** (0.14 mmol, 1 eq.) to afford a colorless aminewithout further purification for the next step.

Following general procedure D2: From the above obtained amine(26.3 mg, 0.13 mmol 1 eq.) and 4-*tert*-Butylbenzenesulfonyl chloride (30.3 mg, 0.13 mmol, 1.0 eq.) to afford a colorless solid **7** (R*_f_*= 0.6, DCM:MeOH=20:1, v/v; 52.4 mg, 0.1 mmol, 77%).

**^1^H NMR** (500 MHz, Chloroform-*d*) δ 7.72 (d, *J =* 8.5 Hz, 2H), 7.54 (d, *J =* 8.6 Hz, 2H), 7.12 (s, 4H), 6.63 (s, 1H), 5.12 (s, 1H), 3.51 (d, *J =* 13.7 Hz, 1H), 3.41 (d, *J =* 1.5 Hz, 1H), 3.12 (d, *J =* 13.7 Hz, 1H), 3.06 (d, *J =* 9.8 Hz, 1H), 2.91 (d, *J =* 9.9 Hz, 1H), 2.78 (dd, *J =* 9.4, 5.2 Hz, 1H), 2.40 – 2.33 (m, 1H), 2.31 (s, 3H), 2.26 – 2.18 (m, 1H), 2.14 – 2.06 (m, 1H), 1.82 (d, *J =* 6.5 Hz, 2H), 1.49 (d, *J =* 14.0 Hz, 1H), 1.32 (s, 9H), 1.19 (s, 3H), 1.06 (d, *J =* 10.5 Hz, 1H), 1.03 (s, 3H).

**^13^C NMR** (126 MHz, CDCl_3_) δ 157.0, 156.6, 135.5, 131.8, 130.1, 128.8, 128.1, 126.2, 122.0, 59.0, 57.8, 51.2, 48.2, 47.5, 40.2, 39.0, 35.3, 34.4, 33.5, 31.2, 27.4, 27.1, 23.8, 21.0.

**HRMS (ESI)** for C_30_H_42_N_3_O_3_S: calculated 524.2941 [M+H]^+^, found 524.2944.

[α]^20^_D_ = +46.7° (CHCl_3_, *c* = 0.2).

**General synthesis of 13:**

To a solution of alcohol **12** (58.7 mg, 0.15 mmol) and pyridine (24.3 µL, 0.3 mmol) in anhydrous DCM (0.8 mL) at 0 °C, 4-Me-phenylchloroformate (33.7 µL, 0.225 mmol) was added in one portion. The resulting suspension was stirred at room temperature for overnight, then diluted with DCM (2 mL) and washed quickly with 1 M HCl (5 mL) and brine (5 mL), dried (Na_2_SO_4_) and concentrated to afford a colorless solid foam. The crude product was purified by automated column chromatography (4 g silica column, 2 to 40% EtOAc in hexanes over 20 min,) to afford the desired compound **13** (R*_f_*= 0.8, PE:EA=5:1, v/v; 77.8 mg, 98%) as a white amorphous solid.

**((3aS,4R,6R,7aS)-2-((4-(tert-butyl)phenyl)sulfonyl)-5,5-dimethyloctahydro-3aH-4,6-methanoisoindol-3a-yl)methyl p-tolyl carbonate (13):**

**^1^H NMR** (500 MHz, Chloroform-*d*) δ 7.74 (d, *J =* 8.6 Hz, 2H), 7.54 (d, *J =* 8.5 Hz, 2H), 7.18 – 7.14 (m, 2H), 6.96 (d, *J =* 8.5 Hz, 2H), 4.17 (d, *J =* 10.6 Hz, 1H), 4.00 (d, *J =* 10.6 Hz, 1H), 3.16 (dd, *J =* 9.2, 6.9 Hz, 1H), 3.11 (d, *J =* 9.9 Hz, 1H), 3.07 (dd, *J =* 9.2, 3.1 Hz, 1H), 3.01 (d, *J =* 9.9 Hz, 1H), 2.34 (s, 3H), 2.33 – 2.28 (m, 1H), 2.23 (dtd, *J =* 10.4, 3.9, 1.4 Hz, 2H), 2.01 (d, *J =* 1.5 Hz, 1H), 1.90 (dq, *J =* 5.4, 2.8 Hz, 1H), 1.66 (ddd, *J =* 13.2, 4.4, 2.8 Hz, 1H), 1.39 (d, *J =* 10.5 Hz, 1H), 1.31 (s, 9H), 1.23 (s, 3H), 1.00 (s, 3H).

**^13^C NMR** (126 MHz, CDCl_3_) δ 156.8, 154.0, 148.9, 136.0, 131.9, 130.1, 128.1, 126.1, 120.8, 73.1, 58.0, 56.8, 50.4, 46.2, 40.1, 39.2, 35.3, 34.4, 34.2, 31.2, 27.3, 27.0, 23.4, 21.0.

**HRMS** (ESI) for C_30_H_40_NO_5_S: calculated 526.2622 [M+H]^+^, found 526.2625.

[α]^20^_D_ = -14.3° (CHCl_3_, *c* = 1.0).

**((3aR,4R,6R)-2-((4-(tert-butyl)phenyl)sulfonyl)-5,5-dimethyloctahydro-3aH-4,6-methanoisoindol-3a-yl)methyl benzoate (15):**

To a solution of **12** (25.0 mg, 63.9 µmol) in THF (0.5 mL) was added NEt_3_ (22 µL, 0.160 mmol, 2.5 eq.) and benzoyl chloride (8.8 µL, 76.6 µmol, 1.2 eq.), and the reaction mixture was stirred at room temperature for 20 h. The solvent was removed under reduced pressure. Column chromatography (PE/EtOAc 16:1, v/v) afforded a colorless solid **15** (R*_f_*= 0.8, PE:EA=5:1, v/v; 19.0 mg, 38.3 µmol, 60%).

**^1^H-NMR** (CDCl_3_, 600 MHz): δ = 7.79–7.81 (m, 2 × Ar-H), 7.71–7.74 (m, 2 H, 2 × Ar-H), 7.56 (tt, *J =* 7.4, 1.3 Hz, 1 H, Ar-H), 7.47–7.50 (m, 2 H, 2 × Ar-H), 7.39–7.42 (m, 2 H, 2 × Ar-H), 4.15 (d, *J =* 11.0 Hz, 1 H, CH_B_), 4.11 (d, *J =* 11.0 Hz, 1 H, CH_A_), 3.17–3.24 (m, 3 H, 3 × CH), 1.01 (s, 3 H, CH_3_), 2.98 (d, *J =* 9.7 Hz, 1 H, CH), 2.32–2.39 (m, 2 H, 2 × CH), 2.23–2.27 (m, 1 H, CH), 2.03 (dd, *J =* 6.4, 5.0 Hz, 1 H, CH), 1.89–1.93 (m, 1 H, CH), 1.67–1.73 (m, 1 H, CH), 1.50 (d, *J =* 10.5 Hz, 1 H, CH), 1.25 (s, 9 H, 3 × CH_3_), 1.24 (s, 3 H, CH_3_).

**^13^C-NMR** (CDCl_3_, 150 MHz):δ = 166.5, 156.7, 133.2, 132.0, 129.8, 129.6, 128.6, 128.1, 126.0, 69.9, 58.3, 57.2, 50.5, 46.9, 40.2, 39.2, 35.2, 34.7, 34.5, 31.1, 27.4, 27.1, 23.5.

**HRMS** (ESI) for C_29_H_37_NO_4_S: calculated 496.2516 [M+H]^+^, found 496.2518.

**General procedure of 16:**

To a solution of **11** (0.2 mmol) in THF (2 mL) was added NEt_3_ (56.7 µL, 0.4 mmol, 2 eq.) and benzoyl chloride (55.68 mg, 0.24 mmol, 1.2 eq.), and the reaction mixture was stirred at room temperature for 20 h. The solvent was removed under reduced pressure. Column chromatography (PE/EtOAc 16:1, v/v) afforded a colorless solid **S46** (R*_f_*= 0.2, PE:EA=2:1, v/v; 19.0 mg, 38.3 µmol, 60%).

**(4-(*tert*-butyl)phenyl)((3aS,4R,6R,7aS)-3a-(hydroxymethyl)-5,5-dimethyloctahydro-2H-4,6-methanoisoindol-2-yl)methanone (S46):**

**^1^H NMR** (500 MHz, Methylene Chloride-*d*_2_) δ 7.41 (s_br_, 4H), 3.82 (d, *J =* 45.2 Hz, 1H), 3.70 – 3.49 (m, 4H), 3.28 (s, 1H), 2.38 – 2.15 (m, 3H), 2.02 (s, 1H), 1.87 (td, *J =* 5.5, 2.7 Hz, 1H), 1.64 (s, 1H), 1.33 (s, 9H), 1.22 (d, *J =* 9.6 Hz, 3H), 1.13 (d, *J =* 22.5 Hz, 1H), 0.98 (s, 3H).

**^13^C NMR** (126 MHz, CD_2_Cl_2_) δ 169.4, 153.4, 134.5, 127.5, 125.5, 68.8, 60.2, 58.4, 56.0, 51.8, 47.0, 47.0, 41.0, 39.4, 35.5, 35.2, 31.4, 28.2, 27.6, 23.9.

**HRMS** (ESI) for C_23_H_34_NO_2_: calculated 356.2584 [M+H]^+^, found 356.2584.

[α]^20^_D_ = +4.3° (CHCl_3_, *c* = 1.0).

**((3aS,4R,6R,7aS)-2-(4-(tert-butyl)benzoyl)-5,5-dimethyloctahydro-3aH-4,6-methanoisoindol-3a-yl)methyl (4-iodophenyl)carbamate (16):**

Following general procedure E2: From **S46** (53.33 mg, 0.15 mmol) and 1-iodo-4-isocyanatobenzene (73.5 mg, 1.2 eq.). Purification by column chromatography (PE/EtOAc 15:1, v/v) afforded a colorless solid **93** (R*_f_*= 0.6, PE:EA=3:1, v/v; 83.2 mg, 0.117 mmol, 78%).

**^1^H NMR** (500 MHz, Chloroform-*d*) δ 7.52 (d, *J =* 8.7 Hz, 2H), 7.33 (s_br_, 4H), 7.18 – 7.06 (m, 2H), 6.99 (s_br_, 1H), 4.23 (s_br_, 1H), 4.06 – 3.60 (m, 4H), 3.33 (dd, *J =* 24.5, 11.7 Hz, 1H), 2.29 (m, 3H), 2.04 – 1.96 (m, 1H), 1.85 (s_br_, 1H), 1.39 (d, *J =* 12.9 Hz, 1H), 1.25 (s, 9H), 1.21 – 1.15 (m, 4H), 0.98 (s, 3H).

**^13^C NMR** (126 MHz, CD_2_Cl_2_) δ 169.2, 153.7, 138.7, 138.2, 134.2, 127.5, 125.6, 120.9, 86.1, 71.0, 59.6, 54.6, 51.9, 50.1, 47.3, 40.8, 39.3, 35.7, 35.1, 31.3, 28.1, 27.5, 23.7.

**HRMS (ESI)** for C_30_H_38_IN_2_O_3_: calculated 601.1922 [M+H]^+^, found 601.1903.

[α]^20^_D_ = +4.5° (CHCl_3_, *c* = 1.0).

**General procedure of 18-19:**

**General procedure a:** A mixture of amino (39.5 mg, 0.2 mmol), benzenesulfinamide (197.6 mg, 0.3 mmol), CuBr_2_ (44.0 mg, 20 mol%) and toluene (10 mL) in a 100 mL round-bottom flask was stirred for 12 h at room temperature, and then concentrated in vacuo. The product was purified by flash column chromatography using n-pentane/ethyl acetate (20:1 to 10:1, v/v) as the eluent to afford the product as a colorless liquid (67%).

**General procedure b**：To a solution of the substrate that obtained from the above step (0.07 mmol) in THF (1.0 mL) was added a 1.0 M solution of TBAF in THF (0.14 mL,0.14 mmol) at room temperature. The resulting solution was stirred for 3 h. The reaction mixture was evaporated in vacuo and the crude mixture was purified by a simply silica gel flash column chromatography (PE:EA=4:1,v/v) to afford quant. products **17**,which was used in next step immediately.

**General procedure E2:** Following general procedure **E2**: From **17** (13.67 mg, 0.04 mmol) and 1-iodo-4-isocyanatobenzene (1.2 eq.) to afford **18-19**. Purification by column chromatography (PE:EA =20:1) obtained the products **18-19** (R*_f_*= 0.6, PE:EA=3:1, v/v; 14 mg. 0.02 mmol, 62%).

**(3aS,4R,6R,7aS)-3a-(((*tert*-butyldimethylsilyl)oxy)methyl)-5,5-dimethyloctahydro-1H-4,6-methanoisoindole (S47):**

**^1^H NMR** (500 MHz, Chloroform-d)δ 3.75 – 3.70 (m, 1H), 3.56 (d, *J =* 7.8 Hz, 1H), 3.50 – 3.46 (m, 1H), 3.40 (d, *J =* 11.8 Hz, 1H), 3.12 (dq, *J =* 11.9, 6.7, 5.6 Hz, 2H), 2.46 – 2.40 (m, 1H), 2.35 (dt, *J =* 9.5, 5.4 Hz, 1H), 2.31 – 2.25 (m, 1H), 1.98 (d, *J =* 6.0 Hz, 2H), 1.76 (dt, *J =* 13.6, 3.0 Hz, 1H), 1.35 – 1.31 (m, 1H), 1.25 (s, 3H), 0.98 (s, 3H), 0.89 (d, *J =* 0.8 Hz, 9H), 0.12 – 0.07 (m, 6H).

**^13^C NMR** (126 MHz, CDCl_3_) 68.7, 55.5, 54.07, 53.9, 52.6, 45.5, 40.6, 38.9, 33.3, 32.5, 28.1, 27.5, 26.1, 24.0, 18.4, -5.2 (2C).

**HRMS (ESI)** for C_18_H_36_NOSi: calculated 310.2561 [M+H]^+^, found 310.2561.

**(4-(tert-butyl) phenyl) ((3aS,4R,6R,7aS)-3a-((((4-iodophenyl) carbamoyl)oxy) methyl)-5,5-dimethyloctahydro-2H-4,6-methanoisoindol-2-yl) (methylidyne)-λ^6^-sulfanolate (18):**

**Physical state**: white solid

**^1^H NMR** (500 MHz, Chloroform-*d*) δ 7.95 (s, 1H), 7.58 (d, *J =* 8.7 Hz, 4H), 7.50 (d, *J =* 8.5 Hz, 2H), 7.23 (d, *J =* 8.4 Hz, 2H), 4.33 (d, *J =* 10.4 Hz, 1H), 3.98 (d, *J =* 10.4 Hz, 1H), 3.62 (dd, *J =* 10.4, 8.2 Hz, 1H), 3.48 (d, *J =* 10.4 Hz, 1H), 3.33 (d, *J =* 10.4 Hz, 1H), 2.48 (dd, *J =* 10.3, 4.9 Hz, 1H), 2.36 – 2.25 (m, 2H), 2.20 (ddt, *J =* 11.3, 8.3, 2.5 Hz, 1H), 1.94 (dd, *J =* 6.4, 4.9 Hz, 1H), 1.90 (d, *J =* 2.6 Hz, 1H), 1.47 (d, *J =* 10.4 Hz, 1H), 1.43 (d, *J =* 14.1 Hz, 1H), 1.32 (s, 9H), 1.25 (s, 3H), 1.02 (s, 3H).

**^13^C NMR** (126 MHz, CDCl_3_) δ 154.2, 153.6, 141.1, 138.2, 137.8, 125.9, 125.5, 120.4, 85.8, 70.8, 54.2, 51.2, 46.8, 40.3, 38.9, 34.9, 34.5, 33.6, 31.2, 27.3, 27.1, 23.7.

**HRMS** (ESI) for C_29_H_38_IN_2_O_3_S: calculated 621.1642 [M+H]^+^, found 621.1651.

[α]^20^_D_ = - 87.3° (CHCl_3_, *c* = 0.25).

**(4-(tert-butyl) phenyl)((3aS,4R,6R,7aS)-3a-((((4-iodophenyl)carbamoyl)oxy)methyl)-5,5-dimethyloctahydro-2H-4,6-methanoisoindol-2-yl)(methylidyne)-λ^6^-sulfanolate (19):**

**Physical state**: oil foams

**^1^H NMR** (500 MHz, Chloroform-*d*) δ 7.58 (dd, *J =* 8.7, 3.3 Hz, 4H), 7.50 (d, *J =* 8.6 Hz, 2H), 7.25 (s, 1H), 7.21 (d, *J =* 8.4 Hz, 2H), 4.18 (d, *J =* 10.6 Hz, 1H), 4.02 (d, *J =* 10.7 Hz, 1H), 3.53 – 3.43 (m, 1H), 3.32 (d, *J =* 10.0 Hz, 1H), 3.14 (dd, *J =* 9.4, 2.4 Hz, 1H), 2.43 (d, *J =* 10.0 Hz, 1H), 2.34 (t, *J =* 4.0 Hz, 2H), 2.12 (ddd, *J =* 10.6, 5.1, 3.1 Hz, 1H), 1.90 (dt, *J =* 5.9, 2.8 Hz, 1H), 1.85 (dd, *J =* 6.4, 5.0 Hz, 1H), 1.65 (dd, *J =* 8.8, 2.8 Hz, 1H), 1.35 (s, 9H), 1.28 (d, *J =* 10.3 Hz, 1H), 1.20 (s, 3H), 1.04 (s, 3H).

**^13^C NMR** (126 MHz, CDCl_3_) δ 154.5, 153.6, 140.2, 138.0, 137.9, 126.1, 125.4, 120.7, 86.3, 70.5, 57.9, 51.7, 50.1, 46.9, 40.4, 39.2, 35.1, 34.9, 34.6, 31.4, 27.5, 27.3, 23.6.

**HRMS** (ESI) for C_29_H_38_IN_2_O_3_S: calculated 621.1642 [M+H]^+^, found 621.1651.

[α]^20^_D_ = +85.3° (CHCl_3_, *c* = 0.25).

**Synthesis of 41:**

**((3a*S*,4*R*,6*R*,7a*S*)-2-((4-(*tert*-butyl)phenyl)sulfonyl)-5,5-dimethyloctahydro-3a*H*-4,6-methanoisoindol-3a-yl)methyl (3-azidophenyl)carbamate (41):**

To a solution of **40** (30.0 mg, 57.1 µmol) in MeCN (0.5 mL) at 0 °C was added *tert*-butyl nitrite (10 µL, 85.6 µmol, 1.5 eq.) and trimethylsilyl azide (9.0 µL, 68.5 µmol, 1.2 eq.).^[18]^ The cooling bath was removed and stirring at room temperature was continued for 1 h. The solvent was removed under reduced pressure. Column chromatography (PE/EtOAc = 10:1, v/v) afforded a colorless oil foams (R*_f_*= 0.6, PE:EA=3:1, v/v; 22.0 mg, 39.9 µmol, 70%).

**^1^H-NMR** (500 MHz, CDCl_3_): δ 7.74 (d, *J =* 8.5 Hz, 2H), 7.54 (d, *J =* 8.6 Hz, 2H), 7.28 – 7.23 (m, 1H), 7.17 (s_br_, 1H), 7.03 (m, 1H), 6.74 (ddd, *J =* 8.0, 2.2, 0.9 Hz, 1H), 6.59 (s, 1H), 4.10 (d, *J =* 11.2 Hz, 1H), 3.98 (d, *J =* 10.6 Hz, 1H), 3.21 (dd, *J =* 9.3, 6.8 Hz, 1H), 3.11 – 3.05 (m, 2H), 3.04 (dd, *J =* 5.5, 3.7 Hz, 1H), 2.34 – 2.23 (m, 2H), 2.23 – 2.17 (m, 1H), 1.96 (dd, *J =* 6.4, 4.9 Hz, 1H), 1.92 – 1.86 (m, 1H), 1.65 – 1.59 (m, 1H), 1.34 (d, *J =* 10.6 Hz, 1H), 1.31 (s, 9H), 1.22 (s, 3H), 1.01 (s, 3H).

**^13^C-NMR** (125 MHz, CDCl_3_):δ 156.9, 153.2, 141.1, 139.3, 132.2, 130.4, 128.1, 126.1, 115.09, 114.2, 109.3, 77.4, 70.3, 58.2, 57.0, 50.6, 46.4, 40.1, 39.1, 35.3, 34.5, 34.1, 31.2, 27.4, 27.1, 23.5.

**HRMS** (ESI) for C_29_H_38_N_5_O_4_S: calculated 552.2639 [M+H]^+^, found 552.2645.

**Synthesis of compounds 42:**

**((3a*S*,4*R*,6*R*,7a*S*)-2-((4-(tert-butyl) phenyl) sulfonyl)-5,5-dimethyloctahydro-3a*H*-4,6-methanoisoindol-3a-yl)methyl (4-hydroxyphenyl)carbamate (42):**

To a solution of **44** (75.0 mg, 0.139 mmol) in DCM (1 mL) at −78 °C was added dropwise BBr_3_ (694 µL, 0.694 mmol, 1 m in DCM, 5.0 eq.) and the mixture was allowed to warm to room temperature over 16 h. The reaction mixture was poured into sat. aq. NaHCO_3_ solution, extracted with DCM (2 × 20 mL), dried over MgSO_4_ and the solvent was removed under reduced pressure. Column chromatography (PE/EtOAc 3:1 to 2:1, v/v) afforded a colorless oil foams (R*_f_*= 0.4, PE:EA=1:1, v/v; 60.0 mg, 0.114 mmol, 82%).

**^1^H-NMR** (CDCl_3_, 500 MHz): δ 7.68 (d, *J =* 8.1 Hz, 2H), 7.52 – 7.46 (m, 2H), 7.16 – 7.05 (m, 2H), 6.70 (d, *J =* 8.9 Hz, 2H), 6.19 (s, 1H), 5.19 (s, 1H), 3.94 (s_br_, 2H), 3.10 (s_br_, 1H), 3.00 (m, 3H), 2.22 (m, 2H), 2.17 – 2.10 (m, 1H), 1.89 – 1.79 (m, 2H), 1.55 (d, *J =* 12.1 Hz, 1H), 1.26 (s, 1H), 1.23 (s, 9H), 1.15 (s, 3H), 0.93 (s, 3H).

**^13^C-NMR** (CDCl_3_, 125 MHz):δ = 23.5, 27.1, 27.4, 29.8, 31.2, 34.3, 34.5, 35.3, 39.1, 40.2, 46.5, 50.7, 57.0, 58.2, 70.1, 115.9, 121.4, 126.1, 128.1, 132.2, 156.9.

**HRMS** (ESI) for C_29_H_39_N_2_O_5_S: calculated 527.2574 [M+H]^+^, found 527.2578.

**Synthesis of 53:**

To a solution of **iDeg-2** (63.7 mmol, 0.1 mmol,1.0 eq.), PdCl_2_(PPh_3_)_2_ (7 mg, 0.1 eq.), CuI (5.7 mg, 0.3 eq.) in Et_3_N (0.25 M, 0.4 eq.) was added but-3-yn-1-ol (10 µL, 1.3 eq.) and the mixture was stirred at room temperature for 24 h under Ar atmosphere. After completion, the resulting mixture was concentrated under reduced pressure and the residue was subjected to column chromatography on silica gel (PE:EA=10:1 to 3:1, v/v) to give the desired coupling product (R*_f_*= 0.2, PE:EA=3:1, v/v, 56 mg, 0.1 mmol, quant.) **53**.

**((3a*S*,4*R*,6*R*,7a*S*)-2-((4-(tert-butyl) phenyl)sulfonyl)-5,5-dimethyloctahydro-3a*H*-4,6-methanoisoindol-3a-yl)methyl (4-(4-hydroxybut-1-yn-1-yl)phenyl)carbamate (53):**

**Physical state**: white solid

**^1^H NMR** (600 MHz, CDCl_3_) δ 7.74 (d, *J =* 8.5 Hz, 2H), 7.53 (d, *J =* 8.5 Hz, 2H), 7.34 (d, *J =* 8.6 Hz, 2H), 7.28 (s, 2H), 6.66 (s, 1H), 4.07 (d, *J =* 10.7 Hz, 1H), 3.99 (d, *J =* 10.9 Hz, 1H), 3.81 (t, *J =* 6.3 Hz, 2H), 3.22 (dd, *J =* 9.2, 6.8 Hz, 1H), 3.06 (d, *J =* 2.1 Hz, 2H), 3.03 (dd, *J =* 9.3, 3.0 Hz, 1H), 2.68 (t, *J =* 6.3 Hz, 2H), 2.28 (ddt, *J =* 13.1, 7.1, 3.0 Hz, 2H), 2.18 (ddd, *J =* 10.5, 6.1, 1.7 Hz, 1H), 2.15 (s_br_, 1H), 1.95 (dd, *J =* 6.5, 4.9 Hz, 1H), 1.88 (dd, *J =* 5.5, 3.0 Hz, 1H), 1.61 (dt, *J =* 12.3, 3.2 Hz, 1H), 1.33-1.31 (m, 10H), 1.22 (s, 3H), 1.00 (s, 3H).

**^13^C NMR** (151 MHz, CDCl_3_) δ 156.9, 153.2, 137.5, 132.6, 132.3, 128.1, 126.1, 118.4, 85.8, 82.2, 70.3, 61.3, 58.2, 57.0, 50.6, 46.4, 40.2, 39.1, 35.3, 34.5, 34.1, 31.2, 27.4, 27.1, 23.9, 23.5.

**HRMS** (ESI) for C_33_H_42_N_2_O_5_SNa: calculated: 601.2702 [M+Na]^+^, found 601.2704.

[α]^20^_D_ = -6.0° (CHCl_3_, *c* = 1.0).

**Synthesis of 60:**

To a solution of **S45**(30.0 mg, 57.1 µmol) in acetonitrile (0.5 mL) at 0 °C was added *tert*-butyl nitrite (10 µL, 85.6 µmol, 1.5 eq.) and trimethylsilyl azide (9.0 µL, 68.5 µmol, 1.2 eq.).^[18]^ The cooling bath was removed and stirring at room temperature was continued for 1 h. The solvent was removed under reduced pressure. Column chromatography (PE/EtOAc 8:1 → 6:1, v/v) afforded a colorless oil foams **27** (R*_f_*= 0.5, PE:EA=3:1, v/v; 28.0, 52.2 µmol, 91%).

**((3a*S*,4*R*,6*R*,7a*S*)-2-((4-(*tert*-butyl) phenyl) sulfonyl)-5,5-dimethyloctahydro-3a*H*-4,6-methanoisoindol-3a-yl) methyl 1*H*-benzo[d][1,2,3]triazole-1-carboxylate (27):**

**^1^H NMR** (CDCl_3_, 500 MHz): δ = 8.13 (dt, *J =* 8.3, 1.0 Hz, 1 H, Ar-H), 8.06 (dt, *J =* 8.3, 1.0 Hz, 1 H, Ar-H), 7.69–7.76 (m, 2 H, 2 × Ar-H), 7.66 (ddd, *J =* 8.2, 7.1, 1.0 Hz, 1 H, Ar-H), 7.51 (ddd, *J =* 8.2, 7.1, 1.0 Hz, 1 H, Ar-H), 7.41–7.47 (m, 2 H, 2 × Ar-H), 4.46 (d, *J =* 10.5 Hz, 1 H, CH_B_),

4.36 (d, *J =* 10.6 Hz, 1 H, CH_A_), 3.47 (dd, *J =* 9.2, 6.8 Hz, 1 H, CH), 3.21 (d, *J =* 9.9 Hz, 1 H, CH), 3.15 (dd, *J =* 9.2, 3.0 Hz, 1 H, CH), 3.05 (d, *J =* 9.8 Hz, 1 H, CH), 2.41–2.47 (m, 1 H, CH), 2.37 (dddd, *J =* 12.5, 10.5, 3.2, 2.0 Hz, 1 H, CH), 2.27 (dtd, *J =* 10.5, 6.2, 1.9 Hz, 1 H, CH), 2.09 (dd, *J =* 6.4, 4.9 Hz, 1 H, CH), 1.92–1.97 (m, 1 H, CH), 1.73 (ddd, *J =* 13.2, 4.2, 2.8 Hz, 1 H, CH), 1.47 (d, *J =* 10.5 Hz, 1 H, CH), 1.26 (s, 9 H, 3 × CH_3_), 1.16 (s, 3 H, CH_3_), 1.06 (s, 3 H, CH_3_).

**^13^C NMR** (CDCl_3_, 125 MHz):δ = 156.6, 149.0, 145.9, 131.8, 131.7, 130.5, 128.1, 126.1, 126.0, 120.6, 113.4, 73.2, 58.3, 56.9, 50.5, 46.4, 40.1, 39.2, 35.1, 34.6, 34.1, 31.0, 27.3, 27.1, 23.5.

**HRMS** (ESI) for C_29_H_37_N_4_O_4_S: calculated 537.2530 [M+H]^+^, found 537.2534.

**General procedure for the synthesis of 81 and 100:**

**(3a*S*,4*R*,6*R*,7a*S*)-2-((2-bromo-4-(*tert*-butyl) phenyl)sulfonyl)-3a-(((*tert*-butyldimethylsilyl)oxy)methyl)-5,5-dimethyloctahydro-1*H*-4,6-methanoisoindole (S48)**

A solution of **S33** (90.1 mg, 0.2 mmol), Et_3_N (83.4 µL, 0.6 mmol, 3 eq.), and TBSCl (60.3 mg, 0.4 mmol, 2 eq.) in DMF (5 mL) was stirred for 20 minutes under an argon atmosphere, Afterwards, the reaction mixture was diluted with water and the product was extract with Et_2_O, The organic layer was washed with water and brine, dried over anhydrous NaSO_4_, the residue was purified by chromatography (PE:EA=15:1 to 10:1, v/v) to afford liquid product **S48** (R*_f_*= 0.6, PE:EA=10:1, v/v; 98.8 mg, 0.17 mmol, 85%).

**^1^H NMR** (700 MHz, CDCl_3_) δ 7.97 (d, *J =* 8.3 Hz, 1H), 7.71 (d, *J =* 1.9 Hz, 1H), 7.41 (dd, *J =* 8.4, 1.9 Hz, 1H), 3.44 (dd, *J =* 9.1, 6.9 Hz, 1H), 3.43 (d, *J =* 9.6 Hz, 1H), 3.31 (d, *J =* 9.6 Hz, 1H), 3.29 – 3.27 (m, 1H), 3.17 (d, *J =* 9.6 Hz, 1H), 3.10 (d, *J =* 9.6 Hz, 1H), 2.28 (dd, *J =* 13.1, 10.6 Hz, 1H), 2.25 – 2.17 (m, 2H), 1.88 (d, *J =* 6.2 Hz, 2H), 1.67 (ddd, *J =* 12.9, 4.9, 2.0 Hz, 1H), 1.46 (d, *J =* 10.3 Hz, 1H), 1.32 (s, 9H), 1.21 (s, 3H), 0.94 (s, 3H), 0.76 (s, 9H), -0.11 (d, *J =* 8.5 Hz, 6H).

**^13^C NMR** (176 MHz, CDCl_3_) δ 157.7, 134.3, 133.2, 132.4, 124.6, 120.8, 68.2, 58.9, 56.4, 52.9, 46.6, 40.5, 39.3, 35.2, 34.6, 34.5, 31.1, 27.6, 27.4, 26.0, 23.7, 18.2, -5.4, -5.5.

**HRMS** (ESI) for C_28_H_46_BrNO_3_SSiNa: calculated 606.2049 [M+Na]^+^, found 606.2040.

[α]^20^_D_ = -176.12° (CHCl_3_, *c* = 0.25).

**(3a*S*,4*R*,6*R*,7a*S*)-2-((4-(tert-butyl)-2-cyclopropylphenyl)sulfonyl)-3a-(((*tert*-butyldimethylsilyl)oxy)methyl)-5,5-dimethyloctahydro-1*H*-4,6-methanoisoindole (S49)**:

In a 10 mL sealed tube, **S49** (292.4 mg, 0.5 mmol), cyclopropylboronic acid (85.8 mg, 2.0 eq.

), K_3_PO_4_ （265.3 mg, 1.25 mmol), Pd(OAc)_2_ (11.3 mg, 10 mol%), P(Cy)_3_ (28 mg, 20 mol%) were added successively under Ar. Then, toluene/H_2_O = 10:1 (5:0.5 mL) was added. The tube was sealed with a teflon lined cap and the reaction mixture was stirred at 110 ˚C for 24 h in an oil bath. After cooling to room temperature, the mixture was concentrated under vacuum and the residue was purified by column chromatography on silica gel (PE:EA=20:1, v/v) to give the corresponding products **S49** (R*_f_*= 0.6, PE:EA=10:1, v/v; 98.8 mg, 0.17 mmol, 85%).

**^1^H NMR** (700 MHz, CDCl_3_) δ 7.83 (d, *J =* 8.4 Hz, 1H), 7.23 (dd, *J =* 8.4, 2.0 Hz, 1H), 6.85 (d, *J =* 2.0 Hz, 1H), 3.42 (d, *J =* 9.6 Hz, 1H), 3.29 (d, *J =* 9.6 Hz, 1H), 3.24 (dd, *J =* 8.8, 6.7 Hz, 1H), 3.14 (dd, *J =* 8.9, 2.2 Hz, 1H), 3.10 (d, *J =* 9.4 Hz, 1H), 3.05 (d, *J =* 9.4 Hz, 1H), 2.99 – 2.95 (m, 1H), 2.32 – 2.25 (m, 1H), 2.23 (ddt, *J =* 8.5, 6.1, 3.0 Hz, 1H), 2.17 (dtd, *J =* 10.3, 6.2, 2.0 Hz, 1H), 1.87 (d, *J =* 6.2 Hz, 2H), 1.62 (ddd, *J =* 13.1, 4.7, 2.3 Hz, 1H), 1.52 (d, *J =* 10.3 Hz, 1H), 1.28 (s, 9H), 1.21 (s, 3H), 1.10 – 1.08 (m, 2H), 0.94 (s, 3H), 0.81 – 0.78 (m, 2H), 0.76 (s, 9H), -0.10 (s, 3H), -0.13 (s, 3H).

**^13^C NMR** (176 MHz, CDCl_3_) δ 155.1, 142.7, 131.5, 129.5 121.1, 120.1, 67.1, 57.1, 55.3, 51.3, 45.6 39.3, 38.2, 34.0, 33.6, 33.3, 30.0, 26.4, 26.0, 24.8, 22.5, 17.1, 11.3, 10.5, 10.4, -6.5, -6.6.

**HRMS** (ESI) for C_28_H_46_BrNO_3_SSiNa: calculated 568.3257 [M+Na]^+^, found 568.3248.

[α]^20^_D_ = -72.2° (CHCl_3_, *c* = 0.5).

**((3a*S*,4*R*,6*R*,7a*S*)-2-((4-(*tert*-butyl)-2-cyclopropylphenyl)sulfonyl)-5,5-dimethyloctahydro-3a*H*-4,6-methanoisoindol-3a-yl)methyl (4-iodophenyl)carbamate (81):**

To a solution of **S49** ((169.2 mg, 0.3 mmol, 1.0 eq.) in anhydrous THF (6 mL) were added dropwise of 1.0 M TBAF solution in THF (0.39 mL, 0.39 mmol, 1.3 eq.). After stirring for 1 h at ambient temperature, the reaction was quenched with water, extracted with DCM, washed with brine, dried over MgSO4, and concentrated in vacuo. The residue were purified by column chromatography (EtOAc:Hexanes, gradient from 15:85 to 40:60, v/v) to afford the alcohol (quant.).

Following general procedure E2: From the alcohol (151.1 mg, 0.35 mmol) and 1-iodo-4-isocyanatobenzene (102.9 mg, 0.42 mmol, 1.2 eq.). Purification by column chromatography (PE/EtOAc = 20:1 to 10:1, v/v) afforded a colorless solid **81** (R*_f_*= 0.7, PE:EA=3:1, v/v; 170 mg, 0.25 mmol, 72%).

**^1^H NMR** (600 MHz, CD_2_Cl_2_)δ 7.84 (d, *J =* 8.4 Hz, 1H), 7.60 (d, *J =* 8.8 Hz, 2H), 7.27 (dd, *J =* 8.4, 2.0 Hz, 1H), 7.18 (d, *J =* 8.3 Hz, 2H), 6.89 (d, *J =* 2.0 Hz, 2H), 4.09 (s, 2H), 3.46 – 3.39 (m, 1H), 3.29 (d, *J =* 10.0 Hz, 1H), 3.04 (d, *J =* 10.0 Hz, 1H), 3.01 (dd, *J =* 9.6, 2.7 Hz, 1H), 2.89 – 2.84 (m, 1H), 2.36 – 2.26 (m, 2H), 2.19 (dtd, *J =* 10.4, 6.3, 1.6 Hz, 1H), 1.96 (dd, *J =* 6.5, 5.0 Hz, 1H), 1.88 (dt, *J =* 5.3, 2.8 Hz, 1H), 1.57 (dd, *J =* 9.0, 2.8 Hz, 1H), 1.35 (d, *J =* 10.4 Hz, 1H), 1.27 (s, 9H), 1.22 (s, 3H), 1.11 – 1.08 (m, 2H), 1.02 (s, 3H), 0.84 – 0.81 (m, 2H).

**^13^C NMR** (151 MHz, CD_2_Cl_2_) δ 157.2, 153.5, 144.3, 138.4, 138.3, 133.3, 130.7, 122.7, 121.7, 120.9, 86.3, 70.6, 58.0, 56.6, 51.0, 46.7, 40.6, 39.4, 35.4, 34.9, 34.4, 31.1, 27.4, 27.3, 23.5, 12.6, 11.7, 11.6.

**HRMS** (ESI) for C_32_H_41_IN_2_O_4_SNa: calculated 699.1729 [M+Na]^+^, found 699.1730.

[α]^20^_D_ = -22.1° (CHCl_3_, *c* = 1.0).

**((3a*S*,4*R*,6*R*,7a*S*)-2-((4-(*tert*-butyl)-2-cyclopropylphenyl)sulfonyl)-5,5-dimethyloctahydro-3aH-4,6-methanoisoindol-3a-yl)methyl (4-ethynylphenyl)carbamate (100)**:

Following general procedure F: From **81** (33.8 mg, 0.05 mmol) and trimethylsilylacetylene (14 µl,0.1 mmol, 2.0 eq.). Purification by column chromatography (PE/EtOAc 15:1 to 10:1 to 5:1, v/v) afforded a colorless solid (R*_f_*= 0.8, PE:EA=3:1, v/v; 60%).

**^1^H NMR (**500 MHz, CD_2_Cl_2_) δ 7.84 (d, *J =* 8.4 Hz, 1H), 7.47 – 7.40 (m, 2H), 7.36 (d, *J =* 8.6 Hz, 2H), 7.28 (dd, *J =* 8.4, 2.0 Hz, 1H), 6.93 (s, 1H), 6.89 (d, *J =* 2.0 Hz, 1H), 4.11 (s, 2H), 3.47 – 3.41 (m, 1H), 3.29 (d, *J =* 10.0 Hz, 1H), 3.09 (s, 1H), 3.05 (d, *J =* 10.2 Hz, 1H), 3.03 – 3.00 (m, 1H), 2.87 (tt, *J =* 8.4, 5.2 Hz, 1H), 2.35 – 2.28 (m, 2H), 2.20 (dtd, *J =* 10.4, 6.3, 1.6 Hz, 1H), 1.97 (dd, *J =* 6.5, 5.0 Hz, 1H), 1.89 (td, *J =* 5.3, 3.1 Hz, 1H), 1.57 (dd, *J =* 8.9, 2.8 Hz, 1H), 1.35 (d, *J =* 10.4 Hz, 1H), 1.28 (s, 9H), 1.23 (s, 3H), 1.10 (ddt, *J =* 9.3, 5.2, 2.1 Hz, 2H), 1.03 (s, 3H), 0.84 – 0.80 (m, 2H).

**^13^C NMR** (126 MHz, CD_2_Cl_2_)δ 157.2, 153.1, 144.3, 139.1, 133.3, 133.3, 130.7, 128.5, 122.7, 121.7, 118.6, 111.9, 83.6, 76.8, 70.6, 58.0, 56.7, 51.0, 46.7, 40.6, 39.4, 35.4, 34.9, 34.4, 31.1, 27.4, 27.3, 23.5, 14.4, 12.6, 11.7, 11.6.

**HRMS** (ESI) for C_34_H_42_N_2_O_4_SNa: calculated: 597.2763 [M+Na]^+^, found 597.2743.

[α]^20^_D_ = +18.7° (CHCl_3_, *c* = 0.25).

**Synthesis of 90 and 91:**

i) To a solution of ester (10 mmol, 1.00 eq.) in diethyl ether (0.25 M) was added LiAlH_4_ (22.5 mmol, 2.25 eq.) in four approximately equal portions over 10 minutes at 0 °C. The reaction was allowed to warm to room temperature and stirred for an additional 30 minutes. After TLC monitoring showed complete consumption of the methyl ester, the flask was returned to an ice bath and quenched with 1 M aqueous HCl. The primary alcohol product was extracted with diethyl ether (3 times). The combined organic layers were dried over anhydrous MgSO4, filtered, and concentrated under reduced pressure. The obtained alcohol was used in next step without further purification.

ii) To a solution of (COCl)_2_ (0.94 mL, 12.0 mmol) in DCM (26 mL) was added DMSO (1.7 mL, 24 mmol) at -78 °C. After being stirred for 30 min, the alcohol (1.43 mg, 9.5 mmol) was added to the reaction mixture. The resulting mixture was stirred for 1 h at the same temperature. Then, to the mixture was added Et_3_N (6.6 mL, 48 mmol), and warmed to room temperature. After being stirred for 1 h, the reaction was quenched by water, and extracted with DCM twice. The extract was washed with water and brine respectively, dried, and concentrated to afford the residue. Purification by column chromatography (PE to PE:EA=50:1, v/v) afforded the desired product (0.92g, 65%) as a colorless oil **S50**.

**2-methyl-2-phenylpropanal (S50):**

**^1^H NMR** (500 MHz, CDCl_3_) δ 9.51 (s, 1H), 7.41 – 7.36 (m, 2H), 7.31 – 7.27 (m, 3H), 1.47 (s, 6H).

**^13^C NMR** (126 MHz, CDCl_3_) δ 202.4, 141.4, 129.0, 127.4, 126.9, 50.6, 22.6.

iii) A solution of *n*-BuLi (4.7 mL, 7.5 mmol) was added slowly to a stirred suspension of (methoxymethyl)triphenylphosphonium chloride (2.57 g, 7.5 mmol) in THF (16 mL) at 0 °C. After stirring the dark red mixture at same temperature with 30 min, a solution of aldehyde **S50** (922.8 mg, 6.5 mmol) in THF (5 mL) was added. After stirring for 24 h, 2N HCl (1 mL of an aqueous solution) was added slowly to the yellow mixture which was stirred for further 3.5 h. NaHCO_3_ (saturated aqueous solution) was added, the crude product was extracted with DCM (2 times) and the combined extracts were dried over MgSO_4_. Purification by flash chromatography (gradient; 5 to 20% EtOAc/hexanes) gave a desired liquid product (R*_f_*= 0.8, PE:EA=3:1, v/v; **S51**, 108 mg, 11%).

**3-Methyl-3-phenylbutanal (S51):**

**^1^H NMR** (500 MHz, CDCl_3_) δ 9.51 (s, 1H), 7.41 – 7.37 (m, 2H), 7.35 (dd, *J =* 8.7, 6.9 Hz, 2H), 7.25 – 7.20 (m, 1H), 2.68 (d, *J =* 3.1 Hz, 2H), 1.47 (s, 6H).

**^13^C NMR** (126 MHz, CDCl_3_) δ 203.2, 147.5, 128.7, 126.5, 125.7, 56.7, 36.9, 29.5.

iv) **S51** (108 mg, 0.66 mmol) was added into a solution of sodium chlorite (4.62 mmol, 7 eq.), monobasic sodium phosphate (3.48 mmol, 5 eq.), and 2-methyl-2-butene (169 µL) in a 20:1 *tert*-butyl alcohol-water mixture (1 mL) at room temperature. After 30 min, the solvents were evaporated, and the residue was dissolved in 1 M hydrochloric acid and extracted with ether (3 times). The combined extracts were washed with brine, dried, filtered through silica gel, and evaporated to afford the crude carboxyl acid without further purification.

v) The yellow crude carboxylic acid was dissolved in MeCN (2 mL) and treated with an excess of MeI (2.0 eq.) and Cs_2_CO_3_ (3.0 eq.). The mixture was stirred at room temperature for 3 h. then water was added, the mixture was washed by EA for 3 times, the organic phase was washed by brine and then dried by NaSO_4_. The unconsumed MeI was removed by evaporation, and the reaction mixture was filtered through silica gel and evaporated, No other further purification was required for the crude ester.

vi) From the above obtained ester (96 mg, 0.5 mmol) to afford the desired methyl 3-(4-(chlorosulfonyl)phenyl)-3-methylbutanoate,^[15]^ which was used in next step immediately (68 mg, 50%).

vii) Following general procedure D2: From **11** (48.8 mg, 0.25 mmol) and methyl 3-(4-(chlorosulfonyl) phenyl)-3-methylbutanoate (0.25 mmol, 1.0 eq.). Purification by column chromatography (PE/EtOAc 4:1) afforded a colorless solid **S52** (R*_f_*= 0.3, PE:EA=2:1, v/v; 60.6 mg, 0.13 mmol, 54%).

**Methyl 3-(4-(((3a*S*,4*R*,6*R*,7a*S*)-3a-(hydroxymethyl)-5,5-dimethyloctahydro-2*H*-4,6-methanoisoindol-2-yl)sulfonyl)phenyl)-3-methylbutanoate (S52):**

**^1^H NMR** (500 MHz, CDCl_3_) δ 7.74 (d, *J =* 8.5 Hz, 2H), 7.51 (d, *J =* 8.5 Hz, 2H), 3.50 (s, 3H), 3.42 – 3.34 (m, 2H), 3.16 (dd, *J =* 9.0, 7.0 Hz, 1H), 3.03 (dd, *J =* 9.1, 3.0 Hz, 1H), 2.99 (d, *J =* 9.7 Hz, 1H), 2.90 (d, *J =* 9.7 Hz, 1H), 2.66 (d, *J =* 2.0 Hz, 2H), 2.30 – 2.24 (m, 1H), 2.24 – 2.15 (m, 2H), 1.89 (dd, *J =* 6.5, 5.0 Hz, 1H), 1.86 (dq, *J =* 5.3, 2.7 Hz, 1H), 1.62 (ddd, *J =* 12.8, 4.2, 2.6 Hz, 1H), 1.48 (s, 3H), 1.47 (s, 3H), 1.33 (d, *J =* 10.4 Hz, 1H), 1.20 (s, 3H), 0.93 (s, 3H).

**^13^C NMR** (126 MHz, CDCl_3_) δ 171.8, 153.3, 132.1, 128.2, 126.3, 68.1, 58.8, 57.0, 52.4, 51.5, 48.3, 46.2, 40.4, 39.2, 37.7, 34.3, 29.0, 28.9, 27.6, 27.5, 23.7.

**HRMS** (ESI) for C_24_H_35_NO_5_S: calculated: 472.2134 [M+Na]^+^, found 472.2122.

[α]^20^_D_ = -14.1° (CHCl_3_, *c* = 1.0).

**Methyl 3-(4-(((3a*S*,4*R*,6*R*,7a*S*)-3a-((((4-iodophenyl)carbamoyl)oxy)methyl)-5,5-dimethyloctahydro-2*H*-4,6-methanoisoindol-2-yl)sulfonyl)phenyl)-3-methylbutanoate (90):**

Following general procedure E2: From **S52** (22.5 mg, 0.05 mmol) and 1-iodo-4-isocyanatobenzene (22 mg, 1.5eq.). Purification by column chromatography (PE/EtOAc 20:1 to 10:1, v/v) afforded a colorless solid **90** (R*_f_*= 0.7, PE:EA=3:1, v/v; 0.03 mmol, 60%).

**^1^H NMR** (700 MHz, CDCl_3_) δ 7.74 (d, *J =* 8.5 Hz, 2H), 7.58 (d, *J =* 8.7 Hz, 2H), 7.52 (d, *J =* 8.5 Hz, 2H), 7.14 (d, *J =* 8.3 Hz, 2H), 6.86 (s, 1H), 4.30 (d, *J =* 11.0 Hz, 1H), 3.78 (s_br_, 1H), 3.45 (s, 3H), 3.10 (s, 1H), 3.08 (d, *J =* 2.5 Hz, 1H), 3.02 (dd, *J =* 9.1, 6.6 Hz, 1H), 2.90 (d, *J =* 9.7 Hz, 1H), 2.75 (d, *J =* 15.1 Hz, 1H), 2.65 (d, *J =* 15.1 Hz, 1H), 2.34 (dd, *J =* 5.8, 2.9 Hz, 1H), 2.33 – 2.30 (m, 1H), 2.24 – 2.20 (m, 1H), 1.91 – 1.85 (m, 2H), 1.65 – 1.62 (m, 1H), 1.54 (s, 3H), 1.49 (s, 3H), 1.46 (s, 1H), 1.44 (d, *J =* 2.0 Hz, 3H), 1.23 (s, 3H), 1.04 (s, 3H).

**^13^C NMR** (176 MHz, CDCl_3_) δ 171.9, 153.6, 153.5, 138.0, 137.9, 132.3, 128.2, 126.3, 121.1, 86.6, 69.9, 58.5, 56.8, 51.6, 50.9, 47.9, 47.0, 40.2, 39.2, 37.6, 34.5, 34.2, 29.7, 28.6, 27.4, 27.1, 23.5.

**HRMS** (ESI) for C_31_H_39_IN_2_O_6_SNa: calculated: 717.1471 [M+Na]^+^, found 717.1477.

[α]^20^_D_ = +64.0° (CHCl_3_, *c* = 0.5).

**3-(4-(((3a*S*,4*R*,6*R*,7a*S*)-3a-((((4-iodophenyl)carbamoyl)oxy)methyl)-5,5-dimethyloctahydro-2*H*-4,6-methanoisoindol-2-yl)sulfonyl)phenyl)-3-methylbutanoic acid (91):**

To a solution of **S52** (0.08 mmol, 1.0 eq.) in ethanol (0.1 M), cooled in an ice bath, a solution of sodium hydroxide (10 mg, 0.24 mmol, 3 eq.) in ethanol (0.1 M) was added dropwise. The resulting mixture was refluxed for 6 h, and then the solution was acidified with 2 N HCl to pH = 2. Extraction of the resulting mixture with diethyl ether, drying of the combined organic extracts over magnesium sulfate and evaporation of the solvent afforded carboxylic acid without purification for next step.

Following general procedure E3: From the crude carboxylic acid(0.08 mmol) and 1-iodo-4-isocyanatobenzene (22.1 mg, 1.2 eq). Purification by column chromatography (DCM:MeOH = 50:1 to 20:1, v/v) afforded a colorless oil foams **91** (R*_f_*= 0.5, DCM:MeOH=3:1, v/v; 0.04 mmol, totally 50 % for 2 steps).

**^1^H NMR** (700 MHz, Methylene Chloride-*d*_2_) δ 7.70 (s_br_, 2H), 7.64 (d, *J =* 8.3 Hz, 2H), 7.57 (d, *J =* 8.3 Hz, 2H), 7.15 (s_br_, 2H), 6.85 (s, 1H), 4.01 – 3.86 (m, 2H), 3.12 (d, *J =* 9.2 Hz, 1H), 3.09 (d, *J =* 10.0 Hz, 1H), 2.73 (s, 2H), 2.70 – 2.64 (m, 2H), 2.35 (d, *J =* 12.3 Hz, 1H), 2.26 (dq, *J =* 11.9, 7.0 Hz, 2H), 2.02 – 1.96 (m, 1H), 1.91 (tt, *J =* 5.6, 2.8 Hz, 1H), 1.69 – 1.64 (m, 1H), 1.49 (s, 7H), 1.26 (s, 3H), 1.02 (s, 3H).

**^13^C NMR** (176 MHz, CD_2_Cl_2_) δ 173.5, 153.7, 148.9, 138.3, 138.0, 132.2, 128.2, 126.9, 121.1, 86.8, 70.3, 58.4, 57.4, 54.2, 51.0, 47.9, 46.8, 40.5, 39.4, 37.9, 34.7, 34.6, 29.0, 27.4, 27.3, 23.4.

**HRMS** (ESI) for C_30_H_37_IN_2_O_6_SNa: calculated: 703.1315 [M+Na]^+^, found 703.1315.

[α]^20^_D_ = + 40.0° (CHCl_3_, *c* = 0.5).

# **X-Ray Structure Analysis of 18**

The crystal structure of compound **18** was determined using a *Bruker D8 Venture* four-circle diffractometer equipped with a *PHOTON II CPAD detector* (Bruker AXS GmbH). X-ray radiation was generated by the IμS microfocus source with either Cu (λ = 1.54178 Å) or Mo (λ = 0.71073 Å) radiation (Incoatec GmbH), equipped with HELIOS mirror optics and a single-hole collimator (Bruker AXS GmbH). A suitable single crystal of **18** was coated with perfluoropolyalkyl ether (inert oil) and mounted on a MicroMount (MiTeGen).

Data were collected using the APEX4 Suite (v.2021.10-0), incorporating SAINT for data integration and SADABS for absorption correction (Bruker AXS GmbH). The processing and finalization of the crystal structure and refinement were carried out using Olex2.^[19]^ The structure was solved with ShelXT^[20]^ using intrinsic phasing and further refined with ShelXL^[21]^ refinement package via least-squares minimization.

Non-hydrogen atoms were refined anisotropically. Carbon-bound hydrogen atoms were placed in idealized positions and refined using a riding model with fixed isotropic displacement parameters: C–H = 0.95–1.00 Å, *U*iso(H) = 1.5 *U*eq for CH₃ and 1.2*U*eq for CH₂ and CH groups. The N-bound hydrogen atoms on N1 were located from the difference Fourier map and refined independently.

Crystallographic data for compound **18** has been deposited with the **Cambridge Crystallographic Data Centre (CCDC)** under **deposition number 2472577**. These data are available free of charge from the CCDC via [www.ccdc.cam.ac.uk](https://www.ccdc.cam.ac.uk), or by contacting CCDC at 12 Union Road, Cambridge CB2 1EZ, UK (fax: +44-(0)1223-336033; email: [deposit@ccdc.cam.ac.uk](mailto:deposit@ccdc.cam.ac.uk)).

Recrystallization of compound **18** was carried out at 23 °C in dichloromethane via slow vapor-phase exchange with *n*-pentane.

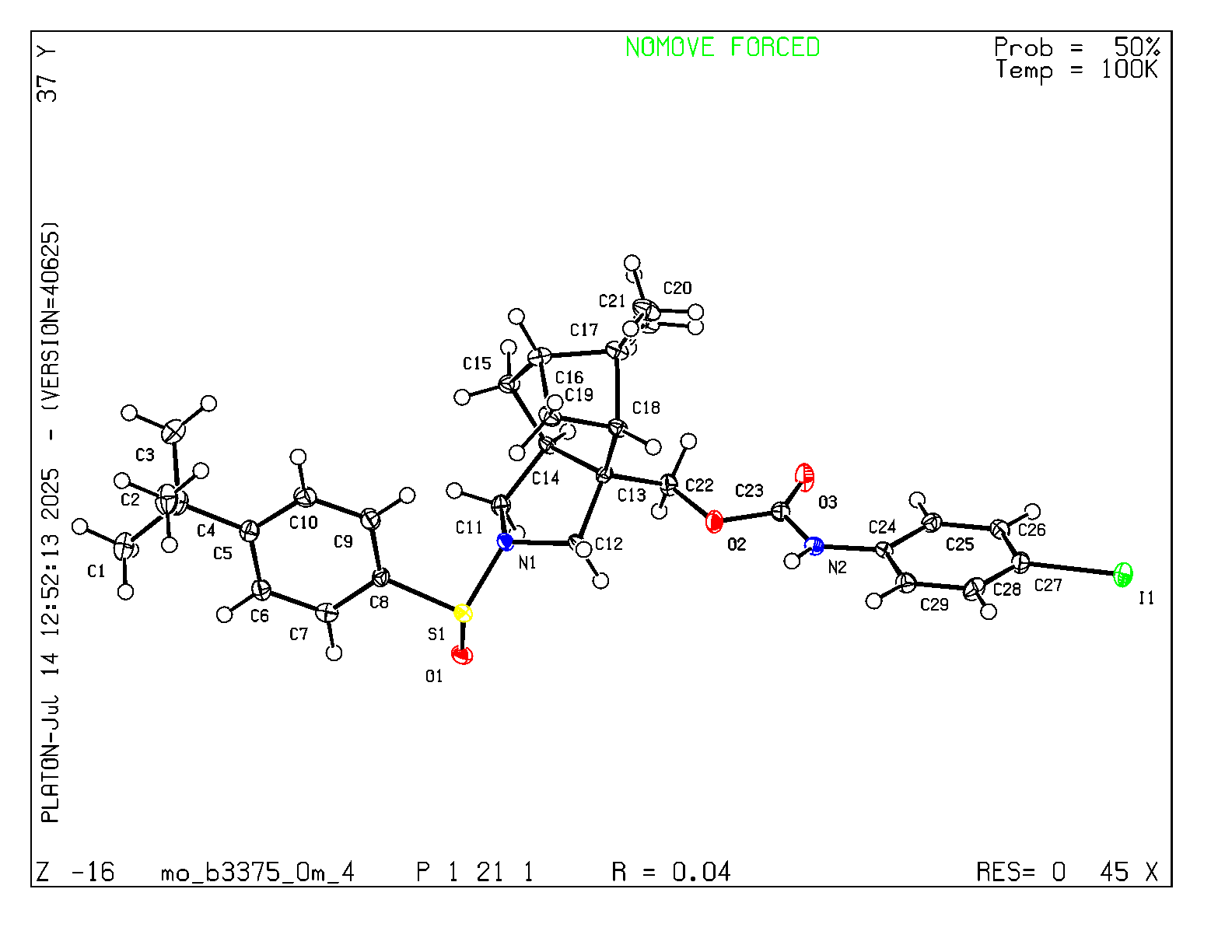


## **Fig. S14: X-ray crystal structure of 18.**

Ellipsoid plot of **18** [C_29_H_37_IN_2_O_3_S (M =620,56 g/mol)] at the 50% probability level. See Supplementary Table 4 for additional details. Crystallographic data have been deposited at the Cambridge Crystallographic Data Centre and copies can be obtained on request, free of charge, by quoting the publication citation and the deposition number CCDC: 2472577.

##

## **Table S4:**Crystal data and structure refinement for **18**.

| Empirical formula | C_29_H_37_IN_2_O_3_S |
| --- | --- |
| Formula weight | 620.56 |
| Temperature/K | 100.00 |
| Crystal system | monoclinic |
| Space group | *P*2_1_ |
| *a*/Å | 7.6277(3) |
| *b*/Å | 13.5521(5) |
| *c*/Å | 14.0074(6) |
| *α*/° | 90 |
| *β*/° | 102.533(2) |
| *γ*/° | 90 |
| Volume/Å^3^ | 1413.46(10) |
| Z | 2 |
| *ρ*_calc_g/cm^3^ | 1.458 |
| *μ*/mm^‑1^ | 1.239 |
| F(000) | 636.0 |
| Crystal size/mm^3^ | 0.31 × 0.163 × 0.106 |
| Radiation | MoK*α* (*λ* = 0.71073) |
| 2*Θ* range for data collection/° | 4.232 to 57.43 |
| Index ranges | -10 ≤ h ≤ 10, -18 ≤ k ≤ 18, -18 ≤ l ≤ 18 |
| Reflections collected | 7325 |
| Independent reflections | 7325 [*R*_int_ = 0.0829, *R*_sigma_ = 0.0421] |
| Data/restraints/parameters | 7325/1/338 |
| Goodness-of-fit on F^2^ | 1.094 |
| Final *R* indexes [*I*>=2*σ* (I)] | *R*_1_ = 0.0410, w*R*_2_ = 0.0737 |
| Final *R* indexes [all data] | *R*_1_ = 0.0508, w*R*_2_ = 0.0774 |
| Largest diff. peak/hole / e Å^-3^ | 0.53/-1.28 |
| Flack parameter | -0.023(11) |

**Pharmacokinetic and Tolerability analysis**

In vivo pharmacology characterization was carried out at Pharmacelsus GmbH, Saarbrücken, Germany, using male C57Bl/6 mice. Food and water was provided ad libitum before and during each study. All experimental procedures were approved by and conducted in accordance with the regulations of the local Animal Welfare Authorities. Plasma samples were analysed by liquid chromatography tandem-mass spectrometry using a generic method. Pharmacokinetic parameters were calculated by non-compartmental analysis. For determination of the pharmacokinetic parameters, iDeg-6 was administered at 1 mg/kg as IV bolus in 20% DMSO/ 80% PEG400. A dose escalation study was performed for PO and IP administration over 3-5 days with iDeg-6 formulated in 5% DMSO/ 30% PEG400/ 65% of an aqueous solution containing 30% (w/v) HPβCD and 0.5% (w/v) HPMC. Up to 120 mg/kg PO and 30 mg/kg IP no clinical signs were observed. At 60 mg/kg IP individual animals showed piloerection, subdued behavior shortly after iDeg-6 administration. Due to the larger plasma exposure of iDeg-6 the IP route was selected for efficacy studies.

Pharmacokinetic (PK) data for iDeg-6.

|  | PK parameters following 1 mg/kg IV | | | | | PK parameters following 30 mg/kg IP | | | |
| --- | --- | --- | --- | --- | --- | --- | --- | --- | --- |
|  | t_1/2_ [h] | C_0_ [ng/mL] | AUC_0-inf_ [ng/mL*h] | V_z_  [l/kg] | Cl [l/h/kg] | t_1/2_  [h] | C_max_ [ng/mL] | AUC_0-inf_ [ng/mL*h] | F  [%] |
| iDeg-6 | 1.1 | 1124 | 414 | 3.82 | 2.42 | 4.0 | 872 | 7036 | 57 |

# **Representative NOESY Correlations for The Determination of Diastereomers**

**((3aR,4S,6S,7aR)-2-((4-(*tert*-butyl)phenyl)sulfonyl)-5,5-dimethyloctahydro-3*aH*-4,6-methanoisoindol-3a-yl)methyl (4-iodophenyl)carbamate (20):**

The **H** that connected with **C**^7a^ in compound **20** was identified by COSY:

**((3aR,4aS,5aR,6aR)-2-((4-(tert-butyl)phenyl)sulfonyl)-5,5-dimethyloctahydrocyclopropa[f]isoindol-3a(1H)-yl)methyl (4-iodophenyl)carbamate (21):**

The **H** that connected with **C**^6a^ in compound **21** was identified by COSY:

**(1R,2S,3R,5R)-1'-((4-(*tert*-butyl)phenyl)sulfonyl)-6,6-dimethylspiro[bicyclo[3.1.1]heptane-2,3'-pyrrolidin]-3-yl (4-iodophenyl)carbamate (22):**

The connection of compound **22** was identified:

**(1S,2R,3R,5S)-1'-((4-(*tert*-butyl)phenyl)sulfonyl)-5-isopropylspiro[bicyclo[3.1.0]hexane-2,3'-pyrrolidin]-3-yl (4-iodophenyl)carbamate (23):**

The key connection of **23** was identified by COSY：

**(1S,2R,3R,5S)-1'-((4-(*tert*-butyl)phenyl)sulfonyl)-5-isopropylspiro[bicyclo[3.1.0]hexane-2,3'-pyrrolidin]-3-yl (4-iodophenyl)carbamate (24):**

The key connection of **24** was identified by COSY：

**Methyl (1S,3S,3aS,4R,6R,7aS)-2-((4-(tert-butyl)phenyl)sulfonyl)-3a-((((4-iodophenyl)carbamoyl)oxy)methyl)-5,5-dimethyl-3-phenyloctahydro-1H-4,6-methanoisoindole-1-carboxylate (27):**

The key connection of **27** was identified by COSY：

**((3aS,4R,6R,7aS)-2-((4-(*tert*-butyl)-2-methylphenyl)sulfonyl)-5,5-dimethyloctahydro-3aH-4,6-methanoisoindol-3a-yl)methyl (4-ethynylphenyl)carbamate (98 (iDeg-6)):**

# **References**

[1] V. I. Savych, V. L. Mykhalchuk, P. V. Melnychuk, A. O. Isakov, T. Savchuk, V. M. Timoshenko, S. A. Siry, S. O. Pavlenko, D. V. Kovalenko, O. V. Hryshchuk, V. A. Reznik, B. A. Chalyk, V. S. Yarmolchuk, E. B. Rusanov, P. K. Mykhailiuk, *J. Org. Chem.* **2021**, *86*, 13289-13309.

[2] S. X. Ge, D. Jung, R. Yao, *Bioinformatics* **2020**, *36*, 2628-2629.

[3] E. Hennes, P. Lampe, L. Dotsch, N. Bruning, L. M. Pulvermacher, S. Sievers, S. Ziegler, H. Waldmann, *Angew. Chem. Int. Ed. Engl.* **2021**, *60*, 9869-9874.

[4] K. J. Livak, T. D. Schmittgen, *Methods* **2001**, *25*, 402-408.

[5] T. K. Littlejohn, O. Takikawa, D. Skylas, J. F. Jamie, M. J. Walker, R. J. Truscott, *Protein Expr. Purif.* **2000**, *19*, 22-29.

[6] W. Kabsch, *Acta Cryst. D* **2010**, *66*, 125-132.

[7] A. J. McCoy, R. W. Grosse-Kunstleve, P. D. Adams, M. D. Winn, L. C. Storoni, R. J. Read, *J. Appl. Crystallogr.* **2007**, *40*, 658-674.

[8] D. Liebschner, P. V. Afonine, M. L. Baker, G. Bunkoczi, V. B. Chen, T. I. Croll, B. Hintze, L. W. Hung, S. Jain, A. J. McCoy, N. W. Moriarty, R. D. Oeffner, B. K. Poon, M. G. Prisant, R. J. Read, J. S. Richardson, D. C. Richardson, M. D. Sammito, O. V. Sobolev, D. H. Stockwell, T. C. Terwilliger, A. G. Urzhumtsev, L. L. Videau, C. J. Williams, P. D. Adams, *Acta Cryst. D* **2019**, *75*, 861-877.

[9] P. Emsley, B. Lohkamp, W. G. Scott, K. Cowtan, *Acta Cryst. D* **2010**, *66*, 486-501.

[10] X.-F. Cheng, T. Yu, Y. Liu, N. Wang, Z. Chen, G.-L. Zhang, L. Tong, B. Tang, *Org. Lett.* **2022**, *24*, 2087-2092.

[11] G. Buchi, W. Hofheinz, P. J. V., *J. Am. Chem. Soc.* **1969**, *91*, 6473.

[12] F. L. Haut, C. Habiger, K. Speck, K. Wurst, P. Mayer, J. N. Korber, T. Müller, T. Magauer, *J. Am. Chem. Soc.* **2019**, *141*, 13352-13357.

[13] A. V. Malkov, I. R. Baxendale, M. Bella, V. Langer, J. Fawcett, D. R. Russell, D. J. Mansfield, M. Valko, P. Kocovsky, *Organometallics* **2001**, *20*, 673-690.

[14] L. A. Paquette, R. J. Ross, Y. J. Shi, *J. Org. Chem.* **1990**, *55*, 1589-1598.

[15] L.-J. Zhong, Z.-Q. Xiong, X.-H. Ouyang, Y. Li, R.-J. Song, Q. Sun, X. Lu, J.-H. Li, *J. Am. Chem. Soc.* **2022**, *144*, 339-348.

[16] S. N. Bhattacharya, C. Eaborn, D. R. M. Walton, *J. Chem. Soc. C* **1968**, 1265-1267.

[17] E. Hennes, B. Lucas, N. S. Scholes, X.-F. Cheng, D. C. Scott, M. Bischoff, K. Reich, R. Gasper, M. Lucas, T. T. Xu, L. M. Pulvermacher, L. Dotsch, H. Imrichova, A. Brause, K. R. Naredla, S. Sievers, K. Kumar, P. Janning, M. Gersch, P. J. Murray, B. A. Schulman, G. E. Winter, S. Ziegler, H. Waldmann, *bioRxiv* **2025**.

[18] K. Barral, A. D. Moorhouse, J. E. Moses, *Org. Lett.* **2007**, *9*, 1809-1811.

[19] L. J. B. O. V. Dolomanov, R. J. Gildea, J. A. K. Howard, H. Puschmann, *J. Appl. Crystallogr.* **2009**, *42*, 339.

[20] G. M. Sheldrick, *Acta Cryst. A* **2014**, *71*, 3-8.

[21] G. M. Sheldrick, *Acta Cryst. C* **2015**, *71*, 3-8.

# **Publication Licenses**

# **
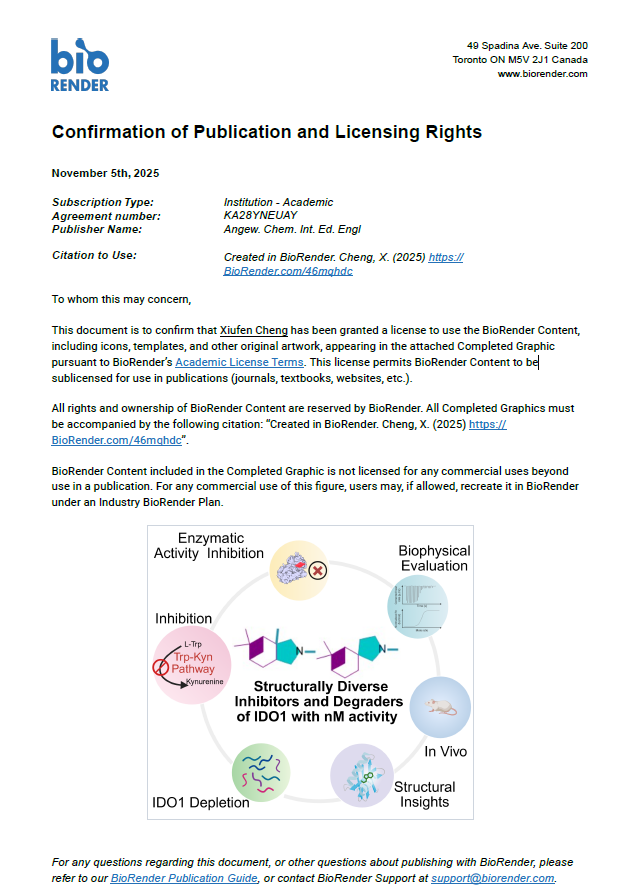
**

# **
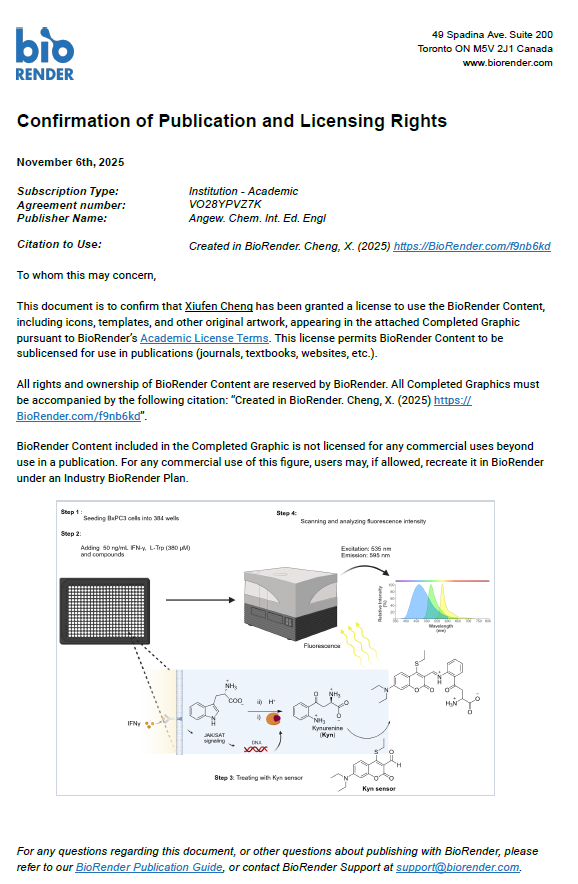
**

# **
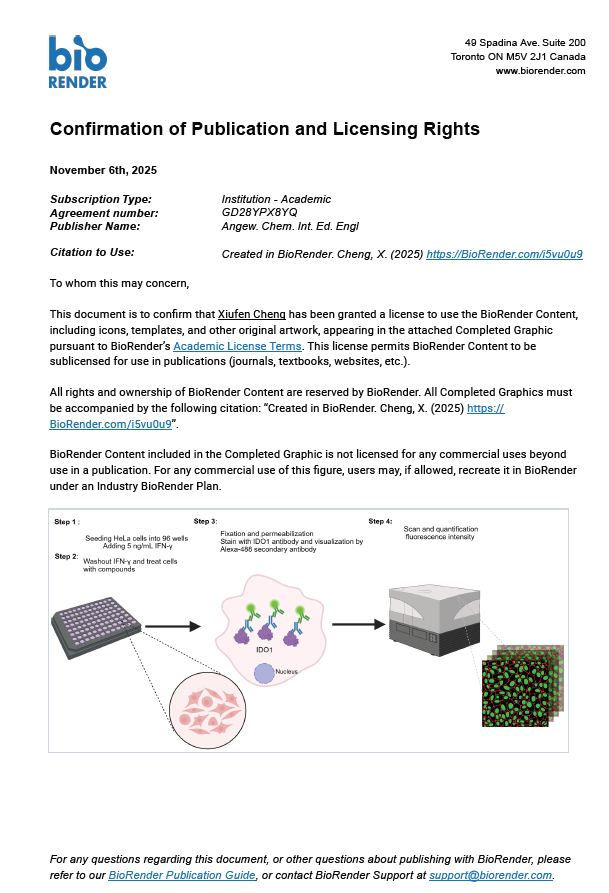
**

# **NMR** **Spectra**


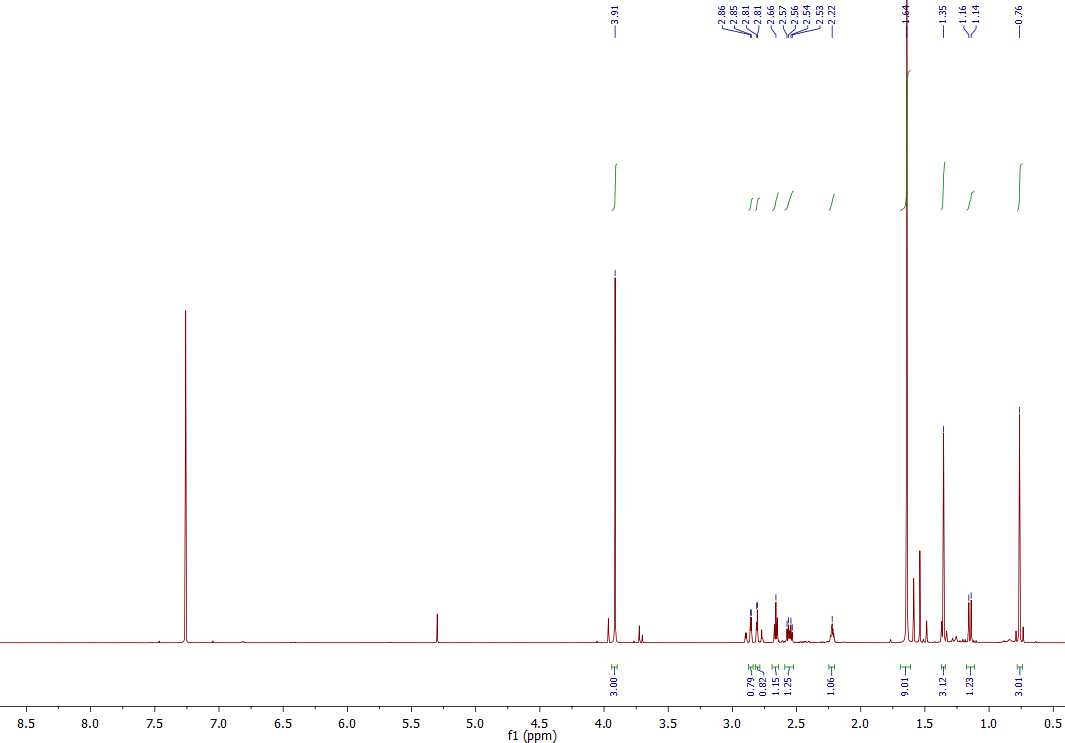


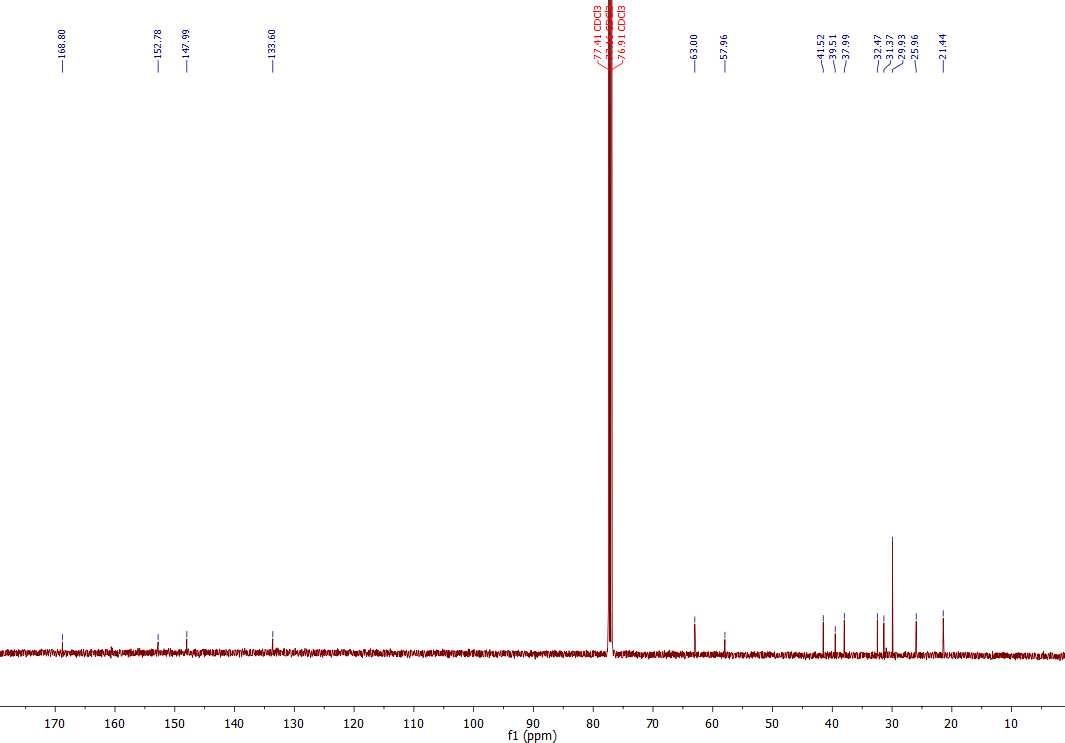


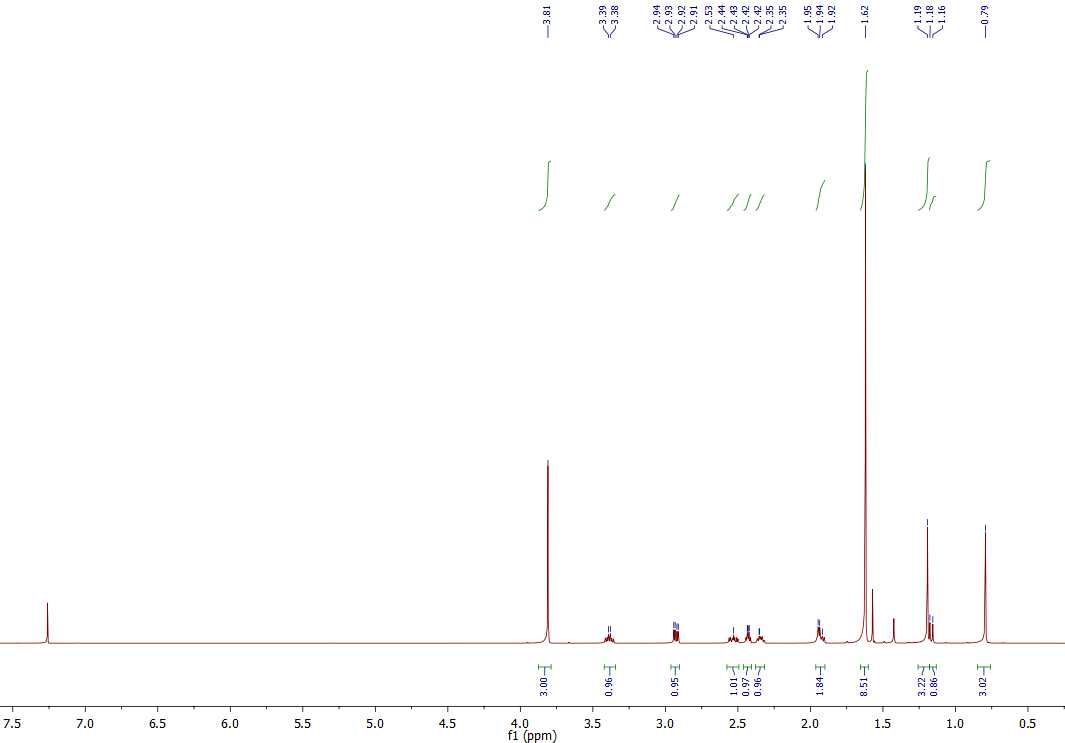


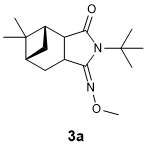

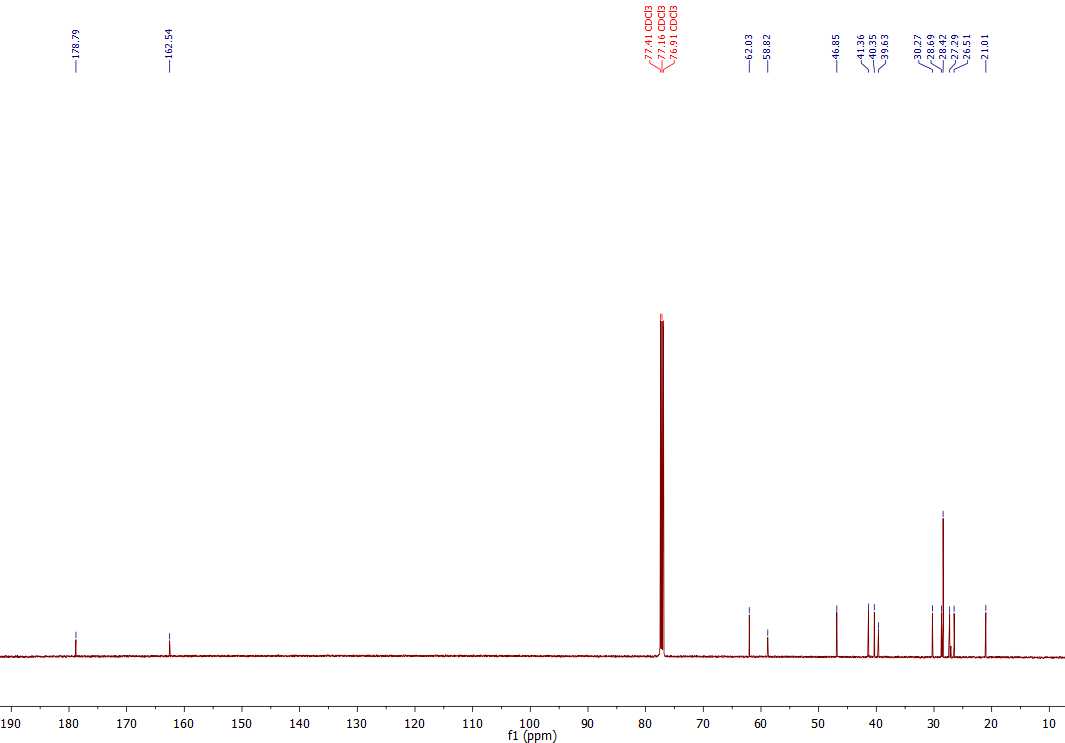


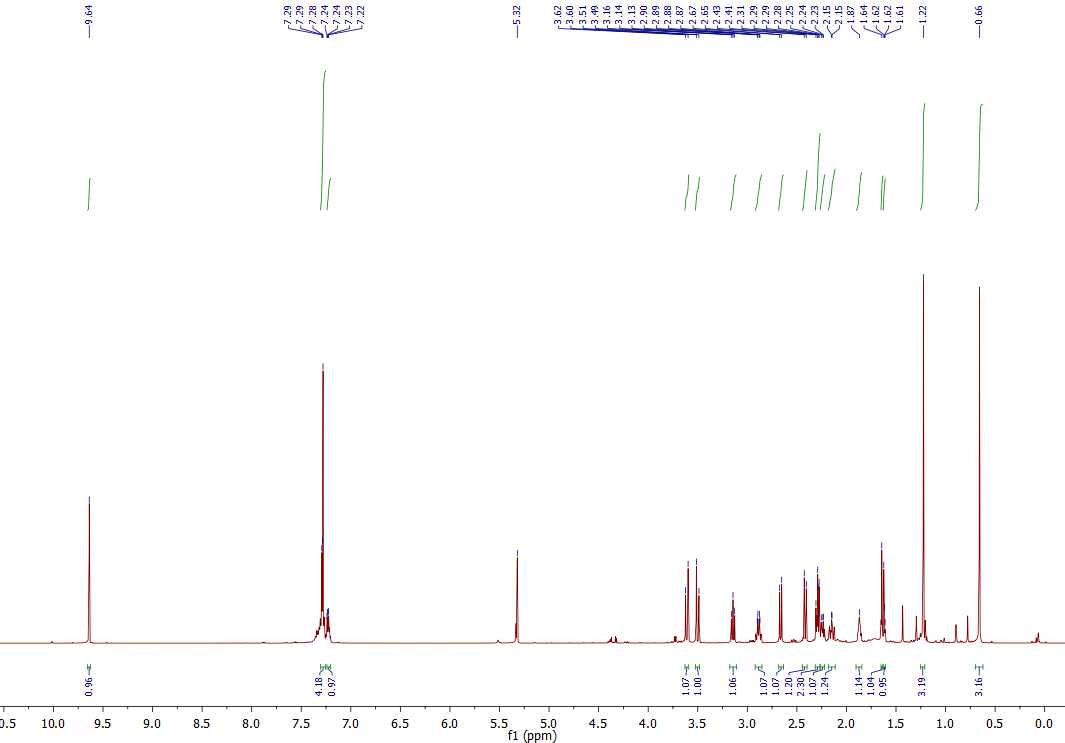


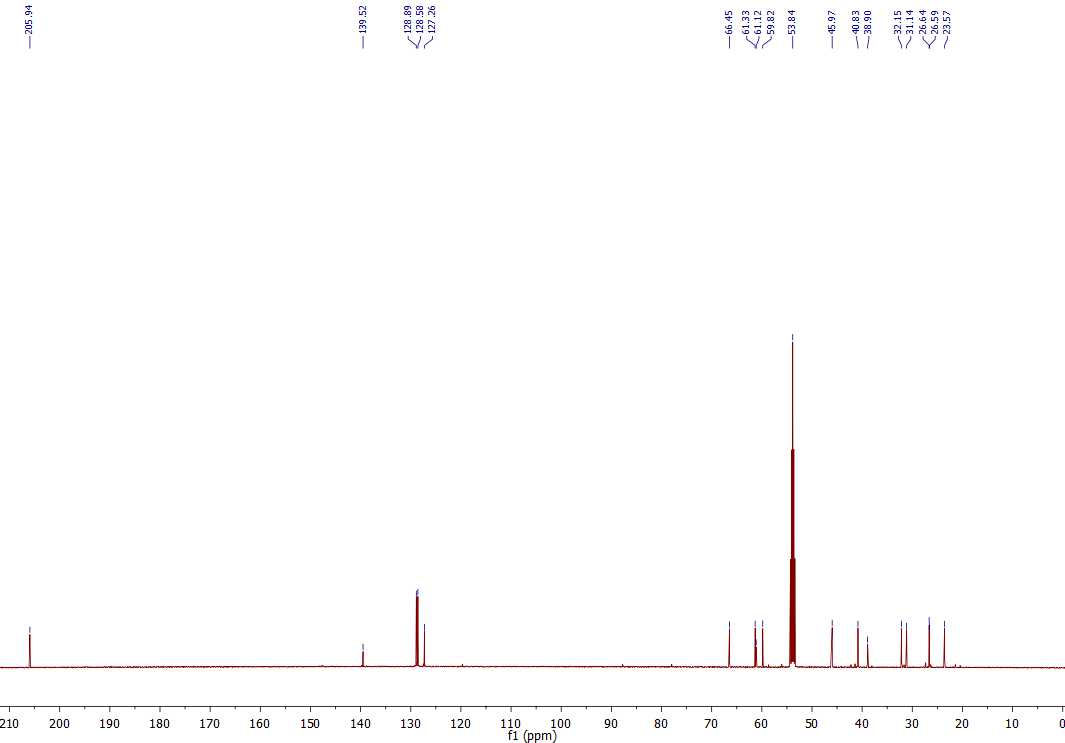


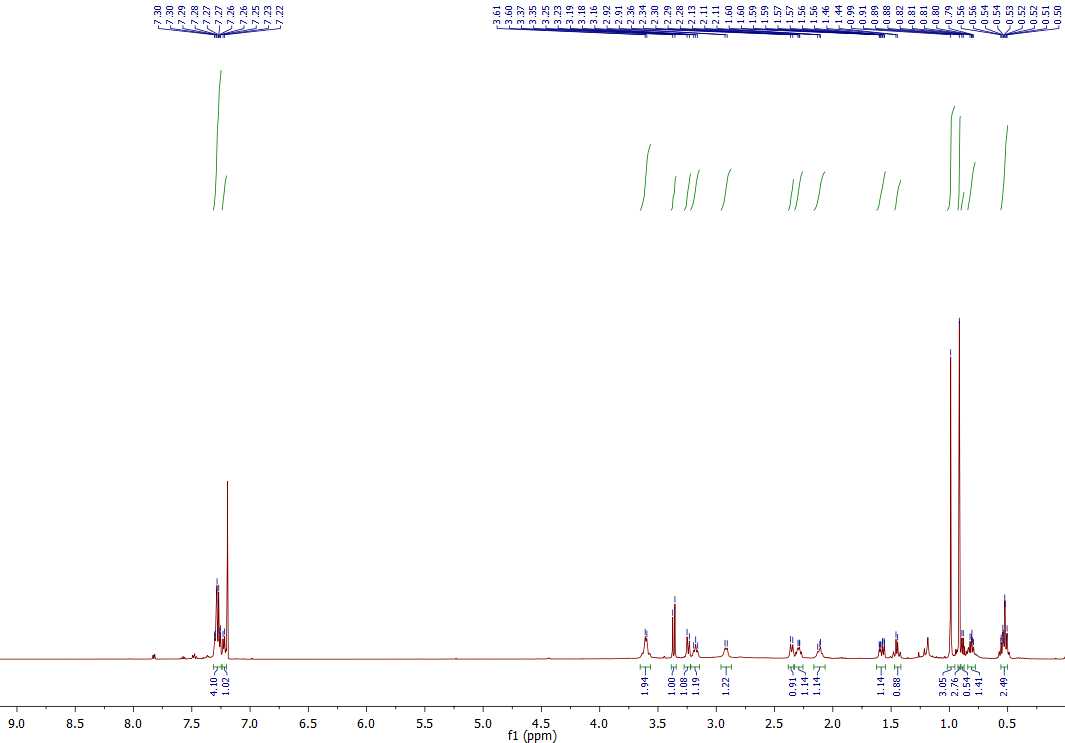


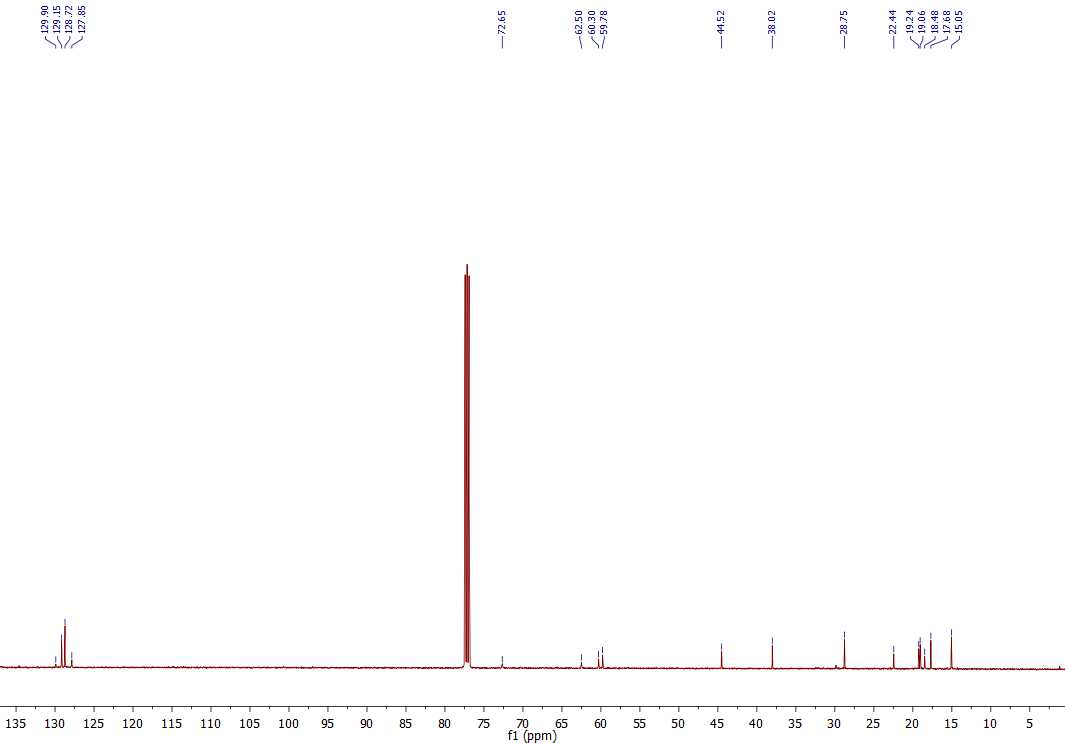


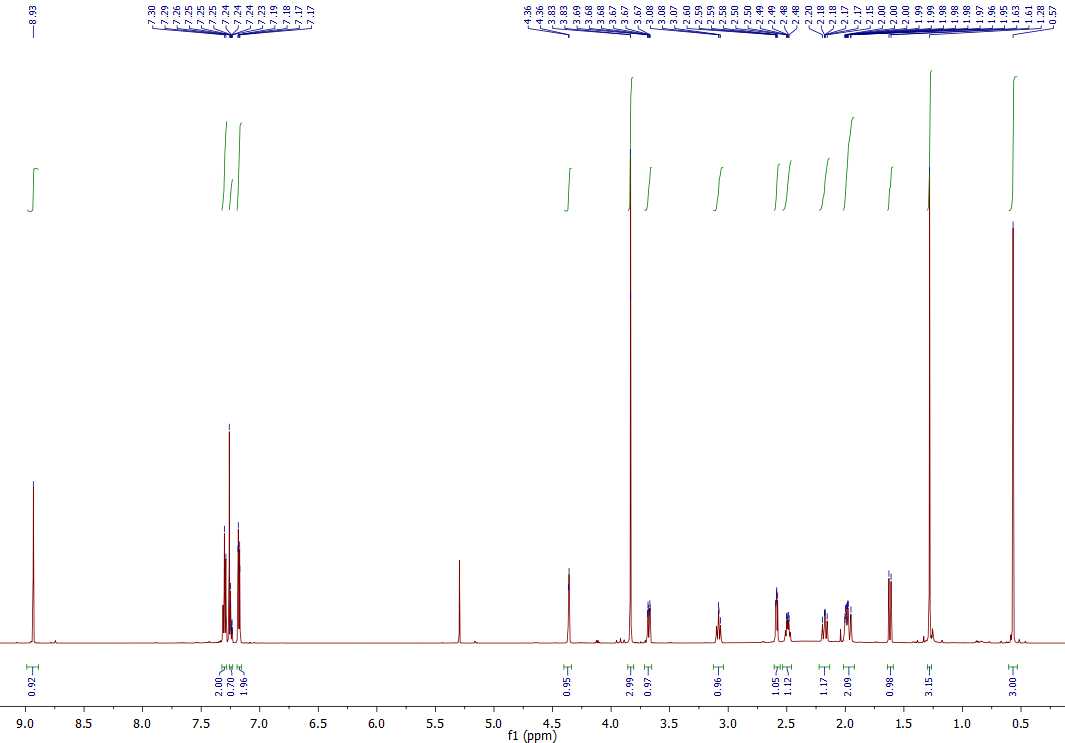


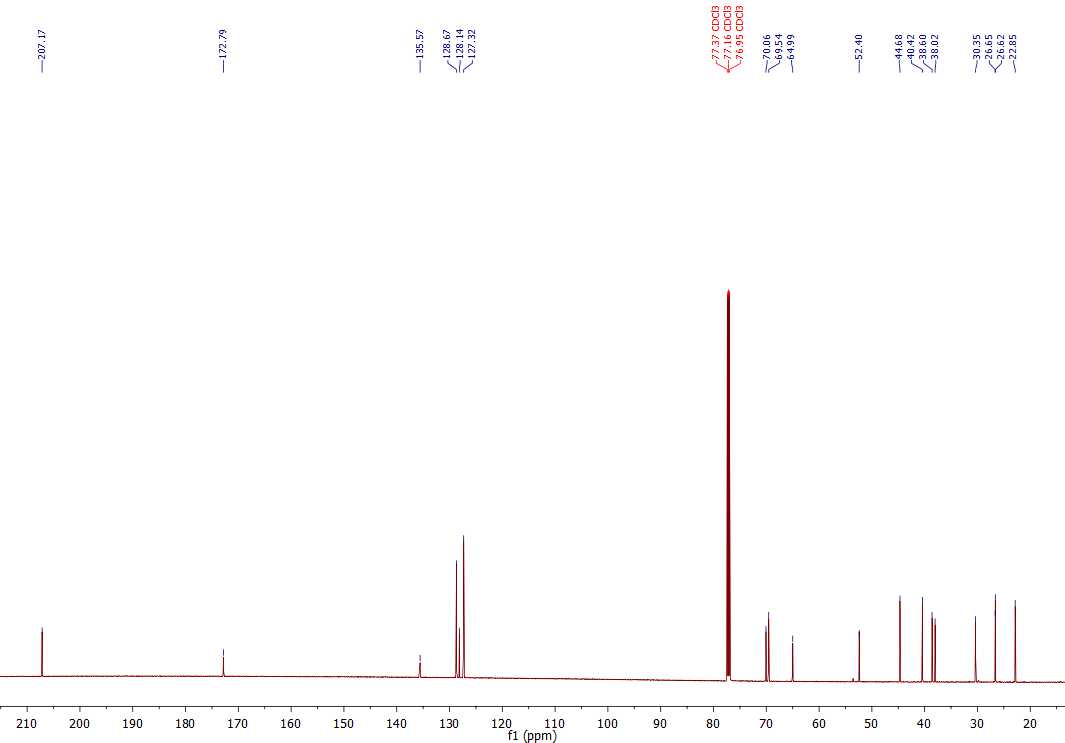


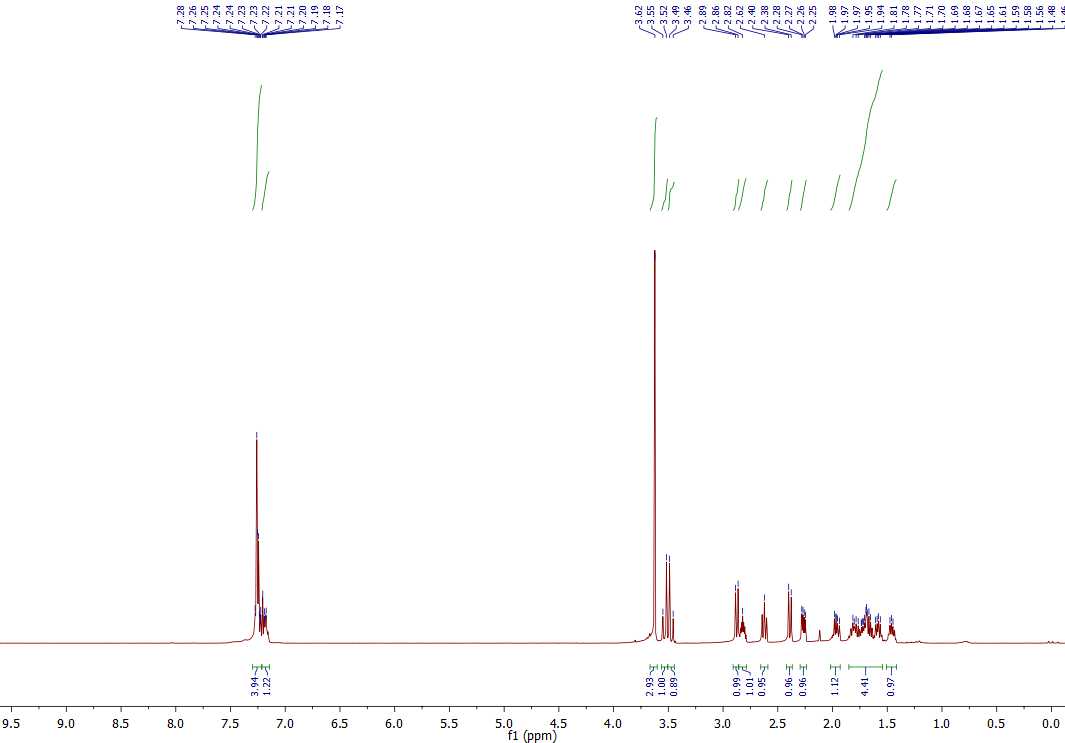


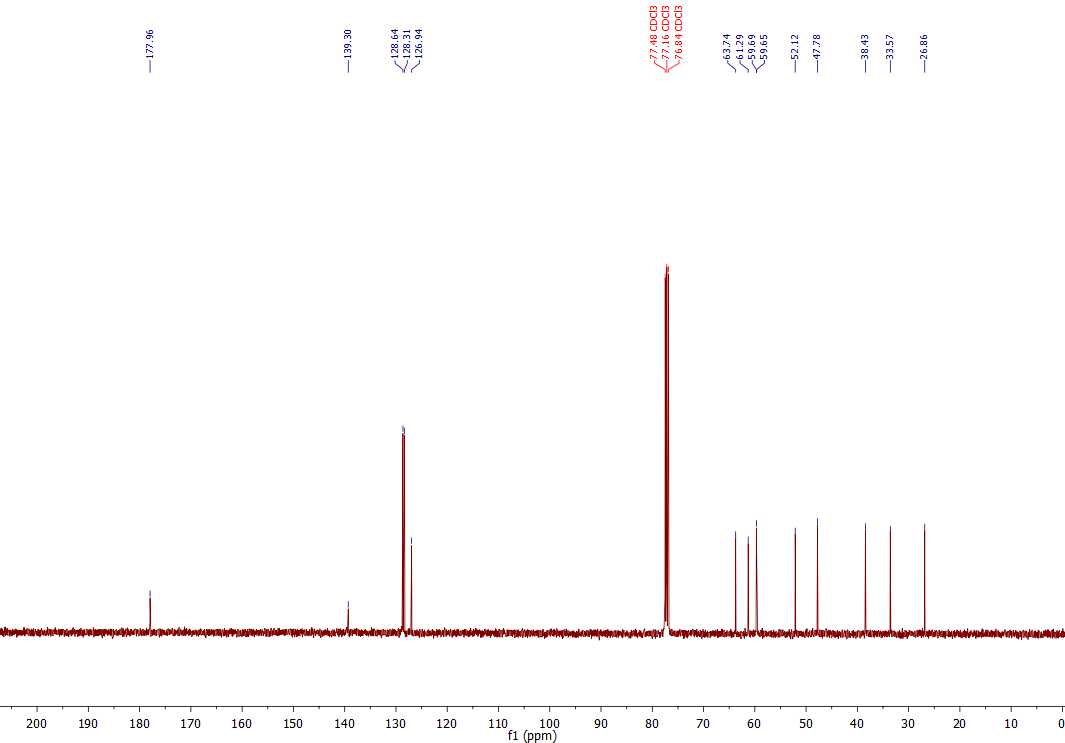


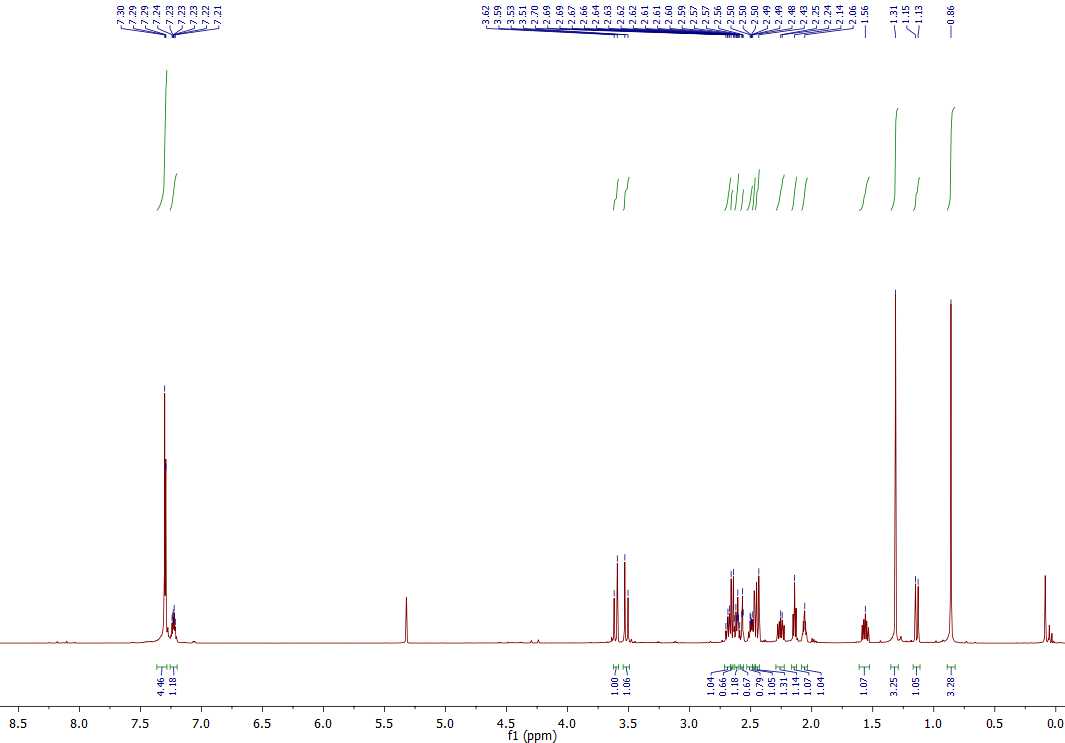


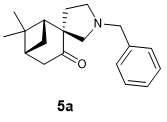

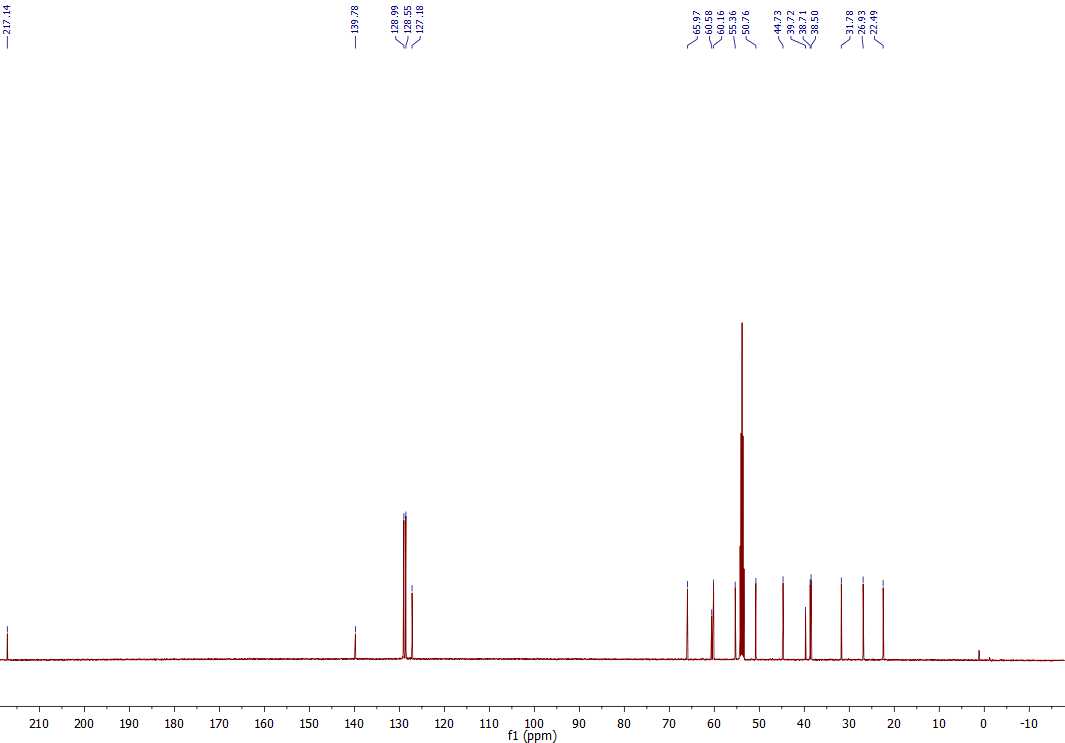


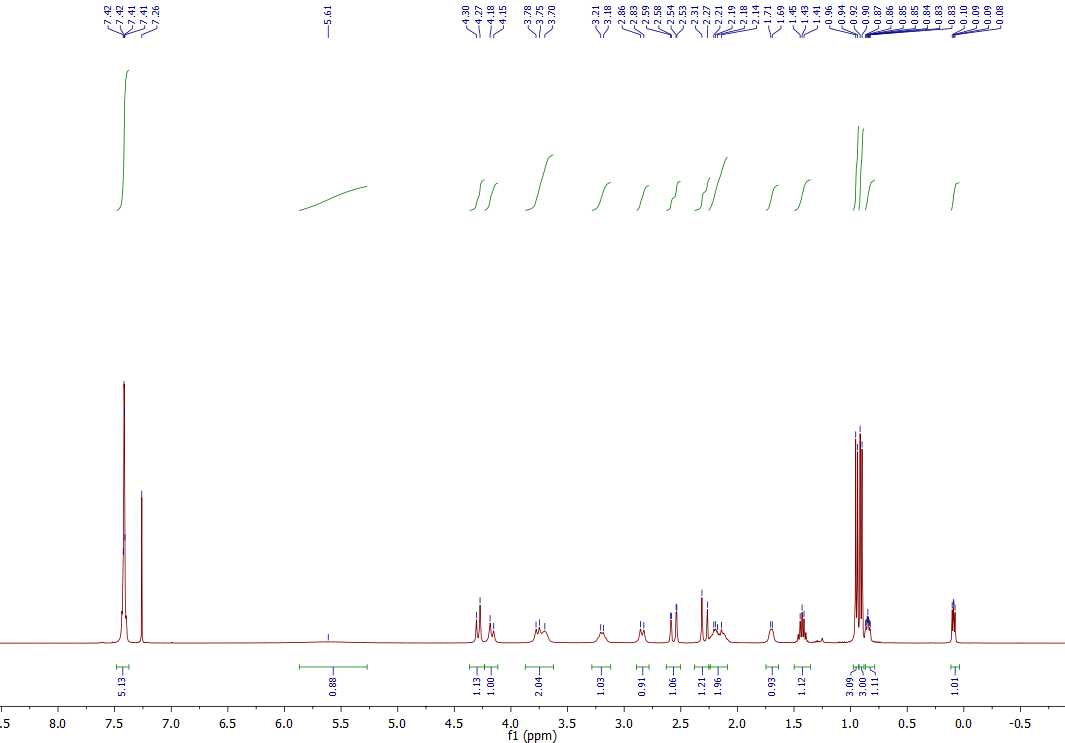


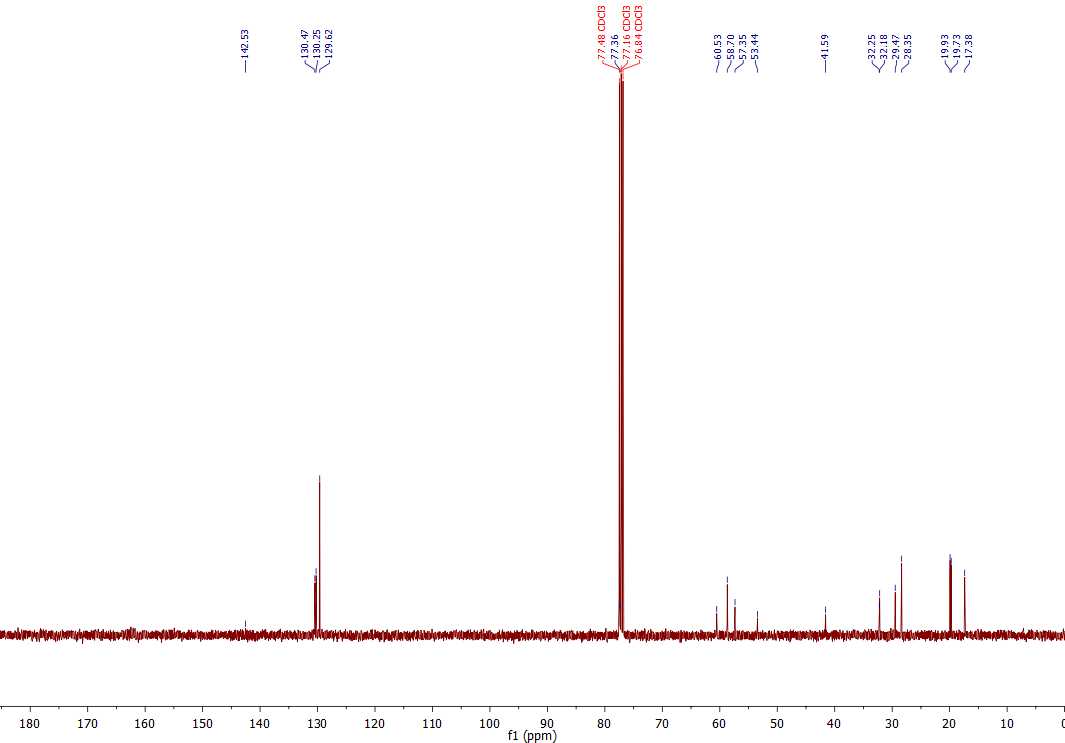


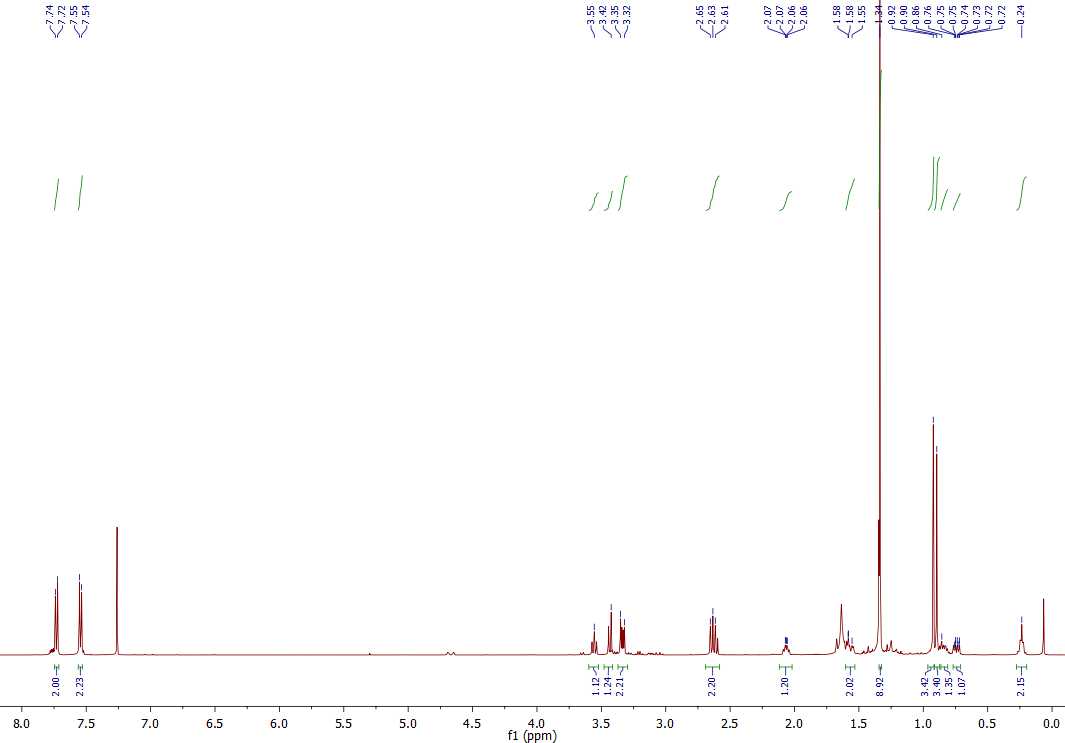


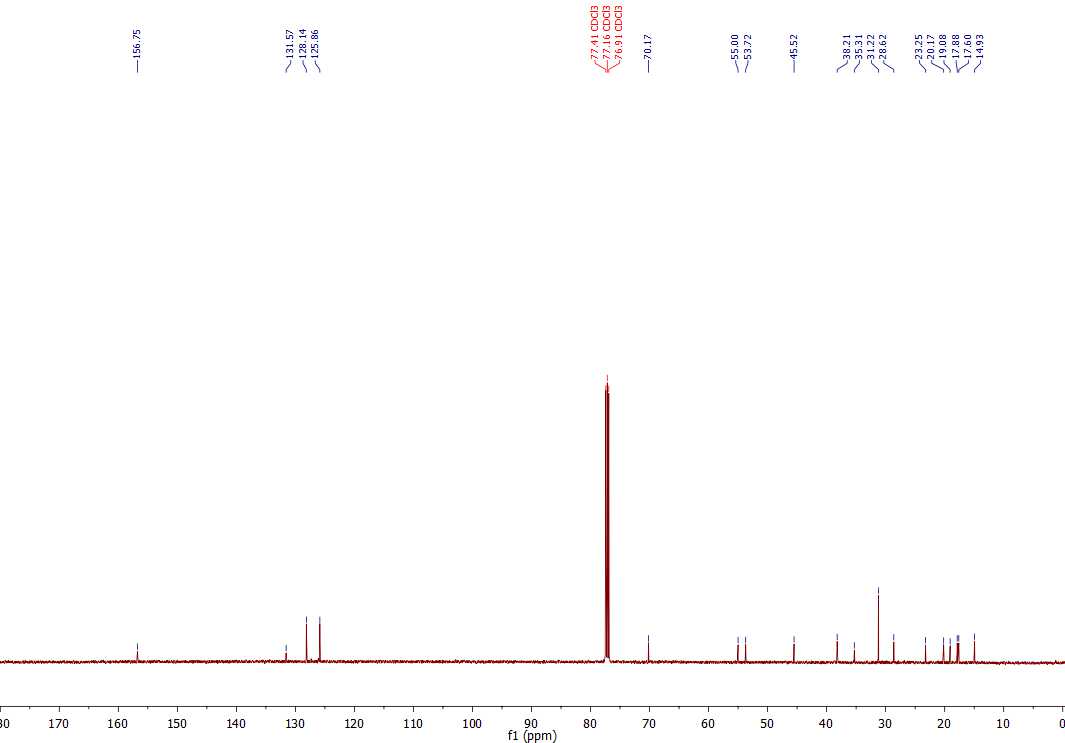


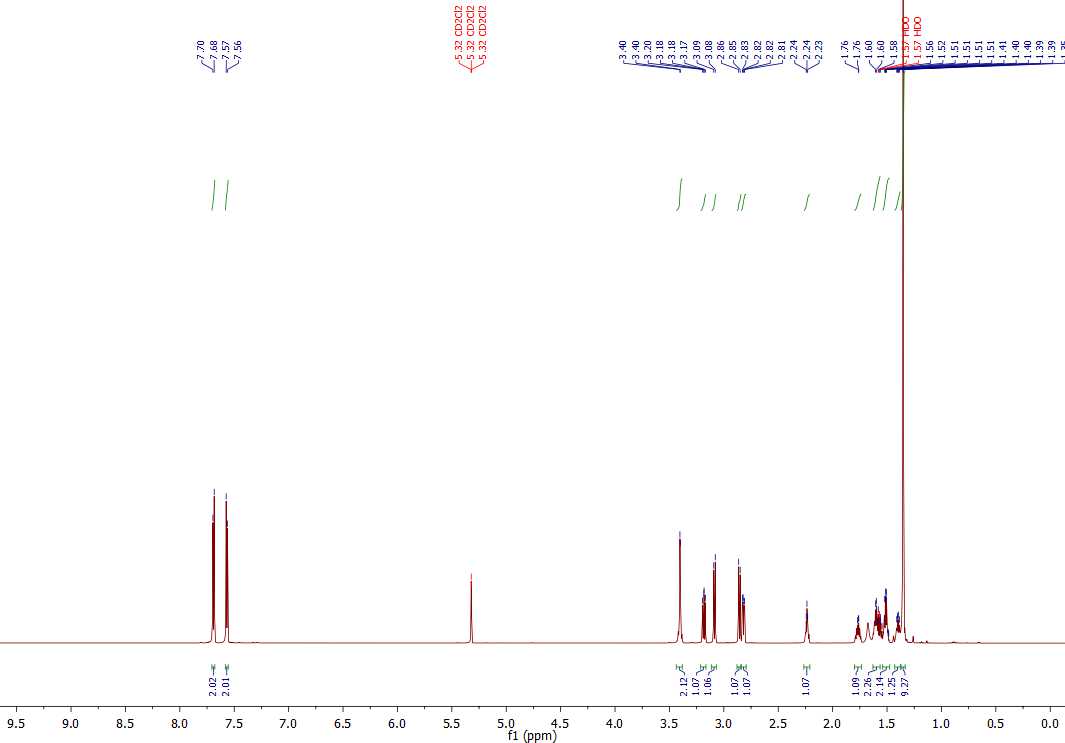


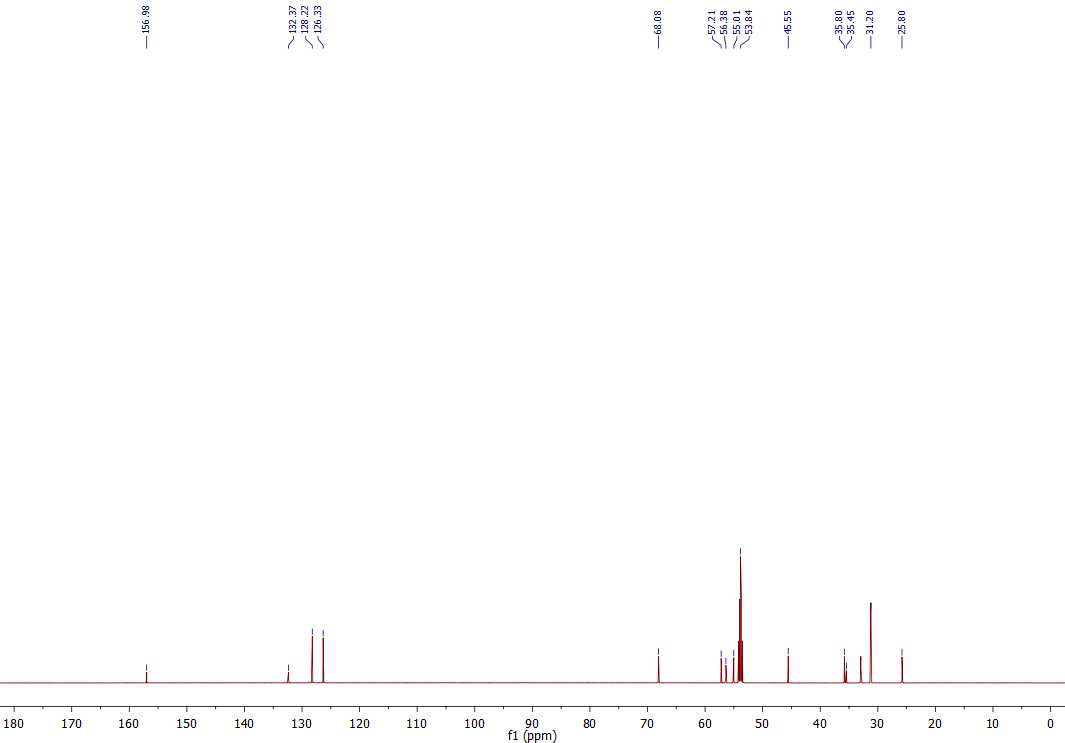


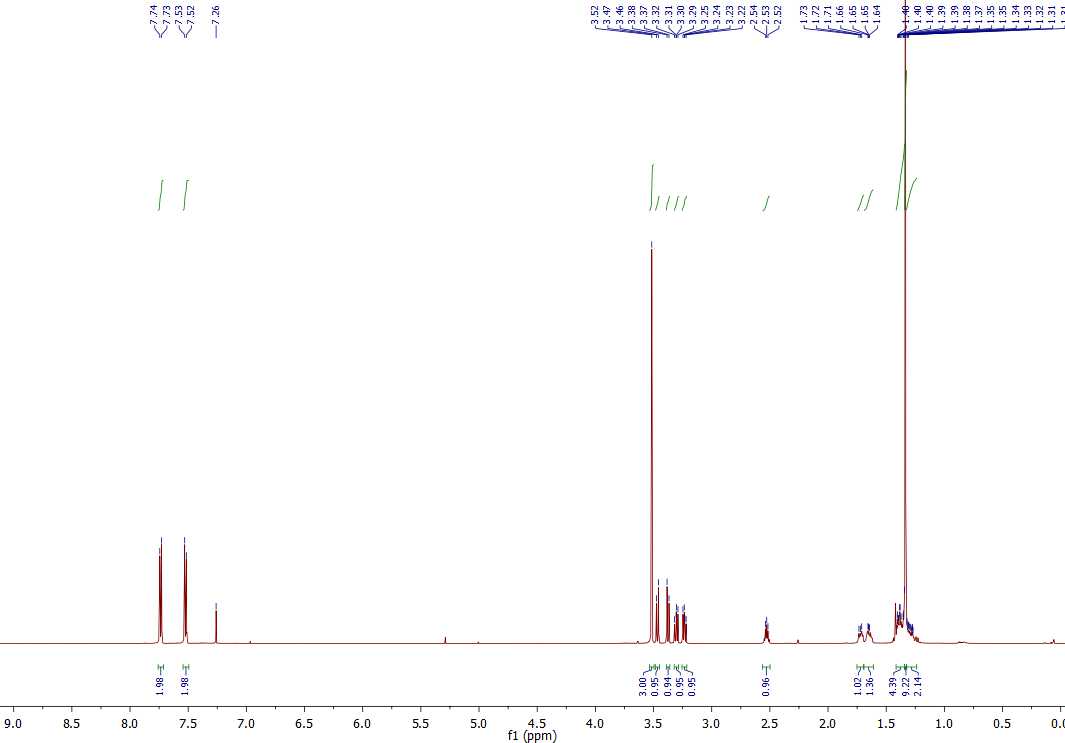


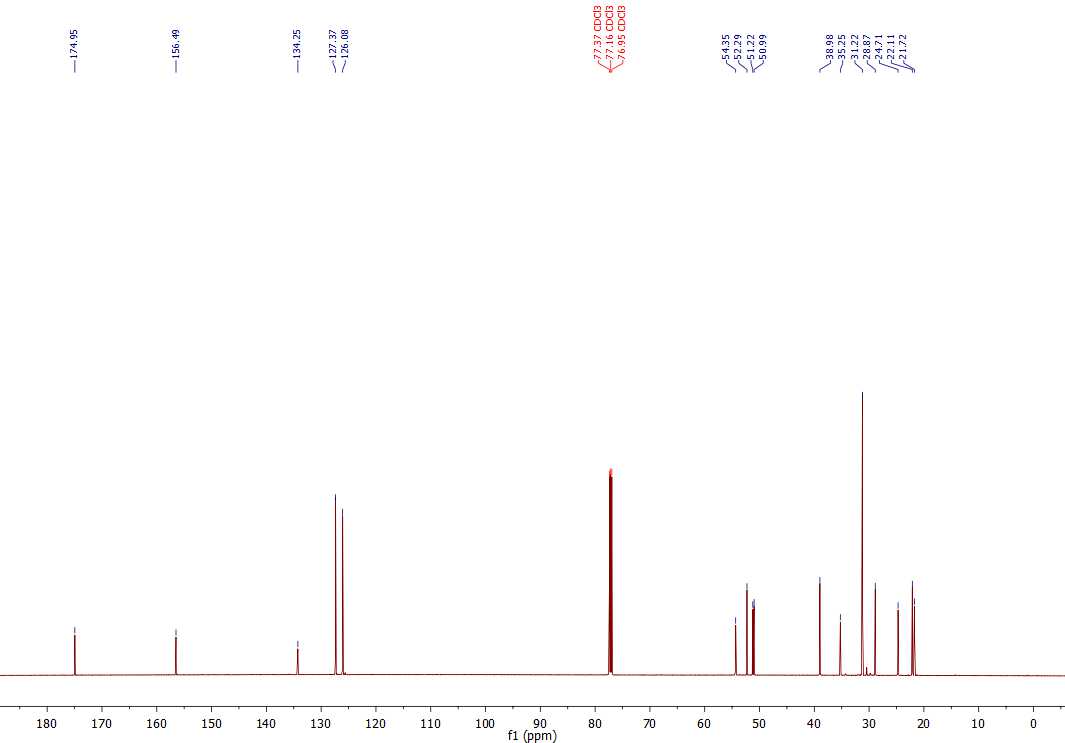


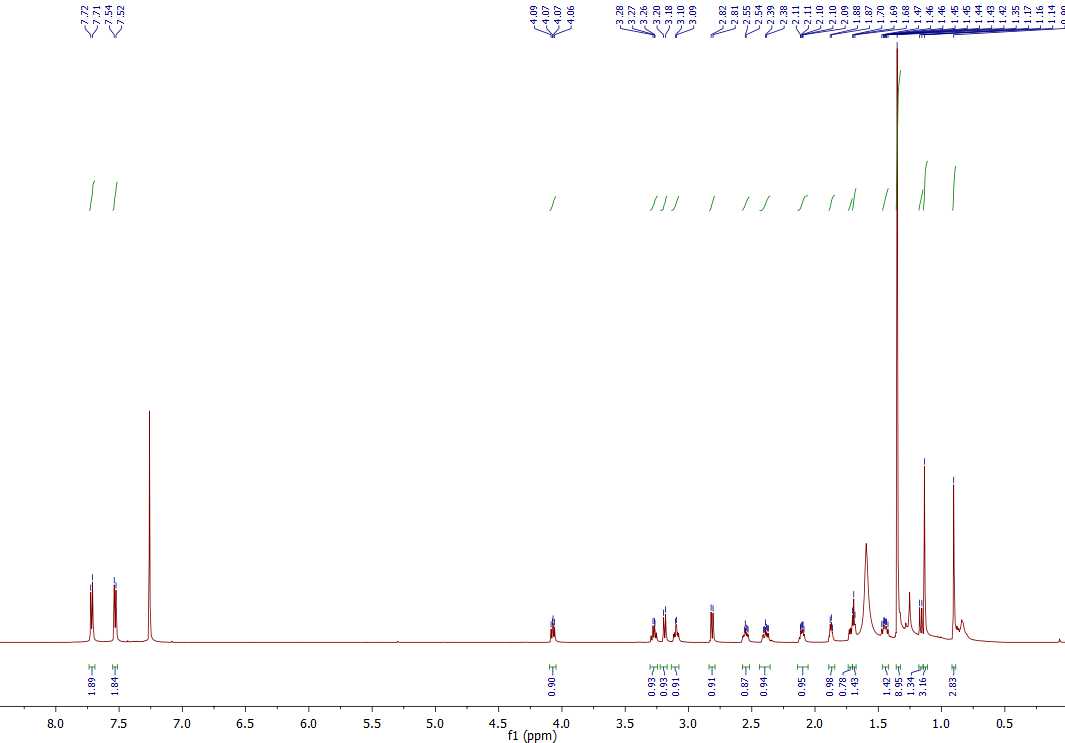


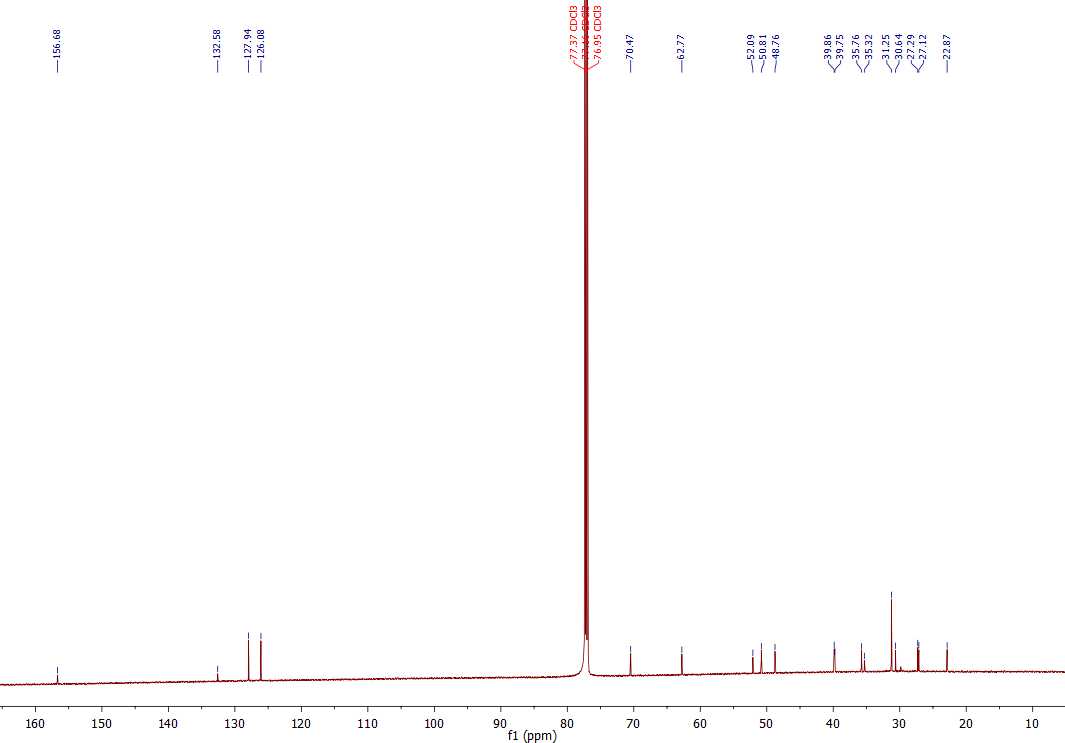


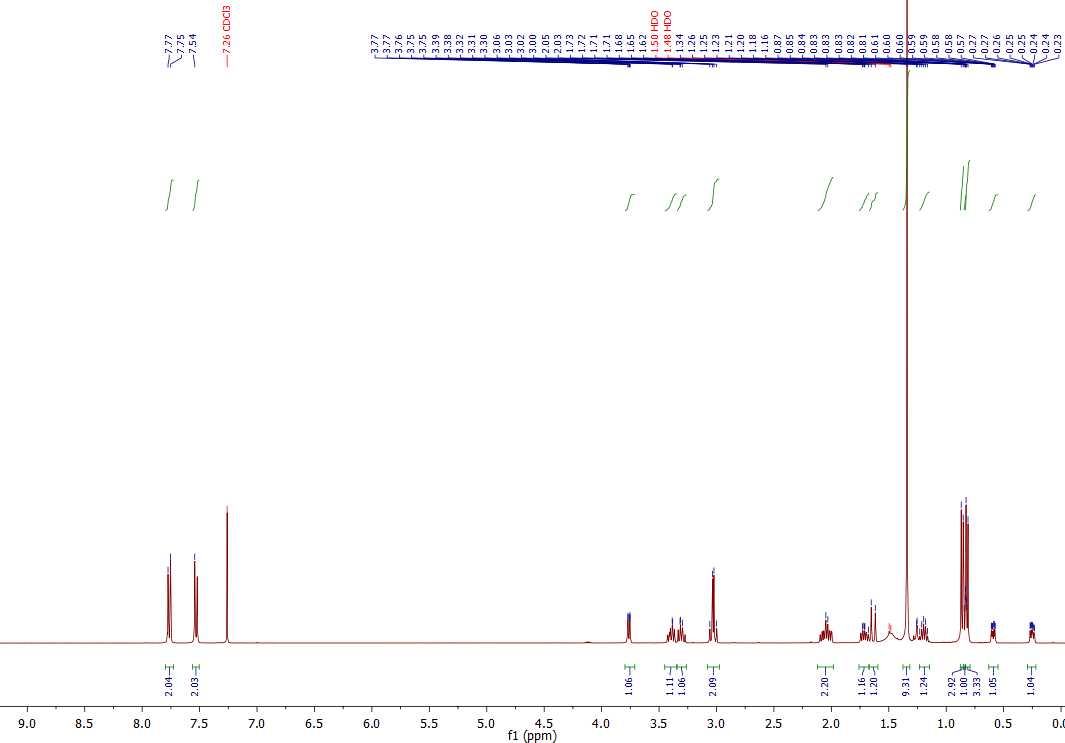


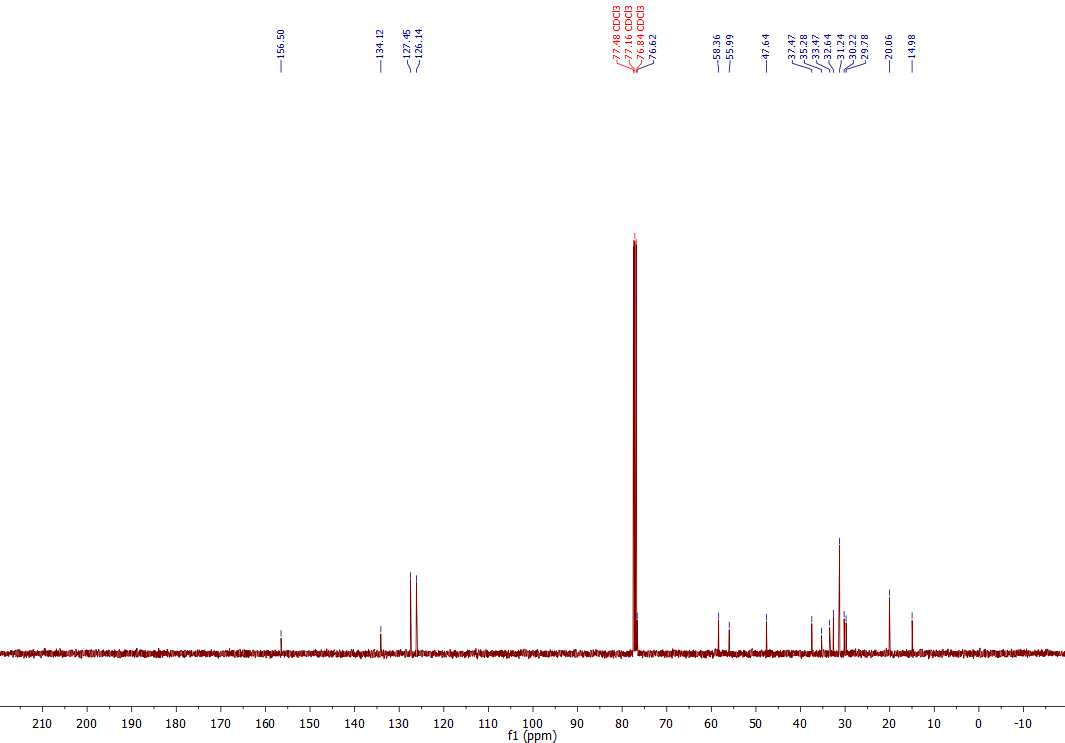


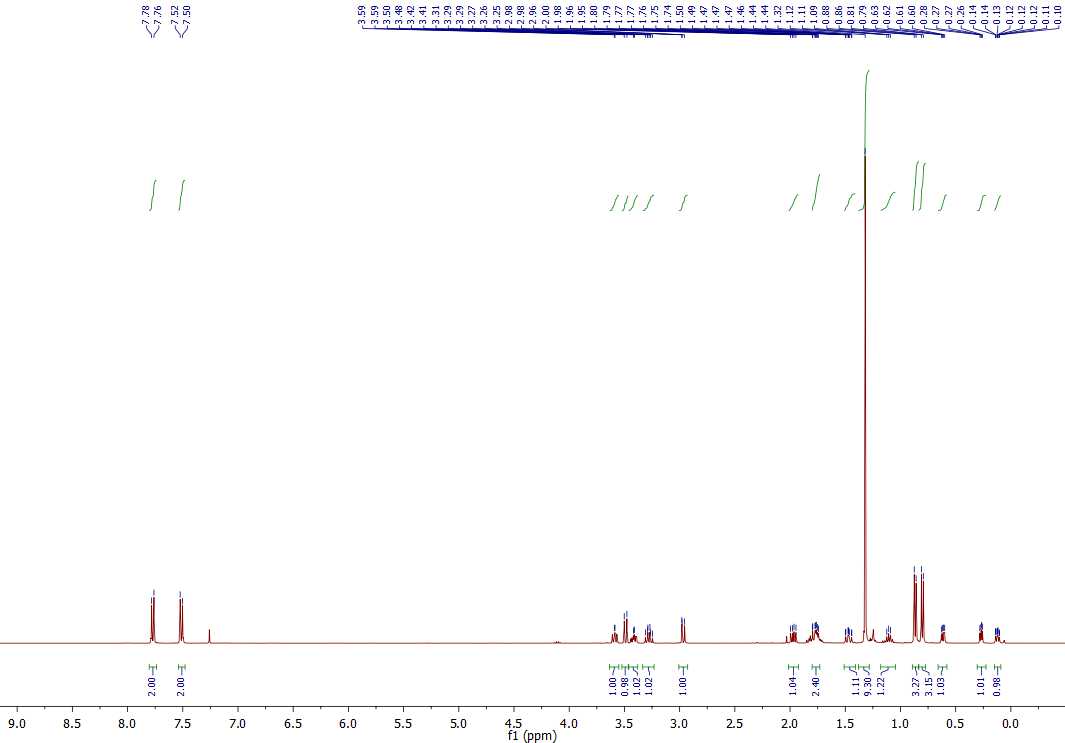


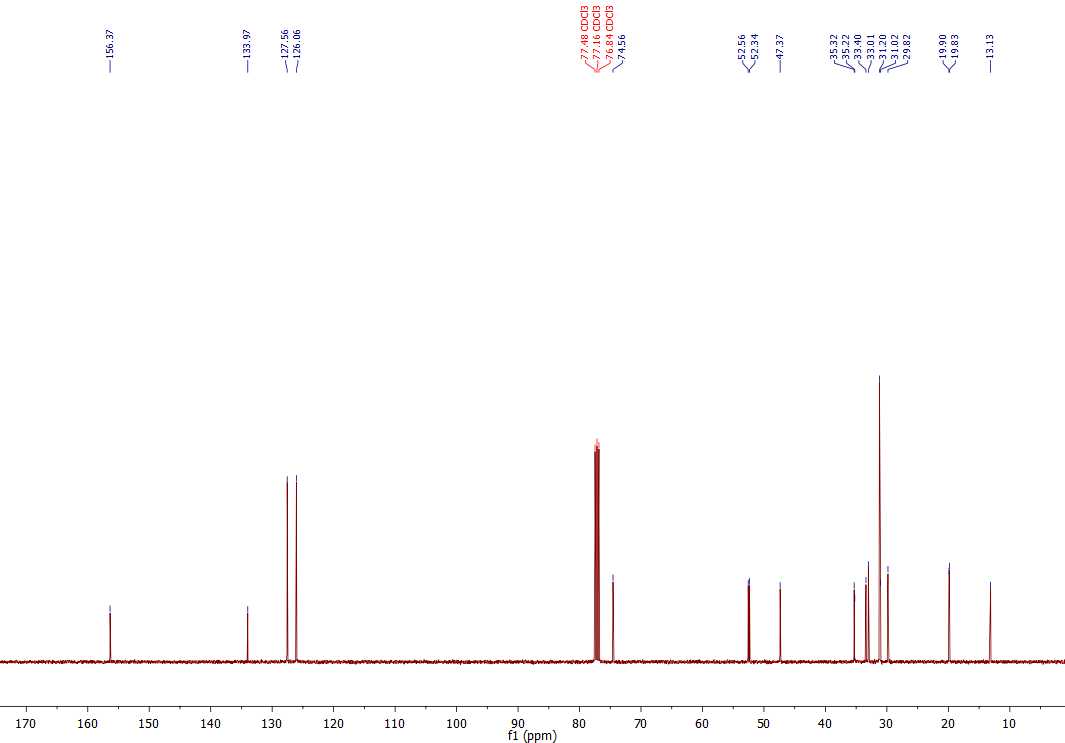


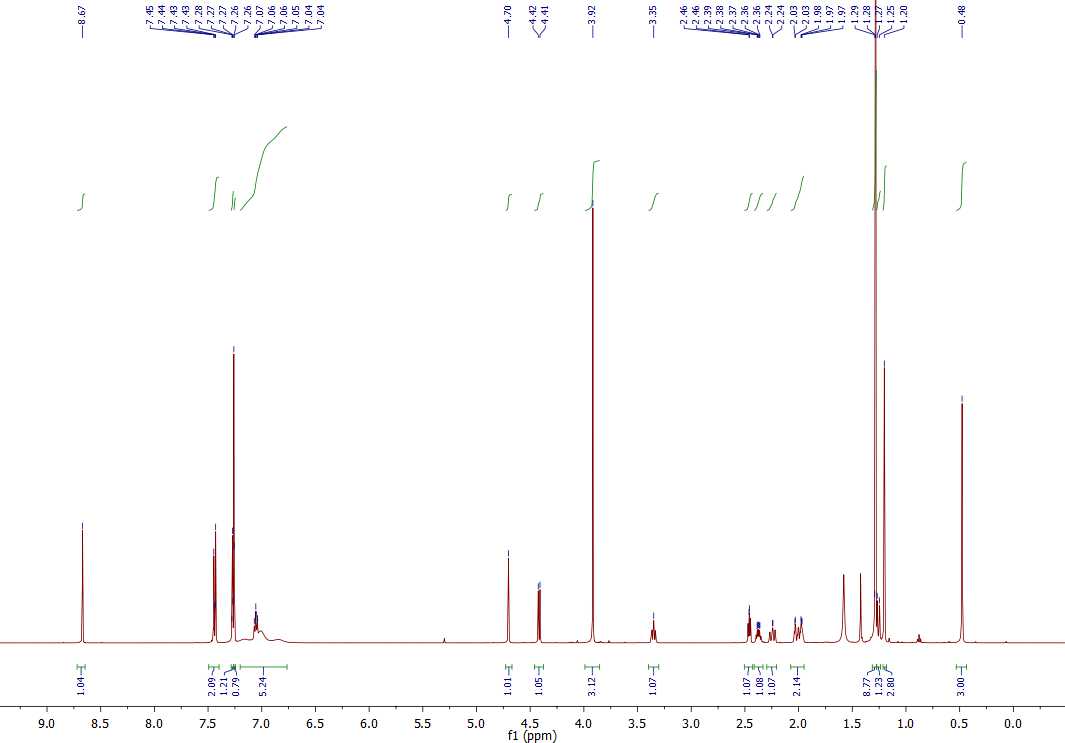


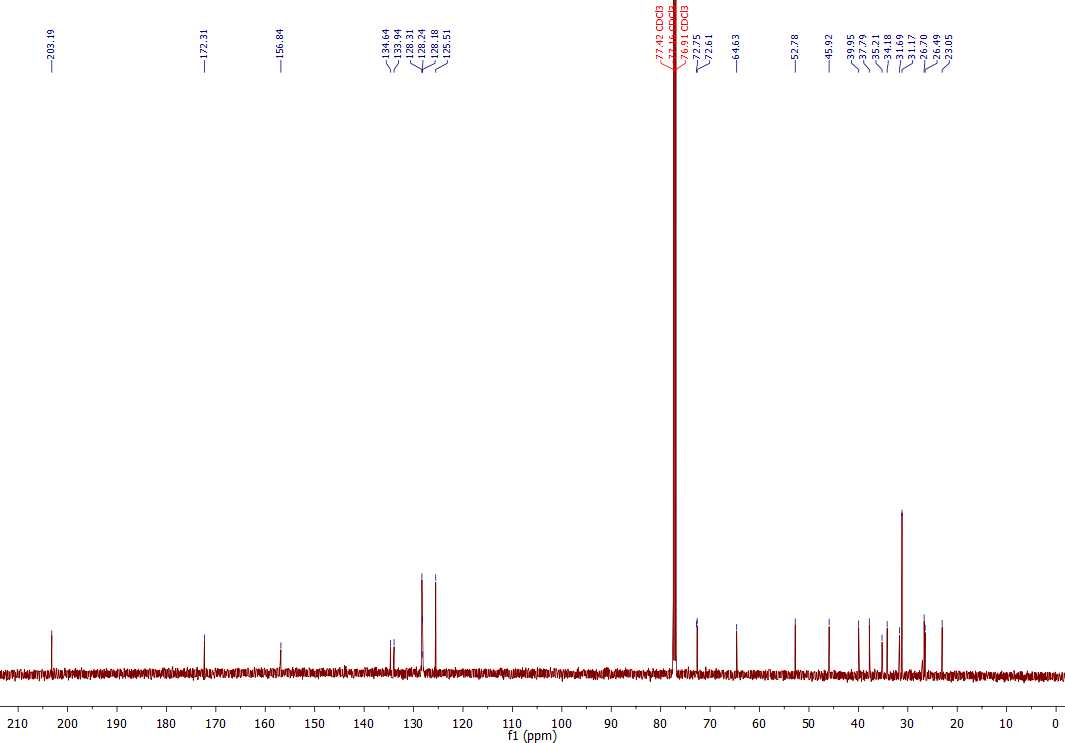


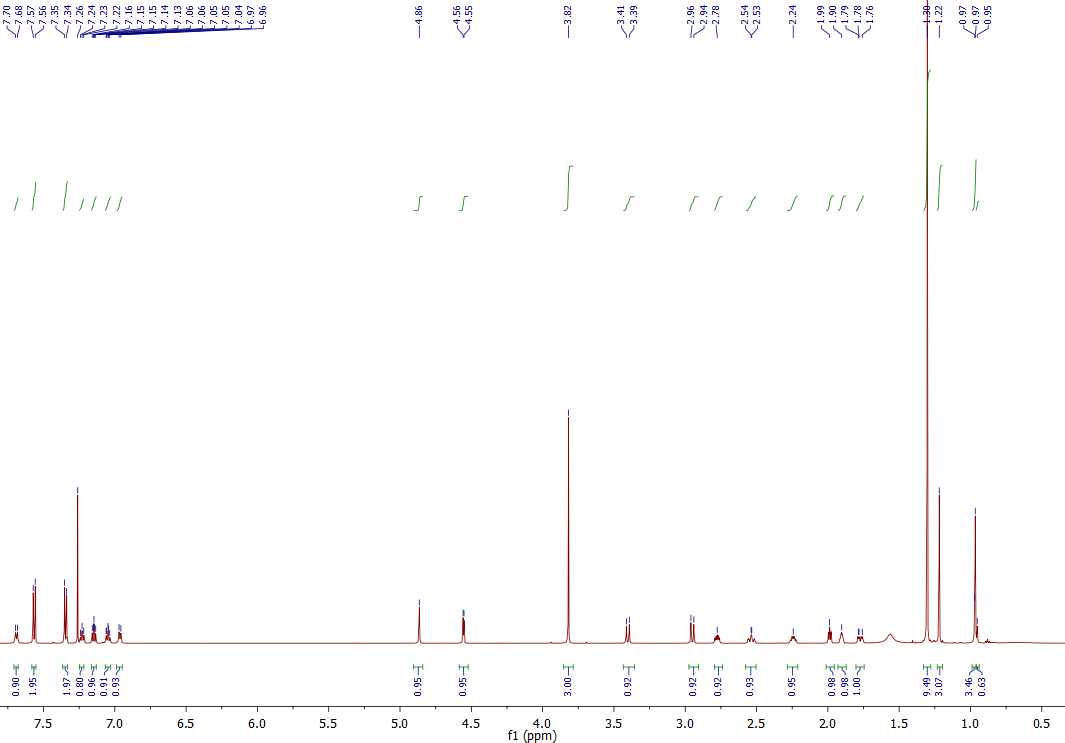


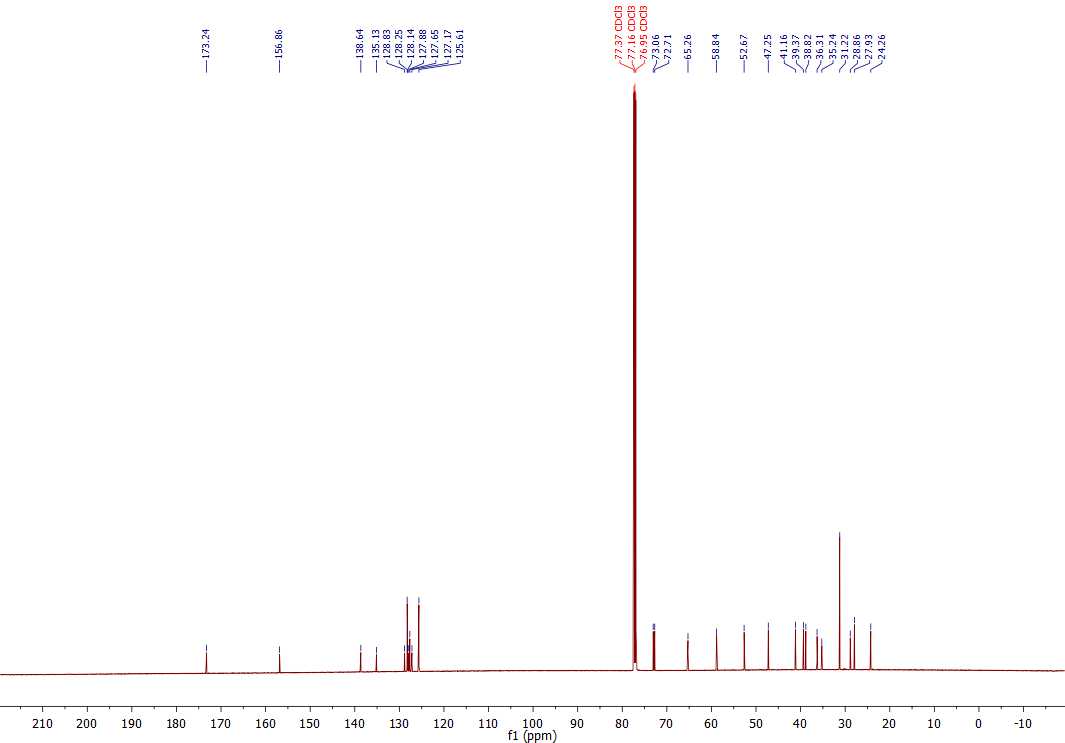


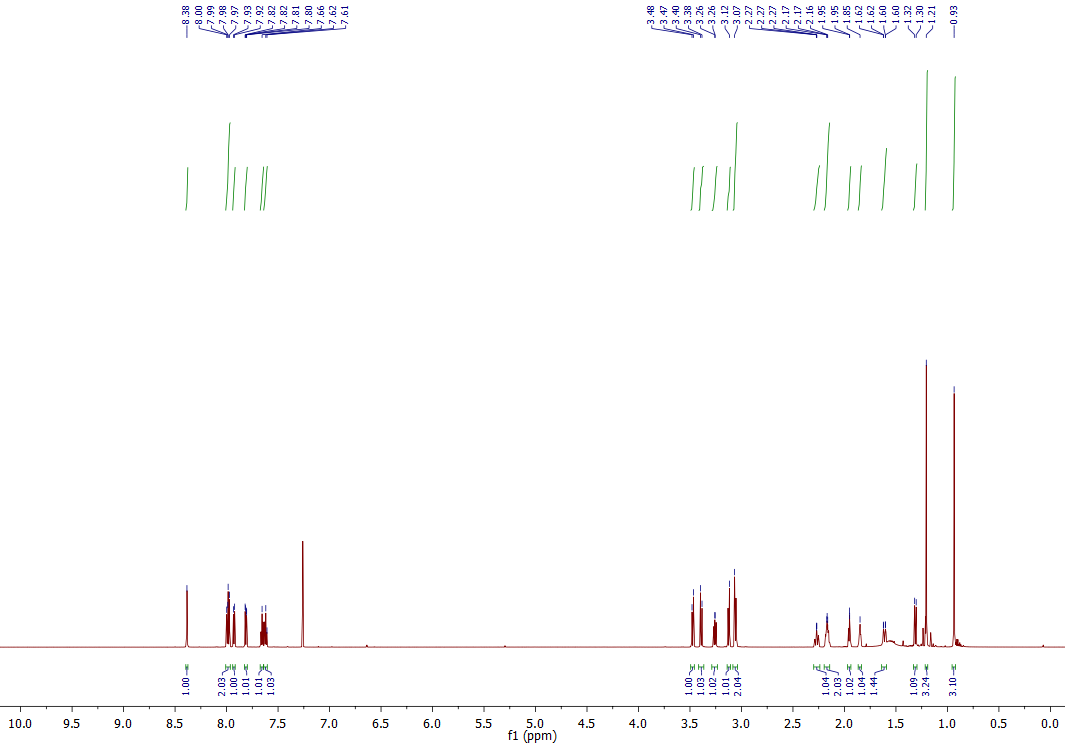


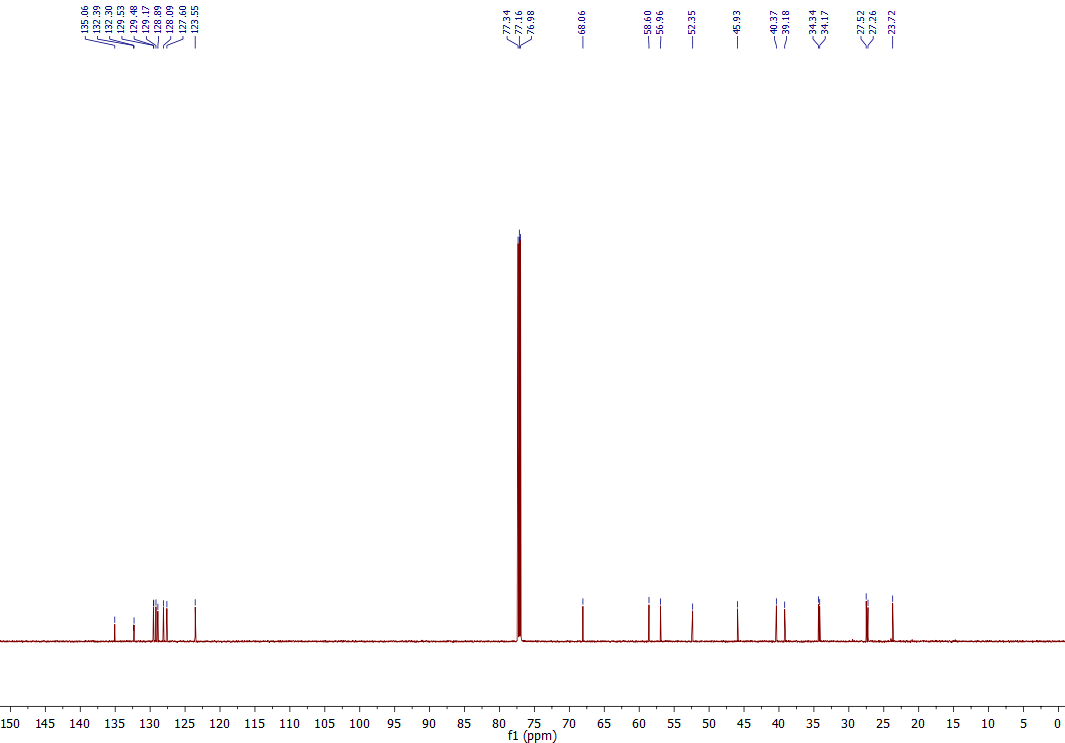


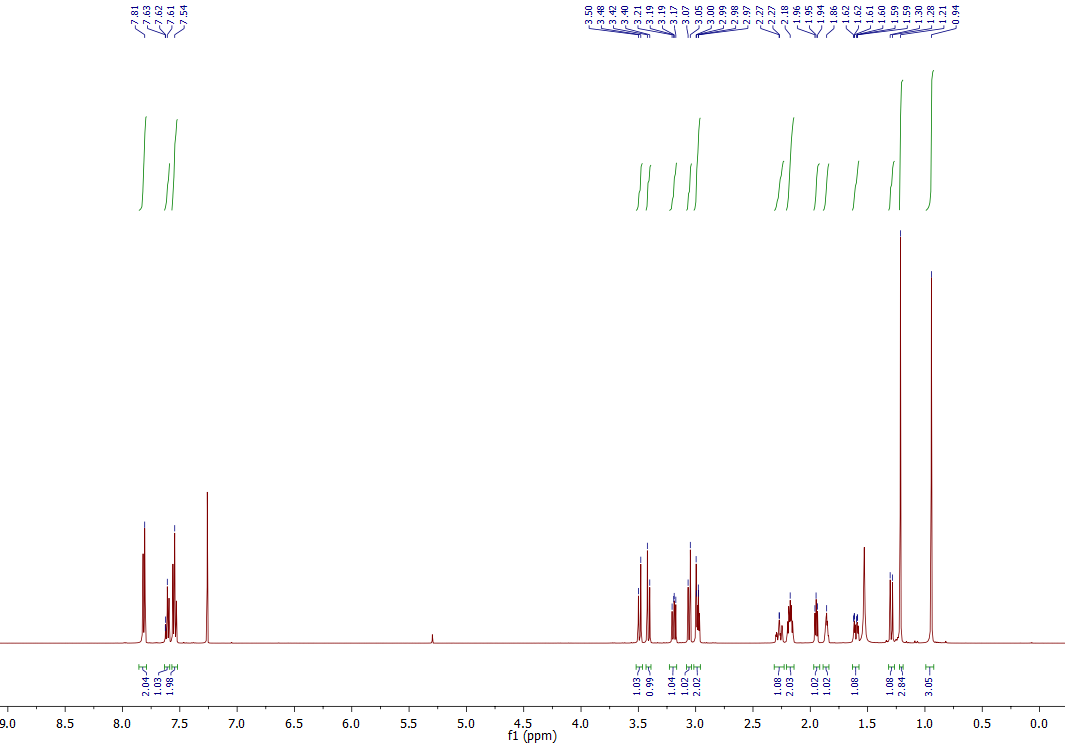


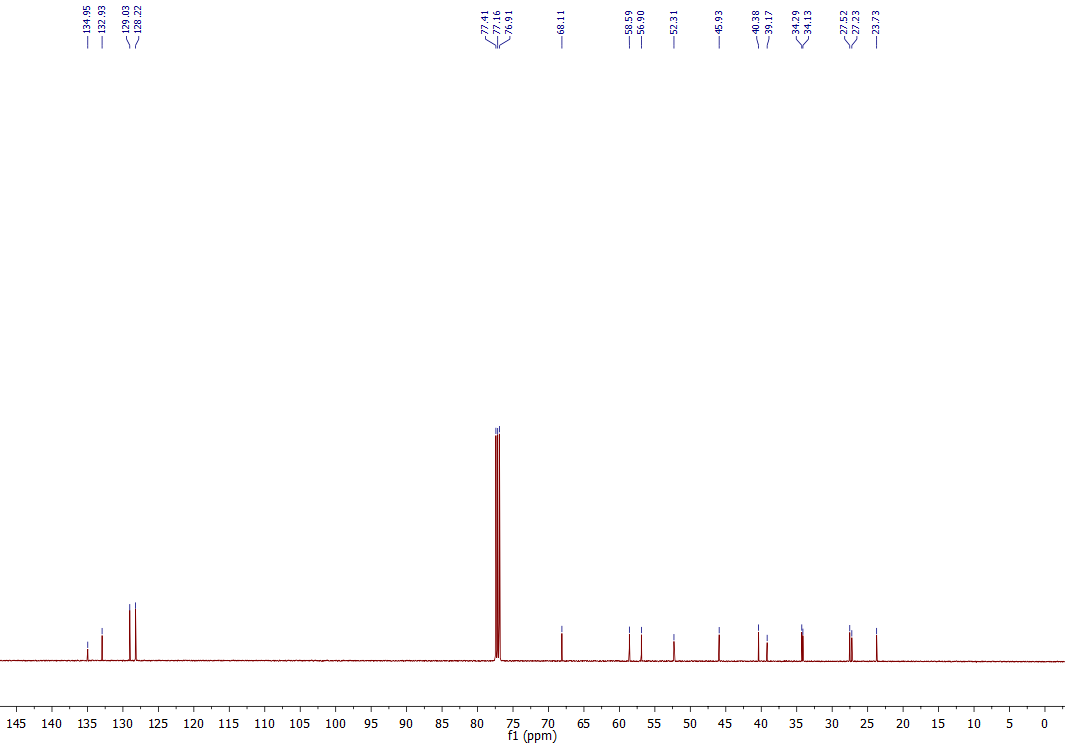


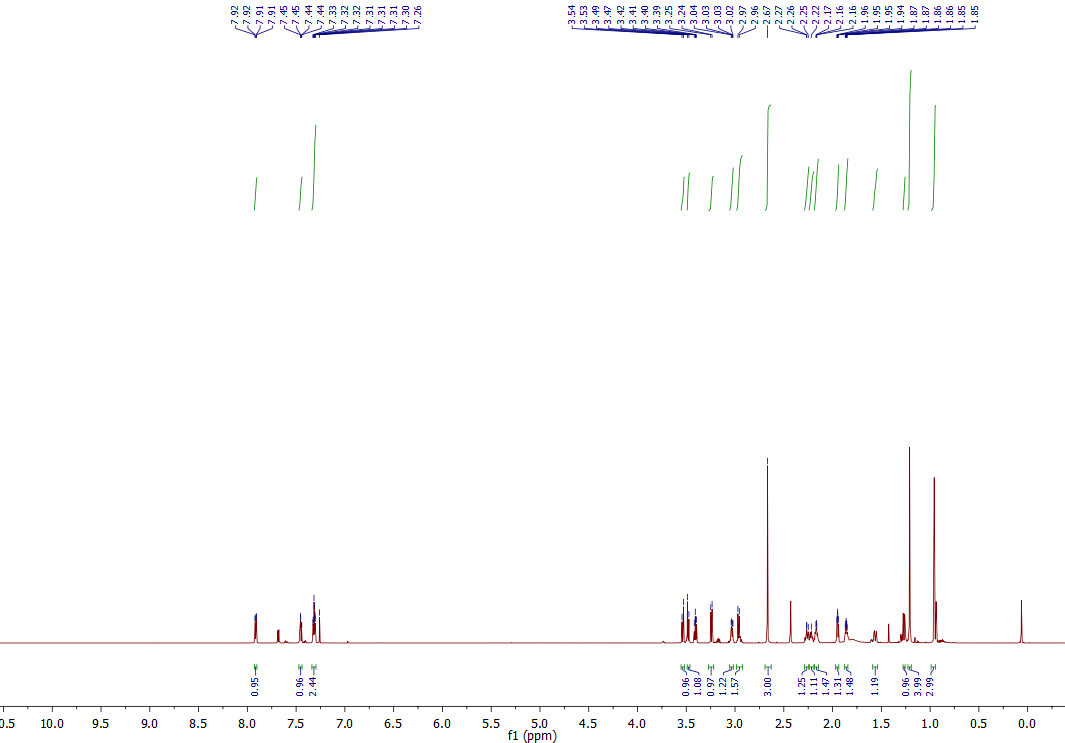


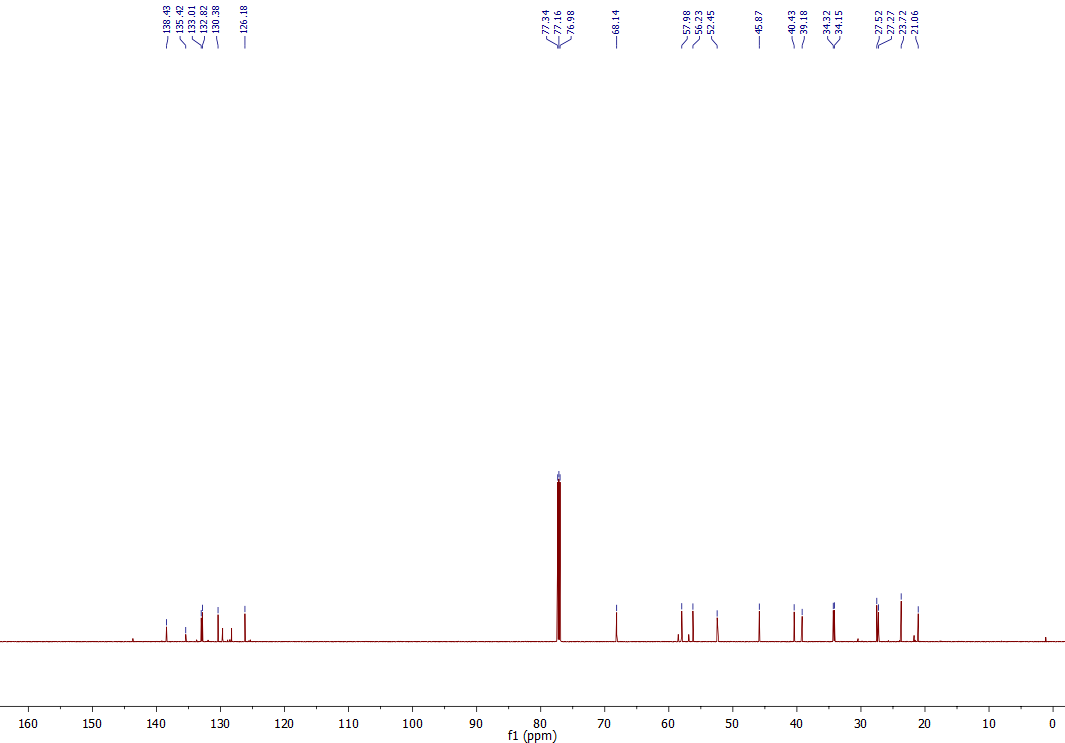


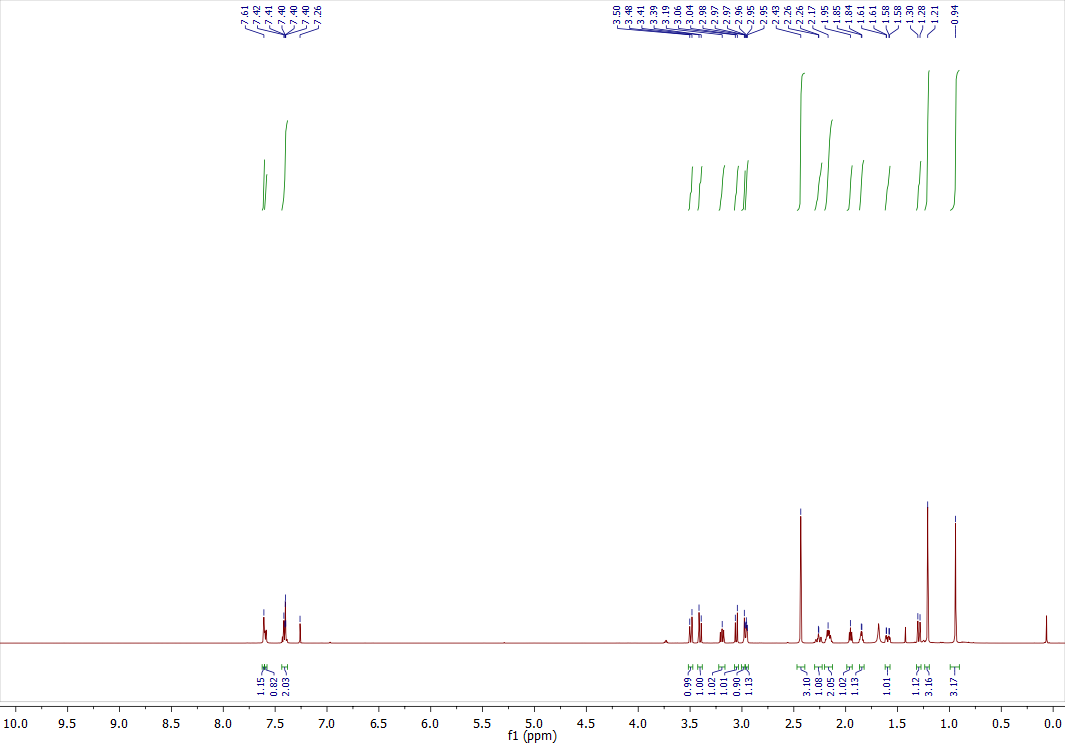


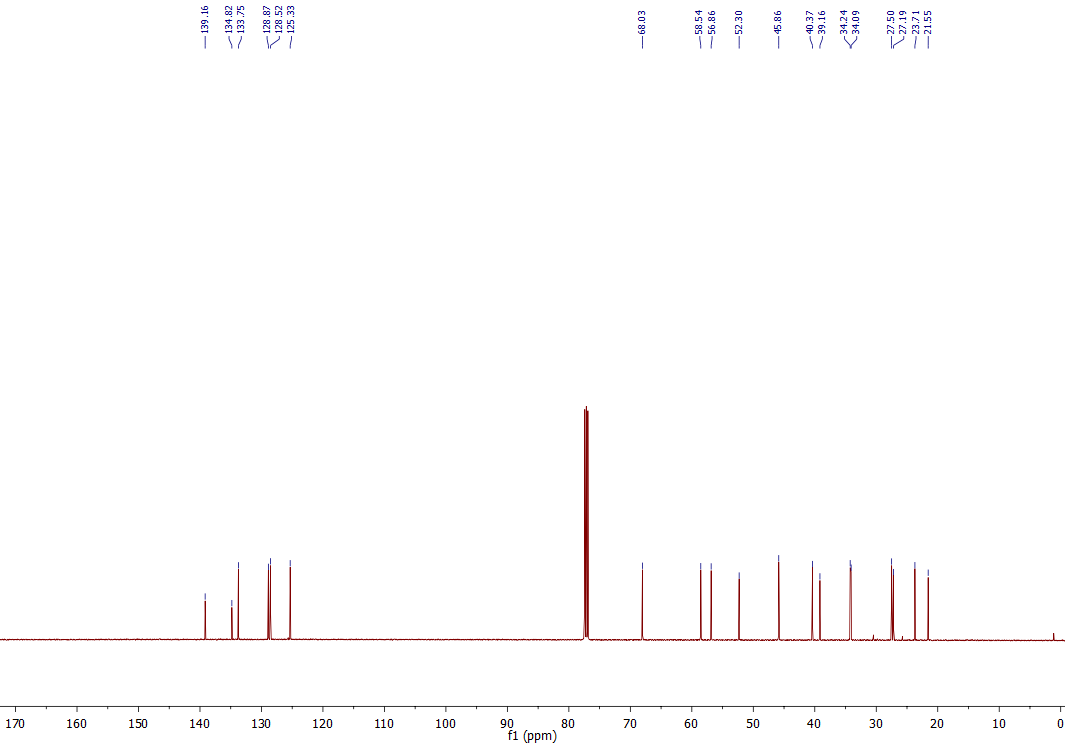


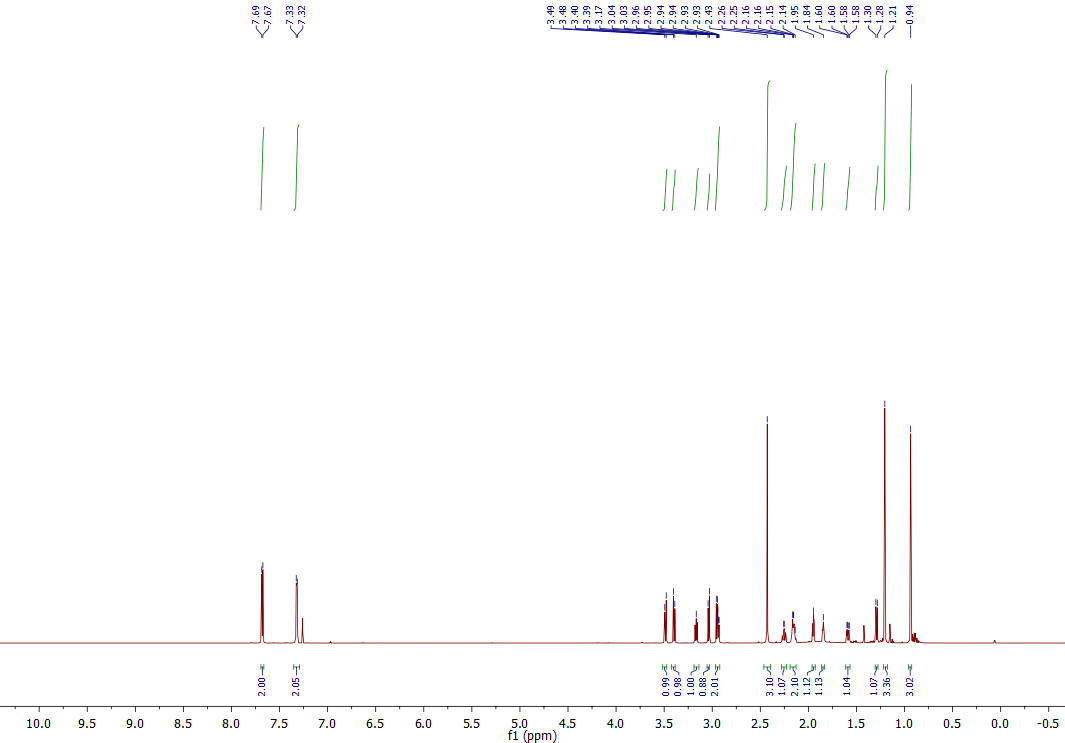


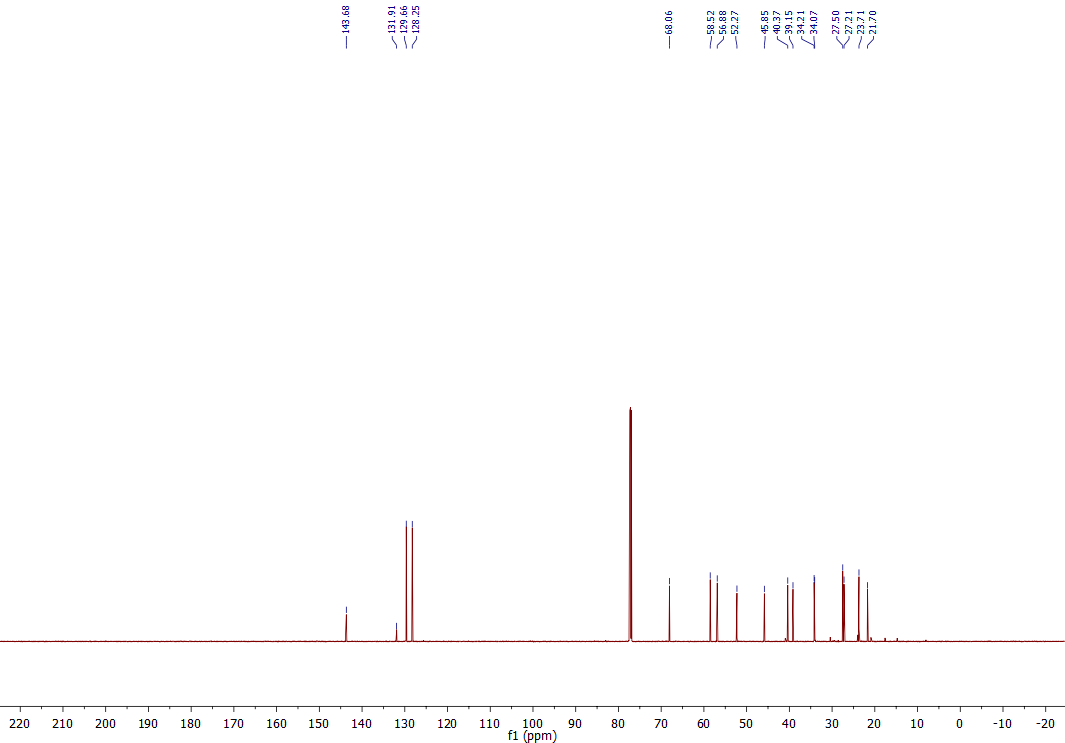


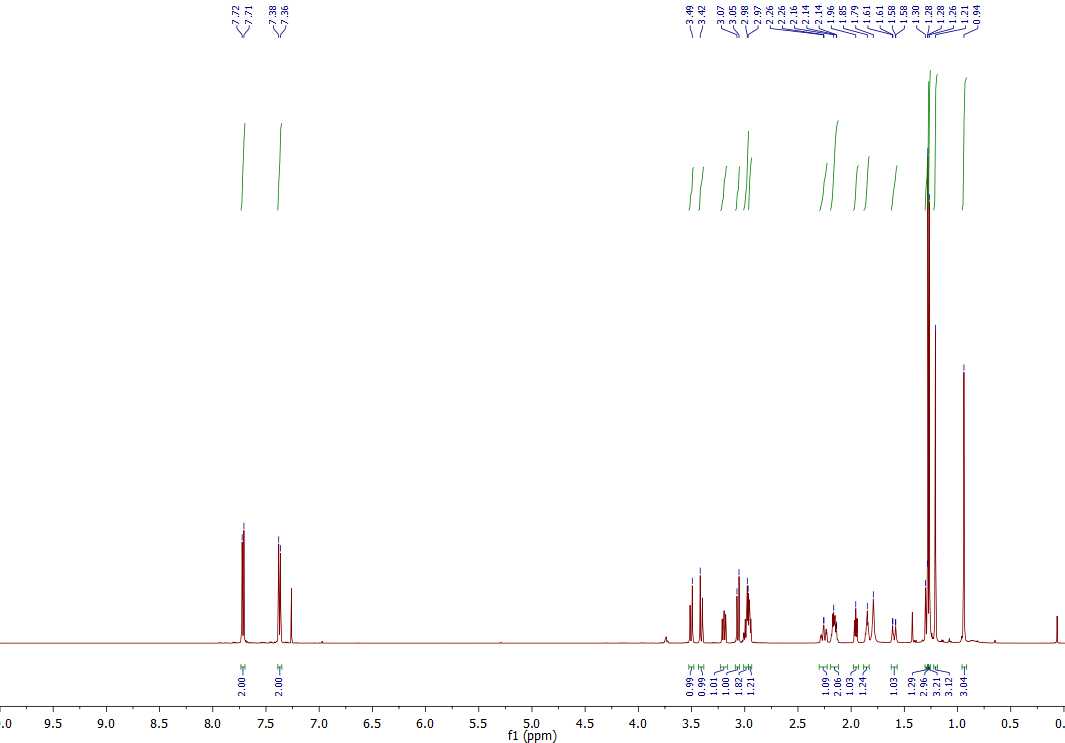


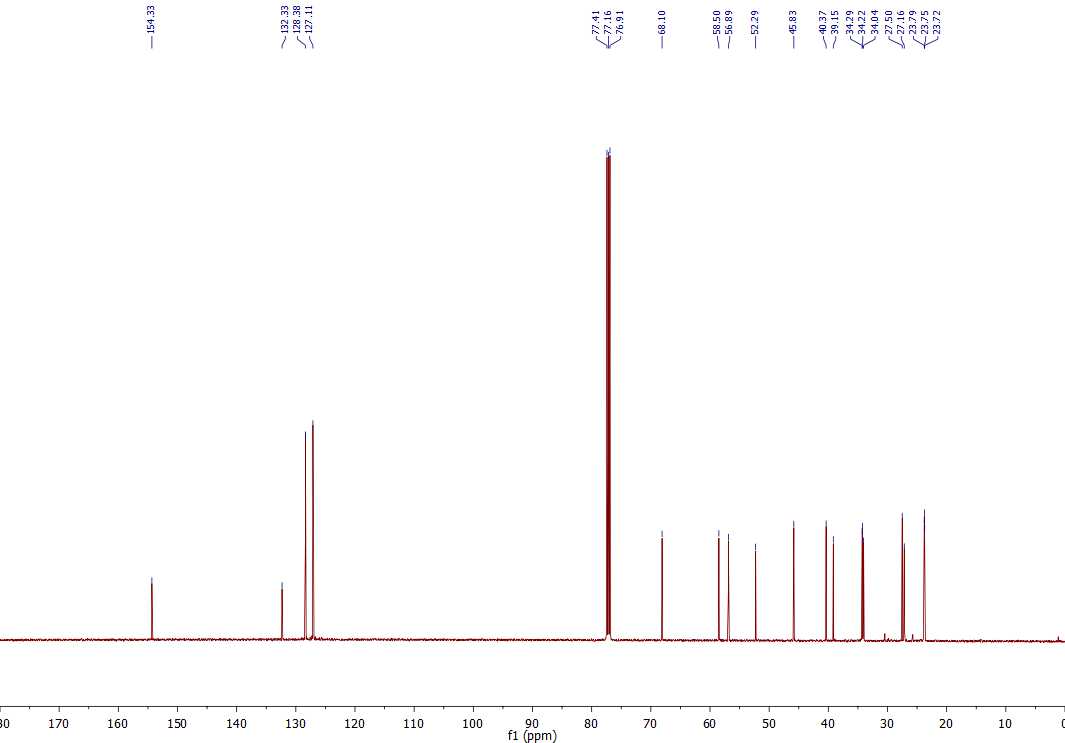


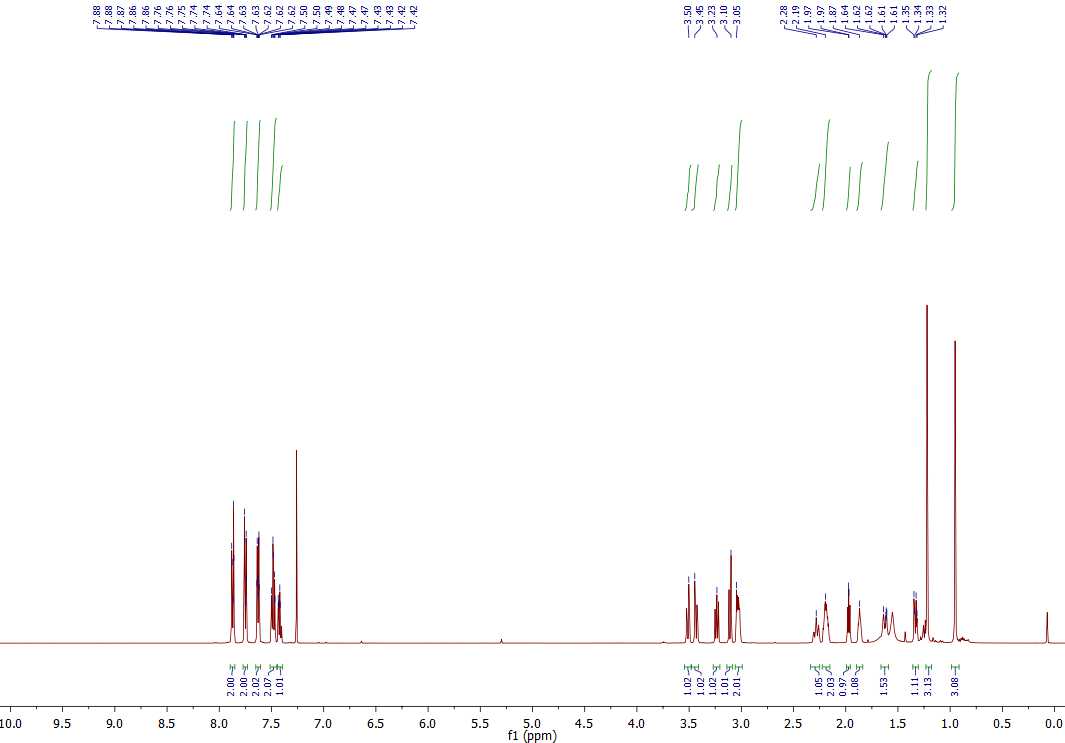


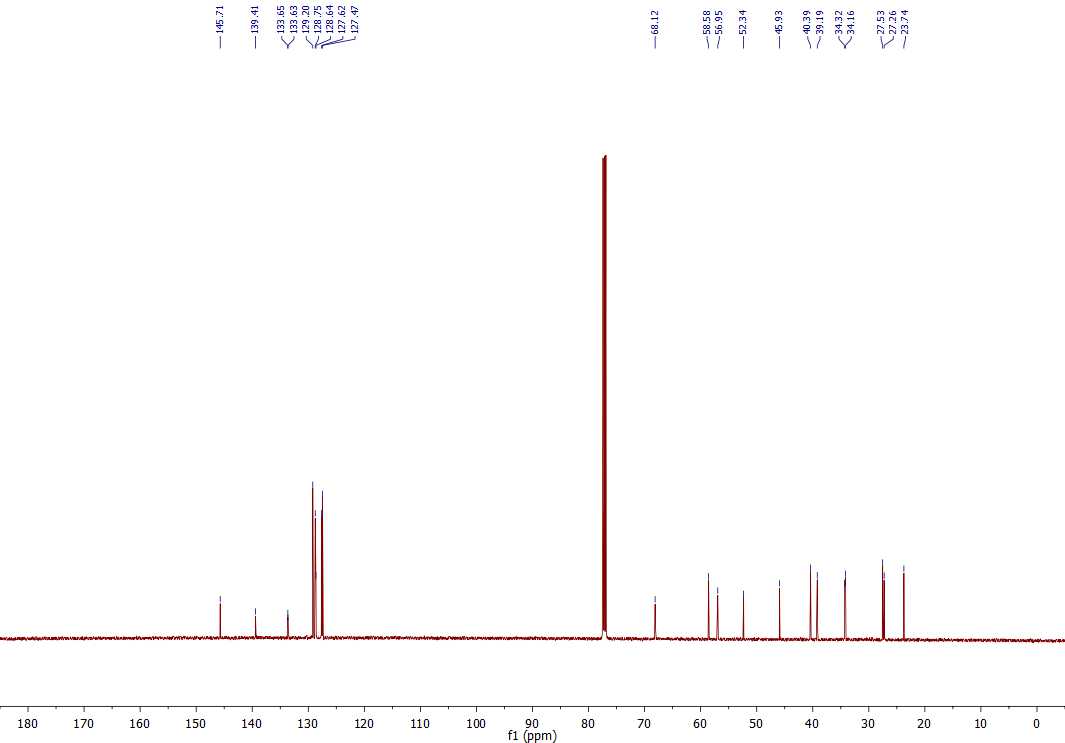


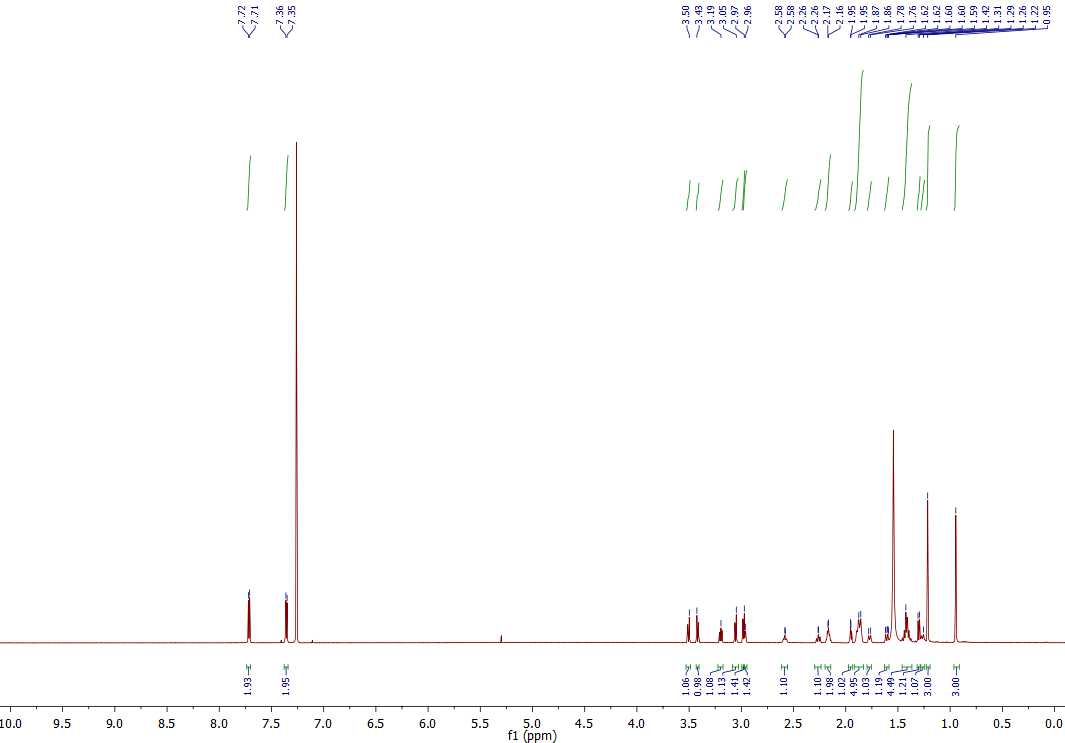


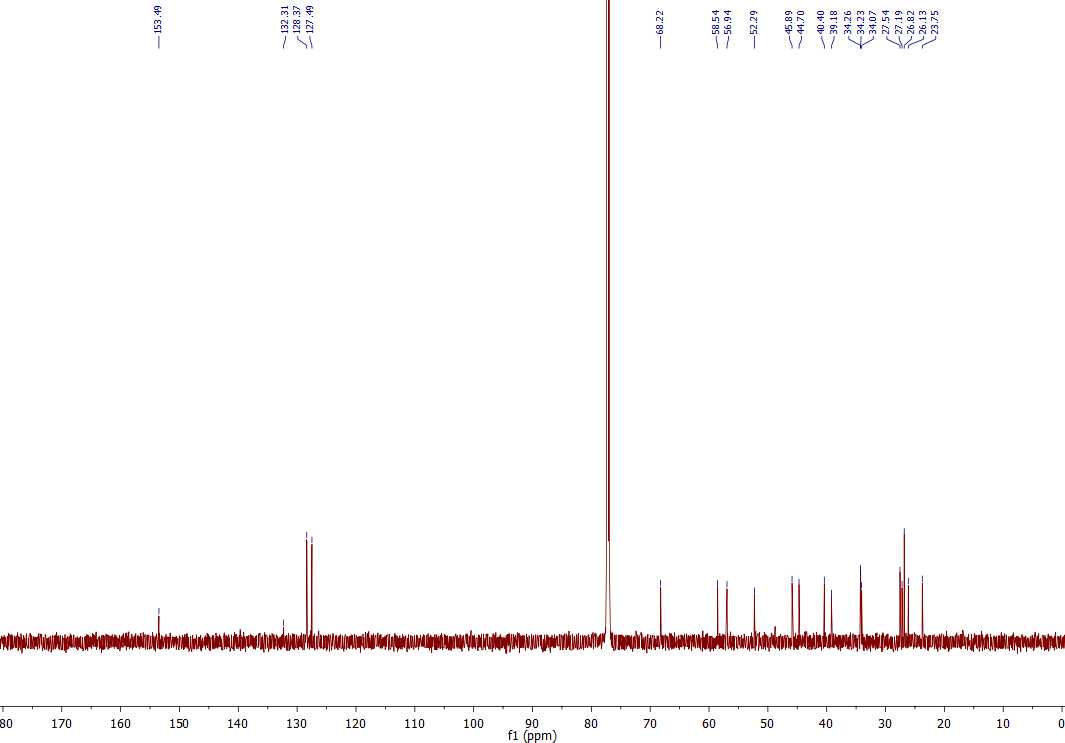


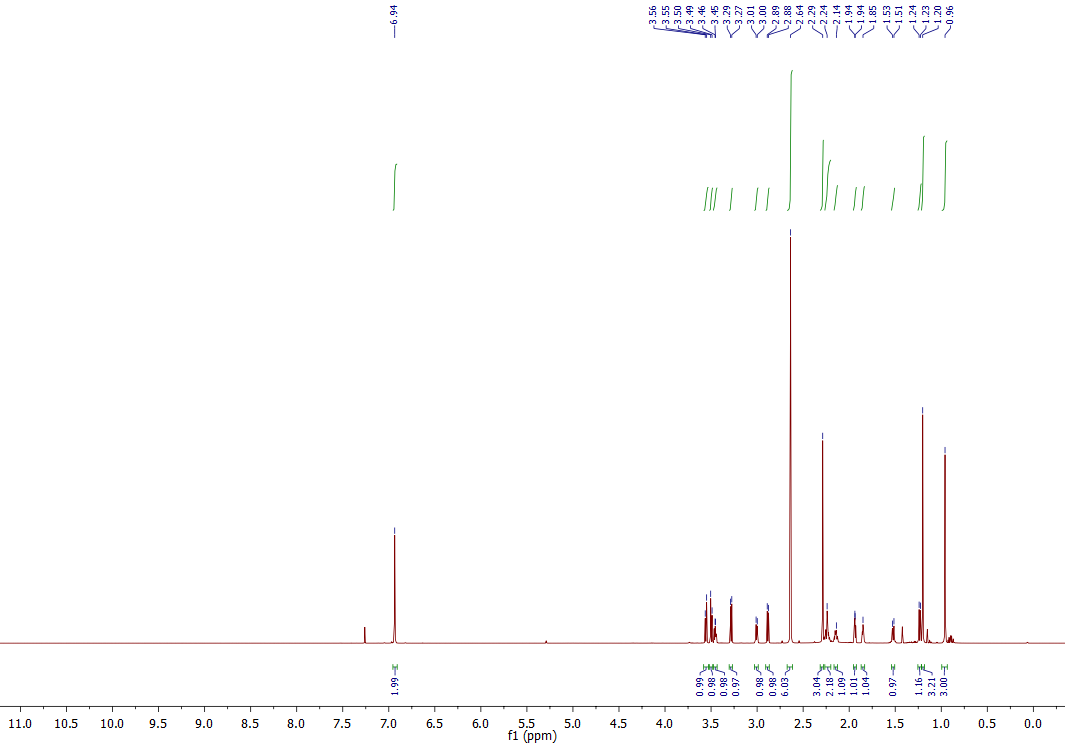


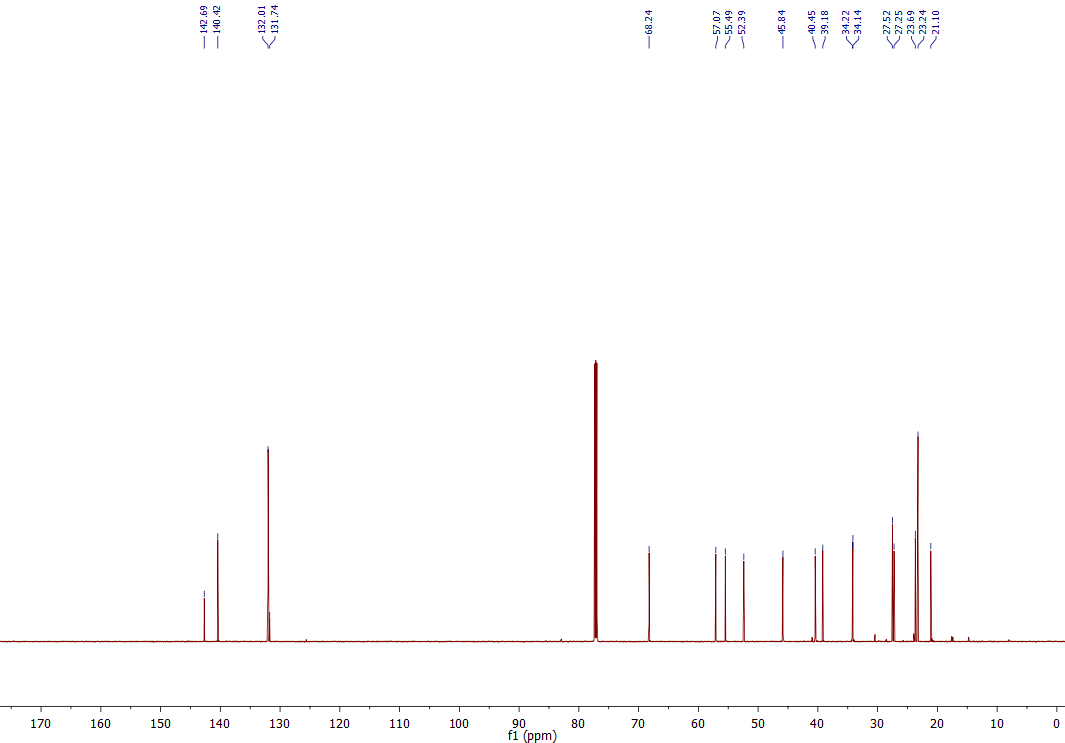


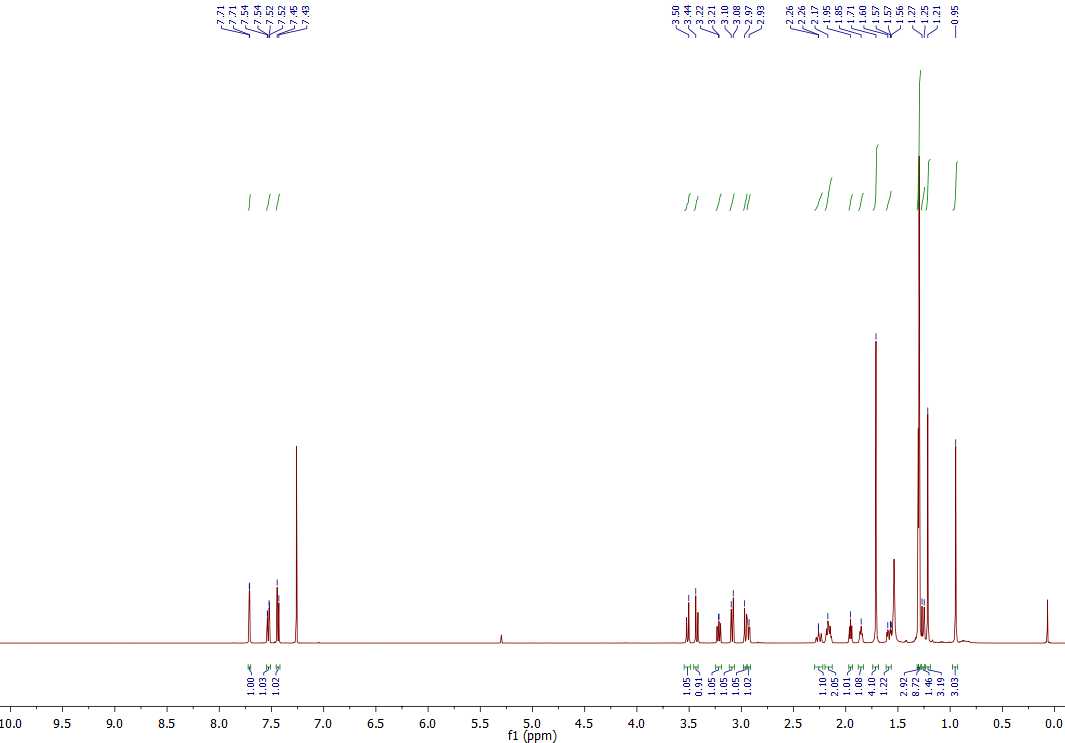


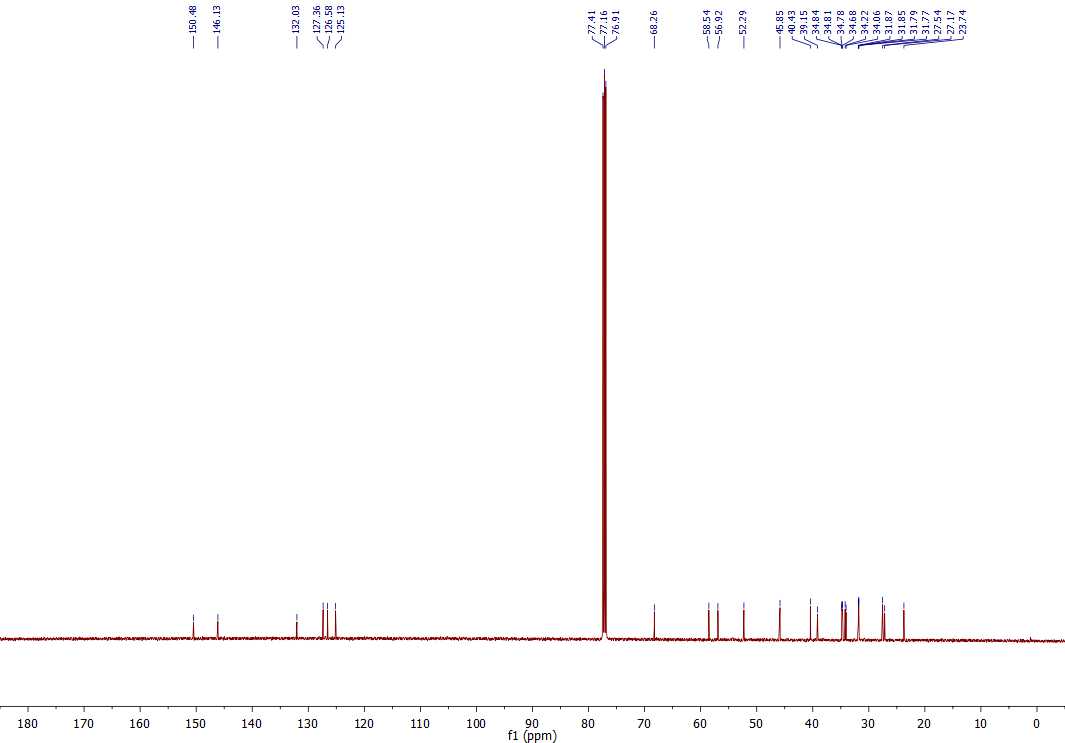


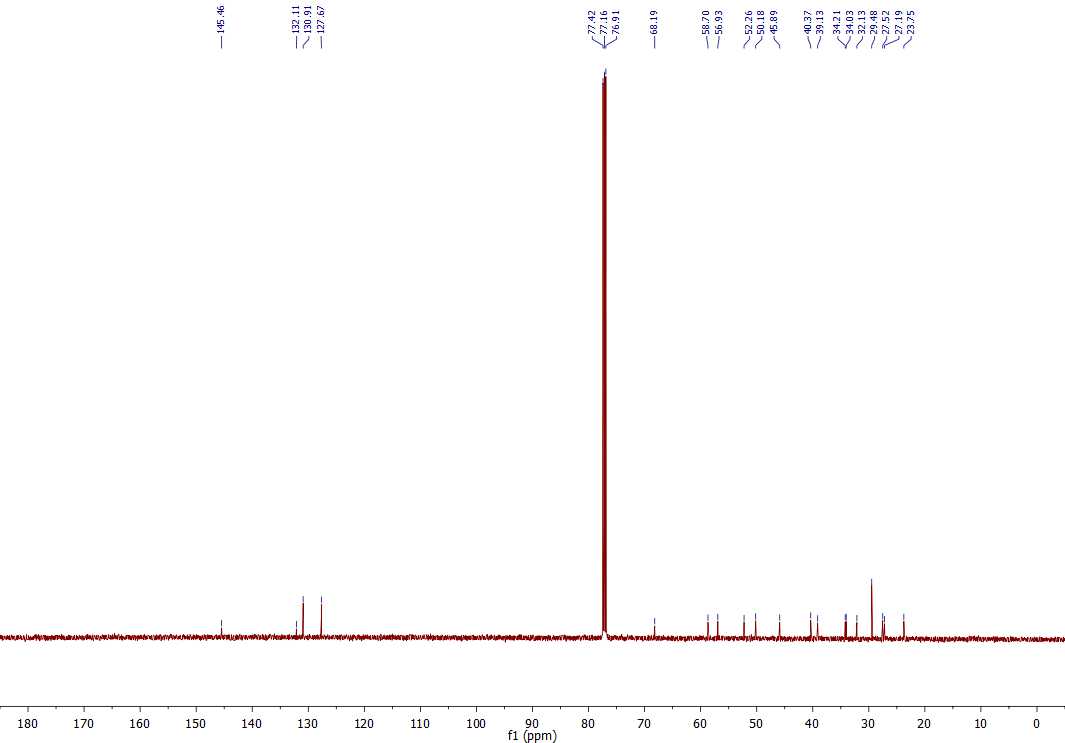


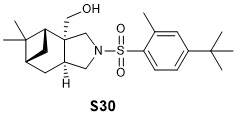


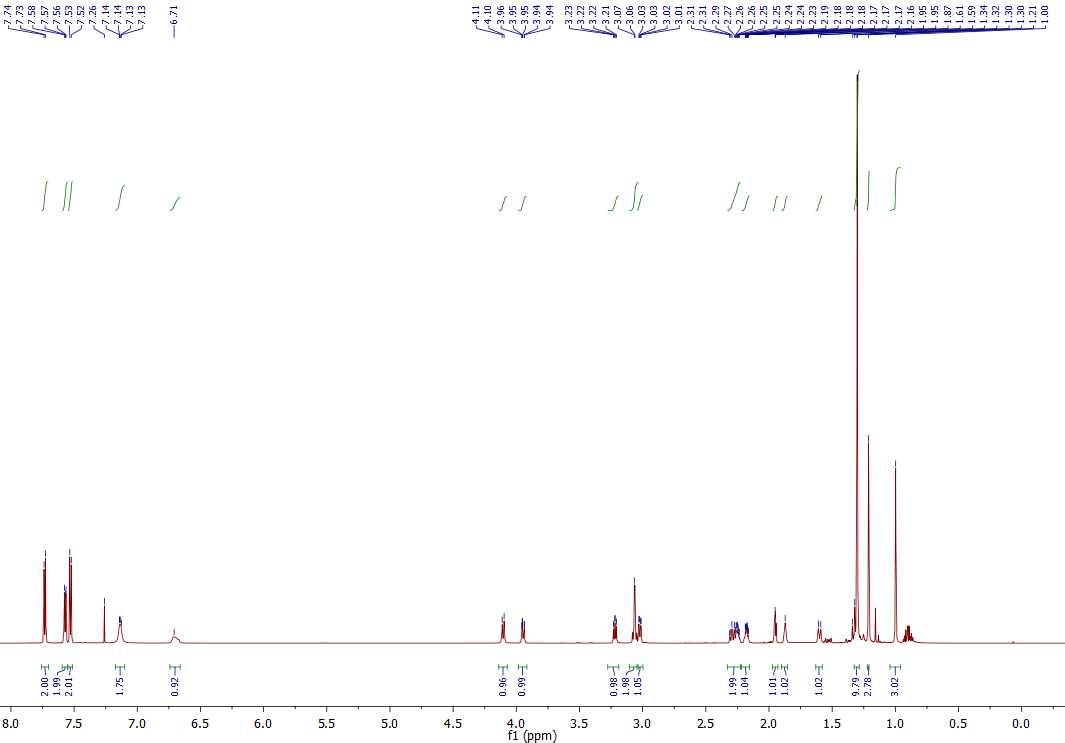


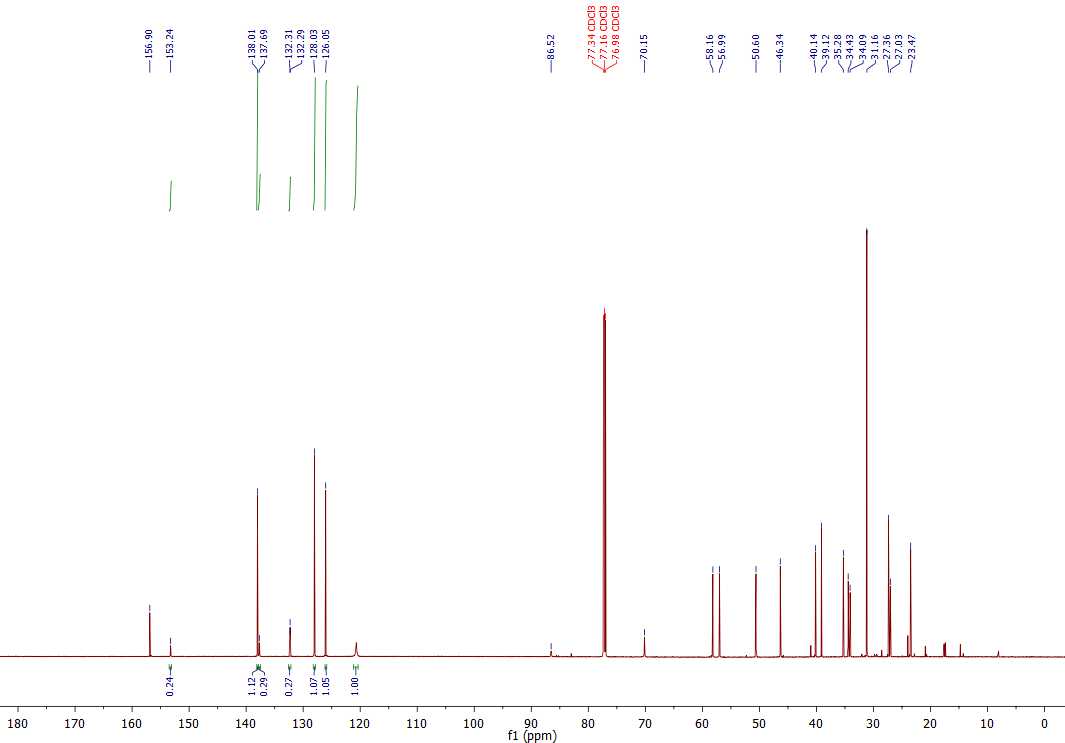


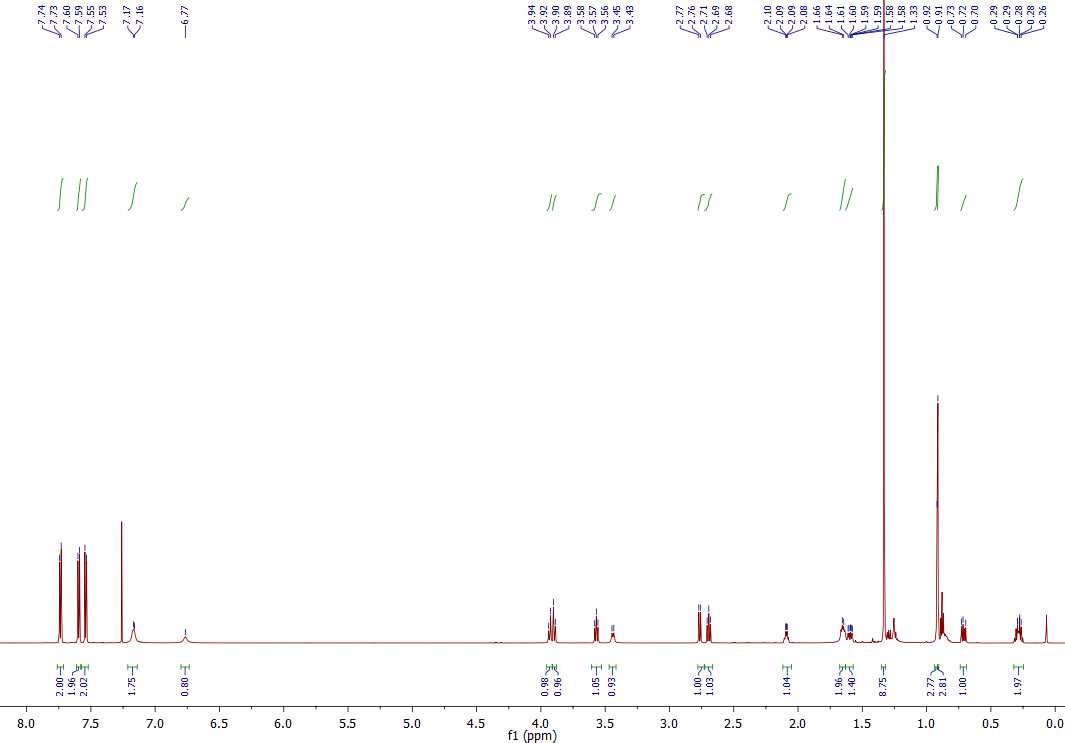


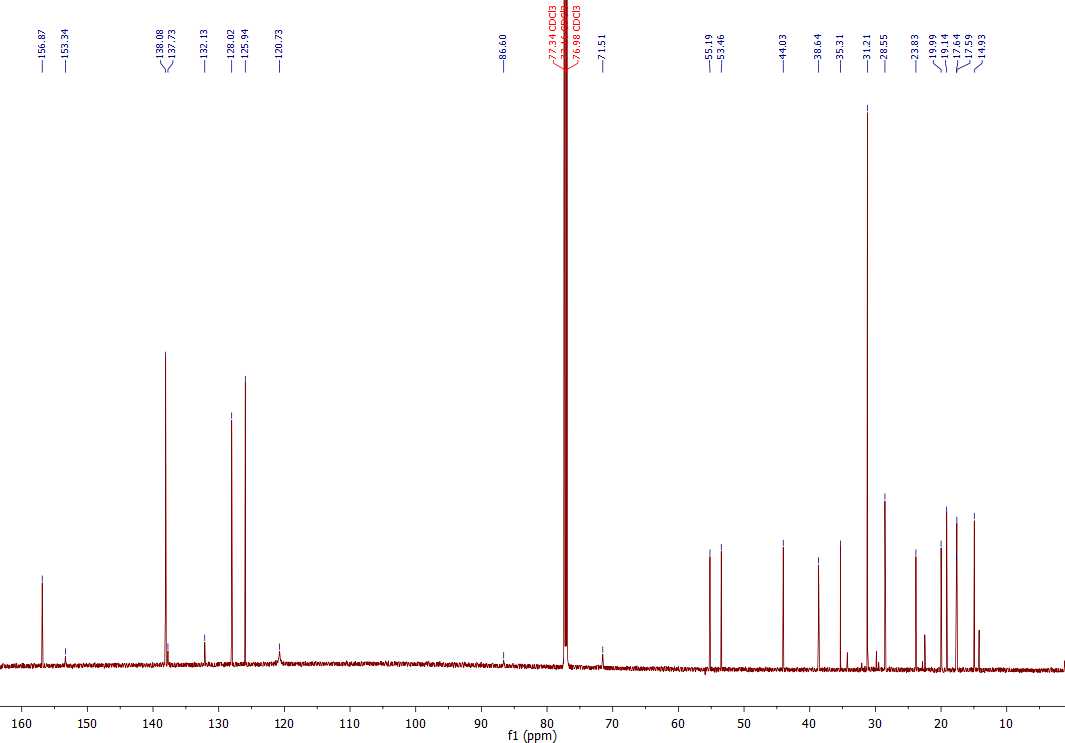


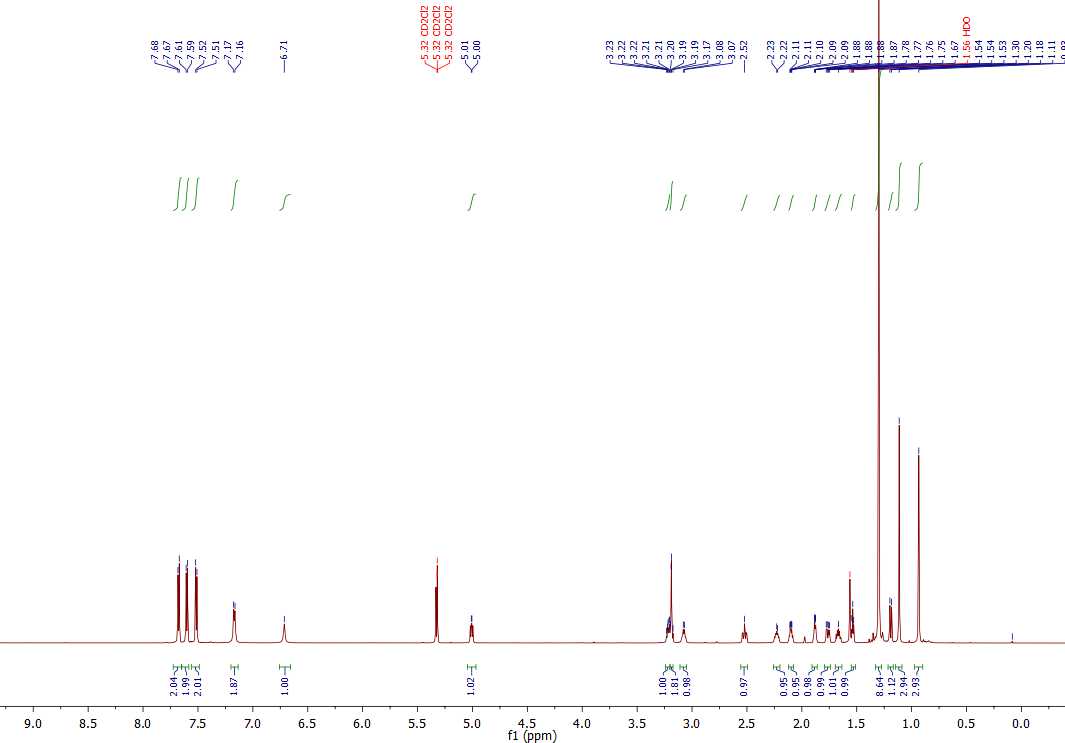


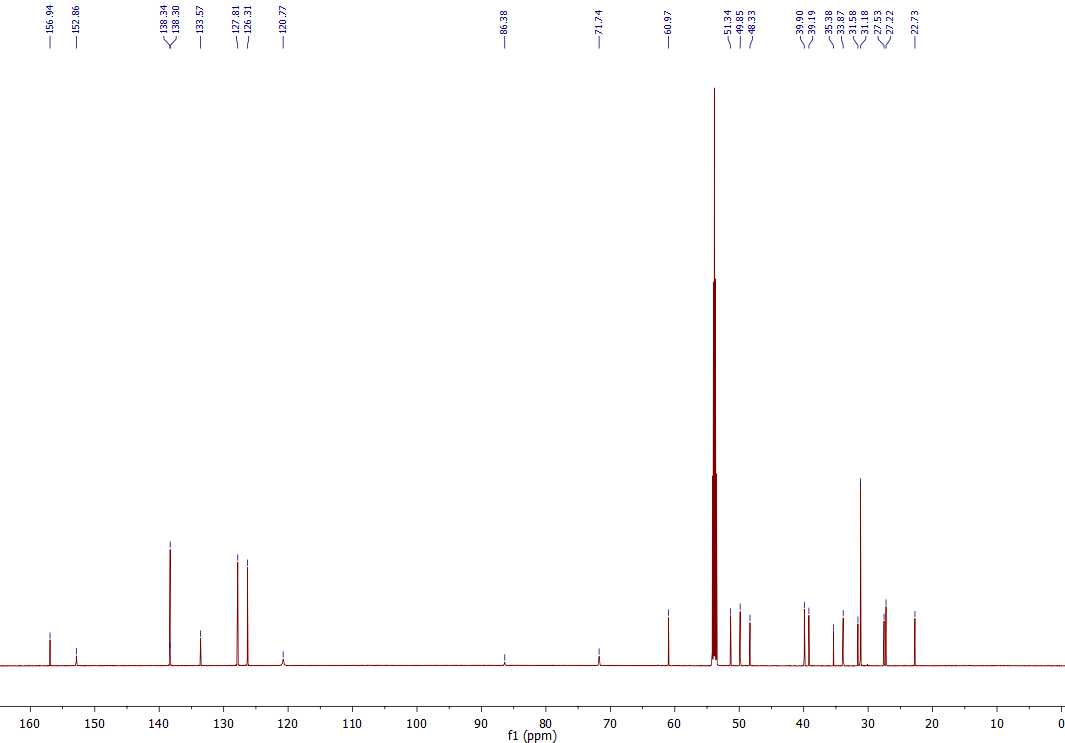


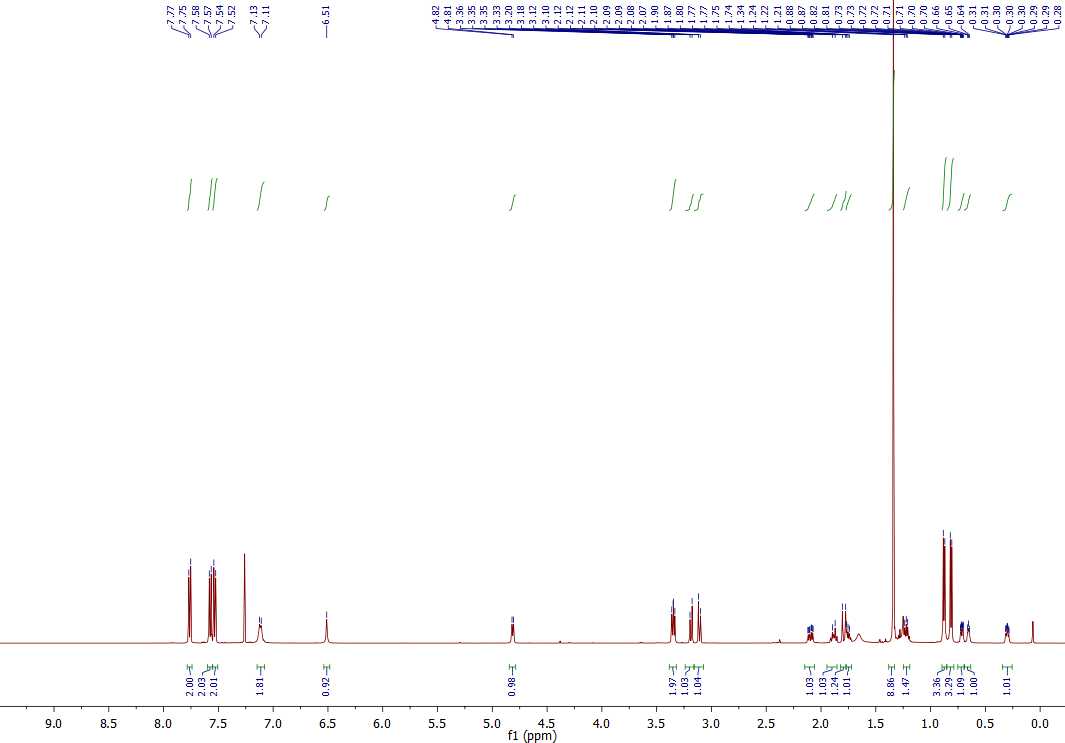


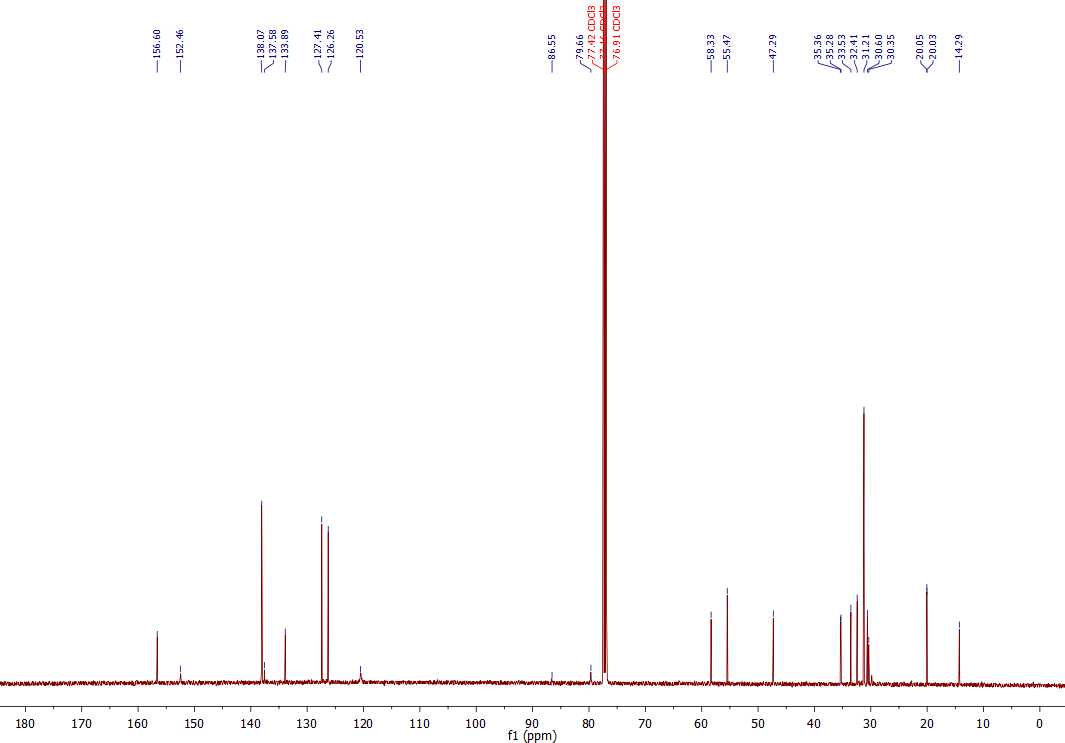


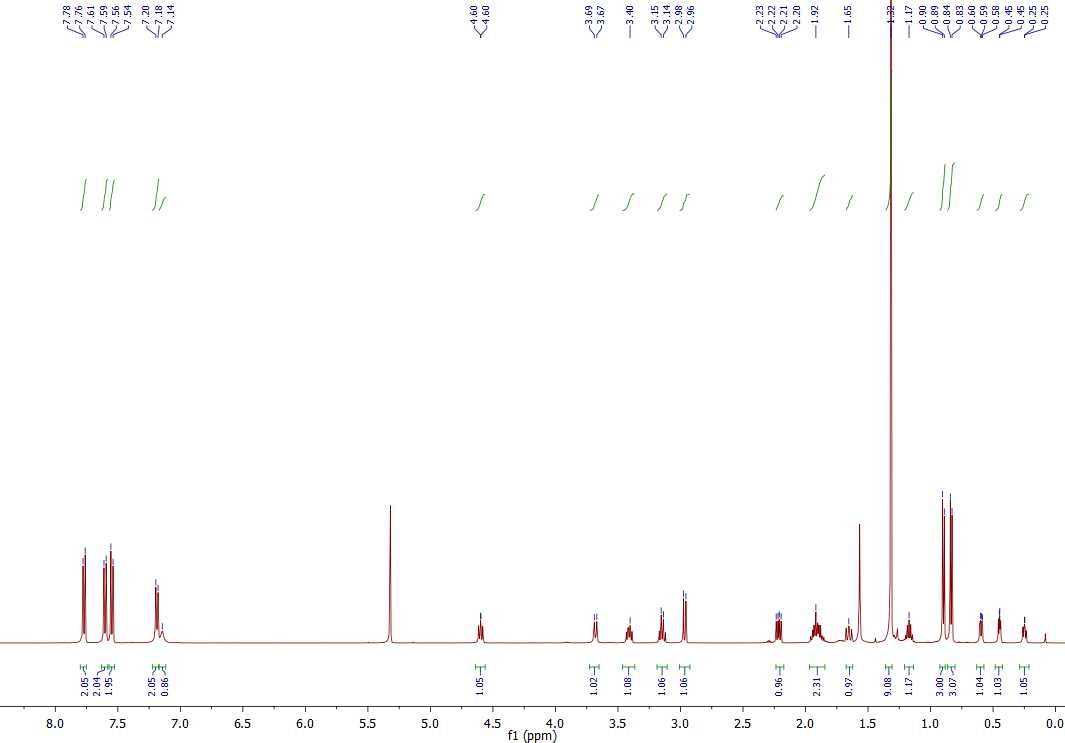


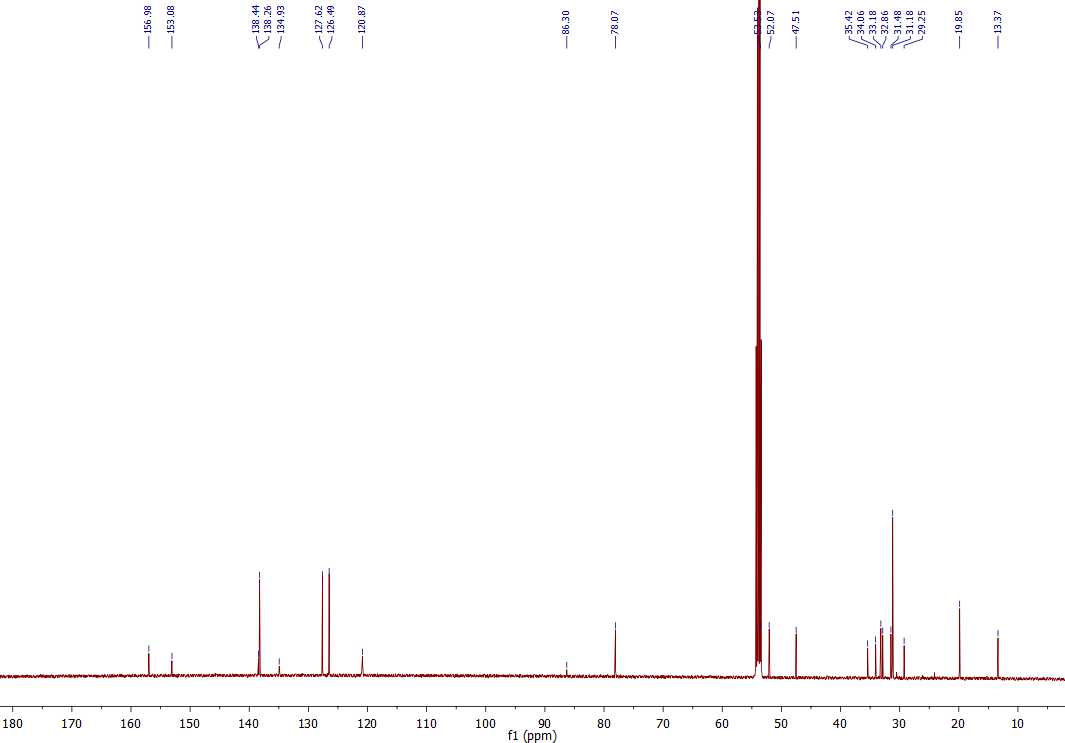


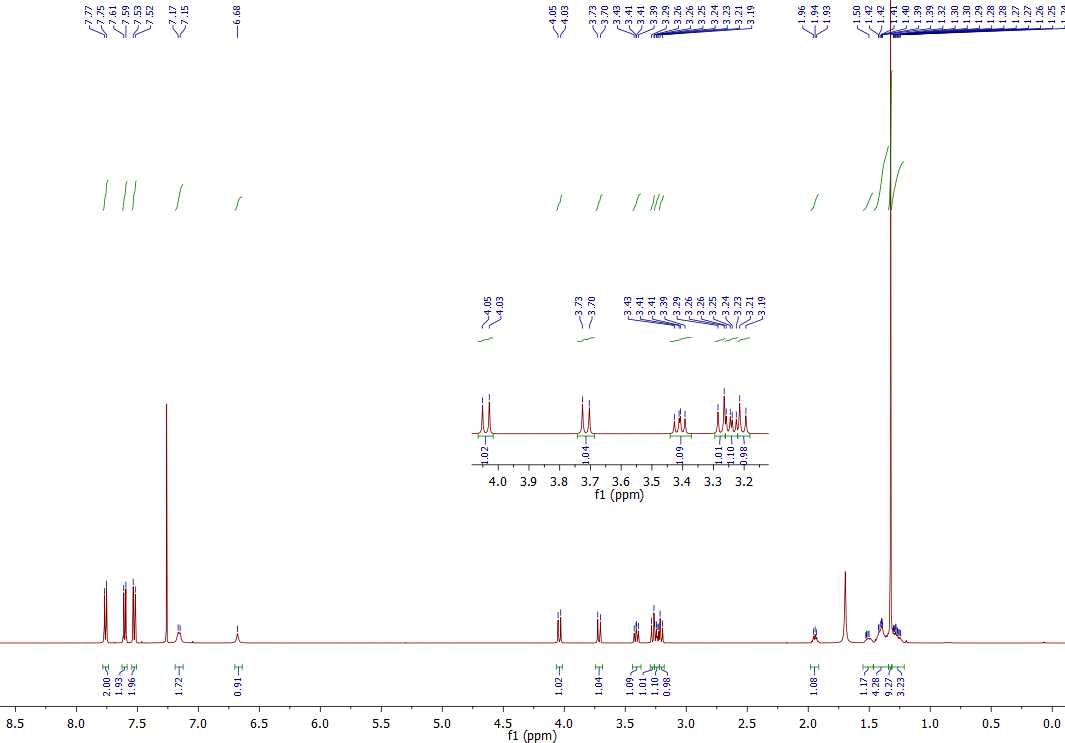


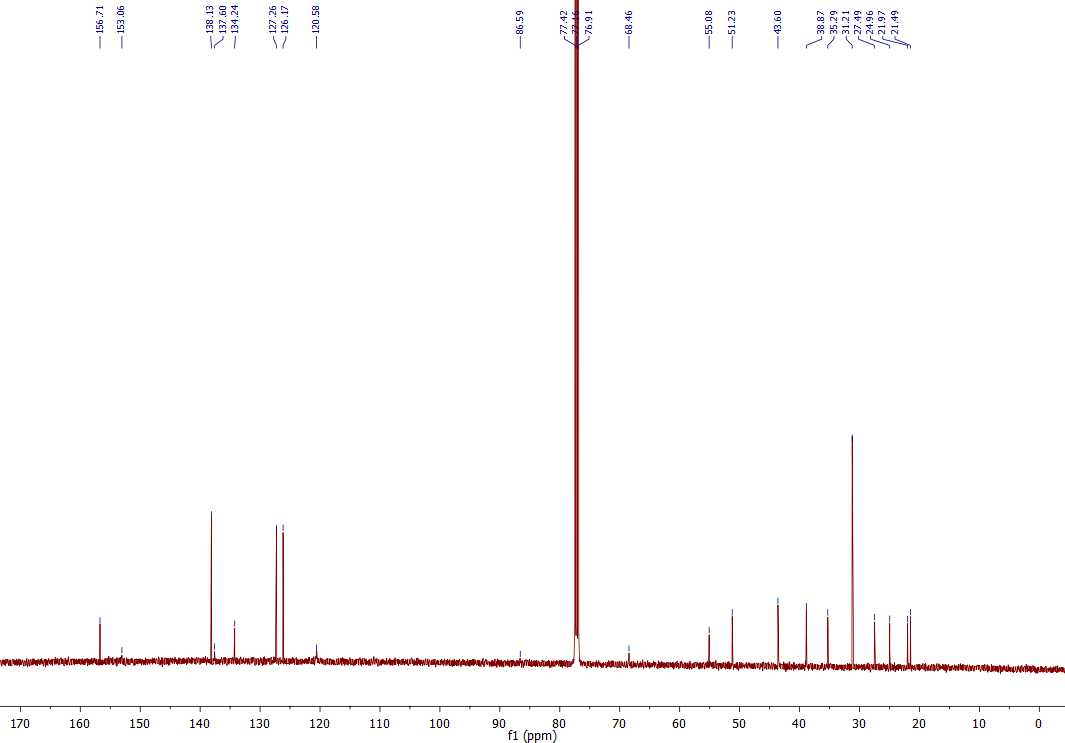


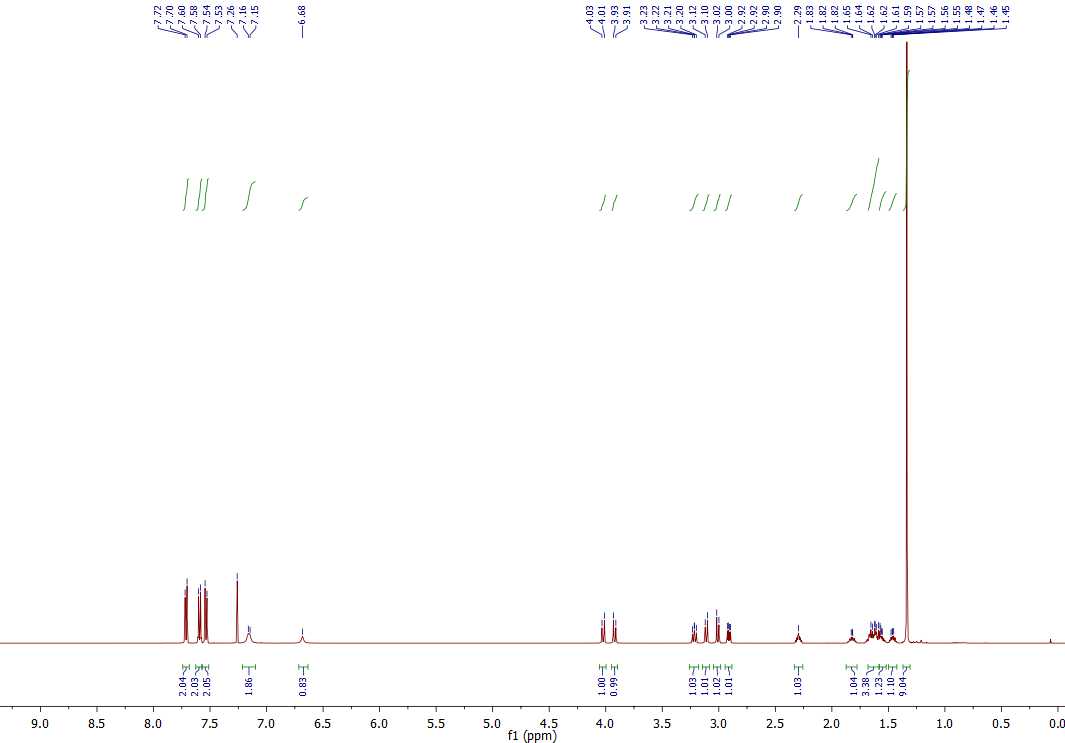


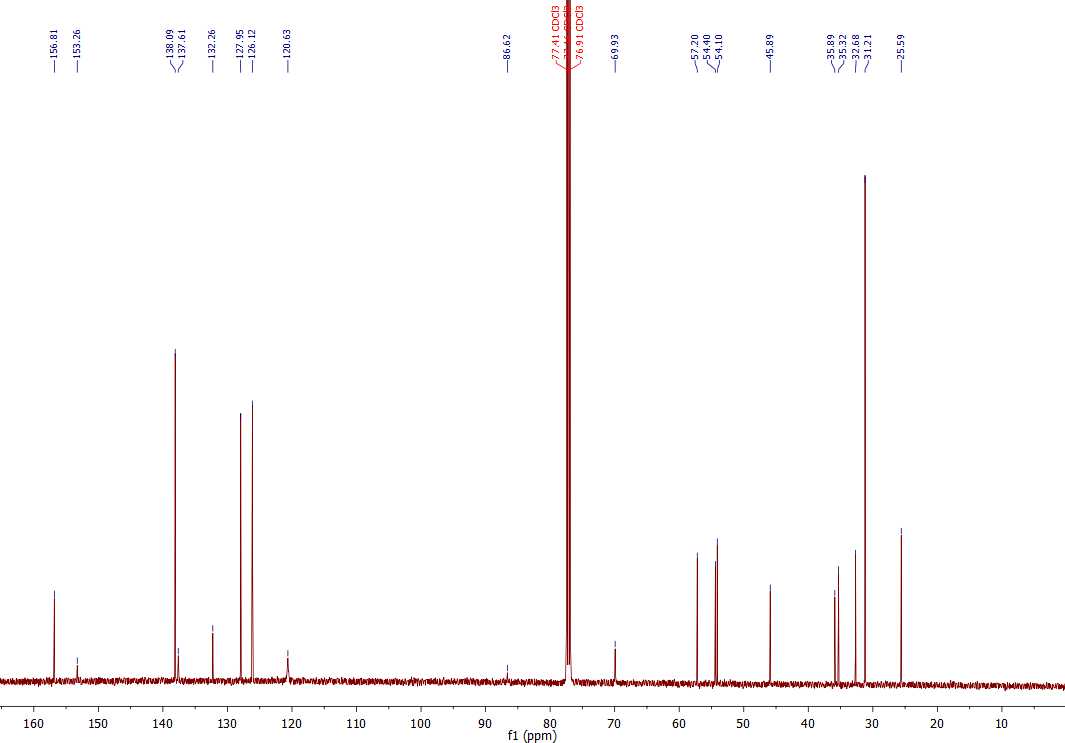


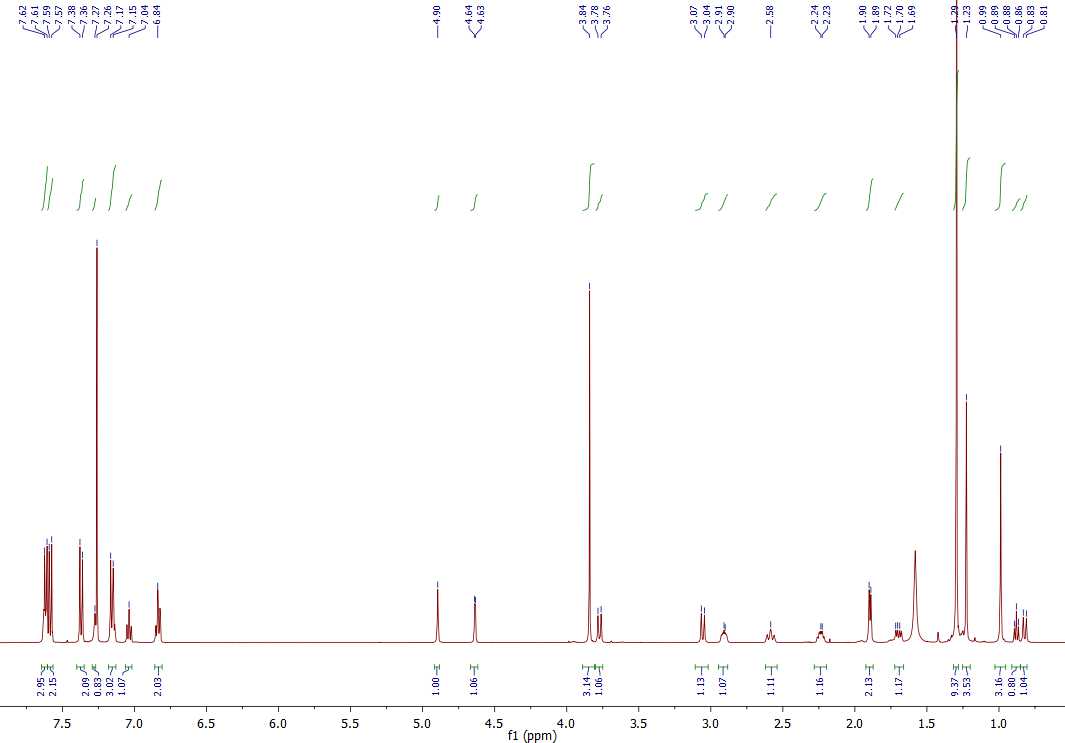


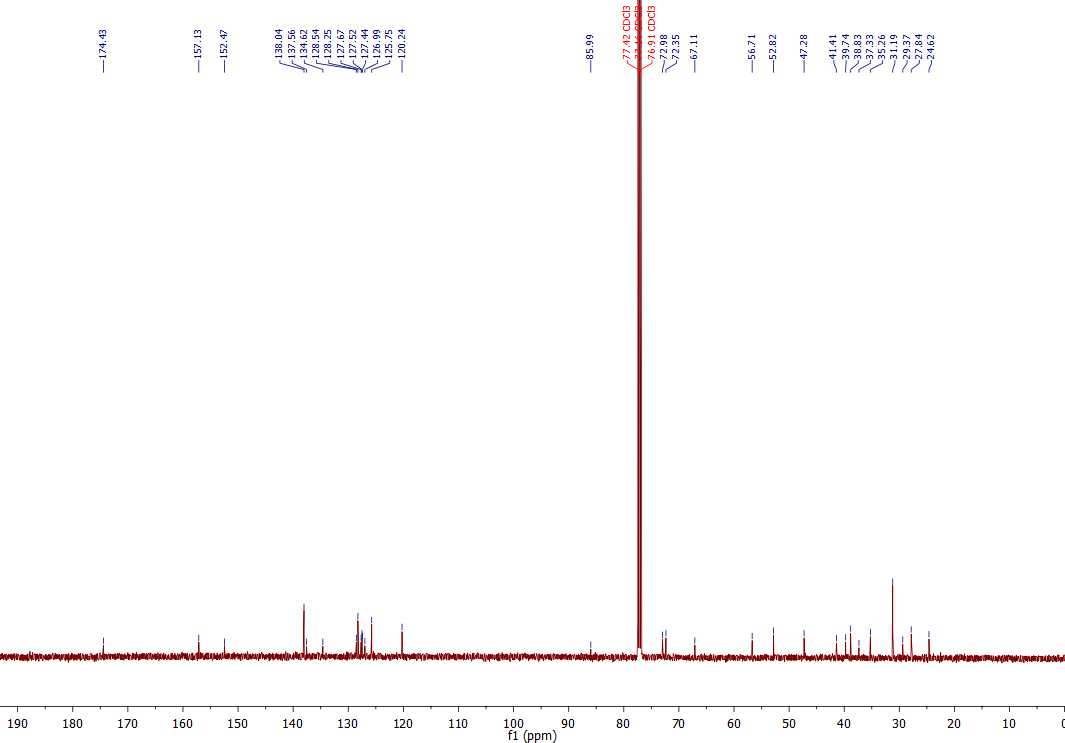


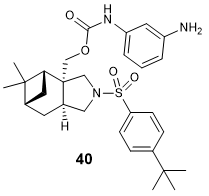


**
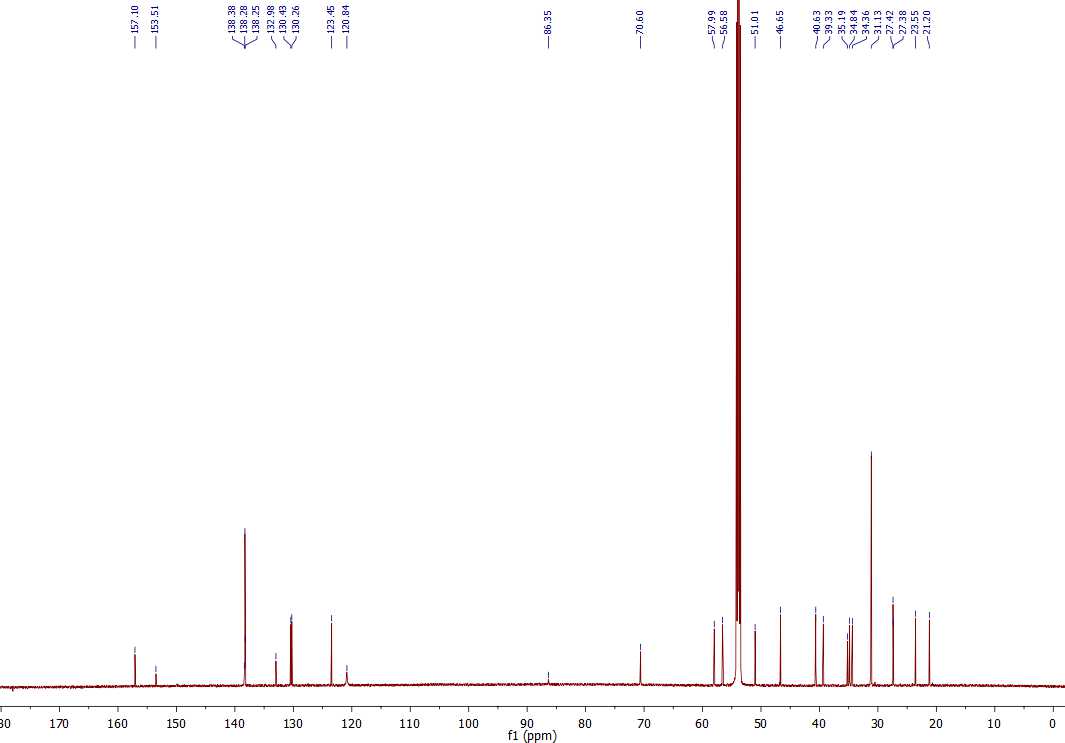
**

**
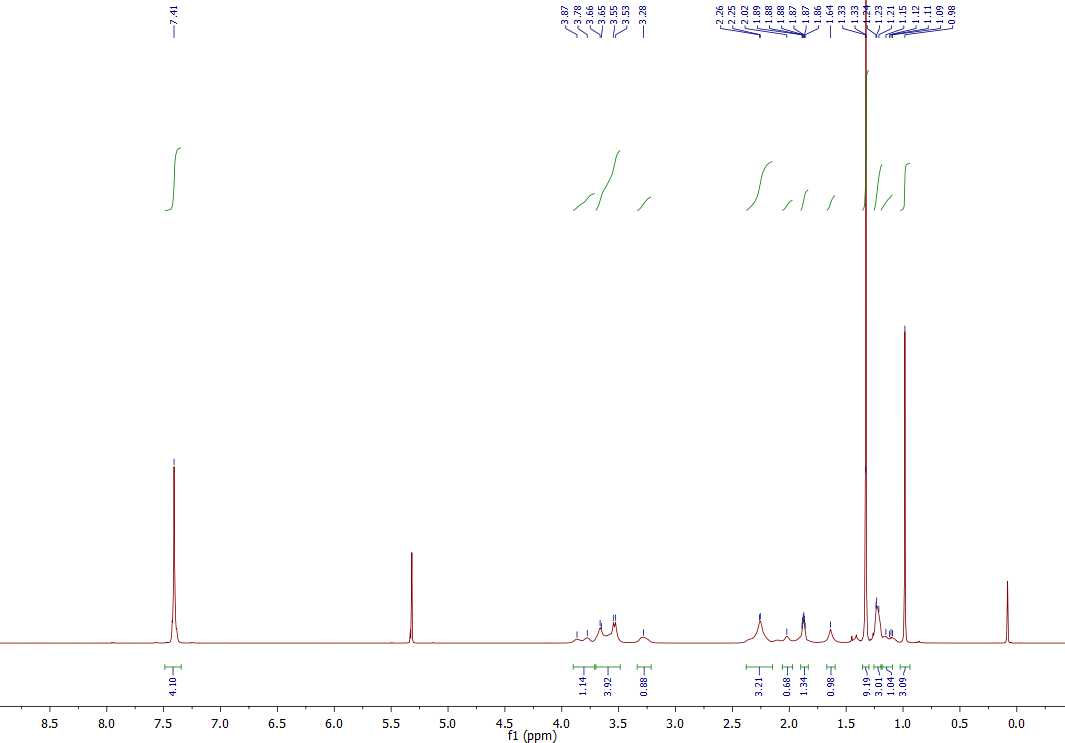
**

**
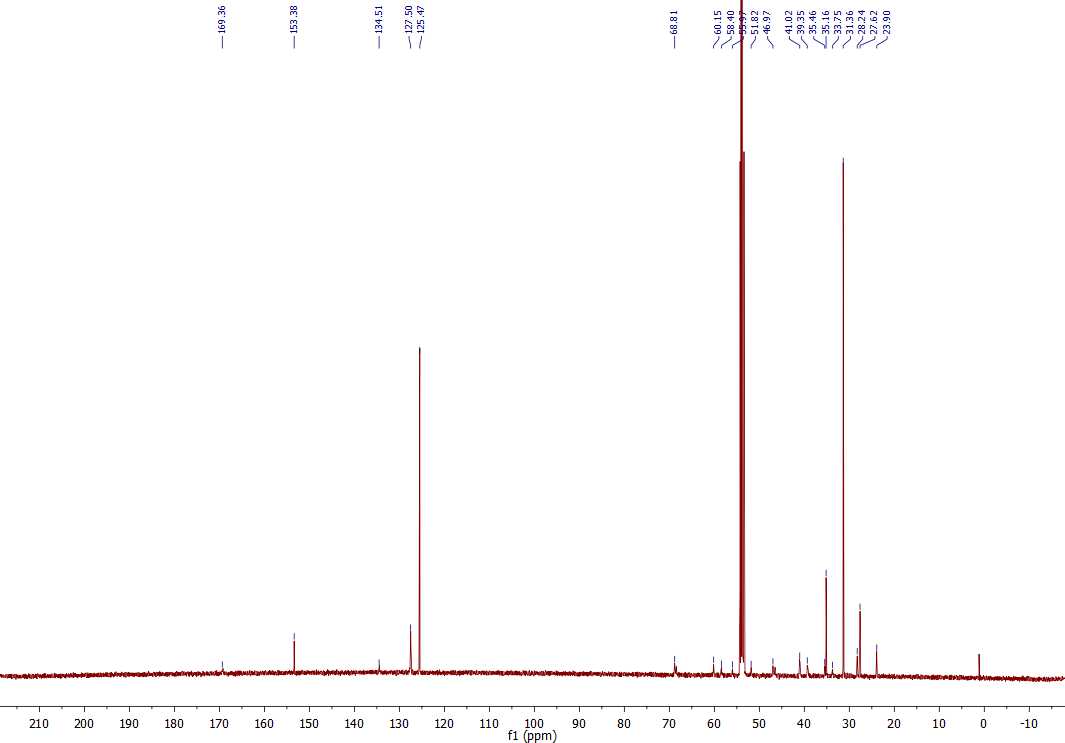
**

**
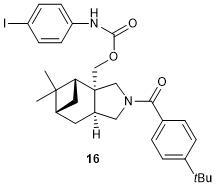
**

**
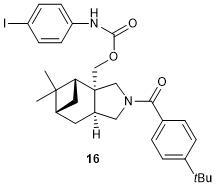

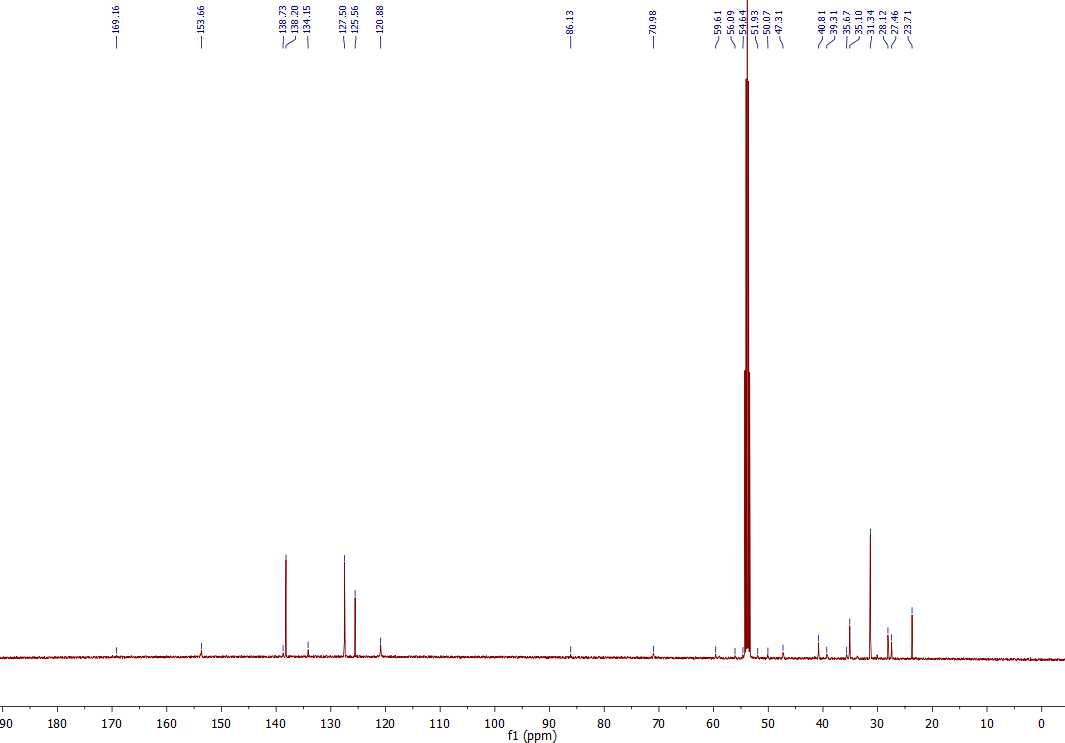
**

**
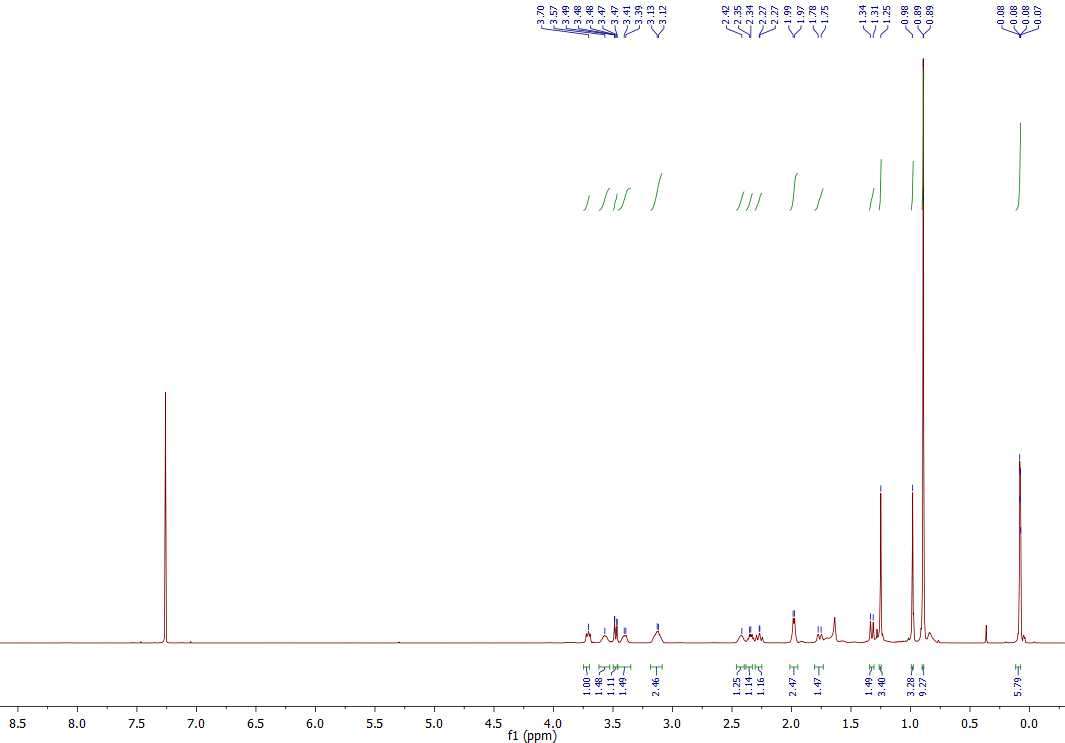
**

**
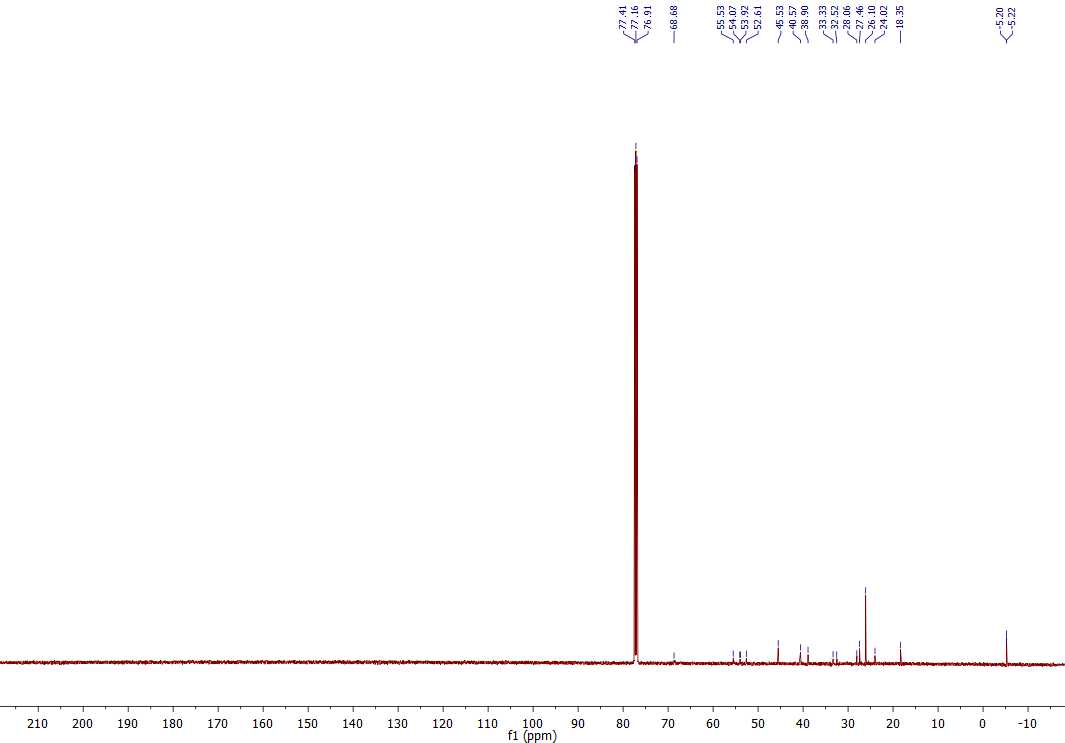
**
